# An Optimized Route to the Syringolin Natural Products Enables Combinatorial Synthesis of Selective, Bioactive Inhibitors of the *Plasmodium falciparum* 20S Proteasome

**DOI:** 10.1021/acs.jmedchem.5c03223

**Published:** 2026-04-01

**Authors:** Xilin Gu, Pavla Fajtova, Nicholas L. Yan, Aditi Saxena, Fan Fei, Jiyun Zhu, Eric Tse, Arthur Melo, Euna Yoo, Nathan H. Buchwald, Daniel R. Southworth, Matthew Bogyo, Joseph L. Derisi, Jason E. Gestwicki, Anthony J. O’Donoghue, Jason K. Sello

**Affiliations:** 1. Department of Pharmaceutical Chemistry, University of California, San Francisco, San Francisco, CA, 94158 USA.; 2. Skaggs School of Pharmacy and Pharmaceutical Sciences, University of California San Diego, La Jolla, CA, 92093,USA; 3. Institute for Neurodegenerative Diseases, University of California, San Francisco, CA, 94158 USA; 4. Chan Zuckerberg Biohub San Francisco, San Francisco, California,94158 USA.; 5. Current Address: Department of Immunology and Infectious Disease, Harvard T.H. Chan School of Public Health, Boston, MA, 02115 USA; 6. Department of Pathology, Stanford School of Medicine, Stanford University, Stanford, CA 94305 USA; 7. Current Address: Department of Biological Chemistry and Molecular Pharmacology, Harvard Medical School, Boston, MA, 02115 USA; 8. Current address: Center for Cancer Research, National Cancer Institute. Frederick, MD, 21702-1201 USA; 9. Department of Chemistry, Brown University, Providence, RI 02912, United States; 10. Department of Biochemistry and Biophysics, UCSF, San Francisco, California, 94143 USA.

## Abstract

The syringolin natural products are covalent inhibitors of the 20S proteasome that inspire therapeutic development. Here, we report a new route to the syringolins amenable to solution and solid-phase synthesis that overcomes a problematic macrocyclization. Exploiting our synthetic approach and substrate mimicry models for proteasome inhibition by the syringolins, we generated a collection of hypothetically selective inhibitors of the *Plasmodium falciparum* proteasome, which is an emerging target for anti-malarial drugs. We identified compounds from the library having high second-order rate constants for *Plasmodium* proteasome inhibition and nanomolar anti-parasitic activity. They exhibited selectivity for the *Plasmodium* proteasome over the human proteasome. We solved cryo-EM structures of an inhibitor bound to both 20S proteasomes, revealing key contacts favoring species-selective inhibition. Together, this work provides an improved route to syringolin analogs, sheds new light on substrate mimicry by the syringolins, and provides a structural basis for the pursuit of new anti-malarial drugs.

## Introduction

The 20S proteasome is a highly conserved, multi-catalytic protein complex that serves as the catalytic core of the 26S proteasome. The 26S proteasome is the central effector of the ubiquitin-proteasome system, which maintains cellular proteostasis by degrading misfolded, damaged, or regulatory proteins. This process governs critical cellular processes including cell cycle progression, transcriptional regulation, and apoptosis.^1^ Given its essential role in cellular homeostasis, the 20S proteasome has emerged as a compelling therapeutic target for a broad range of indications.^2^ The druggability of this enzyme was first established in the discovery of lactacystin, a natural product that has potent anti-cancer activity via inhibition of the 20S proteasome.^3^ Subsequently, the structures and activities of other natural products inhibitors of the proteasome (*e.g.*, epoximicin) inspired the development of analogs and completely novel scaffolds having clinical use or potential.^4, 5^ The latter category is exemplified by bortezomib, the first clinically useful 20S proteasome inhibitor. It has been used successfully for the treatment of relapsed/refractory multiple myeloma since 2003. More recently, newer 20S proteasome inhibitors like carfilzomib^6^ and ixazomib^7^ (derived from epoximicin and bortezomib, respectively), which exhibit marked improvements in potency and tolerability have been approved for use in oncology. Beyond oncology, the 20S proteasome has emerged as a promising therapeutic target in infectious diseases. Over the past two decades, inhibitors of the 20S proteasome in multiple pathogens have been developed in drug discovery programs,^2^ including *Plasmodium falciparum* (an etiological agent of malaria),^8–19^ *M. tuberculosis* (causative agent of tuberculosis),^20–28^ *Trichomonas vaginalis* (pathogen of trichomoniasis)^29–31^ and *Trypanosoma brucei* (causative agent of human African trypanosomiasis).^32^

The syringolins (and the related glidobactins) are a family of naturally occurring proteasome inhibitors that are attractive starting points for drug development (Figure 1A).^34^ They were originally isolated from the plant pathogen *Pseudomonas syringae pv. syringae* in 1998 and act as virulence factors via inhibition of plant proteasomes.^33, 35, 36^ They are selective and irreversible inhibitors of the 20S proteasome, making them valuable both as chemical probes and potential therapeutic leads.^34^ They stand apart from the epoxyketone natural products that covalently inhibit the 20S proteasome through substrate mimicry (*i.e.*, epoximicin and carmaphycin) in that they are macrocyclic and have an α,β-unsaturated enamide warhead (Figure 1B). Nevertheless, the syringolins also occupy the enzyme’s active site like a substrate. In a crystal structure of the syringolins in complex with the yeast 20S proteasome, Gröll and colleagues found that the syringolin macrolactam substituent structurally mimics the P1 residue of a substrate via occupancy of the active site’s S1 pocket, whilst the side of an amino acid residue in the peptidourea side chain is reminiscent of a substrates P3 position through binding in the S3 pocket of the active site (Figure 1B).^37, 38^ Although the syringolins form an adduct at all of the proteasome’s catalytic β subunits (*i.e*., β1, β2, and β5) in that structure, they exhibit high selectivity for the proteasome without significant reaction with other cellular proteins when incubated with mammalian cell lysates.^39^ The high degree of selectivity of the syringolins for the 20S proteasome, relative to other covalent inhibitors, has been deemed an asset in drug discovery efforts.

In the past 15 years, there have been multiple reports of the optimization of the syringolins for use as therapeutics. Indeed, based on their apparent binding mode, several syringolin analogs have been synthesized and evaluated for activity against malignancies like multiple myeloma^39–48^ and leukemia^49^ and tuberculosis.^23^ Some efforts have been focused on optimization of the macrocycle via ring contraction^50^ and expansion^45, 50^ and through strategic replacement of atoms within the ring.^48^ Others have examined alternatives to the peptidourea side chain like amides.^43, 44, 51, 52^ Additionally, efforts have been made to optimize the subunit selectivity via replacing substituents on the macrolactam and in the peptidourea side chain with structures that are reminiscent of the subunit’s preferred substrates.^40^ Successes in and lessons from the aforementioned reports justify efforts to exploit the syringolin scaffold in the development of clinically useful 20S proteasome inhibitors.

Despite on-going efforts to develop syringolin-based proteasome inhibitors, their synthesis remains challenging. The first synthesis of a syringolin natural product (*i.e*., syringolin B) was reported by Clerc and co-workers;^37, 53^ though successful, its length and inefficient transformations have likely precluded its use by other investigators to access structurally related molecules. Subsequent reports of syringolin syntheses by Stephenson^54^ and by Pirrung^55^ were markedly more efficient, but yields were highly dependent on the identity of the macrocyclization substrate. In the Stephenson synthesis of syringolin A, the macrocyclization of an olefinic substrate via an amidation was effected in only 15% yield.^54^ In the Pirrung syntheses of syringolin A and syringolin B (which has a single olefin in the macrolactam), they utilized a modified Horner-Wadsworth-Emmons (HWE) reaction for the key macrocyclization and the yields of the respective products were 81% and 65%.^55^ In a distinct strategy, Ichikawa and co-workers performed macrocyclization via an Ugi four-component reaction that suffered from low-yield and lack of stereochemistry control.^51^ The relative superiority of the macrocyclization via olefination route has rendered it as the choice reaction for chemical syntheses of syringolin analogs. Accordingly, our group used olefination for the syntheses of syringolin analogs designed as selective inhibitors of the *Mycobacterium tuberculosis* 20S proteasome^23^ and subunit-selective inhibitors of the human 20S proteasome.^49^ In our syntheses and in those described above, the yields of the macrocyclization were no greater than 60% and highly sensitive to the structure of the ring closure substrate.^23, 41–44, 49, 51, 52^ With respect to the latter point, we found that macrocyclization of a valinol-derived substrate occurred in 58% yield, whereas the same reaction for a substrate made from phenylalaninol resulted in a 19% yield (Table 1).^23, 49^ Substrate sensitivity and modest yields in the macrocyclization limited the practicality of synthesis of the natural products and diverse analogs thereof.

Herein, we describe novel synthetic routes for the preparation of syringolin analogs, the development of potent and selective *Plasmodium* 20S proteasome inhibitors, and the structural elucidation of their binding mode to the *Plasmodium* 20S proteasome via cryogenic electron microscopy (cryo-EM). More broadly, the work outlined here provides a platform through which pharmacologically useful species- and subunit-selective inhibitors of 20S proteasomes can be generated from the syringolin scaffold.

## Results

### Design of an improved route to syringolins

In a comprehensive analysis of the reported syntheses of syringolins, we postulated that the poor macrocyclization efficiency could be attributed to the planarity of the two secondary amide moieties common to every reactant. In principle, the planarity and rigidity of the secondary amides are either an enthalpic penalty for macrocyclization or a problematic determinant that conformationally separates the reactive centers. In light of these considerations, we proposed that strategic replacement of a secondary amide of the substrate with a less conformationally restricted tertiary amide would impart flexibility that facilitates propinquity of the reactive termini in the macrocyclization. This strategy is contingent on the post-cyclization conversion of the tertiary amide to the secondary amide found in all of the syringolin natural products and analogs.^34^ Thus, we selected *N*-dimethoxybenzyl (DMB) tertiary amides as moieties because the *C*-*N* bond can be cleaved under mildly acidic conditions to yield the desired secondary amide. Our proposal was justified by precedents in which macrocyclizations are highly dependent on the presence of DMB-tertiary amides in the substrates.^56, 57^

To determine if installation of a DMB moiety could facilitate macrocyclization, we conducted model studies using a phenylalaninol-derived substrate that we previously reported to cyclize inefficiently (Reaction a, Table 1).^23, 49^ We used a Dess-Martin oxidation of the primary alcohol to yield the aldehyde reactant of the HWE macrocyclization. Because the macrocyclization substrate has two different secondary amides that could be sites for DMB installation (Table 1), we synthesized two substrates having a DMB moiety on either of the amides and compared their efficiencies for macrocyclization. For the substrate **1b** wherein R_1_ = H and R_2_ = DMB, we were surprised to recover the aldehyde intermediate and not the desired macrocycle from the reaction. The simplest explanation for the macrocyclization failure is that the DMB moiety sterically hindered the proximal aldehyde such that the HWE reaction was disfavored. In contrast, the substrate **1c** in which R_1_ = DMB and R_2_ = H underwent ring closure in a yield four-times higher than a macrocyclization with a substrate lacking a DMB moiety (19% yield versus 83% yield) (Table 1, reactions a and c). These observations affirmed our prediction that substrates having a DMB tertiary amides will undergo more efficient ring closure and are consistent with the structure-dependence of macrocyclization substrates for this family of molecules.

To further investigate the yield enhancement by a tertiary amide, we synthesized a substrate having a methyl substituent rather than a DMB moiety (Table 1, reaction d) and subjected it to the ring closure conditions. Interestingly, the yield of the reaction was markedly lower than that with the DMB substrate, yet slightly better than that in a reaction with a substrate having secondary amides. The methyl group does not exert the same influence as the DMB moiety on reaction yield, suggesting that the bulkiness and conformational influence of the former in the tertiary amide are insufficient to exert a strong influence over the macrocyclization.

To evaluate the general applicability of the DMB group in promoting macrocyclization, we prepared substrates from a range of amino alcohols, including the valinol building block used in syntheses of syringolin natural products (Table 2).^54, 55^ Notably, all substrates having the DMB moiety exhibited substantially higher macrocyclization yields than those without, indicating that the strategy overcomes the substrate-sensitivity.

After confirming that the DMB moiety facilitates macrocyclization, we sought to effect its removal without decomposition of the acid-labile macrolactam. We evaluated several acidic conditions with and without scavengers for the benzyl carbocation (see supporting information Table S1). Initially, we found that aqueous trifluoroacetic acid (TFA) alongside the cation scavenger triisopropyl silane effected removal of both the DMB and Cbz protecting groups in nearly quantitative yield. However, this condition did yield trace amounts of an *N*-benzyl adduct at the alpha-amine group, which proved challenging to remove in subsequent steps. We were more gratified to find that co-treatment of the macrolactam with TFA and the carbocation scavenger thioanisole resulted in quantitative removal of both the DMB and Cbz groups, yielding the desired macrolactam.

Owing its proximity to the cyclization site and its potential to influence the conformational preferences of the substrate akin to the aforementioned DMB, we predicted that the identity of the alpha-amino protecting group could influence macrocyclization efficiency. Thus, we prepared substrates having various carbamates protecting groups in place of the Cbz common to all of the substrates in the ring closures described above. As summarized in Table 3, we observed clear correlations between protecting group structure and cyclization efficiency. Relative to a substrate with the Cbz protecting group, the cyclization yield was the lowest wherein a substrate having a bulky Boc protecting group on lysine (**5f**, Table 3). The apparent inverse correlation between steric bulk and macrocyclization efficiency held with substrates having other less sterically encumbered carbamates. Interestingly, the stereochemistry of the carbamate has a substantial influence over the yield, as evidenced by reactions **c** and **d** in Table 3, which have *N*-benzyl carbamates derived from chiral secondary alcohols. Having optimized both the DMB deprotection conditions and choice of the carbamate, we were able to realize a solution-phase synthesis of syringolin B methyl ester (Scheme 1A).

The success of the DMB-based macrocyclization strategy prompted us to adapt our solution-phase route for syntheses of syringolin B and analogs thereof to a solid-phase format. To explore this possibility, we prepared 5-(4-formyl-3,5-dimethoxyphenoxy)pentanoic acid which is analogous to the aldehyde precursor of the DMB moiety, yet has a carboxylic acid handle that enables attachment to solid-phase synthesis resins having amines. From the corresponding aromatic aldehyde functionalized resin, we envisioned an efficient and modular synthetic route towards syringolins that leverages the intermediacy of a tertiary amide ring closure substrate. In pilot experiments, we pursued solid-phase synthesis of syringolin B methyl ester by first coupling 5-(4-formyl-3,5-dimethoxyphenoxy)pentanoic acid to a commercially available 4-methylbenzyhydrylamine (MBHA) resin having a 0.6 millimole/gram loading. Next, we anchored the ε-amine of α-*N*-Fmoc lysine *O*-allyl ester to the MBHA resin via reductive amination and subsequently acylated the amine with 2-(diethoxyphosphoryl)acetic acid. Then, palladium-mediated deprotection of the allyl ester yielded a carboxylic acid, which was subsequently coupled to L-valinol. The primary alcohol in the product was oxidized to an aldehyde using Dess–Martin periodinane enabling macrocyclization via a HWE reaction promoted by Zn(OTf)₂ in the presence of TMEDA and TEA. Following cyclization, the Fmoc protecting group of the lysine residue was removed using 4-methylpiperidine and the unmasked alpha amino group was coupled with Fmoc-Valine using HATU. Removal of the Fmoc group from valine set the stage for completion of the total synthesis via urea formation in the reaction of the α-amine with a commercially available isocyanate derived from valine. In the final step, the resin was treated TFA to release the desired product (syringolin B methyl ester) which was easily purified by column chromatography (Scheme 1B).

The success of our synthesis of syringolin B methyl ester through a 10-step synthesis from the aromatic aldehyde was tempered by the 15% overall yield. The low yield prompted us to carefully analyze the cleavage reaction and we found that syringolin B methyl ester was accompanied by a dimeric species in a ~1:1 ratio. The extent of dimer formation suggested that the loading of the MBHA resin (loading 0.6 millimole/gram) was problematically high. Accordingly, we planned to execute the same synthetic route towards syringolin B on a lower loading resin. Because a lower loading form of the MBHA resin was not readily available, we elected to use an amine-terminating Tentagel^®^ resin (loading 0.26 millimole/g) and coupled it with 5-(4-formyl-3,5-dimethoxyphenoxy)pentanoic acid, as was the case for the MBHA resin. Execution of the syringolin B synthesis on the Tentagel^®^ resin generated the desired product in 30% yield along with the dimer in 15% yield. We cannot account for all the differences in the physical properties of the resins, but it appeared that the lower loading correlated with a more favorable ratio of the desired product to dimer.

In any case, we developed a superior synthetic strategy for the preparation of syringolins in both solution- and solid-phase. Its simplicity and comparatively high yield will enable syntheses of syringolin analogs in numbers and on scales that have been difficult to achieve to date.

### Syringolin Analogs Have Potent Anti-*Plasmodium* Activity

Having a practical route to syringolin analogs, we contemplated indications for which bespoke proteasome inhibitors could have significant therapeutic value and recognized the potential of molecules targeting the *Plasmodium falciparum* 20S proteasome as anti-malarial agents.^8–18^ Because much is known about both the means by which syringolins inhibit 20S proteasomes and the structural bases of inhibition of the *P. falciparum* proteasome, we were well-poised to design and synthesize inhibitors of the *P. falciparum* enzyme based on the syringolin scaffold. The syringolins are known to be substrate-mimicking and covalent inhibitors of the proteasome. In the Gröll model for proteasome inhibition,^33^ the substituent on the macrolactam mimics the P1 residue of the substrate by occupying the S1 pocket while the amino acid appended to the macrolactam mimics a substrate’s P3 residue and fits into the S3 pocket (Figure 1B). Peptidomimetic inhibitors incorporating a D-amino acid in the P3 have been shown to be highly selective for the *P. falciparum* proteasome over the human constitutive proteasome.^17, 18^ Therefore, we hypothesized that incorporating the D-amino acid into the same position syringolin scaffold would generate a species-selective inhibitor. Accordingly, we synthesized compound **A-1**, which differs from syringolin B methyl ester in that it has a D-valine residue in place of the native L-valine appended to the macrolactam (Table 4).

We compared the activity of syringolin B methyl ester and **A-1** in assays with human cells and *P. falciparum*. Interestingly, both compounds exhibited anti-*Plasmodium* activity, but only syringolin B methyl ester was cytotoxic to human foreskin fibroblasts (HFFs). Indeed, the selectivity indices of syringolin B methyl ester and **A-1** were 16 and 73, respectively, in assays with parasite versus those HFFs (Table 4). The enhanced selectivity of **A-1** did come at the expense of potency against *P. falciparum* (*i.e*., two-fold less potency relative to syringolin B methyl ester); nevertheless, our observations mirror those previously reported for selective, peptidomimetic *Pf* proteasome inhibitors having a D-amino acid at the P3 position.^17, 18^

Our strategy for the efficient solution and solid-phase synthesis of the syringolins and the observation that a D-amino acid at the P3 position confers species selectivity for the *Pf* proteasome prompted us to synthesize a small library of analogs. We envisioned a collection having various aliphatic, aromatic, and polar residues at both the P1 and P3-mimicking positions on the syringolin scaffold. Accordingly, we designed and synthesized a series of eighteen syringolin analogs incorporating three different D-amino acid residues at the P3 position and six different variants of amino side chains at the P1 position. We note that compounds with the acid-labile glutamate *tert*-butyl ester (**B1**-**B6**, Table 5) were prepared in solution to avoid the acid-cleavage required for solid-phase synthesis (Scheme 1B). Initially, the anti-parasitic activity of these compounds was assessed in assays with *P. falciparum* W2 cultures at a concentration of 100 nM (Table 5). The assay readout was the extent of parasitemia (*i.e*., growth of *Plasmodium*). The three most potent compounds had the side chains of either 3-fluorophenylalanine or phenylalanine at P1 and D-valine(**A-4**), D-glutamate *tert*-butyl ester(**B-5**), or D-phenylalanine(**C-5**) at P3 (Table 5). Upon further analysis, we noted certain structure-activity relationships among the compounds. The tolerance for multiple amino acids at the P3 position stood in sharp contrast to stricter requirements at the P1 position. Indeed, two of the three most potent compounds had the 3-fluorophenylalanine side chain at P1 (**B-5** and **C-5**). Curiously, we do note that **A-4** having the side chain phenylalanine at the P1 position was more active than an analog having 3-fluorophenylalanine at the same position (**A-5**). To gain further insights into the structure activity relationships, we tested the three most potent inhibitors from the collection of eighteen syringolin analogs (*i.e.,* **A-4, B-5** and **C-5**) alongside **A-1** and **A-5** in multi-point dose response parasitemia assays with *P. falciparum* W2 to determine their EC_50_ values (Table 6). Remarkably, the most potent compound, **C-5**, had an EC_50_ of 20.1 nM, which is comparable to the single-digit to mid-nanomolar potencies reported for more extensively optimized *Plasmodium* proteasome inhibitors.^9, 11, 17^

### Syringolin Analogs are Selective for the β5 Subunit of the *Pf* Proteasome.

While the anti-parasitic activity of the syringolin analogs was promising, we wanted to assess the activity of the most potent analogs in *in vitro* biochemical experiments to affirm their capacity to inhibit the proteasome. Initially, we measured the analogs’ second-order rate constants for inhibition of the β5 subunit of the purified *Pf* proteasome and found that C-5 was the potent compound (53,300 M^−1^s^−1^), whilst the least active compound was A-1 (1,570 M^−1^s^−1^) (Table 6). From these results, the compounds having D-glutamate-*tert*-butyl ester (**B-5**) and D-phenylalanine (**C-5**) appear to be more active than the analog with D-valine (**A-5**) at the same position. In parallel assays that measure inhibition of the β2 and β1 subunits, we found that there was no activity against β2 and only slight inhibition of β1 at 3 μM (see supporting s S2), indicating pronounced selectivity for the β5 subunit. Notably, differences between biochemical inhibition and cell-based anti-parasitic activity were observed for closely related analogs, such as **A-4** and **A-5**. Although **A-5** exhibits β5 inhibition comparable to that of **A-4** in biochemical assays, its reduced anti-parasitic potency could reflect differences in cellular penetrance rather than intrinsic differences in proteasome inhibition.

We speculate that the strong bias towards the β5 subunit is based on complementarity between the chymotrypsin-like S1 pocket of the enzyme and the aromatic moiety on **C-5**’s macrolactam. To clarify the basis of selectivity, we solved a 2.6 Å cryo-electron microscopy (cryo-EM) structure of the *Pf* 20S proteasome in complex with **C-5**. We first observed clear density corresponding to the inhibitor connected to Thr1 of the β5 subunits only, affirming the observed subunit-selectivity (Table 5 and Figure 2, A-C). Moreover, we found that the *m*-fluorobenzyl moiety of **C-5** occupies the S1 pocket formed exclusively by the β5 subunit (Figure 2D). Interestingly, the structure revealed other facets of peptidomimicry that diverged from the structure of the syringolin B natural product in complex with the yeast 20S proteasome.^33^ We found that the side chain of the D-phenylalanine in **C-5**’s peptidourea moiety is oriented towards the solvent-exposed inner chamber of the 20S proteasome instead of binding the S3 pocket formed by the β5 and β6 subunits. The S3 pocket in *Pf* 20S is occupied by the ureido moiety of **C-5**, including the constituent valine methyl ester. The apparent orientation of the terminal valine of the peptidourea side chain of **C-5** into the S3 pocket of the *Pf*20S also stands in sharp contrast to the syringolin B-yeast 20S proteasome structure wherein the analogous pocket is occupied by the valine residue appended directly to the macrolactam. Notably, the differences in side-chain orientation between **C-5** and syringolin B are aligned with opposing stereochemistry of the amino acid appended to their macrolactams (*i.e*., *R*- configuration in the former and *S*-configuration in the latter). It is intriguing that inhibitors sharing the same scaffold appear to mimic proteasome substrates in different fashions.

The high resolution of our cryo-EM structure also revealed key contacts that might explain potency. Specifically, we found that the amide of the macrolactam distal to the catalytic threonine appears to be both a hydrogen bond acceptor and donor with the backbone of Ser21 and Gly47, respectively (Figure 3A). The *m*-fluorobenzyl substituent on the macrolactam occupies the hydrophobic S1 pocket formed by residues Ala20, Val31, Lys33, Met45, Ala49 and Cys52 (Figure 3B). Interestingly, although important for selectivity and potency, the D-phenylalanine does not appear to make direct contacts with the enzyme. Rather, the aromatic ring is positioned directly over the proximal amide moiety in **C-5** that forms hydrogen bonds with the backbones of Ser21 and Ala49 (Figure 3C). We speculate that the D-phenylalanine likely acts as a hydrophobic cover, shielding these hydrogen bonds from competing solvent interactions. Finally, the ureido group and the valine methyl ester in **C-5** make several hydrogen bonds, particularly with the side-chains of residues Ser27 of β5 and Asp153 and Ser157 of β6 (Figure 3D). Those moieties of **C-5** also occupy the S3 pocket formed by residues Met22, Ser27, and Val31 of β5 and Ser15, Asn151, Tyr152, Asp153, and Cys159 of β6 (Figure 3E). Together, these findings help explain the improved potency of **C-5** and provide bases for further optimization.

### Syringolin Analogs Exhibit Species-Specific Inhibition *in vitro*

*Pf* proteasome inhibitors will only be viable as therapeutics if they have little to no activity against the human 20S proteasome and low toxicity to human cells. We tested the most potent compounds in the *Pf* proteasome inhibition and parasitemia assays for toxicity to HFFs and found that they had very low toxicity to human cells (Table 6). The species-selectivity for killing (*Pf* versus HFF is >25,000) was exceptional relative to other *Pf* proteasome inhibitors that have been reported in the literature.^8–12, 14, 15, 17, 18^ Importantly, we noted that the same compounds are also weaker inhibitors of purified human 20S constitutive proteasome (Table 6). Indeed, the second-order rate constants for inhibition of the human β5 subunit range from 11- to 45-fold lower than those observed in *Pf* proteasome inhibition assays, which is consistent with the low toxicity to human cells. In contrast, preferential inhibition of the β5 subunit of the human 20S immunoproteasome maxed out at only 2-fold among the *Pf* proteasome inhibitors (see supporting information Figure S2,Table S2). Our finding that species-selectivity indices in proteasome inhibition assays were lower than those in the cytotoxicity assays suggest that *P. falciparum* is intrinsically more sensitive to perturbations in proteasome function than HFFs. These observations are consistent with reports that *Plasmodium* is hyper-sensitive to proteasome inhibition.^17, 18^

It is also noteworthy that differences in cytotoxicity between closely related analogs appears to reflect differences in subunit selectivity rather than the extent of β5 inhibition alone.^58^ For example, syringolin B methyl ester exhibits substantially greater toxicity toward HFFs than A-5, despite similar levels of inhibition of the human β5 subunit under the conditions tested. However, we observed that the more cytotoxic syringolin B methyl ester inhibited the β1, β2, and β5 subunits of the h20S constitutive proteasome, whereas A-5 is selective for β5 of the same enzyme (Figure S2).

To gain insights into the structural basis of species-specificity, we determined a cryo-EM structure of **C-5** in complex with the constitutive h20S proteasome at 2.5 Å resolution. As with the *Pf* 20S structure, we observe the formation of adducts selectively at the β5 proteolytic sites (Fig 4A-C). Curiously, the conformations of both the macrolactam and the peptidourea side chain of **C-5** are quite different in the active sites of the *Pf* and human constitutive 20S proteasomes (Fig 4D). First, the macrolactams have radically different conformations evidenced by completely different spatial orientations of the warhead carbonyls (Figure 4D). As the carbonyls appear to differ by more than 90 degrees from one another, most atoms for the rest of the macrolactams cannot be superimposed (Figure 4D). Second, we found that the orientations of the carbonyl of terminal valine methyl ester are different in the two structures: in h20S, the carbonyl is oriented away from the ureido nitrogens; whilst in *Pf* 20S, the carbonyl is oriented towards them (Figure 4E and 4F). Collectively, it is unclear whether those differences in adduct conformations are the cause or consequence of the species-selectivity of inhibition by **C-5**.

Further comparisons of the structures revealed that **C-5**’s ureido moiety and the terminal valine methyl ester has two fewer hydrogen bonds with h20S than with *Pf* 20S (Fig 4E-F). Specifically, h20S Asp125 is shifted away compared to the analogous residue Asp153 of *Pf* 20S, causing one ureido N-H to be outside of hydrogen bond distance (> 3.5 Å) in h20S. In addition, the ureido carbonyl can hydrogen-bond with the Ser27 sidechain of *Pf* 20S, but a similar interaction is not possible in h20S as the analogous residue is Ala27. The fewer overall interactions that **C-5** makes with h20S compared to *Pf* 20S likely explains its lower rate of inhibition and thus its significantly higher species selectivity for *Pf* 20S.

## CONCLUSION

The syringolin natural products are promising therapeutic leads, but the low to modest efficiencies in the published synthetic routes have been a significant barrier for drug development.^49, 53–55^ Here, we report a conformationally guided strategy for improving the notoriously problematic macrocyclization step. Adaptation of the strategy for solid-phase synthesis enabled efficient synthesis of syringolin B methyl ester and analogs thereof in 10 steps with a single purification. Importantly, the key building blocks used in this approach are either commercially available or can be accessed in a single step from commercially available precursors. Moreover, the solid-phase route minimizes reliance on costly chromatographic purifications. Together, these attributes suggest our route could enable both practical syntheses of syringolin analogs and large-scale syntheses of drug leads at reasonable costs.

Our new route enabled the syntheses of eighteen syringolin analogs having a D-amino acid in the peptidourea side chain, which we found to preferentially inhibit the *Pf* 20S proteasome. A subset of those compounds exhibited potent anti-*Plasmodium* activity *in vitro* at a 100 nanomolar concentration and high second-order rate constants for proteasome inhibition. Their weak inhibition of the human constitutive 20S proteasome and absence of toxicity to human cells at concentrations as high as 500 micromolar are indicative of a high degree of species-selectivity. Our structures of the most potent and selective inhibitor in complex with the *Plasmodium* and human proteasomes provided insights into the bases of inhibition. The presence of the D-amino in the side chain of the proteasome inhibitor orients the terminal valine of the peptidourea side chain into the S3 pocket, which is a distinct mode of peptidomicry relative to the natural product (Figure 5).^33,37^ Importantly, the structures revealed an alternative mode of proteasome active site occupancy that can be used for future efforts for structure-guided optimization.

The identification of selective inhibitors of the 20S proteasome from *Plasmodium falciparum*, the causative agent of the most lethal form of human malaria, having nanomolar potency against the parasite from a library of only 18 compounds is remarkable. This parasite relies heavily on proteasome-mediated protein degradation to maintain proteostasis.^59^ The capacity of proteasome inhibitors to act synergistically with current antimalarials enhances their potential as components of next-generation combination therapies.^60–62^ Because the *Plasmodium* proteasome exhibits distinct structural and functional characteristics relative to the human complex, it has been possible to design species-selective inhibitors with potential as anti-malarials.^8–18^ Those efforts have led to the identification of both covalent and non-covalent inhibitors that selectively target the *Plasmodium* proteasome, offering a novel mechanism of action that complements current antimalarial regimens and retains efficacy against artemisinin-resistant strains.^16, 60–62^ Our inhibitors are structurally distinct for those previously reported and may have greater safety due to a less reactive warhead than the vinyl sulfones^8, 9^ and epoxyketones.^17, 18^ Our successes here have motivated on-going work to develop syringolin-based inhibitors having both greater potency and efficacy in animal models of malaria.

## Experimental Section

### Synthetic Methods

#### General Information

All reactions were conducted, when required, in oven-dried round-bottom flasks under an argon atmosphere, using anhydrous solvents and magnetic stirring. Anhydrous solvents were obtained from Fisher Scientific or Sigma-Aldrich, while all reagents were sourced from commercial suppliers without further purification, including AK Scientific, Fisher Scientific, Ambeed, Chem-Impex International, ChemScene, SigmaAldrich, Combi-Blocks, and TCI America. Solvents such as ethyl acetate (EtOAc), acetone (Ace), hexanes (Hex), and methylene chloride (DCM), methanol (MeOH), used for column chromatography and reaction work-ups, were purchased from VWR International, Sigma-Aldrich, and Fisher Scientific. Compounds were visualized with UV light (254 nm).

Column chromatography was conducted with a Teledyne NextGen 300+ flash chromatography system. Rotary evaporation was carried out at 30°C, 40°C, and 60°C under appropriate pressure, considering the solvent used. Nuclear magnetic resonance (NMR) spectra for ^1^H and ^13^C NMR were recorded on a Bruker Avance III HD 400 MHz spectrometer at 298 K, referenced to the residual solvent resonance peaks (CHCl_3_: 7.26 ppm for 1H and 77.00 ppm for ^13^C, DMSO: 2.50 ppm for ^1^H and 39.52 ppm for ^13^C). Multiplicities are denoted with the following abbreviations: s = singlet, d = doublet, t = triplet, q = quartet, quin = quintet, m = multiplet, or combinations thereof. NMR spectra were processed by either Mestrelab Mnova or Bruker TopSpin. High-resolution mass spectra (HRMS) were acquired using a Waters Xevo G2-XS time-of-flight mass spectrometer. Compound purity and reaction progress were analyzed using a Waters Acquity UPLC-MS system. All reported yields are the average of experiments performed in triplicate.

N^2^-((benzyloxy)carbonyl)-N^6^-(3,4-dimethylbenzyl)-L-lysine (**7**).

Z-Lys-OH (4.2 g,15 mmol,1 eq) was added in methanol (100 mL), to the suspension add 2,4-dimethoxylbenzaldehyde (3.05g,18.3mmol,1.3eq), followed by acetic acid (945 μL, 16.5mmol, 1.1 eq) and NaCNBH_3_. The solution was heated to 40 °C and was stirred under the same temperature overnight. The mixture was filtered, and the solid was dried. The solid was used without further purification.

N^2^-((benzyloxy)carbonyl)-N^6^-(2-(diethoxyphosphoryl)acetyl)-N^6^-(3,4-dimethylbenzyl)-L-lysine (**8**).

Na_2_CO_3_ (1.06 g, 10 mmol, 1 eq) was dissolved in 50ml water, followed by adding N^2^-((benzyloxy)carbonyl)-N^6^-(2,4-dimethoxybenzyl)-L-lysine (**7**, 4.3g, 10mmol, 1eq) and 50mL of 1,4-dioxanes. Diethyl (2-(1H-imidazol-1-yl)-2-oxoethyl)phosphonate (4.84 g, 20 mmol, 2 eq) was dissolved in 50mL 1,4-dioxane, and the solution was added to the reaction mixture dropwise. The resulting solution was stirred for 15 h at room temperature. Organic impurities were removed by washing with ethyl acetate (2 × 50 mL). The aqueous phase was acidified to pH = 2 with 1N HCl and extracted with diethyl ether (4 × 30 mL). The combined organic extracts were dried and concentrated to afford a colorless foam. This was used in the next step without further purification.

##### General Method A: Preparation of the HWE-substrate

N^2^-((benzyloxy)carbonyl)-N^6^-(2-(diethoxyphosphoryl)acetyl)-N^6^-(2,4-dimethoxybenzyl)-L-lysine (**8**, 1.21g, 2 mmol, 1 eq) was dissolved in DCM, PyAOP (1.25 g, 2.4 mmol,1.2eq) and DIPEA (977 μL, 4.8 mmol, 2.4 eq) were added into the solution, followed by corresponding amino alcohol (2.4 mmol,1.2 eq). The reaction mixture was stirred overnight. The solvent was removed *in vacuo* and 150ml ethyl acetate was added. The organic phase was washed with 1M HCl (3×100 mL), sat. NaHCO_3_ (3×100 mL) and brine (100 mL). The organic phase was dried with Na_2_SO_4_, filtered, and concentrated. The resulting residue was purified by flash column chromatography with DCM and acetone.

##### General Method B: Formation of the Syringolin B Macrolactam

The appropriate HWE substrate (0.5 mmol) was added in 10 mL DCM, followed by Dess-Martin periodinane (254.4 mg, 0.6 mmol, 1.2 eq). The suspension was stirred at room temperature for 90 minutes. The solution was diluted in 100ml ethyl acetate, the resulting solution was washed with a 1:1 solution of 5% sodium thiosulfate and sat. sodium bicarbonate (1:1, 3×50 mL). Dry the organic phase with sodium sulfate. The organic phase was dried with Na_2_SO_4_, filtered, and concentrated. The resulting aldehyde was dissolved in 50 mL THF.

Zn(OTf)_2_ (400 mg,1.1 mmol, 2.2 eq) was added in 50 mL THF, followed by TMEDA (70mg, 0.6 mmol, 1.2 eq) and TEA (202 mg, 2 mmol, 4 eq). The suspension was stirred at room temperature for 15 minutes. The previous aldehyde solution was added to the reaction mixture through a dropping funnel in 2 h. The reaction mixture was stirred at room temperature overnight. The solvent was removed *in vacuo,* and 100 mL of ethyl acetate was added. The organic phase was washed with 1 M HCl (3 × 100 mL) and brine (100 mL). The organic phase was dried with Na_2_SO_4_, filtered, and concentrated. The resulting residue was purified by flash column chromatography with DCM and ethyl acetate.

##### General Method C: Syntheses of the Syringolin B analogs

The appropriate macrolactam (0.1 mmol) was dissolved in 90% TFA, and 10% thioanisole. The mixture was stirred for 4 hours at 50 °C, and the solvent was removed by evaporation, and diethyl ether was added to the resulting mixture. The resulting suspension was centrifuged, and the supernatant was discarded. The resulting solid was then added to a solution of HATU (38 mg, 0.1 mmol, 1 eq.), the corresponding carboxylic sidechain (0.1 mmol, 1 eq.), and DIPEA (35μL, 0.2 mmol, 2 eq.). The solution was stirred overnight at room temperature. The solvent was removed by evaporation, and the resulting solid was washed with ethyl acetate and acetone, affording the pure syringolin analogs.

benzyl ((S)-6-(2-(diethoxyphosphoryl)-N-(3,4-dimethylbenzyl)acetamido)-1-(((S)-1-hydroxy-3-methylbutan-2-yl)amino)-1-oxohexan-2-yl)carbamate (**3a**).

Following general method A afforded **3a** as white foam,1.17 g yield: 89%

^1^H NMR (400 MHz, CDCl_3_) δ 7.46 – 7.31 (m, 5H), 6.95 (d, *J* = 8.1 Hz, 1H), 6.52 – 6.40 (m, 2H), 5.83 (d, *J* = 8.0 Hz, 1H), 5.10 (s, 2H), 4.69 – 4.51 (m, 1H), 4.34 – 4.23 (m, 3H), 4.21 (s, 1H), 4.21 – 4.10 (m, 3H), 3.84 – 3.77 (m, 6H), 3.71 (d, *J* = 4.9 Hz, 2H), 3.33 (dd, *J* = 21.0, 14.6 Hz, 1H), 3.05 – 2.92 (m, 2H), 1.93 (tq, *J* = 13.8, 6.2 Hz, 2H), 1.69 (d, *J* = 7.3 Hz, 1H), 1.62 – 1.37 (m, 6H), 0.96 (dd, *J* = 12.3, 6.7 Hz, 6H).

^13^C NMR (100 MHz, CDCl_3_) δ 172.1, 165.8, 160.8, 158.3, 136.5, 128.6, 128.4, 128.0, 127.9, 116.1, 103.9, 98.7, 66.6, 62.9, 62.8, 62.7, 57.1, 55.4, 55.2, 46.5, 42.7, 33.1, 29.0, 26.0, 22.4, 19.5, 19.4, 16.4, 16.3.

Benzyl ((S)-6-(2-(diethoxyphosphoryl)-N-(3,4-dimethylbenzyl)acetamido)-1-(((S)-1-hydroxy-4-methylpentan-2-yl)amino)-1-oxohexan-2-yl)carbamate(**3b**).

Following general method A afforded **3b** as white foam. 1.17 g yield: 87%

^1^H NMR (400 MHz, CDCl_3_) δ 7.36 (5H, s), 7.31 (1H, dt, J = 5.0, 3.0 Hz), 6.95 (1H, d, J = 8.0 Hz), 6.50 – 6.40 (2H, m), 5.81 (1H, d, J = 7.9 Hz), 5.11 (2H, s), 4.70 – 4.52 (1H, m), 4.32 (1H, s), 4.29 (1H, d, J = 7.4 Hz), 4.18 (6H, dqd, J = 8.4, 7.1, 1.4 Hz), 3.88 (1H, d, J = 13.1 Hz), 3.83 – 3.80 (6H, m), 3.76 (1H, d, J = 14.3 Hz), 3.54 (1H, ddd, J = 11.7, 7.2, 4.6 Hz), 3.34 (1H, dd, J = 21.1, 14.5 Hz), 3.06 – 2.92 (2H, m), 1.70 – 1.58 (3H, m), 1.51 (4H, ddd, J = 14.8, 11.3, 6.9 Hz), 1.40 (6H, dd, J = 14.1, 6.9 Hz), 0.93 (6H, d, J = 6.5 Hz)

^13^C NMR (100 MHz, CDCl_3_) δ 171.8, 165.7, 160.8, 158.3, 136.5, 128.6, 128.4, 128.0, 127.9, 116.1, 103.9, 98.7, 66.6, 64.9, 62.9, 55.4, 55.2, 49.7, 46.5, 42.6, 40.0, 26.0, 24.8, 22.9, 22.4, 22.4, 16.4.

Benzyl ((S)-6-(2-(diethoxyphosphoryl)-N-(3,4-dimethylbenzyl)acetamido)-1-(((S)-1-hydroxyhexan-2-yl)amino)-1-oxohexan-2-yl)carbamate (**3c**).

Following general method A afforded **3c** as white foam, 1.21 g yield: 90%

^1^H NMR (400 MHz, CDCl_3_) δ 7.41 – 7.32 (m, 5H), 7.24 (s, 2H), 6.95 (d, J = 8.0 Hz, 1H), 6.50 – 6.40 (m, 2H), 5.82 (d, J = 7.8 Hz, 1H), 5.10 (s, 2H), 4.69 – 4.52 (m, 1H), 4.34 – 4.27 (m, 2H), 4.27 – 4.19 (m, 2H), 4.19 – 4.11 (m, 3H), 3.98 – 3.84 (m, 3H), 3.84 – 3.69 (m, 8H), 3.58 (ddd, J = 11.7, 6.9, 4.7 Hz, 1H), 3.33 (dd, J = 21.1, 14.5 Hz, 1H), 2.99 (td, J = 14.5, 9.3 Hz, 2H), 2.07 (s, 1H), 1.94 (s, 1H), 1.64 – 1.51 (m, 4H), 1.39 (dd, J = 14.6, 7.7 Hz, 7H), 0.90 (dq, J = 6.0, 3.5 Hz, 3H).

^13^C NMR (CDCl_3_, 100 MHz) δ 171.8, 165.8, 165.7, 160.8, 158.3, 136.5, 128.6, 128.4, 128.0, 127.9, 116.1, 103.9, 98.7, 66.6, 64.4, 62.9, 62.8, 55.4, 55.2, 51.6, 46.5, 42.7, 33.3, 30.7, 28.3, 26.0, 22.7, 22.4, 16.4, 16.3, 14.0.

Benzyl ((S)-6-(2-(diethoxyphosphoryl)-N-(3,4-dimethylbenzyl)acetamido)-1-(((S)-1-hydroxy-3-phenylpropan-2-yl)amino)-1-oxohexan-2-yl)carbamate (**3d**).

Following general method A afforded **3d** as white foam.1.2 g yield: 85%

^1^H NMR (400 MHz, CDCl_3_) δ 7.50 (d, J = 8.5 Hz, 1H), 7.40 – 7.22 (m, 11H), 7.22 – 7.14 (m, 1H), 6.95 (d, J = 8.0 Hz, 1H), 6.51 – 6.41 (m, 2H), 5.77 (d, J = 7.8 Hz, 1H), 5.11 (s, 2H), 4.66 (d, J = 16.8 Hz, 1H), 4.42 (s, 1H), 4.33 – 4.21 (m, 3H), 3.85 – 3.78 (m, 7H), 3.74 – 3.61 (m, 1H), 3.57 – 3.49 (m, 1H), 3.35 (dd, J = 21.0, 14.5 Hz, 1H), 3.07 – 2.86 (m, 5H), 2.07 (s, 2H), 1.86 (s, 2H), 1.69 (s, 1H), 1.60 – 1.50 (m, 1H), 1.48 (s, 1H), 1.45 – 1.31 (m, 8H).

^13^C NMR (100 MHz, CDCl_3_) δ 171.6, 165.7, 160.8, 158.3, 138.6, 136.6, 129.4, 128.6, 128.4, 128.4, 128.3, 128.0, 127.9, 126.1, 116.1, 103.9, 98.8, 66.6, 63.0, 62.8, 60.4, 55.4, 55.2, 52.9, 46.5, 42.6, 37.0, 22.4, 21.0, 16.4, 14.2.

Benzyl ((S)-6-(2-(diethoxyphosphoryl)-N-(3,4-dimethylbenzyl)acetamido)-1-(((S)-1-(3-fluorophenyl)-3-hydroxypropan-2-yl)amino)-1-oxohexan-2-yl)carbamate (**3e**).

Following general method A afforded **3e** as white foam. 1.25 g yield: 86%

^1^H NMR (400 MHz, CDCl_3_) δ 7.59 (d, J = 8.5 Hz, 1H), 7.40 – 7.28 (m, 7H), 7.28 – 7.15 (m, 1H), 7.15 – 7.05 (m, 1H), 7.05 – 6.92 (m, 2H), 6.92 – 6.84 (m, 1H), 6.51 – 6.41 (m, 2H), 5.75 (d, J = 7.9 Hz, 1H), 5.11 (d, J = 2.0 Hz, 2H), 4.72 – 4.56 (m, 1H), 4.31 (s, 1H), 4.29 – 4.20 (m, 2H), 4.20 – 4.12 (m, 3H), 4.03 – 3.93 (m, 1H), 3.85 – 3.78 (m, 6H), 3.76 – 3.61 (m, 1H), 3.53 (d, J = 11.4 Hz, 1H), 3.37 (dd, J = 21.0, 14.6 Hz, 1H), 3.10 – 2.84 (m, 4H), 2.20 (s, 1H), 1.94 (d, J = 11.5 Hz, 1H), 1.51 – 1.30 (m, 9H), 1.28 (s, 1H).

^13^C NMR (100 MHz, CDCl_3_) δ 171.6, 165.8, 165.7, 160.8, 158.37, 141.3, 141.2, 136.6, 129.7, 129.6, 128.6, 128.4, 128.0, 127.9, 125.0, 116.4, 116.2, 116.0, 113.1, 112.96, 104.0, 98.8, 77.2, 66.6, 63.0, 62.8, 55.4, 55.2, 55.1, 52.7, 46.4, 42.4, 36.7, 33.3, 29.2, 25.8, 22.42, 16.4, 16.3.

Benzyl ((S)-6-(2-(diethoxyphosphoryl)-N-(3,4-dimethylbenzyl)acetamido)-1-(((S)-1-(3,5-difluorophenyl)-3-hydroxypropan-2-yl)amino)-1-oxohexan-2-yl)carbamate(**3f**).

Following general method A afforded **3f** as white foam. 1.31 g yield: 88%

^1^H NMR (400 MHz, CDCl_3_) δ 7.69 (d, J = 8.5 Hz, 1H), 7.39 – 7.27 (m, 5H), 6.94 (d, J = 8.1 Hz, 1H), 6.89 – 6.76 (m, 2H), 6.63 (tt, J = 9.1, 2.3 Hz, 1H), 6.50 – 6.40 (m, 2H), 5.79 (d, J = 8.0 Hz, 1H), 5.10 (s, 2H), 4.65 (d, J = 16.8 Hz, 1H), 4.61 (s, 1H), 4.30 (s, 1H), 4.28 – 4.11 (m, 6H), 3.93 (d, J = 13.5 Hz, 1H), 3.84 – 3.77 (m, 6H), 3.71 (d, J = 12.0 Hz, 1H), 3.57 – 3.49 (m, 1H), 3.37 (dd, J = 20.9, 14.6 Hz, 1H), 3.06 – 2.82 (m, 4H), 2.19 (d, J = 4.9 Hz, 2H), 2.00 – 1.84 (m, 1H), 1.69 (s, 1H), 1.60 – 1.45 (m, 2H), 1.39 (dt, J = 23.3, 7.1 Hz, 8H).

^13^C NMR (100 MHz, CDCl_3_) δ 171.8, 165.8, 165.7, 164.1, 164.0, 161.7, 161.5, 160.8, 158.3, 156.1, 142.8, 142.7, 136.5, 128.6, 128.5, 128.4, 128.0, 127.9, 116.0, 112.36, 112.3, 112.1, 112.1, 103.9, 101.9, 101.6, 101.4, 98.8, 66.6, 63.0, 63.0, 62.8, 62.7, 55.4, 55.3, 55.2, 55.1, 52.5, 46.4, 42.5, 36.7, 34.3, 33.1, 25.8, 22.4, 16.4, 16.3, 16.2.

Benzyl ((5S,8S,E)-1-(2,4-dimethoxybenzyl)-5-isopropyl-2,7-dioxo-1,6-diazacyclododec-3-en-8-yl)carbamate (**4a**).

Following general method B afforded **4a** as a white solid. 247.0 mg yield: 92%

^1^H NMR (400 MHz, CDCl_3_) δ 7.39 – 7.25 (m, 5H), 7.19 – 7.11 (m, 1H), 7.05 – 6.96 (dd, J = 15.3, 4.8 Hz, 1H), 6.92 – 6.85 (d, J = 8.4 Hz, 1H), 6.46 – 6.35 (m, 3H), 6.06 – 5.99 (d, J = 7.4 Hz, 1H), 5.10 – 5.05 (s, 2H), 4.84 – 4.76 (d, J = 15.0 Hz, 1H), 4.68 – 4.60 (dt, J = 7.5, 3.6 Hz, 1H), 4.46 – 4.36 (q, J = 7.3 Hz, 1H), 4.34 – 4.26 (d, J = 14.9 Hz, 1H), 3.85 – 3.76 (d, J = 1.8 Hz, 6H), 3.61 – 3.50 (m, 1H), 3.17 – 3.06 (dt, J = 15.6, 3.9 Hz, 1H), 2.01 – 1.91 (dt, J = 11.5, 5.1 Hz, 2H), 1.84 – 1.75 (dd, J = 13.7, 6.9 Hz, 2H), 1.48 – 1.35 (m, 1H), 1.31 – 1.19 (dt, J = 17.1, 5.2 Hz, 2H), 1.03 – 0.95 (t, J = 6.7 Hz, 6H).

^13^C NMR (100 MHz, CDCl_3_) δ 171.0, 165.7, 160.1, 158.3, 155.7, 144.4, 136.2, 130.1, 128.5, 128.1, 127.7, 120.4, 118.0, 104.3, 98.3, 66.7, 56.7, 55.3, 55.3, 53.7, 44.5, 43.2, 31.6, 31.5, 27.0, 19.6, 19.3, 17.9.

Benzyl ((5S,8S,E)-1-(3,4-dimethylbenzyl)-5-isobutyl-2,7-dioxo-1,6-diazacyclododec-3-en-8-yl)carbamate (**4b**).

Following general method B afford **4b** as a white solid. 225.9 mg yield: 82%

^1^H NMR (400 MHz, CDCl_3_) δ 7.39 – 7.25 (m, 5H), 7.21 – 7.12 (dd, J = 9.7, 7.1 Hz, 1H), 7.08 – 6.96 (dd, J = 15.2, 4.8 Hz, 1H), 6.88 – 6.82 (d, J = 8.1 Hz, 1H), 6.50 – 6.39 (m, 3H), 6.03 – 5.97 (d, J = 7.4 Hz, 1H), 5.11 – 5.09 (s, 1H), 5.09 – 5.02 (d, J = 12.3 Hz, 1H), 4.85 – 4.77 (d, J = 15.0 Hz, 1H), 4.72 – 4.57 (m, 2H), 4.34 – 4.26 (d, J = 15.0 Hz, 1H), 3.81 – 3.76 (s, 6H), 3.60 – 3.49 (m, 1H), 3.16 – 3.06 (dt, J = 15.6, 3.9 Hz, 1H), 2.01 – 1.93 (dd, J = 8.7, 4.5 Hz, 2H), 1.77 – 1.66 (dt, J = 13.5, 6.7 Hz, 2H), 1.54 – 1.34 (m, 3H), 1.32 – 1.20 (dt, J = 12.8, 8.8 Hz, 2H), 0.96 – 0.87 (dd, J = 11.0, 6.6 Hz, 6H).

^13^C NMR (100 MHz, CDCl_3_) δ 170.8, 165.8, 160.1, 158.3, 155.7, 145.6, 136.1, 130.1, 128.5, 128.1, 127.8, 119.6, 118.0, 104.3, 98.3, 66.8, 55.3, 55.3, 53.7, 49.1, 44.57, 43.16, 42.7, 31.5, 27.0, 25.2, 22.7, 22.1, 17.9.

Benzyl ((5S,8S,E)-5-butyl-1-(2,4-dimethoxybenzyl)-2,7-dioxo-1,6-diazacyclododec-3-en-8-yl)carbamate (**4c**).

Following general method B afforded **4c** as a white solid. 231.4 mg yield: 84%

^1^H NMR (400 MHz, CDCl_3_) δ 7.39 – 7.25 (m, 5H), 7.20 – 7.11 (m, 1H), 7.06 – 6.96 (dd, J = 15.2, 4.8 Hz, 1H), 6.80 – 6.74 (d, J = 8.0 Hz, 1H), 6.47 – 6.38 (m, 3H), 6.02 – 5.95 (d, J = 7.4 Hz, 1H), 5.18 – 5.03 (d, J = 2.7 Hz, 2H), 4.85 – 4.77 (d, J = 14.9 Hz, 1H), 4.66 – 4.53 (m, 2H), 4.35 – 4.26 (d, J = 14.9 Hz, 1H), 3.85 – 3.76 (d, J = 1.0 Hz, 6H), 3.61 – 3.50 (m, 1H), 3.17 – 3.07 (dt, J = 15.5, 3.9 Hz, 1H), 2.02 – 1.90 (td, J = 11.9, 7.8 Hz, 2H), 1.72 – 1.59 (ddt, J = 13.0, 9.1, 6.1 Hz, 1H), 1.58 – 1.46 (dtd, J = 13.8, 8.8, 5.3 Hz, 1H), 1.45 – 1.20 (m, 7H), 0.93 – 0.85 (t, J = 7.1 Hz, 3H).

^13^C NMR (100 MHz, CDCl_3_) δ 170.8, 165.8, 160.1, 158.3, 155.6, 145.4, 136.1, 130.2, 128.5, 128.1, 127.7, 119.6, 118.1, 104.3, 98.3, 66.8, 55.3, 55.3, 53.7, 50.8, 44.5, 43.1, 33.3, 31.5, 28.4, 27.0, 22.3, 17.9, 13.8.

Benzyl ((5S,8S,E)-5-benzyl-1-(3,4-dimethylbenzyl)-2,7-dioxo-1,6-diazacyclododec-3-en-8-yl)carbamate (**4d**).

Following general method B afforded **4d** as a white solid. 243.0 mg yield: 83%

^1^H NMR (400MHz, CDCl_3_): δ8.53–8.35(1H,d), 7.40–7.28(8H,m), 7.30–7.20(1H,d), 7.12–7.02(1H,d), 7.00–6.86(1H,d), 6.84–6.72(1H,d),6.59–6.53(1H,d), 6.54–6.43(2H,m), 5.03–4.92(2H,s), 4.70–4.56(2H,d), 4.39–4.25(1H,s), 4.22–4.11(1H,d), 3.81–3.76(3H,s), 3.76–3.72(3H,s), 3.71–3.58(1H,m), 3.30–3.27(2H,s), 3.11–2.88(2H,m), 2.81–2.70(1H,m), 2.25–2.08(1H,m), 1.69–1.55(2H,s), 1.49–1.32(1H,m), 1.27–1.28(1H,m). 1.06–0.91(1H,m)

^13^C NMR (100 MHz, CDCl_3_) δ 171.6, 165.8, 160.8, 158.3, 138.6, 136.6, 129.4, 128.6, 128.4, 128.3, 128.0, 127.9, 126.1, 116.1, 103.9, 98.8, 66.6, 63.0, 62.9, 62.8, 55.46, 55.27, 52.9, 46.5, 42.6, 37.0, 33.1, 25.9, 22.4, 16.4.

Benzyl ((5S,8S,E)-1-(3,4-dimethylbenzyl)-5-(3-fluorobenzyl)-2,7-dioxo-1,6-diazacyclododec-3-en-8-yl)carbamate (**4e**).

Following general method B afforded **4e** as a white solid. 235.4 mg yield: 78%

^1^H NMR (400 MHz, CDCl_3_) δ 7.32 (s, 5H), 7.21 (td, J = 7.9, 6.0 Hz, 1H), 7.16 – 7.10 (m, 1H), 7.04 – 6.83 (m, 5H), 6.51 – 6.38 (m, 3H), 5.93 (d, J = 7.2 Hz, 1H), 5.08 (s, 2H), 4.92 (s, 1H), 4.79 (d, J = 14.9 Hz, 1H), 4.50 (dt, J = 7.8, 3.5 Hz, 1H), 4.32 (d, J = 15.0 Hz, 1H), 3.77 (d, J = 3.3 Hz, 6H), 3.61 – 3.50 (m, 1H), 3.12 (dd, J = 15.6, 4.8 Hz, 1H), 2.94 (dd, J = 14.3, 6.0 Hz, 1H), 2.71 (dd, J = 14.3, 8.8 Hz, 1H), 1.94 (d, J = 10.5 Hz, 2H), 1.40 (t, J = 9.5 Hz, 1H), 1.32 – 1.16 (m, 2H).

^13^C NMR (100 MHz, CDCl_3_) δ 170.9, 165.6, 164.0, 161.6, 160.2, 158.3, 155.5, 144.5, 136.2, 130.2, 130.1, 129.9, 128.5, 128.1, 127.8, 124.4, 120.4, 117.8, 115.9, 115.7, 114.0, 113.8, 104.3, 98.3, 66.8, 55.3, 55.3, 53.6, 51.6, 44.6, 43.4, 38.7, 31.2, 27.0, 17.8.

Benzyl ((5S,8S,E)-5-(3,5-difluorobenzyl)-1-(3,4-dimethylbenzyl)-2,7-dioxo-1,6-diazacyclododec-3-en-8-yl)carbamate (**4f**).

Following general method B afforded **4f** as a white solid. 245.6 mg yield: 79%

^1^H NMR (400 MHz, CDCl_3_) δ 7.32 (s, 6H), 7.15 (td, J = 9.0, 5.9 Hz, 2H), 6.99 (dd, J = 15.2, 4.6 Hz, 1H), 6.85 – 6.73 (m, 3H), 6.53 – 6.45 (m, 1H), 6.45 – 6.39 (m, 2H), 5.90 (d, J = 7.2 Hz, 1H), 5.08 (s, 2H), 4.79 (d, J = 14.9 Hz, 1H), 4.55 – 4.48 (m, 1H), 4.33 (d, J = 14.9 Hz, 1H), 3.78 (s, 6H), 3.56 (t, J = 13.8 Hz, 1H), 3.14 (dd, J = 15.7, 4.6 Hz, 1H), 2.93 (dd, J = 14.5, 5.6 Hz, 1H), 2.76 (dd, J = 14.5, 9.5 Hz, 1H), 2.00 – 1.89 (m, 2H), 1.41 (s, 1H), 1.25 (d, J = 11.3 Hz, 2H).

^13^C NMR (100 MHz, CDCl_3_) δ 170.9, 165.5, 160.1, 158.3, 155.5, 144.0, 136.1, 130.1, 128.5, 128.1, 127.82 120.6, 117.9, 104.3, 103.9, 98.3, 66.8, 60.4, 55.3, 55.3, 53.72, 51.49, 44.5, 43.3, 31.7, 31.3, 26.9, 21.0, 17.8, 14.2.

Methyl (((R)-1-(((5S,8S,E)-5-isopropyl-2,7-dioxo-1,6-diazacyclododec-3-en-8-yl)amino)-3-methyl-1-oxobutan-2-yl)carbamoyl)-L-valinate (**A-1**).

Following general method C, affording A-1 as white solid. 33mg yield:65%

^1^H NMR (600 MHz, MeOD) δ 7.07 (dd, J = 15.5, 5.0 Hz, 1H), 6.60 (dd, J = 14.8, 8.3 Hz, 1H), 6.38 (d, J = 15.5 Hz, 1H), 4.75 – 4.54 (m, 6H), 4.23 (dq, J = 18.6, 5.8 Hz, 2H), 4.09 (t, J = 5.7 Hz, 1H), 3.69 (s, 3H), 3.56 – 3.45 (m, 1H), 3.13 (dt, J = 15.3, 4.1 Hz, 1H), 2.21 (dq, J = 13.4, 6.8 Hz, 1H), 2.15 (dt, J = 13.0, 6.7 Hz, 1H), 2.12 – 2.05 (m, 1H), 1.88 (ddq, J = 33.6, 13.8, 5.9 Hz, 2H), 1.70 – 1.61 (m, 1H), 1.40 (dt, J = 18.4, 9.2 Hz, 2H), 1.07 (dd, J = 18.4, 6.7 Hz, 7H), 0.99 (dd, J = 11.3, 6.7 Hz, 6H), 0.93 (q, J = 5.9 Hz, 6H).

^13^C NMR (151 MHz, MeOD) δ 173.1, 172.4, 171.5, 168.2, 146.7, 118.9, 59.3, 58.2, 56.9, 51.8, 51.0, 38.4, 31.9, 30.6, 30.2, 29.8, 19.1, 18.4, 18.3, 18.1, 17.0, 16.5, 16.17.

HRMS (ESI) m/z: [M + H]+ Calcd for (C_25_H_44_N_5_O_6_)+: 510.3286; Found 510.3287.

Methyl (((R)-1-(((5S,8S,E)-5-isobutyl-2,7-dioxo-1,6-diazacyclododec-3-en-8-yl)amino)-3-methyl-1-oxobutan-2-yl)carbamoyl)-L-valinate (**A-2**).

Following general method C afforded **A-2** as white solid. 39 mg yield: 75%

^1^H NMR (600 MHz, MeOD) δ 7.03 (dd, J = 15.4, 4.9 Hz, 1H), 6.60 (dd, J = 13.9, 8.4 Hz, 1H), 6.39 (d, J = 15.4 Hz, 1H), 4.70 (d, J = 3.9 Hz, 1H), 4.64 (s, 2H), 4.58 (dt, J = 9.8, 5.6 Hz, 1H), 4.22 (t, J = 5.8 Hz, 1H), 4.09 (d, J = 5.3 Hz, 1H), 3.69 (s, 3H), 3.55 – 3.47 (m, 1H), 3.33 (s, 2H), 3.12 (dt, J = 15.7, 3.9 Hz, 1H), 2.21 (h, J = 6.7 Hz, 1H), 2.17 – 2.06 (m, 2H), 1.91 (ddt, J = 14.5, 11.1, 5.3 Hz, 1H), 1.79 (dp, J = 13.4, 6.8 Hz, 1H), 1.50 (dt, J = 8.1, 4.4 Hz, 2H), 1.46 – 1.35 (m, 2H), 1.31 (s, 1H), 1.10 (q, J = 12.5 Hz, 1H), 1.02 – 0.91 (m, 18H).

^13^C NMR (151 MHz, MeOD) δ 173.1, 172.4, 171.5, 168.2, 159.0, 147.5, 118.2, 59.3, 58.2, 51.8, 51.0, 48.9, 41.7, 38.3, 30.6, 30.2, 29.8, 29.8, 25.0, 21.8, 20.6, 18.4, 18.1, 17.1, 16.6, 16.1.

HRMS (ESI) m/z: [M + H]+ Calcd for (C_26_H_46_N_5_O_6_)+: 524.3443; Found 524.3458.

Methyl (((R)-1-(((5S,8S,E)-5-butyl-2,7-dioxo-1,6-diazacyclododec-3-en-8-yl)amino)-3-methyl-1-oxobutan-2-yl)carbamoyl)-L-valinate (**A-3**).

Following general method C afforded **A-3** as white solid. 35mg yield: 73%

^1^H NMR (600 MHz, MeOD) δ 7.03 (ddd, J = 13.7, 8.6, 4.7 Hz, 1H), 6.60 (dd, J = 14.1, 8.4 Hz, 1H), 6.39 (d, J = 15.4 Hz, 1H), 4.72 – 4.65 (m, 2H), 4.64 (s, 2H), 4.47 (dt, J = 10.3, 5.4 Hz, 1H), 4.22 (dd, J = 14.0, 5.4 Hz, 1H), 4.09 (t, J = 5.7 Hz, 1H), 3.71 (d, J = 19.3 Hz, 3H), 3.51 (t, J = 13.7 Hz, 1H), 3.37 (s, 3H), 3.13 (dd, J = 15.5, 4.4 Hz, 1H), 2.21 (h, J = 6.7 Hz, 1H), 2.17 – 2.05 (m, 2H), 1.95 – 1.86 (m, 1H), 1.70 (dt, J = 14.9, 5.2 Hz, 1H), 1.65 (s, 1H), 1.58 (td, J = 9.0, 4.3 Hz, 1H), 1.51 (t, J = 8.4 Hz, 1H), 1.45 – 1.35 (m, 5H), 1.10 (q, J = 12.4 Hz, 1H), 0.96 (ddt, J = 25.9, 12.9, 6.6 Hz, 15H).

^13^C NMR (151 MHz, MeOD) δ 173.1, 171.5, 168.2, 159.0, 147.4, 118.2, 58.2, 51.0, 50.7, 48.4, 48.0, 47.8, 47.7, 47.6, 47.4, 47.3, 47.1, 38.3, 32.5, 30.6, 30.2, 29.8, 28.54, 22.04, 18.4, 18.1, 17.0, 16.5, 16.1, 12.8.

HRMS (ESI) m/z: [M + H]+ Calcd for (C_26_H_46_N_5_O_6_)+: 524.3443; Found 524.3458.

Methyl (((R)-1-(((5S,8S,E)-5-benzyl-2,7-dioxo-1,6-diazacyclododec-3-en-8-yl)amino)-3-methyl-1-oxobutan-2-yl)carbamoyl)-L-valinate (**A-4**).

Following general method C afforded **A-4** as white solid. 40 mg yield: 71%

1H NMR (400 MHz, DMSO) δ 8.48 (d, J = 8.2 Hz, 1H), 7.88 (d, J = 8.1 Hz, 1H), 7.44 – 7.27 (m, 5H), 7.22 (ddt, J = 8.5, 6.3, 2.8 Hz, 1H), 6.77 (dd, J = 15.3, 4.7 Hz, 1H), 6.50 (d, J = 9.0 Hz, 1H), 6.33 (d, J = 15.4 Hz, 1H), 6.29 (d, J = 8.8 Hz, 1H), 4.60 (s, 1H), 4.11 (ddd, J = 21.0, 8.9, 5.5 Hz, 2H), 3.59 (s, 3H), 3.01 – 2.87 (m, 2H), 2.72 (dd, J = 13.9, 9.6 Hz, 1H), 2.15 (t, J = 13.3 Hz, 1H), 2.02 – 1.82 (m, 2H), 1.55 (s, 1H), 1.45 (s, 1H), 1.34 – 1.20 (m, 2H), 0.96 (q, J = 12.5 Hz, 1H), 0.87 – 0.79 (m, 9H), 0.75 (d, J = 6.9 Hz, 3H).

^13^C NMR (100 MHz, DMSO) δ 173.5, 171.6, 171.4, 166.1, 157.8, 145.8, 138.8, 129.3, 128.8, 126.8, 119.8, 57.9, 57.8, 52.2, 51.9, 51.4, 38.7, 38.4, 31.7, 31.0, 30.7, 19.5, 19.4, 18.1, 17.9, 17.7.

HRMS (ESI) m/z: [M + H]+ Calcd for (C_29_H_44_N_5_O_6_)+: 558.3286; Found 558.3297.

Methyl (((R)-1-(((5S,8S,E)-5-(3-fluorobenzyl)-2,7-dioxo-1,6-diazacyclododec-3-en-8-yl)amino)-3-methyl-1-oxobutan-2-yl)carbamoyl)-L-valinate (**A-5**).

Following general method C afforded **A-5** as a white solid. 25mg yield: 43%

^1^H NMR (400 MHz, DMSO) δ 8.47 (d, J = 8.2 Hz, 1H), 7.89 (d, J = 8.1 Hz, 1H), 7.45 – 7.29 (m, 2H), 7.20 – 7.12 (m, 2H), 7.05 (td, J = 8.6, 2.5 Hz, 1H), 6.78 (dd, J = 15.3, 4.6 Hz, 1H), 6.50 (d, J = 9.0 Hz, 1H), 6.37 – 6.26 (m, 2H), 4.66 – 4.57 (m, 2H), 4.11 (ddd, J = 22.4, 8.9, 5.5 Hz, 2H), 3.59 (s, 3H), 3.28 (d, J = 13.1 Hz, 1H), 2.96 (dd, J = 14.0, 6.1 Hz, 2H), 2.71 (dd, J = 14.0, 10.0 Hz, 1H), 2.20 – 2.07 (m, 1H), 1.92 (dh, J = 26.3, 6.7 Hz, 2H), 1.55 (s, 1H), 1.46 (s, 1H), 1.35 – 1.21 (m, 2H), 0.96 (q, J = 12.5 Hz, 1H), 0.88 – 0.78 (m, 9H), 0.75 (d, J = 6.8 Hz, 3H).

^13^C NMR (100 MHz, DMSO) δ 173.5, 171.6, 171.4, 166.1, 163.7, 161.3, 157.8, 145.5, 141.8, 141.7, 130.6, 130.6, 125.5, 119.9, 116.2, 116.0, 113.8, 113.5, 57.9, 57.83, 52.05, 51.9, 51.4, 38.4, 38.3, 31.7, 31.0, 30.7, 19.5, 19.4, 18.1, 17.9, 17.7.

HRMS (ESI) m/z: [M + H]+ Calcd for (C_29_H_43_FN_5_O_6_)+: 576.3192; Found 576.3230.

Methyl (((R)-1-(((5S,8S,E)-5-(3,5-difluorobenzyl)-2,7-dioxo-1,6-diazacyclododec-3-en-8-yl)amino)-3-methyl-1-oxobutan-2-yl)carbamoyl)-L-valinate (**A-6**).

Following general method C afforded **A-6** as a white solid. 29 mg yield: 49%

^1^H NMR (400 MHz, DMSO) δ 8.49 (d, J = 8.3 Hz, 1H), 7.91 (d, J = 8.1 Hz, 1H), 7.49 – 7.40 (m, 2H), 7.26 – 7.16 (m, 1H), 7.07 (td, J = 8.5, 2.6 Hz, 1H), 6.71 (dd, J = 15.3, 4.7 Hz, 1H), 6.51 (d, J = 8.9 Hz, 1H), 6.35 (d, J = 15.3 Hz, 1H), 6.30 (d, J = 8.8 Hz, 1H), 4.62 (d, J = 14.0 Hz, 2H), 4.11 (ddd, J = 23.8, 8.9, 5.5 Hz, 2H), 3.60 (s, 3H), 2.94 (td, J = 14.9, 8.9 Hz, 2H), 2.82 – 2.72 (m, 1H), 2.17 (t, J = 13.2 Hz, 1H), 1.92 (ddt, J = 32.1, 13.3, 6.6 Hz, 2H), 1.54 (s, 1H), 1.45 (s, 1H), 1.35 – 1.24 (m, 2H), 0.97 (t, J = 12.9 Hz, 1H), 0.87 – 0.79 (m, 9H), 0.76 (d, J = 6.8 Hz, 3H).

^13^C NMR (100 MHz, DMSO) δ 173.5, 171.6, 171.5, 157.8, 144.8, 104.2, 57.9, 57.80, 51.9, 50.7, 31.7, 31.0, 30.6, 19.5, 19.4, 18.1, 17.9.

HRMS (ESI) m/z: [M + H]+ Calcd for (C_29_H_42_F_2_N_5_O_6_)+: 594.3098; Found 594.3090.

*tert*-butyl (R)-5-(((5S,8S,E)-5-isopropyl-2,7-dioxo-1,6-diazacyclododec-3-en-8-yl)amino)-4-(3-((S)-1-methoxy-3-methyl-1-oxobutan-2-yl)ureido)-5-oxopentanoate (**B-1**).

Following general method C afforded **B-1** as a white solid. 36.8 mg yield: 62%

^1^H NMR (400 MHz, DMSO) δ 8.29 (d, J = 8.2 Hz, 1H), 7.91 (d, J = 7.9 Hz, 1H), 7.37 (t, J = 7.1 Hz, 1H), 6.79 (dd, J = 15.3, 4.9 Hz, 1H), 6.49 (d, J = 8.8 Hz, 1H), 6.40 (d, J = 8.1 Hz, 1H), 6.22 (d, J = 15.3 Hz, 1H), 4.66 (dt, J = 7.7, 3.6 Hz, 1H), 4.26 (td, J = 7.9, 5.5 Hz, 1H), 4.18 – 4.02 (m, 2H), 3.61 (s, 3H), 3.32 – 3.21 (m, 1H), 2.95 (d, J = 14.4 Hz, 1H), 2.15 (q, J = 8.2 Hz, 2H), 2.06 (d, J = 13.6 Hz, 1H), 2.03 – 1.91 (m, 1H), 1.82 – 1.73 (m, 2H), 1.71 – 1.52 (m, 2H), 1.38 (s, 9H), 1.29 (s, 1H), 1.24 (d, J = 14.7 Hz, 1H), 0.95 (dd, J = 11.4, 6.6 Hz, 6H), 0.84 (dd, J = 11.2, 6.8 Hz, 6H).

^13^C NMR (100 MHz, DMSO) δ 173.4, 172.1, 171.6, 171.2, 166.2, 157.6, 145.3, 120.1, 80.0, 58.1, 56.5, 52.3, 51.9, 51.6, 38.4, 31.6, 31.6, 30.9, 30.7, 30.6, 29.4, 28.2, 20.3, 19.7, 19.46, 18.2, 17.6.

HRMS (ESI) m/z: [M + H]+ Calcd for (C_29_H_50_N_5_O_8_)+: 596.3654; Found 596.3669.

*tert*-butyl (R)-5-(((5S,8S,E)-5-isobutyl-2,7-dioxo-1,6-diazacyclododec-3-en-8-yl)amino)-4-(3-((S)-1-methoxy-3-methyl-1-oxobutan-2-yl)ureido)-5-oxopentanoate (**B-2**).

Following general method C afforded **B-2** as a white solid. 29 mg yield: 47%

^1^H NMR (400 MHz, DMSO) δ 8.36 (d, J = 8.0 Hz, 1H), 7.90 (d, J = 7.8 Hz, 1H), 7.35 (t, J = 7.1 Hz, 1H), 6.78 (dd, J = 15.2, 4.8 Hz, 1H), 6.49 (d, J = 8.8 Hz, 1H), 6.40 (d, J = 8.1 Hz, 1H), 6.25 (d, J = 15.3 Hz, 1H), 4.61 – 4.55 (m, 1H), 4.42 – 4.34 (m, 1H), 4.26 (q, J = 7.4 Hz, 1H), 4.06 (dd, J = 8.7, 5.3 Hz, 1H), 3.61 (s, 3H), 2.95 (d, J = 14.4 Hz, 1H), 2.14 (q, J = 11.1 Hz, 4H), 1.96 (dt, J = 13.3, 6.6 Hz, 1H), 1.82 – 1.74 (m, 1H), 1.73 – 1.64 (m, 2H), 1.64 – 1.56 (m, 1H), 1.41 (s, 2H), 1.38 (s, 10H), 1.28 (d, J = 13.5 Hz, 2H), 1.04 – 0.75 (m, 15H).

^13^C NMR (100 MHz, DMSO) δ 173.4, 172.1, 171.6, 171.1, 166.2, 157.6, 146.5, 119.4, 80.0, 58.1, 52.3, 52.0, 51.6, 48.7, 41.9, 38.4, 31.6, 30.9, 30.7, 30.4, 29.4, 28.20, 25.34, 23.30, 22.0, 19.4, 18.2, 17.6.

HRMS (ESI) m/z: [M + H]+ Calcd for (C_30_H_52_N_5_O_8_)+: 610.3810; Found 610.3825.

*tert*-butyl (R)-5-(((5S,8S,E)-5-butyl-2,7-dioxo-1,6-diazacyclododec-3-en-8-yl)amino)-4-(3-((S)-1-methoxy-3-methyl-1-oxobutan-2-yl)ureido)-5-oxopentanoate (**B-3**).

Following general method C afforded **B-3** as a white solid. 25 mg yield: 41%

^1^H NMR (400 MHz, DMSO) δ 8.36 (d, J = 8.0 Hz, 1H), 7.90 (d, J = 7.9 Hz, 1H), 7.35 (t, J = 7.0 Hz, 1H), 6.79 (dd, J = 15.3, 4.7 Hz, 1H), 6.49 (d, J = 8.8 Hz, 1H), 6.40 (d, J = 8.1 Hz, 1H), 6.24 (d, J = 15.3 Hz, 1H), 4.59 (dd, J = 8.0, 4.1 Hz, 1H), 4.33 – 4.21 (m, 2H), 4.06 (dd, J = 8.8, 5.4 Hz, 1H), 3.61 (s, 3H), 3.26 (s, 1H), 3.00 – 2.91 (m, 1H), 2.16 (t, J = 8.0 Hz, 2H), 2.09 (t, J = 12.6 Hz, 1H), 1.97 (dq, J = 13.2, 6.7 Hz, 1H), 1.79 (dq, J = 13.6, 7.1 Hz, 1H), 1.65 (dt, J = 14.2, 7.6 Hz, 2H), 1.58 (s, 1H), 1.54 (s, 1H), 1.45 (s, 2H), 1.38 (s, 10H), 1.30 (dt, J = 9.8, 6.7 Hz, 6H), 0.98 (t, J = 12.3 Hz, 1H), 0.91 – 0.82 (m, 9H).

^13^C NMR (100 MHz, DMSO) δ 173.4, 172.1, 171.6, 171.2, 166.2, 157.6, 146.3, 119.3, 80.0, 58.1, 52.3, 51.9, 51.6, 50.5, 38.4, 32.7, 31.6, 30.9, 30.7, 30.4, 29.4, 28.8, 28.2, 22.3, 19.46, 18.2, 17.6, 14.3.

HRMS (ESI) m/z: [M + H]+ Calcd for (C_30_H_52_N_5_O_8_)+: 610.3810; Found 610.3825.

*tert*-butyl (R)-5-(((5S,8S,E)-5-benzyl-2,7-dioxo-1,6-diazacyclododec-3-en-8-yl)amino)-4-(3-((S)-1-methoxy-3-methyl-1-oxobutan-2-yl)ureido)-5-oxopentanoate (**B-4**).

Following general method C afforded **B-4** as a white solid. 35 mg yield: 54%

^1^H NMR (400 MHz, DMSO) δ 8.50 (d, J = 8.2 Hz, 1H), 7.89 (d, J = 7.9 Hz, 1H), 7.40 (d, J = 7.0 Hz, 1H), 7.31 (d, J = 5.4 Hz, 4H), 7.22 (ddd, J = 10.5, 5.9, 3.1 Hz, 1H), 6.78 (dd, J = 15.3, 4.7 Hz, 1H), 6.47 (d, J = 8.8 Hz, 1H), 6.39 – 6.29 (m, 2H), 4.61 (s, 1H), 4.59 – 4.52 (m, 1H), 4.28 – 4.18 (m, 1H), 4.04 (dd, J = 8.8, 5.4 Hz, 1H), 3.59 (s, 3H), 3.00 (s, 1H), 2.97 – 2.87 (m, 1H), 2.76 – 2.66 (m, 1H), 2.15 (q, J = 9.4 Hz, 3H), 1.96 (dq, J = 13.4, 6.8 Hz, 1H), 1.77 (h, J = 7.2 Hz, 1H), 1.63 (dt, J = 14.1, 7.7 Hz, 2H), 1.37 (s, 9H), 1.31 (s, 1H), 1.25 (d, J = 7.0 Hz, 3H), 0.97 (t, J = 13.2 Hz, 1H), 0.83 (dd, J = 11.5, 6.8 Hz, 6H).

^13^C NMR (100 MHz, DMSO) δ 173.4, 172.1, 171.6, 171.2, 166.1, 157.6, 145.8, 138.8, 129.3, 128.7, 126.8, 119.8, 80.0, 58.0, 52.3, 51.9, 51.6, 38.7, 38.3, 31.6, 30.91, 30.72, 30.4, 29.3, 28.1, 19.4, 18.2, 17.6.

HRMS (ESI) m/z: [M + H]+ Calcd for (C_33_H_50_N_5_O_8_)+: 644.3654; Found 644.3688.

*tert*-butyl (R)-5-(((5S,8S,E)-5-(3-fluorobenzyl)-2,7-dioxo-1,6-diazacyclododec-3-en-8-yl)amino)-4-(3-((S)-1-methoxy-3-methyl-1-oxobutan-2-yl)ureido)-5-oxopentanoate (**B-5**). Following general method C afforded **B-5** as a white solid. 40 mg yield: 60%

^1^H NMR (400 MHz, DMSO) δ 8.49 (d, J = 8.2 Hz, 1H), 7.90 (d, J = 7.9 Hz, 1H), 7.45 – 7.31 (m, 2H), 7.20 – 7.12 (m, 2H), 7.05 (td, J = 8.9, 2.6 Hz, 1H), 6.78 (dd, J = 15.3, 4.7 Hz, 1H), 6.47 (d, J = 8.8 Hz, 1H), 6.39 – 6.28 (m, 2H), 4.62 (s, 1H), 4.55 (s, 1H), 4.23 (q, J = 7.3 Hz, 1H), 4.04 (dd, J = 8.8, 5.3 Hz, 1H), 3.59 (s, 3H), 3.01 – 2.87 (m, 2H), 2.70 (dd, J = 15.0, 11.3 Hz, 2H), 2.12 (q, J = 8.4 Hz, 3H), 1.95 (dt, J = 13.4, 6.7 Hz, 1H), 1.76 (t, J = 7.0 Hz, 1H), 1.62 (dd, J = 14.2, 7.5 Hz, 2H), 1.46 (s, 2H), 1.37 (s, 9H), 1.26 (q, J = 11.4 Hz, 5H), 0.96 (t, J = 12.4 Hz, 1H), 0.83 (dd, J = 11.6, 6.8 Hz, 6H).

^13^C NMR (100 MHz, DMSO) δ 173.4, 172.1, 171.6, 171.3, 166.1, 157.6, 145.5, 141.7, 130.6, 130.5, 125.5, 119.9, 116.2, 116.0, 113.7, 80.0, 58.1, 52.3, 52.1, 51.9, 51.65, 38.27, 31.6, 30.9, 30.7, 30.4, 29.3, 28.1, 19.4, 18.2, 17.6.

HRMS (ESI) m/z: [M + H]+ Calcd for (C_33_H_49_FN_5_O_8_)+: 662.3560; Found 662.3594.

*tert*-butyl (R)-5-(((5S,8S,E)-5-(3,5-difluorobenzyl)-2,7-dioxo-1,6-diazacyclododec-3-en-8-yl)amino)-4-(3-((S)-1-methoxy-3-methyl-1-oxobutan-2-yl)ureido)-5-oxopentanoate (**B-6**).

Following general method C afforded **B-6** as a white solid. 39 mg yield: 57%

^1^H NMR (400 MHz, DMSO) δ 8.52 (d, J = 8.3 Hz, 1H), 7.92 (d, J = 7.8 Hz, 1H), 7.44 (q, J = 8.5 Hz, 2H), 7.25 – 7.16 (m, 1H), 7.06 (s, 1H), 6.72 (dd, J = 15.2, 4.7 Hz, 1H), 6.48 (d, J = 8.8 Hz, 1H), 6.40 – 6.32 (m, 2H), 4.63 (s, 1H), 4.54 (d, J = 7.6 Hz, 1H), 4.24 (q, J = 7.2 Hz, 1H), 4.05 (dd, J = 8.8, 5.4 Hz, 1H), 3.59 (s, 3H), 3.13 (d, J = 7.4 Hz, 1H), 3.02 – 2.87 (m, 2H), 2.81 – 2.67 (m, 1H), 2.16 (q, J = 11.8 Hz, 3H), 1.96 (h, J = 6.9 Hz, 1H), 1.77 (dq, J = 14.0, 7.2 Hz, 1H), 1.63 (dq, J = 15.9, 8.2 Hz, 2H), 1.48 – 1.43 (m, 1H), 1.37 (s, 9H), 1.31 (s, 1H), 1.26 (d, J = 7.0 Hz, 4H), 0.96 (t, J = 13.3 Hz, 1H), 0.83 (dd, J = 11.5, 6.7 Hz, 6H).

^13^C NMR (100 MHz, DMSO) δ 173.4, 172.1, 171.6, 171.4, 166.1, 157.6, 144.8, 132.7, 120.4, 112.0, 104.4, 104.1, 80.0, 58.0, 52.3, 51.9, 51.7, 50.7, 38.3, 31.6, 31.36, 30.92, 30.7, 30.3, 29.3, 28.1, 19.4, 18.2, 17.6.

HRMS (ESI) m/z: [M + H]+ Calcd for (C_33_H_48_F_2_N_5_O_8_)+: 680.3465; Found 680.3489.

Methyl (((R)-1-(((5S,8S,E)-5-isopropyl-2,7-dioxo-1,6-diazacyclododec-3-en-8-yl)amino)-1-oxo-3-phenylpropan-2-yl)carbamoyl)-L-valinate (**C-1**).

Following general method C afforded **C-1** as a white solid. 25 mg yield: 45%

^1^H NMR (400 MHz, DMSO) δ 8.25 (d, J = 8.3 Hz, 1H), 7.87 (d, J = 8.1 Hz, 1H), 7.36 (t, J = 7.1 Hz, 1H), 7.27 – 7.11 (m, 5H), 6.78 (dd, J = 15.4, 4.8 Hz, 1H), 6.47 (d, J = 8.9 Hz, 1H), 6.37 (d, J = 8.3 Hz, 1H), 6.18 (d, J = 15.3 Hz, 1H), 4.63 (dt, J = 7.7, 3.6 Hz, 1H), 4.55 (q, J = 7.5 Hz, 1H), 4.12 (td, J = 7.9, 4.7 Hz, 1H), 4.03 (dd, J = 8.8, 5.3 Hz, 1H), 3.60 (s, 3H), 3.24 (q, J = 12.0 Hz, 1H), 2.97 – 2.81 (m, 2H), 2.76 (dd, J = 13.1, 7.4 Hz, 1H), 2.03 – 1.86 (m, 2H), 1.76 (h, J = 6.8 Hz, 1H), 1.35 (d, J = 13.6 Hz, 2H), 1.16 (t, J = 13.5 Hz, 1H), 0.94 (dd, J = 10.7, 6.7 Hz, 6H), 0.79 (t, J = 6.5 Hz, 6H).

^13^C NMR (100 MHz, DMSO) δ 173.4, 171.2, 171.2, 166.1, 157.3, 145.3, 138.0, 129.8, 128.3, 126.5, 120.1, 57.9, 56.4, 54.3, 51.9, 51.4, 39.3, 38.4, 31.6, 30.9, 30.7, 20.31, 19.78, 19.4, 18.1, 17.4.

HRMS (ESI) m/z: [M + H]+ Calcd for (C_29_H_44_N_5_O_6_)+: 558.3286; Found 558.3297.

Methyl (((R)-1-(((5S,8S,E)-5-isobutyl-2,7-dioxo-1,6-diazacyclododec-3-en-8-yl)amino)-1-oxo-3-phenylpropan-2-yl)carbamoyl)-L-valinate (**C-2**).

Following general method C afforded **C-2** as a white solid. 28 mg yield: 49%

^1^H NMR (400 MHz, DMSO) δ 8.31 (d, J = 8.0 Hz, 1H), 7.86 (d, J = 8.0 Hz, 1H), 7.34 (t, J = 7.1 Hz, 1H), 7.27 – 7.21 (m, 2H), 7.18 (dd, J = 9.5, 6.5 Hz, 4H), 6.77 (dd, J = 15.2, 4.7 Hz, 1H), 6.47 (d, J = 8.9 Hz, 1H), 6.36 (d, J = 8.3 Hz, 1H), 6.21 (d, J = 15.3 Hz, 1H), 4.55 (s, 2H), 4.36 (t, J = 6.7 Hz, 1H), 4.03 (dd, J = 8.9, 5.4 Hz, 1H), 3.60 (s, 3H), 3.23 (s, 1H), 2.94 (s, 1H), 2.92 – 2.81 (m, 2H), 2.81 – 2.71 (m, 2H), 2.06 – 1.86 (m, 2H), 1.68 (dt, J = 13.4, 6.8 Hz, 1H), 1.38 (t, J = 7.4 Hz, 4H), 1.18 (d, J = 13.9 Hz, 1H), 0.99 (s, 1H), 0.89 (dd, J = 22.1, 6.6 Hz, 6H), 0.79 (t, J = 6.4 Hz, 7H).

^13^C NMR (100 MHz, DMSO) δ 173.4, 171.2, 166.2, 157.3, 146.5, 129.8, 128.3, 126.5, 58.0, 54.3, 51.9, 48.7, 41.9, 30.9, 25.3, 23.3, 22.0, 19.4, 18.2.

HRMS (ESI) m/z: [M + H]+ Calcd for (C_30_H_46_N_5_O_6_)+: 572.3443; Found 572.3457.

Methyl (((R)-1-(((5S,8S,E)-5-butyl-2,7-dioxo-1,6-diazacyclododec-3-en-8-yl)amino)-1-oxo-3-phenylpropan-2-yl)carbamoyl)-L-valinate (**C-3**).

Following general method C afforded **C-3** as a white solid. 29 mg yield: 50%

^1^H NMR (400 MHz, DMSO) δ 8.32 (d, J = 8.1 Hz, 1H), 7.87 (d, J = 8.0 Hz, 1H), 7.34 (t, J = 6.9 Hz, 1H), 7.27 – 7.21 (m, 2H), 7.21 – 7.12 (m, 3H), 6.77 (dd, J = 15.3, 4.7 Hz, 1H), 6.47 (d, J = 8.8 Hz, 1H), 6.36 (d, J = 8.3 Hz, 1H), 6.19 (d, J = 15.3 Hz, 1H), 4.60 – 4.49 (m, 2H), 4.29 (s, 1H), 4.03 (dd, J = 8.8, 5.4 Hz, 1H), 3.60 (s, 3H), 3.23 (d, J = 11.8 Hz, 1H), 2.97 – 2.71 (m, 3H), 2.05 – 1.86 (m, 2H), 1.56 (d, J = 9.4 Hz, 1H), 1.39 (s, 1H), 1.33 (d, J = 8.2 Hz, 2H), 1.32 – 1.24 (m, 3H), 1.22 – 1.14 (m, 1H), 0.99 (s, 1H), 0.87 (q, J = 7.6 Hz, 4H), 0.79 (t, J = 6.4 Hz, 6H).

^13^C NMR (100 MHz, DMSO) δ 173.4, 171.3, 171.2, 166.2, 157.3, 146.3, 138.0, 129.8, 128.3, 126.5, 119.3, 57.9, 54.3, 51.9, 51.5, 50.4, 38.3, 32.7, 30.9, 30.7, 30.6, 28.81, 22.37, 19.4, 18.1, 17.4, 14.2.

HRMS (ESI) m/z: [M + H]+ Calcd for (C_30_H_46_N_5_O_6_)+: 572.3443; Found 572.3457.

Methyl (((R)-1-(((5S,8S,E)-5-benzyl-2,7-dioxo-1,6-diazacyclododec-3-en-8-yl)amino)-1-oxo-3-phenylpropan-2-yl)carbamoyl)-L-valinate (**C-4**).

Following general method C afforded **C-4** as a white solid. 30 mg yield:49%

^1^H NMR (400 MHz, DMSO) δ 8.44 (d, J = 8.2 Hz, 1H), 7.85 (d, J = 8.1 Hz, 1H), 7.39 (t, J = 7.1 Hz, 1H), 7.35 – 7.25 (m, 5H), 7.25 – 7.10 (m, 7H), 6.76 (dd, J = 15.3, 4.6 Hz, 1H), 6.45 (d, J = 8.9 Hz, 1H), 6.36 – 6.25 (m, 2H), 4.60 (s, 1H), 4.52 (q, J = 7.3 Hz, 2H), 4.01 (dd, J = 8.8, 5.4 Hz, 1H), 3.58 (s, 3H), 3.29 (s, 1H), 3.00 – 2.87 (m, 3H), 2.83 (dd, J = 13.5, 6.4 Hz, 1H), 2.79 – 2.64 (m, 3H), 2.11 – 1.99 (m, 1H), 1.91 (dq, J = 13.3, 6.8 Hz, 1H), 1.37 (s, 1H), 1.18 (t, J = 13.6 Hz, 1H), 1.01 (s, 1H), 0.86 (d, J = 13.1 Hz, 1H), 0.78 (t, J = 6.8 Hz, 6H).

^13^C NMR (100 MHz, DMSO) δ 173.4, 171.2, 166.1, 157.3, 145.8, 138.8, 137.9, 129.7, 129.3, 128.7, 128.3, 126.8, 126.5, 119.7, 58.0, 54.3, 52.2, 51.9, 51.4, 38.7, 38.34, 30.90, 30.8, 30.5, 19.4, 18.1, 17.4.

HRMS (ESI) m/z: [M + H]+ Calcd for (C_33_H_44_N_5_O_6_)+: 606.3286; Found 680.3283.

Methyl (((R)-1-(((5S,8S,E)-5-(3-fluorobenzyl)-2,7-dioxo-1,6-diazacyclododec-3-en-8-yl)amino)-1-oxo-3-phenylpropan-2-yl)carbamoyl)-L-valinate (**C-5**).

Following general method C afforded C-4 as a white solid. 25 mg yield: 40%

^1^H NMR (400 MHz, DMSO) δ 8.43 (d, J = 8.2 Hz, 1H), 7.85 (d, J = 8.0 Hz, 1H), 7.37 (dt, J = 21.7, 7.3 Hz, 2H), 7.18 (dd, J = 17.5, 7.0 Hz, 8H), 7.05 (t, J = 8.8 Hz, 1H), 6.77 (dd, J = 15.4, 4.6 Hz, 1H), 6.45 (d, J = 8.9 Hz, 1H), 6.30 (dd, J = 15.2, 11.7 Hz, 2H), 4.61 (s, 1H), 4.52 (q, J = 7.2 Hz, 2H), 4.01 (dd, J = 8.8, 5.4 Hz, 1H), 3.58 (s, 3H), 3.27 (s, 1H), 3.00 – 2.63 (m, 6H), 2.01 (d, J = 14.0 Hz, 1H), 1.91 (q, J = 6.6 Hz, 1H), 1.37 (d, J = 14.2 Hz, 2H), 1.24 – 1.13 (m, 1H), 1.02 (s, 1H), 0.78 (t, J = 7.1 Hz, 6H).

^13^C NMR (100 MHz, DMSO) δ 173.4, 171.3, 166.1, 163.7, 157.3, 145.5, 141.7, 137.9, 130.6, 130.5, 129.7, 128.3, 126.5, 125.4, 119.9, 116.2, 116.0, 113.7, 58.0, 54.32, 52.04, 51.9, 51.4, 38.3, 38.2, 30.8, 19.4, 18.1.

HRMS (ESI) m/z: [M + H]+ Calcd for (C_33_H_43_FN_5_O_6_)+: 624.3192; Found 624.3231.

Methyl (((R)-1-(((5S,8S,E)-5-(3,5-difluorobenzyl)-2,7-dioxo-1,6-diazacyclododec-3-en-8-yl)amino)-1-oxo-3-phenylpropan-2-yl)carbamoyl)-L-valinate(**C-6**).

Following general method C afforded **C-6** as a white solid. 30 mg yield: 46%

^1^H NMR(DMSO, 400 MHz): δ = 8.45 (d, 1H, J=8.4 Hz), 7.88 (d, 1H, J=8.0 Hz), 7.48 – 7.37 (m, 2H), 7.25 – 7.11 (m, 6H), 7.06 (td, 1H, J=8.5, 2.6 Hz), 6.71 (dd, 1H, J=15.3, 4.6 Hz), 6.45 (d, 1H, J=8.9 Hz), 6.37 – 6.27 (m, 2H), 4.63 (d, 1H, J=7.9 Hz), 4.52 (t, 2H, J=7.4 Hz), 4.01 (dd, 1H, J=8.8, 5.4 Hz), 3.59 (s, 3H), 3.26 (t, 1H, J=10.2 Hz), 2.94 (dd, 2H, J=14.5, 6.8 Hz), 2.90 – 2.79 (m, 1H), 2.79 – 2.69 (m, 2H), 2.06 (t, 1H, J=13.5 Hz), 1.91 (dq, 1H, J=13.3, 6.7 Hz), 1.37 (d, 1H, J=14.2 Hz), 1.20 (d, 1H, J=14.0 Hz), 1.02 (s, 1H), 0.84 (d, 1H, J=12.4 Hz), 0.78 (t, 6H, J=6.9 Hz)

^13^C NMR (100 MHz, DMSO) δ 173.4, 171.3, 171.3, 166.0, 160.3, 157.3, 144.8, 137.9, 132.6, 132.5, 129.7, 128.3, 126.5, 121.7, 120.4, 112.0, 111.8, 104.4, 104.1, 58.0, 54.2, 51.9, 51.5, 50.7, 38.3, 31.3, 30.9, 30.7, 30.4, 19.4, 18.1, 17.4.

HRMS (ESI) m/z: [M + H]+ Calcd for (C_33_H_42_F_2_N_5_O_6_)+: 642.3098; Found 642.3101.

### General Procedure for the Solid Phase Synthesis of Syringolin Analogs

The Tentagel^®^ resin (loading 0.26 millimole/g) was weighed in the container. The resin was swelled in DMF (1 g resin/10 mL) for 1 h. The solvent was removed, and the resin was treated with a 0.2 M mixture of 5-(4-formyl-3,5-dimethoxyphenoxy)pentanoic acid, HATU (3 eq),DIPEA (6eq) in DMF and the reaction was agitated for 2 h. The solvent was removed and wash with DMF (3 times), MeOH (3 times), and DCM (3 times).

The resin was treated with 10ml of mixture of 1% AcOH/DMF and 10 eq. of Fmoc-Lys-*O*-Allyl, the mixture was agitated for 1. Add 20 eq. NaCNBH3 to the mixture and the reaction was agitated overnight. The solvent was removed, and the resin was washed with DMF (3 times),MeOH (3 times), and DCM (3 times).

2-(diethoxyphosphoryl)acetic acid (10 eq) was dissolved in 10ml in DCM, CDI (10 eq) was added in the solution and stirred for 20 min. The resin was treated by the aforementioned mixture and agitated for 2h. The solvent was removed, and the resin was washed with DMF (3 times), MeOH (3 times), and DCM (3 times).

Pd(PPh_3_)_4_ (0.1 eq) and PhSiH_3_ (5 eq) were dissolved in 5 mL DCM. The resin was treated by the aforementioned mixture and agitated for 15 min. Repeat. The solvent was removed, and the resin was washed with DMF (3 times), MeOH (3 times), and DCM (3 times). Then, the resin was treated with 0.2M of mixture of corresponding amino alcohol (4 eq) HOBt (4 eq), PyBop (4 eq), NMM (8 eq), and the reaction was agitated for 2h. The solvent was removed, and the resin was washed with DMF (3 times), MeOH (3 times), and DCM (3 times).

Dess-Martin periodinane (5 eq) was dissolve in DCM/DMF (10:1). The resin was treated by aforementioned mixture and was agitated for 2.5 h. The solvent was removed, and the resin was washed with DMF(3 times), MeOH (3 times), and DCM (3 times). Repeat. Then, Zn(OTf)_2_ (2.2 eq), TMEDA (1.2 eq), and TEA (4 eq) was added in THF. The suspension was vortexed for 15 min and added to resin. The reaction was agitated overnight. The resin was washed with DMF (3 times), MeOH (3 times), and DCM (3 times).

The resin was treated with 20% 4-methyl-piperidine in DMF, and the reaction was agitated for 15 minutes. The solvent was removed, and the resin was washed with DMF (3 times), MeOH (3 times), and DCM (3 times). Repeat. The resin was treated by the 0.2 mixture of corresponding Fmoc-amino acid (3 eq), HATU (3 eq) and DIPEA (6 eq) in DMF and the reaction was agitated for 2h. The solvent was removed, and the resin was washed with DMF (3 times), MeOH (3 times), and DCM (3 times).

The resin was treated with 20% 4-methyl-piperidine in DMF, and the reaction was agitated for 15 minutes. The solvent was removed, and the resin was washed with DMF (3 times), MeOH (3 times), and DCM (3 times). Repeat. Then, the resin was treated by the mixture of methyl (S)-2-isocyanato-3-methylbutanoate (3 eq) and DIPEA(6 eq) in DCM, the reaction was agitated for 4h.

Resin cleavage is achieved by the treatment of mixture of 95%TFA, 2.5% TIPS, 2.5% water and was agitated for 2h. The solution was filtered and evaporated. The final product was purified by prep-TLC with DCM:MeOH (10:1), affording the product as a white solid (yield: 30%).^1^H NMR is agreement with literature.^49^

^1^H and ^13^C NMR data and SMILES strings are available for all analogs in the Supporting Information. Purity was assessed by HPLC, and all compounds possessed greater than 95% purity.

### Procedure for Parasitemia Assay

All experiments in this study were performed using *P. falciparum* strain W2 (MRA-157). Parasites were cultured at 2% hematocrit in RPMI_c_ supplemented with 0.25% Albumax, 2g/L sodium bicarbonate, 0.1mM hypoxanthine, 25mM HEPES (pH 7.4), and 50μg/L gentamycin. Incubators were maintained at 37°C, 5% O_2_, and 5% CO_2_. Parasitemia was maintained below 5% to ensure the health and active replication of parasites.

All compounds were resuspended in pure DMSO at 10 mM and stored at −20°C in 10 μL aliquots. A 200 μM solution of the compounds was then generated and serially diluted √10-fold into a 2% DMSO in 1X PBS solution to maintain the solvent concentration across all wells. Cultures were initiated at 0.5% hematocrit and 0.8% rings, and 190 μL was dispensed into rows of a 96-well U-bottom culture plate. 10μL of the dilution series was then pipetted to mix with the culture, resulting in a 20-fold dilution ranging from 10 μ M to 1 nM. Plates were incubated at 37°C, 5% O_2_, and 5% CO_2_ for 72 hours, fixed in 1:1 in 2% Paraformaldehyde (PFA) for 1 hour at room temperature, and then stained overnight in 50 nM YOYO-1. To quantify parasitemia, each well plate was run on a BDFACSSymphony A1 flow cytometer with a BB15A laser. Each sample was run in technical and biological triplicates, with a collection threshold of 50,000 events. Gating and IC_50_ calculations were then carried out using FlowJo and GraphPad Prism.

### Expression and purification of recombinant *Pf*20S proteasome

Recombinant *Pf*20S was expressed using the baculovirus–insect cell system, as previously described.^30, 63^ Cells were harvested 72 h post-infection, lysed in 50 mM Tris-HCl (pH 7.5), 150 mM NaCl, 1 mM DTT, and 1 mM EDTA, and clarified by centrifugation. The proteasome complexes were purified using tandem Strep-Tactin XT affinity columns, eluted with 50 mM biotin in the same buffer, and further separated by size-exclusion chromatography on a Superose 6 Increase column equilibrated in 50 mM HEPES (pH 7.5), 150 mM NaCl, and 1 mM DTT. Enzymatically active fractions were pooled, concentrated, and stored at –80 °C until use.

### Proteasome Inhibition Assays

Initially, a single inhibitor concentration was tested, which demonstrated inhibition of β5 activity. Subsequently, kinetic inhibition (Ki) studies were performed with β5 only. For preincubation assays, 12.5 μM inhibitor was incubated (30 min, room temperature) with either 1 nM human c20S or 1 nM *Pf* 20S, DMSO alone was used as a control. c20S was activated for 1 h at room temperature with 100 nM PA28α, and assays were performed in 50 mM HEPES (pH 7.5) and 1 mM DTT. Substrate hydrolysis was monitored using Suc-LLVY- AMC (β5, 65 μM), Z-VLR-AMC (β2, 30 μM), and Z-LLE- AMC (β1, 80 μ M). In parallel, *Pf*20S was activated for 1 h at room temperature with 200 nM PA28α, and assays were performed in 50 mM HEPES (pH 7.5), 5 mM MgCl_2_, 1 mM DTT, and 0.01% BSA. Substrate hydrolysis was monitored using Ac-FNKL- AMC (β5, 50 μM), Z-VLR- AMC (β2, 25 μM), and Ac-nPnD- AMC (β1, 60 μM).

For K_i_ determinations, the inhibitor concentrations ranging from 0.603 to 3.125 μM and DMSO control were pre-mixed with substrate, and enzyme was added last. Fluorescence (RFU) was immediately monitored for 3 h. Apparent first-order rate constants (k_obs_) were calculated and plotted against inhibitor concentration to derive K_i_ values. All reactions were performed in a final volume of 8 μL per well. Inhibitors were dispensed using an Echo650 liquid handler (Beckman Coulter, Brea, CA, USA). Pre–steady-state kinetic measurements were carried out for the irreversible inhibitor, and data were analyzed using GraphPad Prism v10.4.1. All assays were performed in triplicate in 384-well black plates (Greiner Bio-One, Monroe, NC, USA) at 37 °C. Fluorescence was recorded with a Synergy HTX Multi-Mode Microplate Reader (BioTek, Winooski, VT, USA) using excitation at 360 nm and emission at 460 nm.

### Associated Content

Synthetic procedures, NMR and mass spectra, and the Molecular Formula Strings (CSV) of key intermediates and final compounds are included in the supporting information file. The experimental procedures for the enzymology and cryo-electron microscopy are also in the supporting information file.

The atomic coordinates for the cryo-EM structures have been deposited in RCSB with PDB IDs 9YUY and 9YUZ.

## Supplementary Material

Gu, J Med Chem 2006 Supporting Information

## Figures and Tables

**Figure 1.**
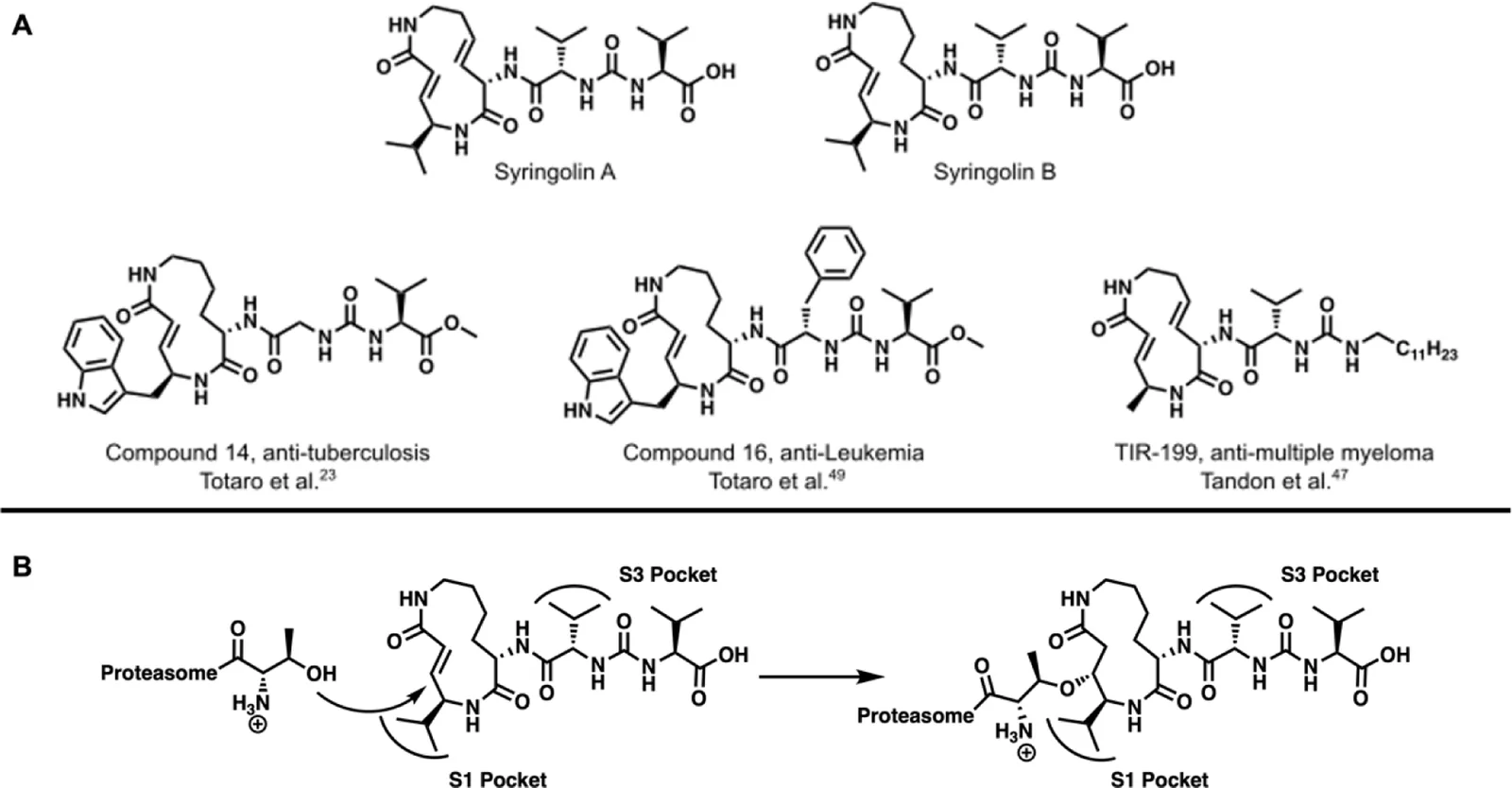
A) Gröll model of syringolin B binding to the Saccharomyces cerevisiae 20S proteasome^33^ B) Structures of the syringolin A and syringolin B natural products alongside synthetic analogs having proteasome inhibition activity.

**Figure 2.**
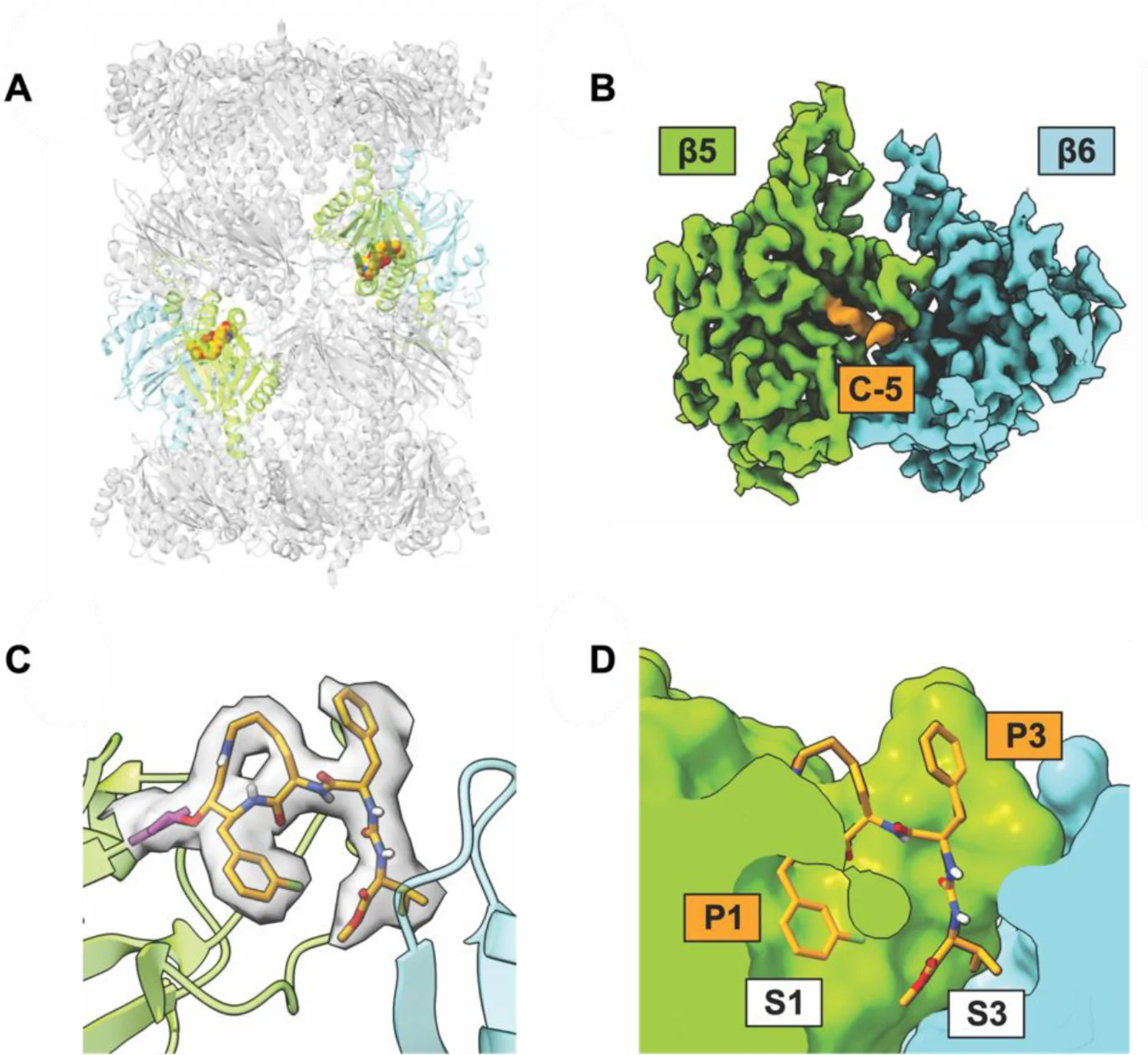
Cryo-EM structure of **C-5** in complex *Pf* 20S. (A) Location of the **C-5** adducts (orange) within the 20S holoenzyme. The β5 and β6 subunits are in green and blue, respectively. (B) Density map reveals the presence of **C-5** at the β5-β6 interface. (C) **C-5** modeled into the density. The Thr1 nucleophile is in magenta. (D) Surface representation of 20S indicating the locations of the S1 and S3 pockets in 20S, along with the P1 and P3 substituents of **C-5**.

**Figure 3.**
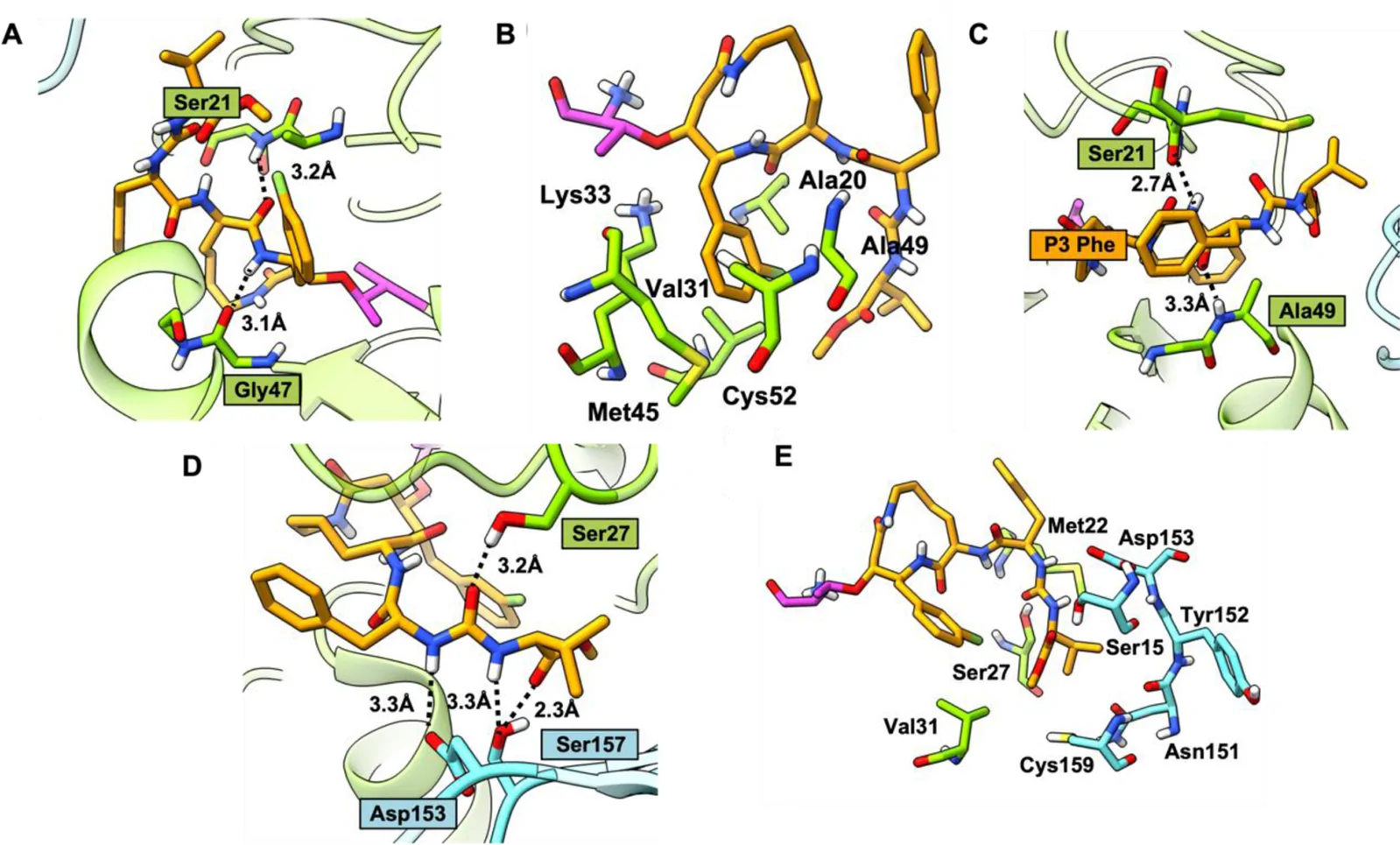
Interactions between **C-5** and *Pf* 20S. (A) Hydrogen bonding interactions involving the macrocyclic amide proximal to the P1 substituent. (B) Residues forming the S1 pocket, which binds the P1 sidechain. (C) Hydrogen bonding interactions involving the P3 amide. The P3 Phe sidechain, which forms a cover over these hydrogen bonds, is labeled. (D) Hydrogen bonding interactions involving the ureido tail and terminal valine methyl ester. (E) Residues forming the S3 pocket, which binds the ureido tail, including the terminal valine methyl ester.

**Figure 4.**
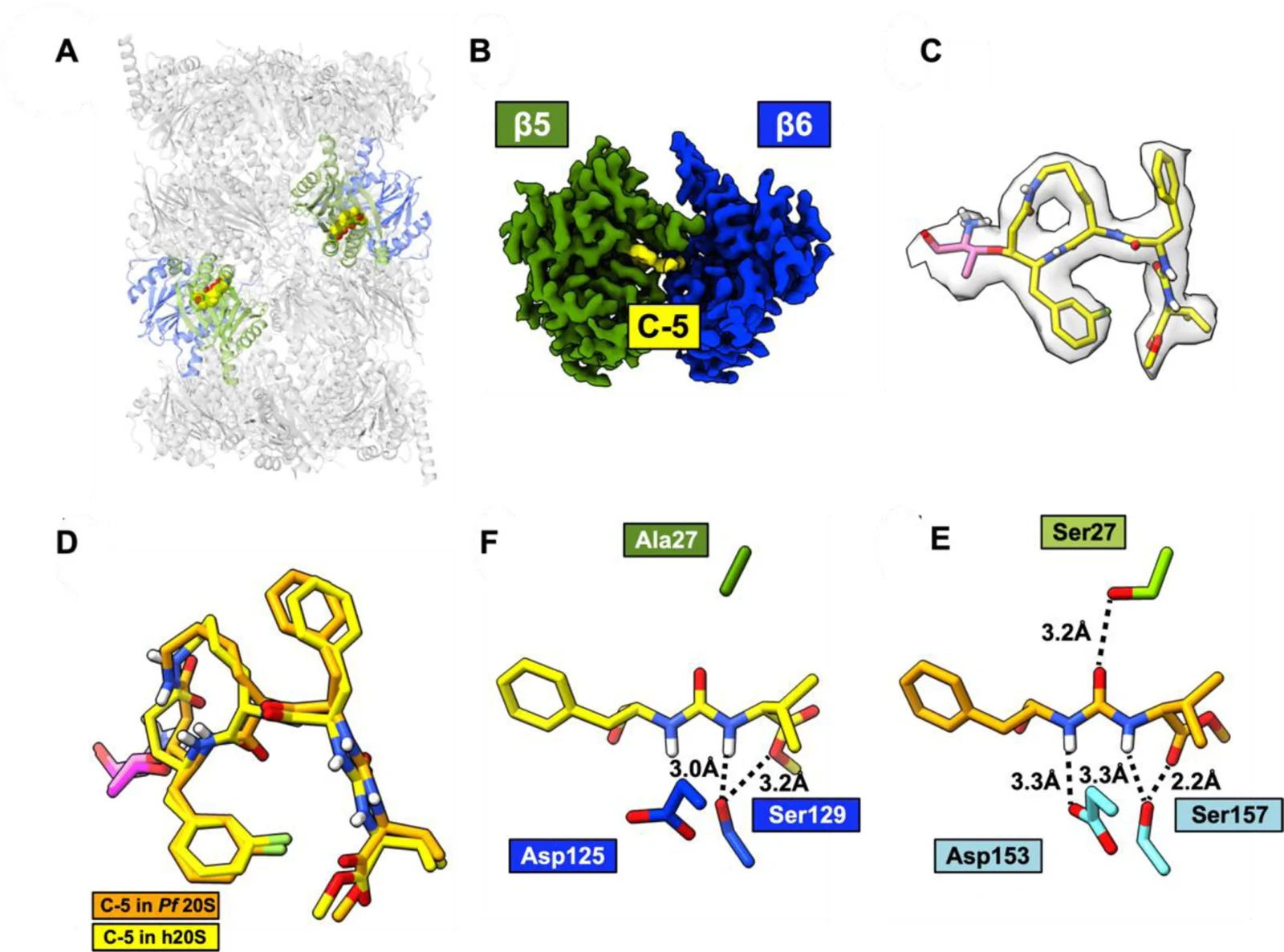
Cryo-EM structure of **C-5** in complex with h20S and comparison with the *Pf* 20S structure. (A) Location of the **C-5** adducts (yellow) within the h20S holoenzyme. The β5 and β6 subunits are in dark green and dark blue, respectively. (B) Density map reveals the presence of **C-5** at the β5-β6 interface. (C) **C-5** modeled into the density. The Thr1 nucleophile is in pink. (D) Comparison of the bound poses of **C-5** in h20S (yellow) and in *Pf* 20S (orange). (E) Hydrogen bonding interactions involving the ureido group and terminal valine methyl ester of **C-5** with h20S. (F) Hydrogen bonding interactions involving the ureido group and terminal valine methyl ester of **C-5** with *Pf* 20S.

**Figure 5.**
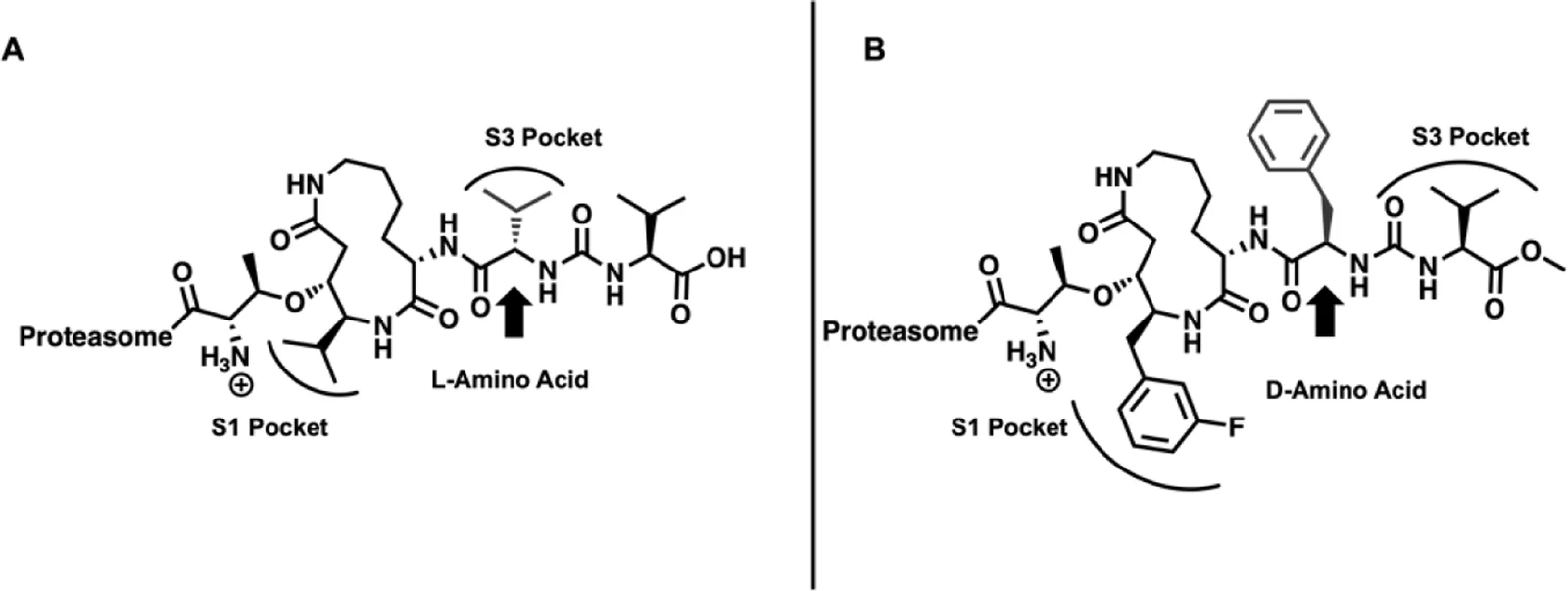
Two models for proteasome active site occupancy. (A) The L-amino acid in the peptidourea side chain is oriented in the S3 pocket. (B) A syringolin with a D-amino acid in the peptidourea side chain has the terminal valine methyl occupying the S3 pocket.

**Scheme 1.**
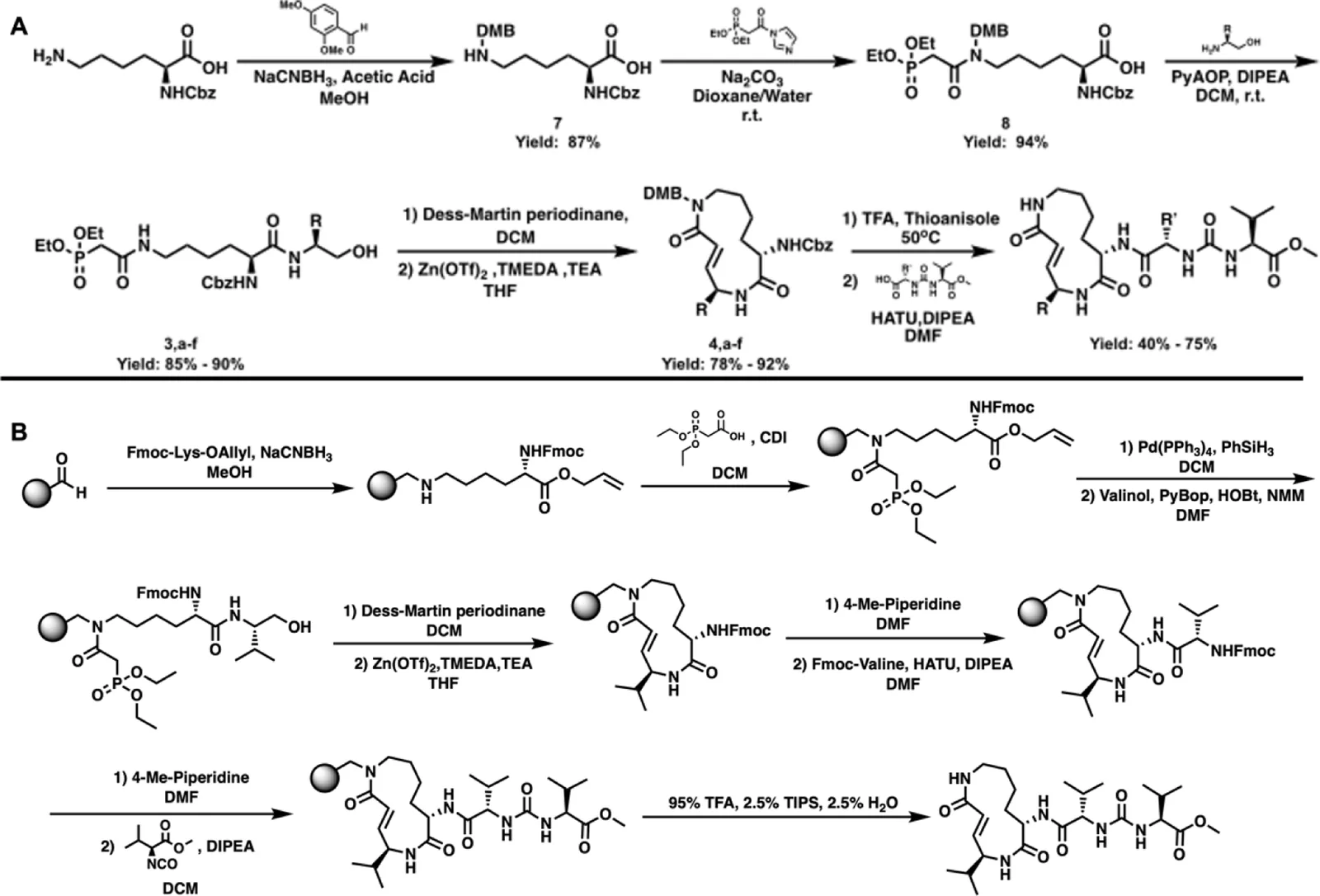
A) Optimized solution-phase route for syntheses of syringolin B analogs. B) Ten-step solid phase synthesis of syringolin B methyl ester

**Table 1.** Macrocyclization yields for substrates having secondary and tertiary amides.

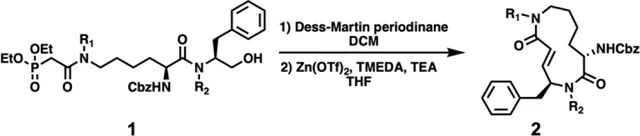			
Reaction	R_1_	R_1_	Yield
a	H	H	19%
b	H	DMB	No Product*
c	DMB	H	83%
d	Me	H	36%

* The aldehyde precursor of macrocyclization was recovered

**Table 2.** Macrocyclization substrate scope

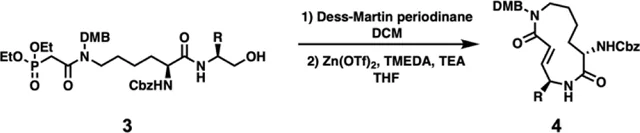			
Entry	R	Yield Without DMB Group	Yield With DMB Group
a	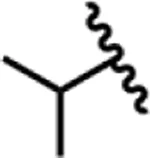	58%	92%
b	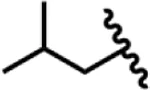	39%	82%
c	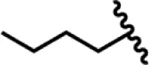	30%	84%
d	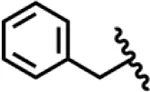	19%	83%
e	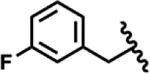	39%	78%
f	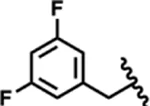	30%	79%

**Table 3.** Macrocyclization yields for substrates having different *N*-protecting groups.

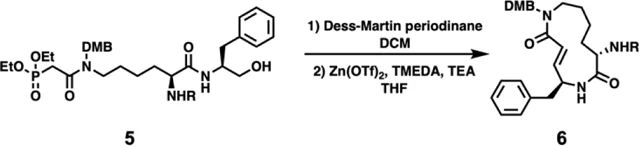			
Entry	Protecting Group R		Yield
a	Cbz	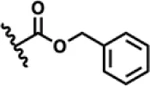	83%
b	Fmoc	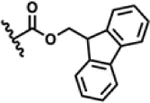	78%
c	*S*-Me-Cbz	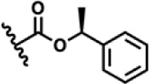	58%
d	*R*-Me-Cbz	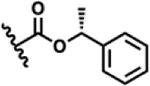	74%
e	Diphenyl-methyl	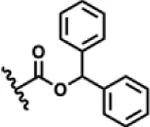	64%
f	Boc	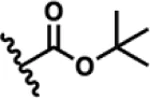	43%

**Table 4.** Species-Selective Cytoxicities of Syringolin B Methyl Ester and An Diastereomer

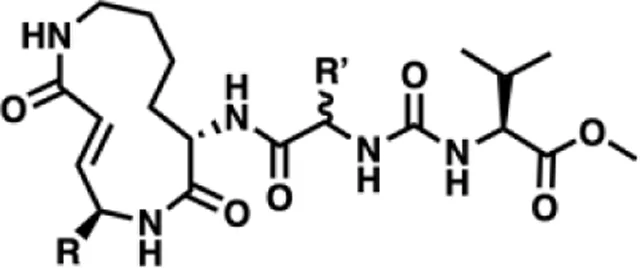					
Compound	R	R’	Cell Potency		
			*Plasmodium W2* EC_50_ (nM)	HFF EC_50_ (nM)	Selective Index
Syringolin B-methyl ester	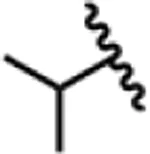	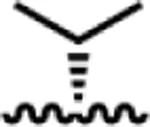	174 ± 28	2590 ± 510	15
A-1	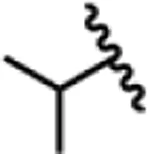	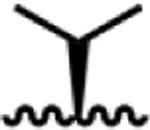	342 ± 35	>25,000	>73

**Table 5.** Anti-*Plasmodium* activities of eighteen syringolin analogs at 100 nM

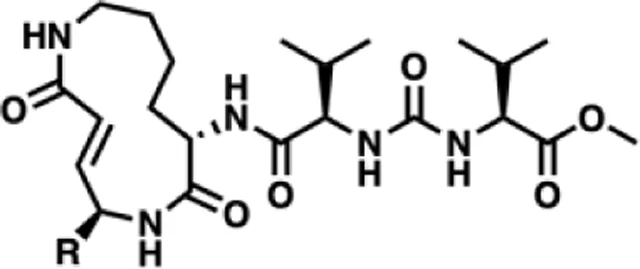			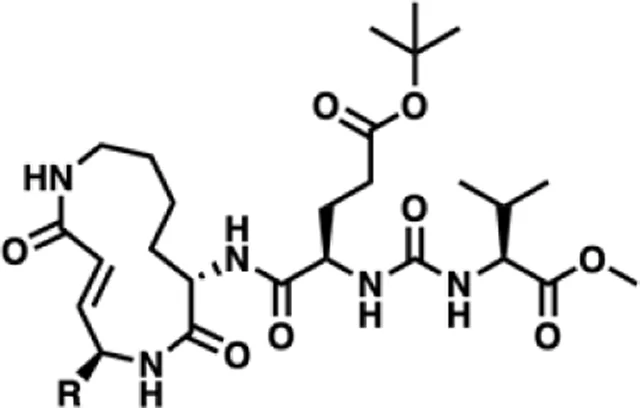			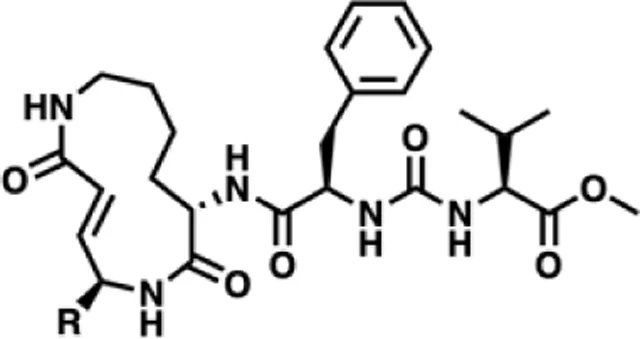		
		
Compound	R	Parasitemia	Compound	R	Parasitemia	Compound	R	Parasitemia
		
A-1	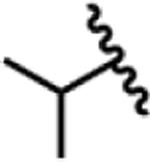	101%	B-1	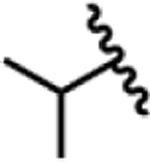	98%	C-1	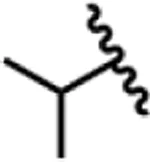	42%
		
A-2	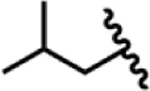	81%	B-2	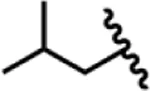	105%	C-2	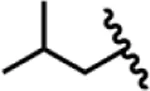	66%
		
A-3	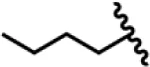	65%	B-3	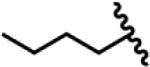	85%	C-3	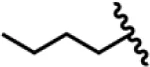	52%
		
A-4	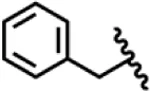	9.5%	B-4	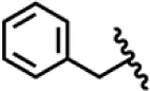	61%	C-4	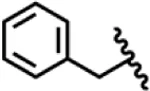	33%
		
A-5	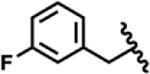	98%	B-5	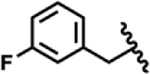	9.7%	C-5	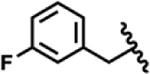	2.8%
		
A-6	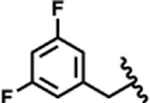	98%	B-6	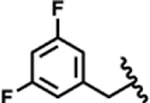	52%	C-6	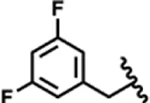	70%
		

**Table 6.** Proteasome Inhibition and Cytotoxicity Data for syringolin B methyl ester and five analogs (*Plasmodium* versus Human)

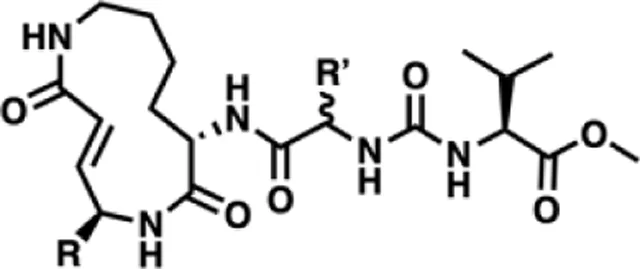								

Compound	R	R’	Cell Potency			Proteasome Inhibition		
			*Plasmodium W2* EC_50_ (nM)	HFF EC_50_ (nM)	Selective Index	*Pf* kinact/Ki M-1*s-1	*Hs* kinact/Ki M-1*s-1	Selective Index
Syringolin B-methyl ester	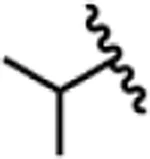	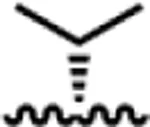	174 ± 28	2590 ±510	15	930 ±210	1,400 ±230	0.7

A-1	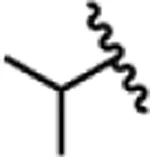	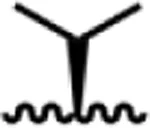	342 ±35	>25,000	>73	1,570 ±160	N.D.	N.D.

A-4	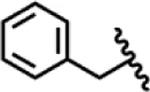	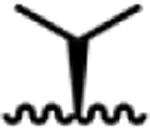	41.0±6.8	>500,000	>12,000	8,620°160	700 ± 70	12

A-5	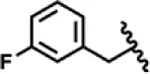	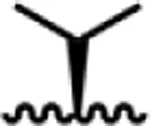	219 ± 76	>500,000	>2,300	10,740 ± 4,100	1,740 ± 560	6.2

B-5	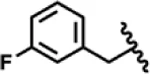	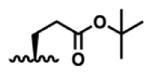	54.5 ± 18.0	246,000 ± 33,000	4500	48,000 ± 22,000	4,100 ± 800	11

C-5	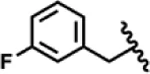	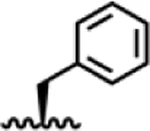	20.1 ± 5.7	>500,000	>25,000	53,300 ± 3,900	1,180 ± 320	45

## Abbreviations

DMB: dimethoxybenzyl
HATU: hexafluorophosphate azabenzotriazole tetramethyl uronium
HFF: human foreskin fibroblasts
HWE: Horner–Wadsworth–Emmons
MBHA: 4-methylbenzhydrylamine
*Pf*: *Plasmodium falciparum*
TEA: triethylamine
