# Supplementary material for: An Optimized Route to the Syringolin Natural Products Enables Combinatorial Synthesis of Selective, Bioactive Inhibitors of the Plasmodium falciparum 20S Proteasome
Source: J Med Chem. Author manuscript; Available in PMC 2026 Nov 3. (PMC13730187; doi:10.1021/acs.jmedchem.5c03223)
Supplement: Supplementary file 1 [file NIHMS2184729-supplement-Gu__J_Med_Chem_2006_Supporting_Information.pdf]

Supporting Information

An Optimized Route to the Syringolin Natural Products Enables Combinatorial Synthesis of Selective, Bioactive Inhibitors of the *Plasmodium falciparum* 20S Proteasome

Xilin Gu,[1] Pavla Fajtova,[2] Nicholas L. Yan,[3] Aditi Saxena,[4,5] Fan Fei,[1] Jiyun Zhu,[6,7] Eric Tse,[3] Arthur Melo,[3] Euna Yoo,[6,8] Nathan H. Buchwald,[9] Daniel R. Southworth,[3,10] Matthew Bogyo,[6] Joseph L. Derisi,[4,8] Jason E. Gestwicki,[1,3] Anthony J. O'Donoghue,[2] Jason K. Sello[1,*]

1. Department of Pharmaceutical Chemistry, University of California, San Francisco, San Francisco, CA, 94158 USA.
2. Skaggs School of Pharmacy and Pharmaceutical Sciences, University of California San Diego, La Jolla, CA, 92093,USA
3. Institute for Neurodegenerative Diseases, University of California, San Francisco, CA, 94158 USA
4. Chan Zuckerberg Biohub San Francisco, San Francisco, California,94158 USA.
5. Current Address: Department of Immunology and Infectious Disease, Harvard T.H. Chan School of Public Health, Boston, MA, 02115 USA
6. Department of Pathology, Stanford School of Medicine, Stanford University, Stanford, CA 94305 USA
7. Current Address: Department of Biological Chemistry and Molecular Pharmacology, Harvard Medical School, Boston, MA, 02115 USA
8. Current address: Center for Cancer Research, National Cancer Institute. Frederick, MD, 21702-1201 USA
9. Department of Chemistry, Brown University, Providence, RI 02912, United States
10. Department of Biochemistry and Biophysics, UCSF, San Francisco, California, 94143 USA.

* Corresponding Author: Jason K.Sello: email: Jason.Sello@UCSF.edu

Table of Content

Synthetic Methods S3
- General Information S3
- Synthesis of the precursors of the macrocyclization substrates S3
  - Synthesis of ε-N-methyl-Lysine S3
  - Synthesis of N[2]-((benzyloxy)carbonyl)-N[6]-(2,4-dimethoxybenzyl)-L-lysine S5
  - Synthesis of the 2,5-dioxopyrrolidin-1-yl carbonates S6
  - Synthesis of the diethoxyphosphorylacetyl-Lysine analogs S7
- Synthesis of the macrocyclization substrates S8
- Synthesis of the Syringolin-B Macrolactam S13
- Synthesis of the D-Glu(OtBu) sidechain S18
- Synthesis of the Syringolin-Based Proteasome Inhibitor Analogs S18
- Condition Screen for the Deprotection of Macrolactam S28
- General Procedure for Syringolin Analog Solid Phase Synthesis S28
- List of Molecular Formula Strings S31

Procedure for Parasitemia Assay..S34
Kinetic Evaluation of Syringolin-based Proteasome Inhibitors......................................S35
Procedure for Human Toxicity Assay...S37
Cryo-EM method...S38
UPLC-MS for Syringolin Analogs..S44
^{1}H and ^{13}C NMR Spectrum..S48

Synthetic Methods

General Information

All reactions were conducted, when required, in oven-dried round-bottom flasks under an argon atmosphere, using anhydrous solvents and magnetic stirring. Anhydrous solvents were obtained from Fisher Scientific or Sigma-Aldrich, while all reagents were sourced from commercial suppliers without further purification, including AK Scientific, Fisher Scientific, Ambeed, Chem-Impex International, ChemScene, SigmaAldrich, Combi-Blocks, and TCI America. Solvents such as ethyl acetate (EtOAc), acetone (Ace), hexanes (Hex), and methylene chloride (DCM), methanol (MeOH), used for column chromatography and reaction work-ups, were purchased from VWR International, Sigma-Aldrich, and Fisher Scientific. Compounds were visualized with UV light (254 nm).

Column chromatography was conducted with a Teledyne NextGen 300+ flash chromatography system. Rotary evaporation was carried out at 30°C, 40°C, and 60°C under appropriate pressure, considering the solvent used. Nuclear magnetic resonance (NMR) spectra for ^{1}H and ^{13}C NMR were recorded on a Bruker Avance III HD 400 MHz spectrometer at 298 K, referenced to the residual solvent resonance peaks ($CHCl_3$: 7.26 ppm for ^{1}H and 77.00 ppm for ^{13}C, DMSO: 2.50 ppm for ^{1}H and 39.52 ppm for ^{13}C). Multiplicities are denoted with the following abbreviations: s = singlet, d = doublet, t = triplet, q = quartet, quin = quintet, m = multiplet, or combinations thereof. NMR spectra were processed by either Mestrelab Mnova or Bruker TopSpin. High-resolution mass spectra (HRMS) were acquired using a Waters Xevo G2-XS time-of-flight mass spectrometer. Compound purity and reaction progress were analyzed using a Waters Acquity UPLC-MS system. All reported yields are the average of experiments performed in triplicate.

Synthesis of the precursors of the macrocyclization substrates

Synthesis of ε-N-methyl-Lysine

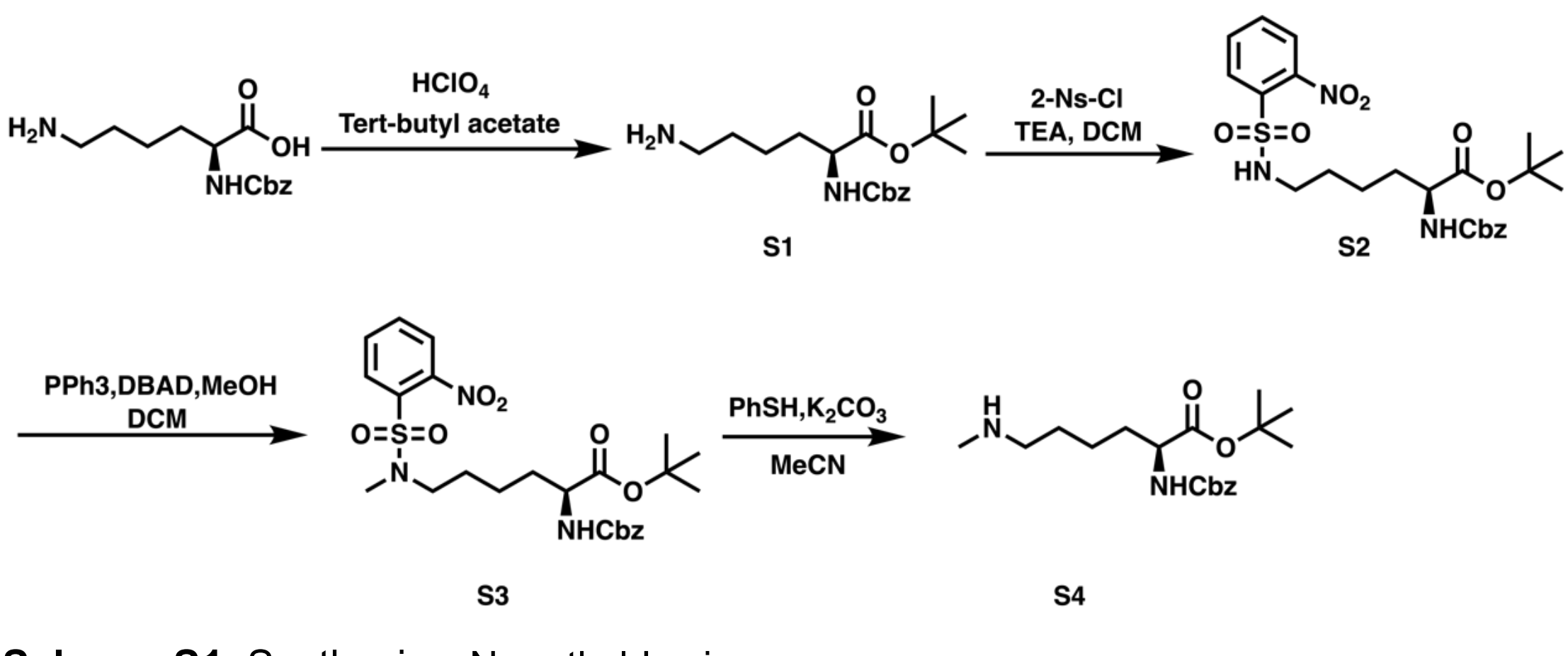

Scheme S1. Synthesis ε-N-methyl-Lysine

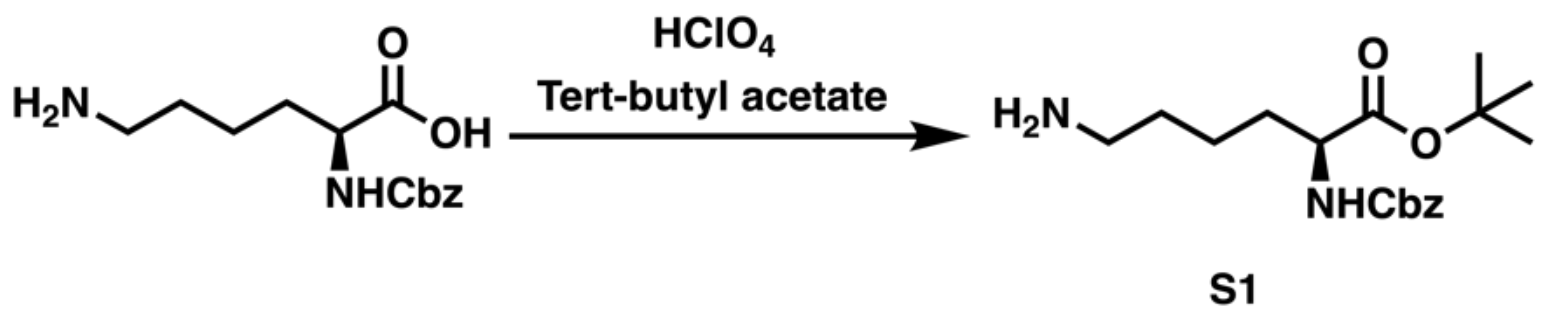

***tert*-butyl ((benzyloxy)carbonyl)-L-lysinate (S1)**

Z-Lysine(2.8 g,10 mmol,1 eq) was added in *tert*-butyl acetate(15 mL). The suspension was cooled to 0°C, and $HClO_4$ (70% aqueous solution,1.42 g,10 mmol,1.0 eq) was added dropwise. The resulting solution was stirred at 0 °C for 1h and at room temperature for 15h. After being diluted with 15mL 1M aqueous HCl solution, the solution was washed with ethyl acetate(2 x 15 mL) and the organic phase was discarded. The aqueous phase was adjusted to pH = 9 with sat. K_2CO_3 solution and was extracted with DCM (3 x 30 mL). The combined organic layers were dried(Na_2SO_4), filtered, and concentrated. The resulting colorless oil was used without further purification.

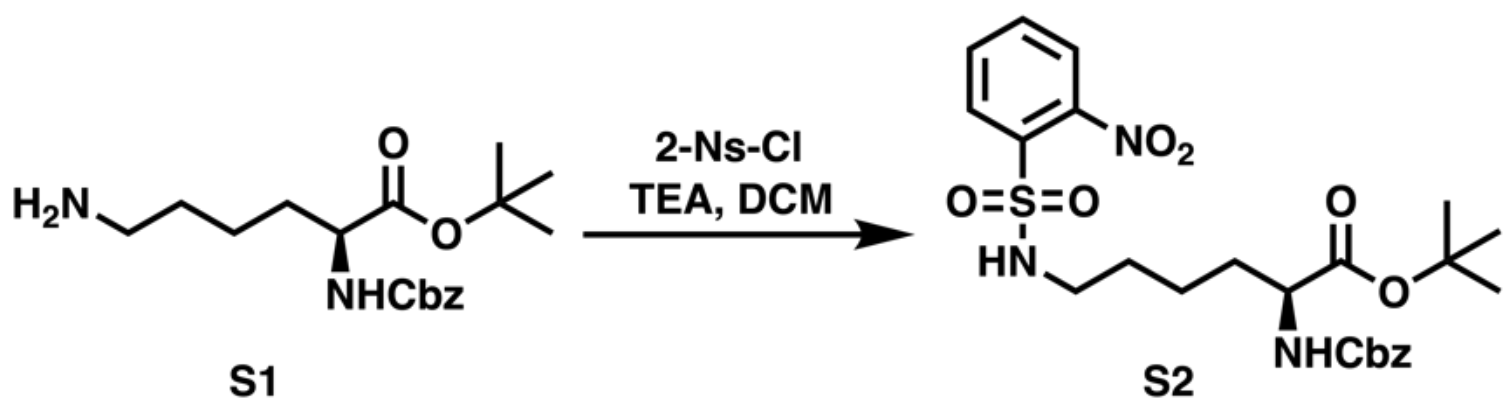

***tert*-butyl N^2-((benzyloxy)carbonyl)-N^6-((2-nitrophenyl)sulfonyl)-L-lysinate (S2)**

tert-butyl ((benzyloxy)carbonyl) -L-lysinate (**S1**, 1.68 g, 5 mmol,1eq) was dissolved in DCM, and 2-nosyl chloride (1.37 g,6.5 mmol,1.3 eq) was added, followed by the addition of TEA(1.01 g, 10 mmol,2 eq). The resulting solution was stirred at room temperature overnight and the solvent was removed *in vacuo*. The resulting oil was redissolved in ethyl acetate, and the resulting solution was washed with 1 M HCl (3 × 50 mL) and brine (50 mL). The organic phase was dried (Na_2SO_4), filtered, and concentrated. The resulting residue was purified by flash column with hexanes and ethyl acetate to afford **S2** as a colorless oil (2.5 g, Yield: 97%).

^{1}H NMR (400 MHz, $CDCl_3$): δ8.17-8.05(m,1H),7.92-7.83(m,1H), 7.80-7.66(m,2H), 7.43-7.30(m,5H), 5.45-5.26(m,2H), 5.18-2.00(s,2H), 4.33-4.18(m,1H), 3.19-2.94(q,2H), 1.87-1.73(m,1H), 1.69-1.53(m,3H), 1.52-1.42(d,9H), 1.41-1.34(m,1H)

^{13}C NMR (100 MHz, $CDCl_3$): δ171.3, 155.9, 133.6, 133.5, 132.7,131.0, 128.5, 128.1, 128.1, 125.3, 82.3, 66.9, 53.9, 43.4, 32.3, 29.0, 27.9, 21.9,

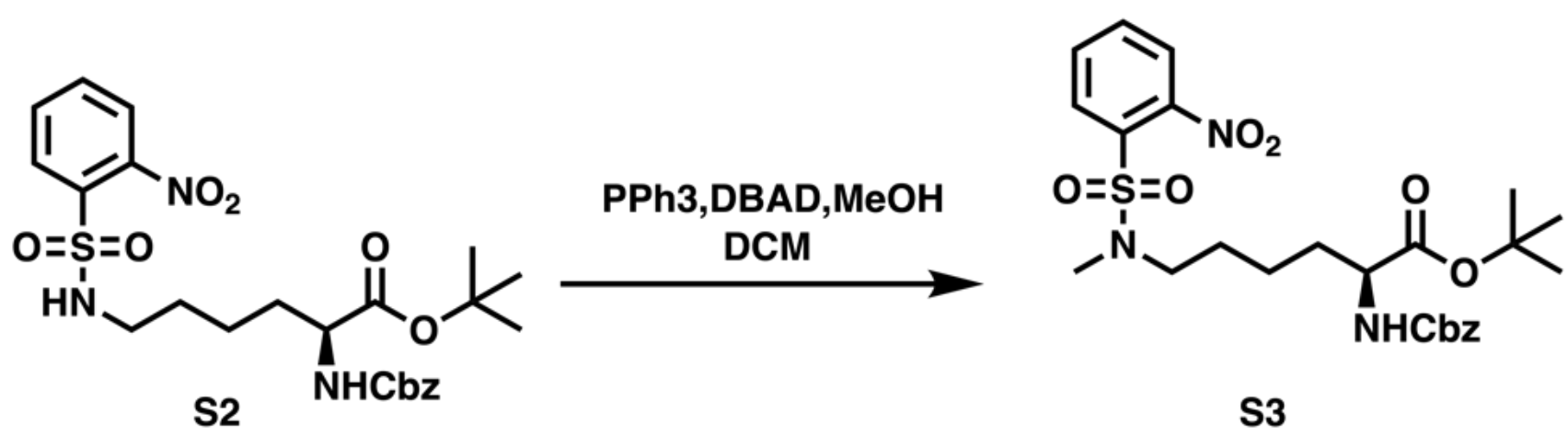

***tert*-butyl N^2-((benzyloxy)carbonyl)-N^6-((2-nitrophenyl)sulfonyl)-L-lysinate (S3)**

tert-butyl ((benzyloxy)carbonyl)-L-lysinate (**S2**, 1.68 g,5 mmol,1 eq) was dissolved in DCM, and 2-nosyl chloride(1.37 g,6.5 mmol,1.3 eq) was added, followed by the addition of TEA(1.01 g, 10 mmol, 2 eq). The resulting solution was stirred at room temperature overnight, and the solvent was removed *in vacuo*. The resulting oil was redissolved in ethyl acetate, and the resulting solution was washed with 1 M HCl (3 × 50 mL) and brine (50 mL). The organic phase was dried (Na_2SO_4), filtered, and concentrated. The resulting residue was purified by flash column with hexanes and ethyl acetate to afford **S3** as a colorless oil (2.5 g, Yield: 97%).

^{1}H NMR (400 MHz, $CDCl_3$): δ8.17-8.05(m,1H),7.92-7.83(m,1H), 7.80-7.66(m,2H), 7.43-7.30(m,5H), 5.45-5.26(m,2H), 5.18-2.00(s,2H), 4.33-4.18(m,1H), 3.19-2.94(q,2H), 1.87-1.73(m,1H), 1.69-1.53(m,3H), 1.52-1.42(d,9H), 1.41-1.34(m,1H)

^{13}C NMR (100 MHz, $CDCl_3$): δ171.3, 155.9, 133.6, 133.5, 132.7,131.0, 128.5, 128.1, 128.1, 125.3, 82.3, 66.9, 53.9, 43.4, 32.3, 29.0, 27.9, 21.9,

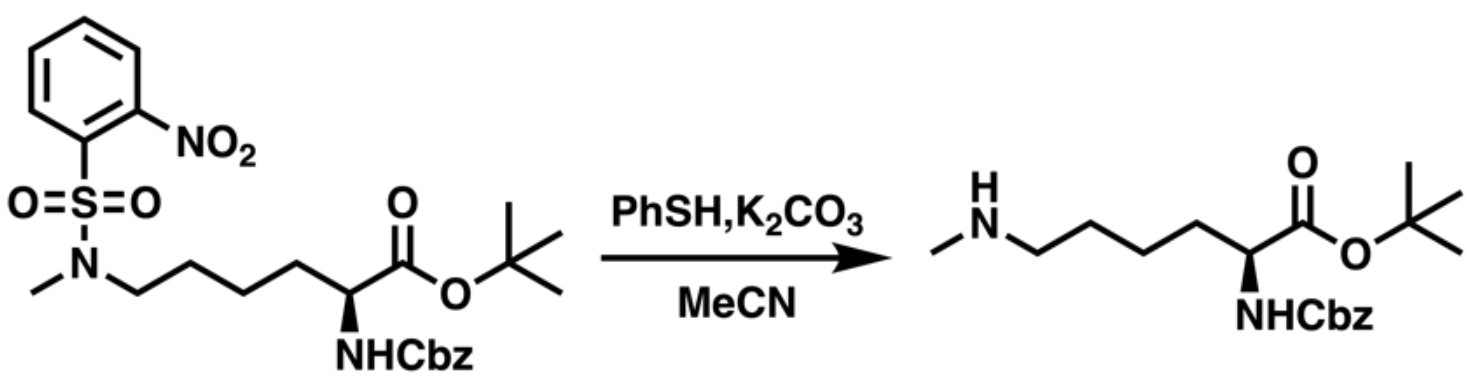

S3 S4

tert-butyl N²-((benzyloxy)carbonyl)-N⁶-methyl-L-lysinate (S4)

tert-butyl N²-((benzyloxy)carbonyl)-N⁶-methyl-N⁶-((2-nitrophenyl)sulfonyl)-L-lysinate(**S3**, 2.82 g, 5.27 mmol,1 eq) was dissolved in acetonitrile, followed by the addition of thiophenol (697.3 mg, 6.32 mmol, 1.2 eq) and K_2CO_3 (2.19 g,15.81 mmol,3 eq). The solution was stirred at room temperature overnight. After being diluted with water, the organic phase was extracted with DCM(3 x 50mL). The combined organic layers were dried (Na_2SO_4), filtered, and concentrated. The resulting residue was purified by flash column by DCM:MeOH=10:1+5%TEA to afford **S3** as a colorless oil(1.549g, yield: 84%).

^{1}H NMR (400 MHz, $CDCl_3$): δ 7.47-7.29(m,5H), 5.60-5.41(t,1H), 5.17-5.04(d,2H), 4.40-4.16(s,1H), 2.56-2.44(s,3H), 1.94-1.76(m,1H), 1.75-1.58(m,3H), 1.52-1.32(11H)

^{13}C NMR (100 MHz, $CDCl_3$): δ171.4, 155.9, 136.3, 128.5, 128.1, 82.1, 66.8, 54.2, 50.5, 46.1, 34.9, 32.4, 28.0, 27.7, 22.5

Synthesis of N²-((benzyloxy)carbonyl)-N⁶-(2,4-dimethoxybenzyl)-L-lysine

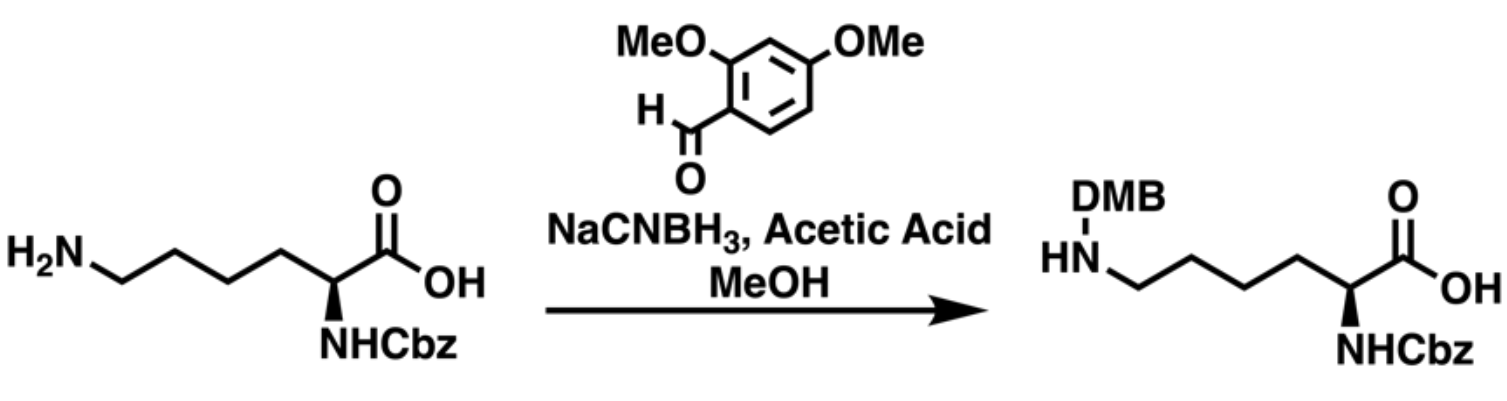

S5

Z-Lys-OH (4.2 g,15 mmol,1 eq.) was added in methanol (100 mL), to the suspension add 2,4-dimethoxylbenzaldehyde (3.05 g,18.3 mmol,1.3 eq), followed by acetic acid (945 μL, 16.5 mmol, 1.1 eq.) and $NaCNBH_3$. The solution was heated to 40 °C and was stirred under the same temperature overnight. The mixture was filtered and the solid was dried. The solid was used without further purification.

Synthesis of the 2,5-dioxopyrrolidin-1-yl carbonates

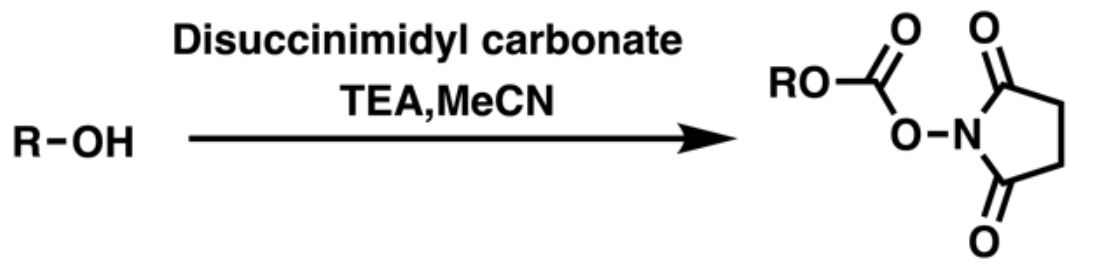

General Methond 1: the appropriate alcohol (1 mmol,1 eq.) was dissolved in acetonitrile, followed by adding disuccinimidyl carbonate (512 mg, 2 mmol, 2 eq.) and TEA (303 mg, 3 mmol, 3 eq.). The resulting solution was stirred at room temperature overnight. The solvent was removed *in vacuo,* and the resulting residue was purified by flash column chromatography with hexane and ethyl acetate.

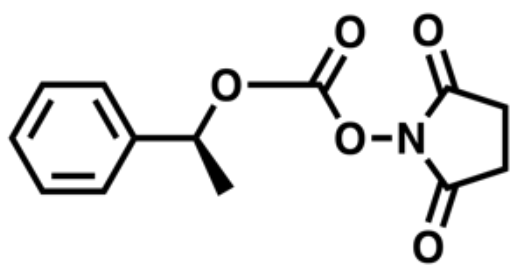

(S)-2,5-dioxopyrrolidin-1-yl (1-phenylethyl) carbonate (S6). Following general method 1 afforded **S6** as a colorless oil. 172 mg Yield: 65%

^{1}H NMR (400 MHz, $CDCl_3$): δ7.48-7.33(m,5H) 5.89-5.79(q,1H) 2.85-2.79(s,4H) 1.75-1.69(t,3H)

^{13}C NMR (100 MHz, $CDCl_3$): δ168.6, 150.9, 139.0, 128.8, 128.7, 126.0, 80.8, 25.4, 22.0,

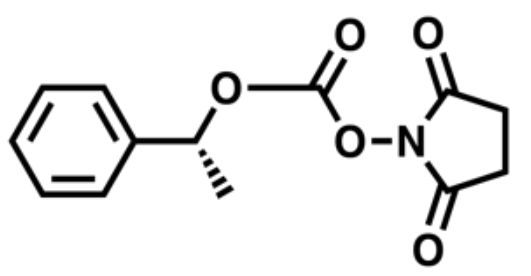

(R)-2,5-dioxopyrrolidin-1-yl (1-phenylethyl) carbonate(S7). Following general method 1 afforded **S7** as a colorless oil.193 mg yield: 73%

^{1}H NMR (400 MHz, $CDCl_3$): δ7.48-7.33(m,5H) 5.89-5.79(q,1H) 2.85-2.79(s,4H) 1.75-1.69(t,3H)

^{13}C NMR (100 MHz, $CDCl_3$): δ168.6, 150.9, 139.0, 128.8, 128.7, 126.0, 80.8, 25.4, 22.0,

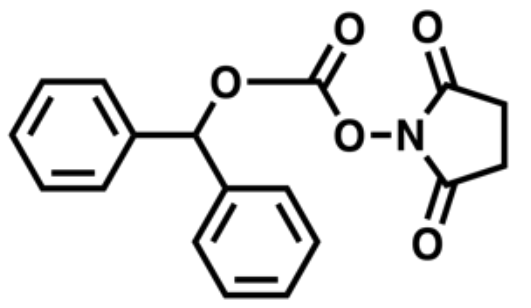

benzhydryl (2,5-dioxopyrrolidin-1-yl) carbonate (S8). Following general method 1 afforded **S8** as a colorless oil. 227 mg yield: 70%

^{1}H NMR (400 MHz, CDCl3) δ 7.59 – 7.48 (m, 2H), 7.44 – 7.31 (m, 8H), 6.99 – 6.29 (d, J = 90.0 Hz, 1H), 3.13 – 2.35 (d, J = 114.0 Hz, 4H).

^{13}C NMR (100 MHz, CDCl3) δ 171.2, 168.5, 151.1, 137.9, 137.5, 128.8, 128.7, 128.7, 128.3, 128.2, 127.0, 126.5, 88.1, 84.5, 25.4, 25.3, 14.2.

Synthesis of the diethoxyphosphorylacetyl-Lysine analogs

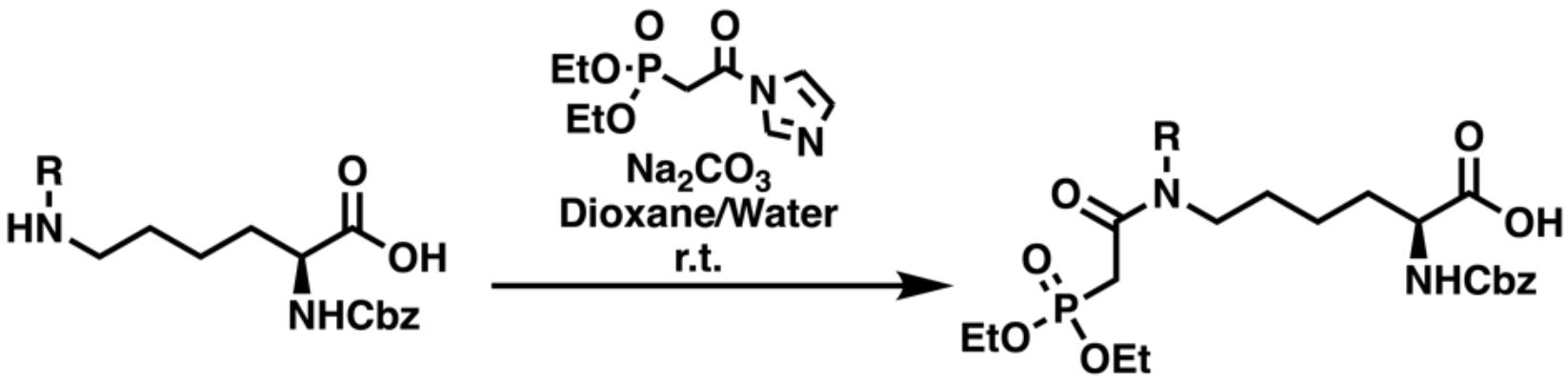

S4 or S5

Na_2CO_3(1.06 g, 10 mmol, 1 eq.) was dissolved in 50ml water, followed by adding corresponding Z-lysine or lysine analog (**S4** or **S5**,10 mmol,1 eq.) and 50mL of 1,4-dioxanes. Diethyl (2-(1H-imidazol-1-yl)-2-oxoethyl)phosphonate (4.84 g,20 mmol, 2eq.) was dissolved in 50ml 1,4-dioxane and the solution was added to the reaction mixture dropwise. The resulting solution was stirred for 15 h at room temperature. Organic impurities were removed by washing with ethyl acetate (2 x 50mL). The aqueous phase was acidified to pH 2 with 1N HCl and extracted with diethyl ether (4 x 30 mL). The combined organic extracts were dried and concentrated to afford a colorless foam. This was used in the next step without further purification.

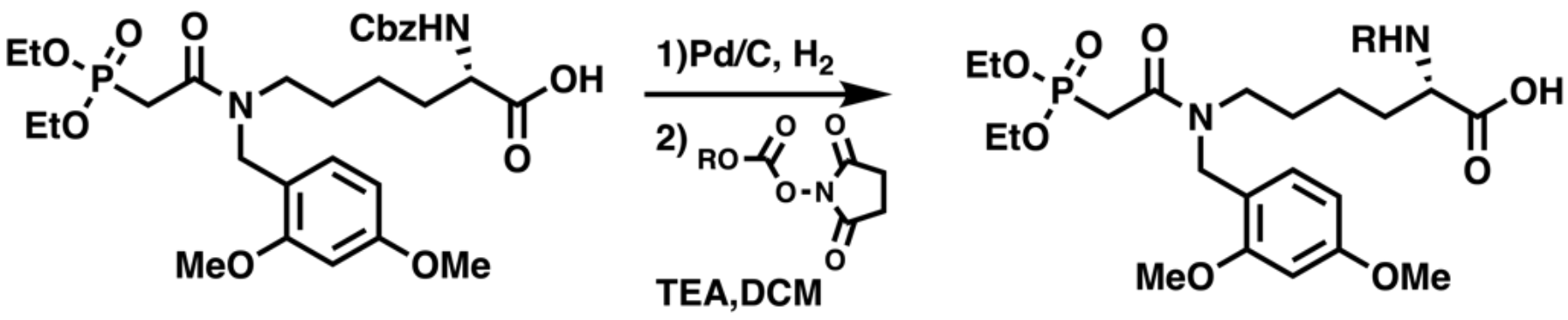

S9

S9 (608mg, 1mmol, 1eq) was dissolved in methanol,followed by adding 200mg Pd/C, and bubbling H_2 gas into the solution for 3h. The solution was filtered through a Celite® pad, and the solvent was removed *in vacuo*. The resulting oil was added in DCM followed by appropriate NHS-ester or Boc anhydride (2 mmol, 2eq.), and TEA (303 mg ,3eq.). The solution was stirred at room temperature overnight. The solvent was removed *in vacuo, and 100* mL of ethyl acetate was added. The organic phase was washed with 1M HCl (3x 100 mL), and brine(100 mL). The organic phase was dried (Na_2SO_4), filtered, and concentrated to afford a colorless foam. This was used in the next step without further purification.

Synthesis of the macrocyclization substrates

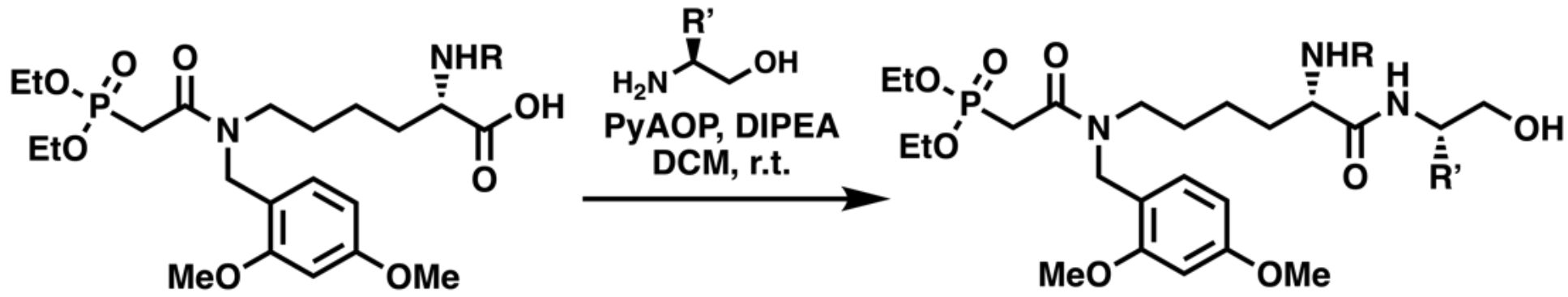

General Method 2: The appropriate L-lysine analogs (2 mmol, 1 eq.) were dissolved in DCM, PyAOP(1.25 g,2.4 mmol,1.2 eq.), and DIPEA(977μL, 4.8 mmol, 2.4 eq.) were added to the solution, followed by the corresponding amino alcohol (2.4 mmol,1.2 eq.). The reaction mixture was stirred overnight. The solvent was removed *in vacuo* and 150mL ethyl acetate was added. The organic phase was washed with 1M HCl(3 x 100mL), sat. $NaHCO_3$ (3 x 100 mL) and brine(100 mL). The organic phase was dried(Na_2SO_4), filtered, and concentrated. The resulting residue was purified by flash column chromatography with DCM and acetone.

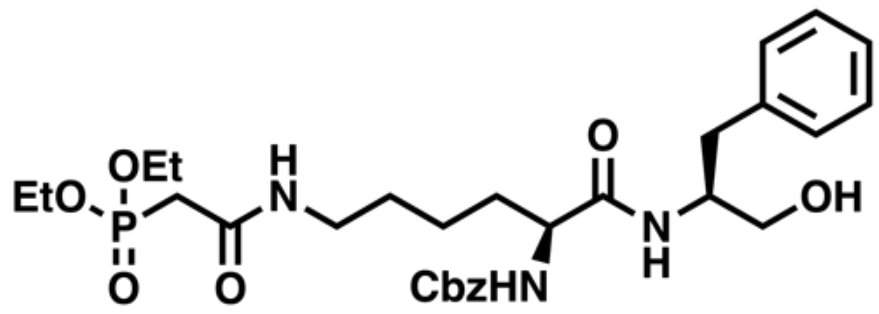

Benzyl ((S)-6-(2-(diethoxyphosphoryl)acetamido)-1-(((S)-1-hydroxy-3-phenylpropan-2-yl)amino)-1-oxohexan-2-yl)carbamate (S10)

Following general method 2 afforded **S10** as white foam,970 mg yield:82%

NMR ^{1}H (400 MHz, $CDCl_3$): δ 7.38- 7.29 (m, 5H), 7.24- 7.16 (m, 6H), 6.11- 6.09 (d, 1H), 5.10- 5.04 (s, 2H), 4.18- 4.13 (m, 2H), 4.12- 4.05 (q, 4H), 3.64- 3.62 (m, 1H), 3.53- 3.49 (m, 1H), 3.21- 3.19 (m, 2H), 2.89- 2.82 (m, 4H), 1.76- 1.72 (m, 1H), 1.67- 1.62 (m, 1H), 1.47- 1.46 (m, 2H), 1.36- 1.34 (m, 1H), 1.31- 1.28 (t, 6H);

^{13}C (100 MHz, $CDCl_3$): δ 172.0, 164.4, 156.2, 138.0, 136.3, 129.3, 128.5, 128.4, 128.1, 127.9, 126.3, 66.8, 62.9, 62.9, 62.8, 55.0, 52.9, 39.1, 37.0, 35.7, 34.47, 32.26, 28.5, 22.2, 16.3, 16.2;

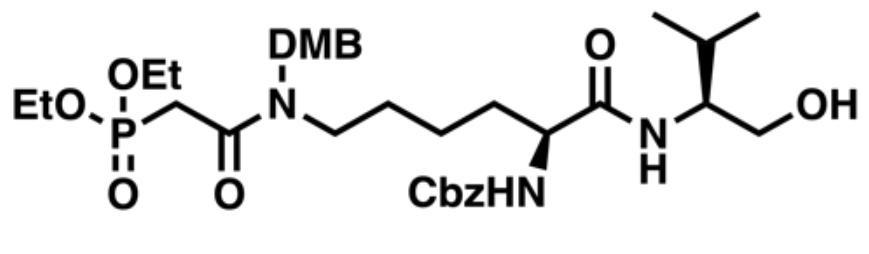

Benzyl ((S)-6-(2-(diethoxyphosphoryl)-N-(3,4-dimethylbenzyl)acetamido)-1-(((S)-1-hydroxy-3-methylbutan-2-yl)amino)-1-oxohexan-2-yl)carbamate (S11)

Following general method 2 afforded **S11** as white foam,1.17 g yield:89%

^{1}H NMR (400 MHz, $CDCl_3$) Rotamers δ 7.46 – 7.31 (m, 5H), 6.95 (d, *J* = 8.1 Hz, 1H), 6.52 – 6.40 (m, 2H), 5.83 (d, *J* = 8.0 Hz, 1H), 5.10 (s, 2H), 4.69 – 4.51 (m, 1H), 4.34 – 4.23 (m, 3H), 4.21 (s, 1H), 4.21 – 4.10 (m, 3H), 3.84 – 3.77 (m, 6H), 3.71 (d, *J* = 4.9 Hz, 2H), 3.33 (dd, *J* = 21.0, 14.6 Hz, 1H), 3.05 – 2.92 (m, 2H), 1.93 (tq, *J* = 13.8, 6.2 Hz, 2H), 1.69 (d, *J* = 7.3 Hz, 1H), 1.62 – 1.37 (m, 6H), 0.96 (dd, *J* = 12.3, 6.7 Hz, 6H).

^{13}C NMR (100 MHz, $CDCl_3$) Rotamers δ 172.1, 165.8, 160.8, 158.3, 136.5, 128.6, 128.4, 128.0, 127.9, 116.1, 103.9, 98.7, 66.6, 62.9, 62.8, 62.7, 57.1, 55.4, 55.2, 46.5, 42.7, 33.1, 29.0, 26.0, 22.4, 19.5, 19.3, 16.4, 16.3.

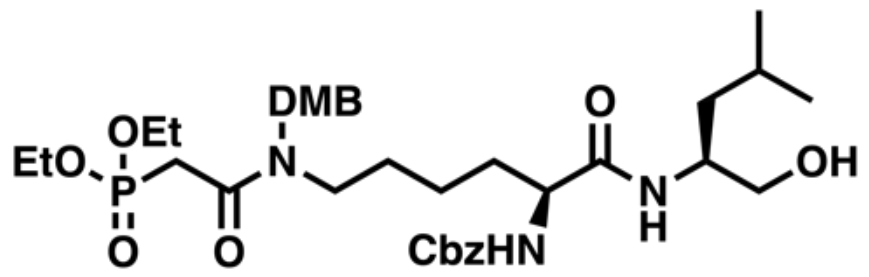

benzyl ((S)-6-(2-(diethoxyphosphoryl)-N-(3,4-dimethylbenzyl)acetamido)-1-(((S)-1-hydroxy-4-methylpentan-2-yl)amino)-1-oxohexan-2-yl)carbamate (S12)

Following general method 2 afforded **S12** as white foam. 1.17 g yield:87%

^{1}H NMR ($CDCl_3$, 400 MHz) Rotamers δ 7.36 (5H, s), 7.31 (1H, dt, *J* = 5.0, 3.0 Hz), 6.95 (1H, d, *J* = 8.0 Hz), 6.50 – 6.40 (2H, m), 5.81 (1H, d, *J* = 7.9 Hz), 5.11 (2H, s), 4.70 – 4.52 (1H, m), 4.32 (1H, s), 4.29 (1H, d, *J* = 7.4 Hz), 4.18 (6H, dqd, *J* = 8.4, 7.1, 1.4 Hz), 3.88 (1H, d, *J* = 13.1 Hz), 3.83 – 3.80 (6H, m), 3.76 (1H, d, *J* = 14.3 Hz), 3.54 (1H, ddd, *J* = 11.7, 7.2, 4.6 Hz), 3.34 (1H, dd, *J* = 21.1, 14.5 Hz), 3.06 – 2.92 (2H, m), 1.70 – 1.58 (3H, m), 1.51 (4H, ddd, *J* = 14.8, 11.3, 6.9 Hz), 1.40 (6H, dd, *J* = 14.1, 6.9 Hz), 0.93 (6H, d, *J* = 6.5 Hz)

^{13}C NMR (100 MHz, $CDCl_3$) Rotamers δ 171.8, 165.7, 160.8, 158.3, 136.5, 128.6, 128.4, 128.0, 127.9, 116.1, 103.9, 98.7, 66.6, 64.9, 62.9, 55.4, 55.2, 49.7, 46.5, 42.6, 40.0, 26.0, 24.8, 22.9, 22.4, 22.4, 16.4.

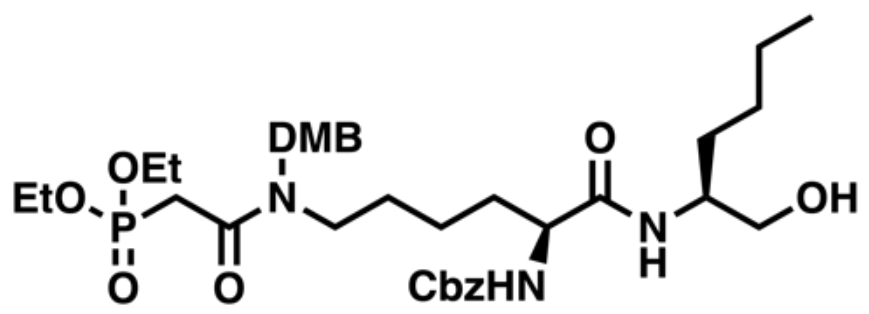

benzyl ((S)-6-(2-(diethoxyphosphoryl)-N-(3,4-dimethylbenzyl)acetamido)-1-(((S)-1-hydroxyhexan-2-yl)amino)-1-oxohexan-2-yl)carbamate (S13)

Following general method 2 afforded **S13** as white foam, 1.21 g yield:90%

^{1}H NMR (400 MHz, $CDCl_3$) Rotamers δ 7.41 – 7.32 (m, 5H), 7.24 (s, 2H), 6.95 (d, *J* = 8.0 Hz, 1H), 6.50 – 6.40 (m, 2H), 5.82 (d, *J* = 7.8 Hz, 1H), 5.10 (s, 2H), 4.69 – 4.52 (m, 1H), 4.34 – 4.27 (m, 2H), 4.27 – 4.19 (m, 2H), 4.19 – 4.11 (m, 3H), 3.98 – 3.84 (m, 3H), 3.84 – 3.69 (m, 8H), 3.58 (ddd, *J* = 11.7, 6.9, 4.7 Hz, 1H), 3.33 (dd, *J* = 21.1, 14.5 Hz, 1H), 2.99 (td, *J* = 14.5, 9.3 Hz, 2H), 2.07 (s, 1H), 1.94 (s, 1H), 1.64 – 1.51 (m, 4H), 1.39 (dd, *J* = 14.6, 7.7 Hz, 7H), 0.90 (dq, *J* = 6.0, 3.5 Hz, 3H).

^{13}C NMR (100 MHz, $CDCl_3$,) Rotamers δ 171.8, 165.8, 165.7, 160.8, 158.3, 136.5, 128.6, 128.4, 128.0, 127.9, 116.1, 103.9, 98.7, 66.6, 64.4, 62.9, 62.8, 55.4, 55.2, 51.6, 46.5, 42.7, 33.3, 30.7, 28.3, 26.0, 22.7, 22.4, 16.4, 16.3, 14.0.

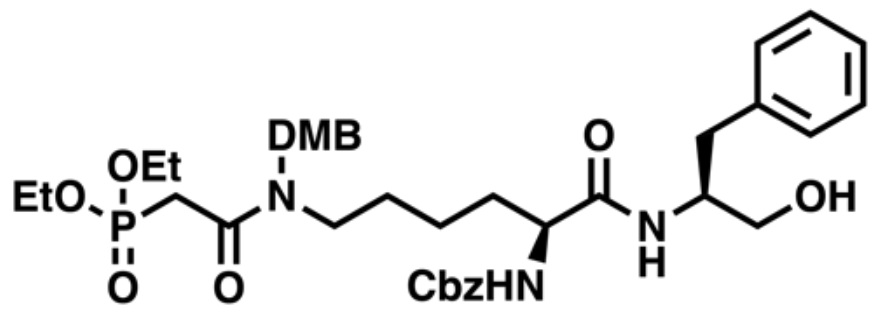

benzyl ((S)-6-(2-(diethoxyphosphoryl)-N-(3,4-dimethylbenzyl)acetamido)-1-(((S)-1-hydroxy-3-phenylpropan-2-yl)amino)-1-oxohexan-2-yl)carbamate (S14)

Following general method 2 afforded **S14** as white foam.1.2 g yield:85%

^{1}H NMR (400 MHz, $CDCl_3$) Rotamers δ 7.50 (d, *J* = 8.5 Hz, 1H), 7.40 – 7.22 (m, 11H), 7.22 – 7.14 (m, 1H), 6.95 (d, *J* = 8.0 Hz, 1H), 6.51 – 6.41 (m, 2H), 5.77 (d, *J* = 7.8 Hz, 1H), 5.11 (s, 2H), 4.66 (d, *J* = 16.8 Hz, 1H), 4.42 (s, 1H), 4.33 – 4.21 (m, 3H), 3.85 – 3.78 (m, 7H), 3.74 – 3.61 (m, 1H), 3.57 – 3.49 (m, 1H), 3.35 (dd, *J* = 21.0, 14.5 Hz, 1H), 3.07 – 2.86 (m, 5H), 2.07 (s, 2H), 1.86 (s, 2H), 1.69 (s, 1H), 1.60 – 1.50 (m, 1H), 1.48 (s, 1H), 1.45 – 1.31 (m, 8H).
^{13}C NMR (100 MHz, $CDCl_3$) Rotamers δ 171.6, 165.7, 160.8, 158.3, 138.6, 136.6, 129.4, 128.6, 128.4, 128.4, 128.3, 128.0, 127.9, 126.1, 116.1, 103.9, 98.8, 66.6, 63.0, 62.8, 60.4, 55.4, 55.2, 52.9, 46.5, 42.6, 37.0, 22.4, 21.0, 16.4, 14.2.

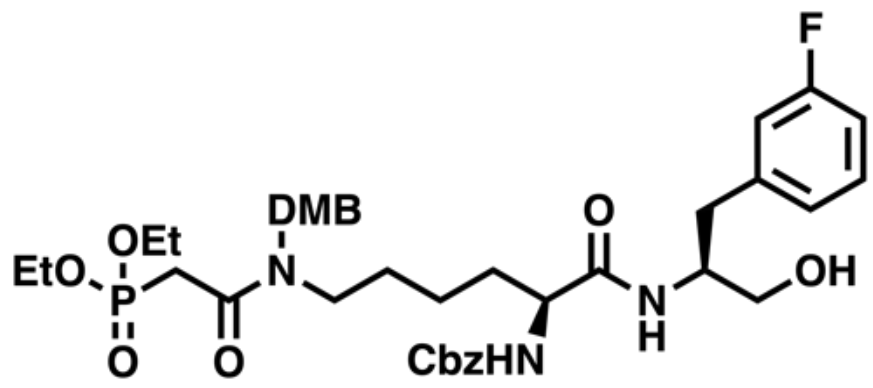

benzyl ((S)-6-(2-(diethoxyphosphoryl)-N-(3,4-dimethylbenzyl)acetamido)-1-(((S)-1-(3-fluorophenyl)-3-hydroxypropan-2-yl)amino)-1-oxohexan-2-yl)carbamate (S15)

Following general method 2 afforded **S15** as white foam. 1.25 g yield: 86%
^{1}H NMR (400 MHz, $CDCl_3$) Rotamers δ 7.59 (d, *J* = 8.5 Hz, 1H), 7.40 – 7.28 (m, 7H), 7.28 – 7.15 (m, 1H), 7.15 – 7.05 (m, 1H), 7.05 – 6.92 (m, 2H), 6.92 – 6.84 (m, 1H), 6.51 – 6.41 (m, 2H), 5.75 (d, *J* = 7.9 Hz, 1H), 5.11 (d, *J* = 2.0 Hz, 2H), 4.72 – 4.56 (m, 1H), 4.31 (s, 1H), 4.29 – 4.20 (m, 2H), 4.20 – 4.12 (m, 3H), 4.03 – 3.93 (m, 1H), 3.85 – 3.78 (m, 6H), 3.76 – 3.61 (m, 1H), 3.53 (d, *J* = 11.4 Hz, 1H), 3.37 (dd, *J* = 21.0, 14.6 Hz, 1H), 3.10 – 2.84 (m, 4H), 2.20 (s, 1H), 1.94 (d, *J* = 11.5 Hz, 1H), 1.51 – 1.30 (m, 9H), 1.28 (s, 1H).
^{13}C NMR (100 MHz, $CDCl_3$) Rotamers δ 171.6, 165.8, 165.7, 160.8, 158.3, 141.3, 141.2, 136.6, 129.7, 129.6, 128.6, 128.4, 128.0, 127.9, 125.0, 116.4, 116.2, 116.0, 113.1, 112.9, 103.9, 98.8, 77.2, 66.6, 63.0, 62.8, 55.4, 55.2, 55.1, 52.7, 46.4, 42.4, 36.7, 33.3, 29.2, 25.8, 22.4, 16.4, 16.3.

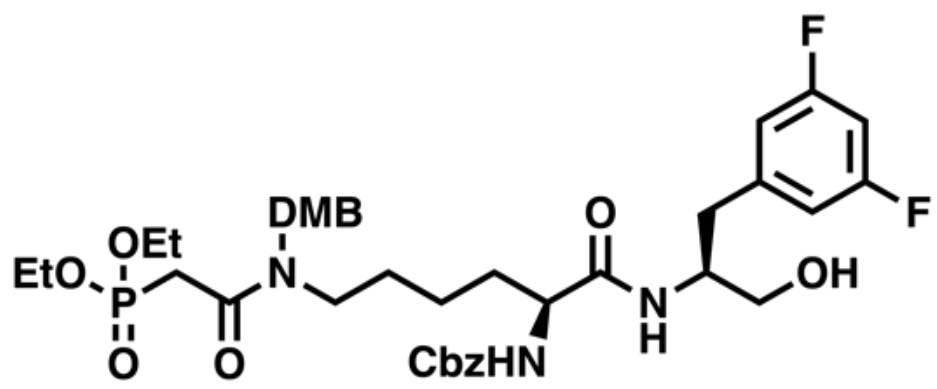

benzyl ((S)-6-(2-(diethoxyphosphoryl)-N-(3,4-dimethylbenzyl)acetamido)-1-(((S)-1-(3,5-difluorophenyl)-3-hydroxypropan-2-yl)amino)-1-oxohexan-2-yl)carbamate (S16)

Following general method 2, afforded **S16** as white foam. 1.31 g yield: 88%
^{1}H NMR (400 MHz, $CDCl_3$) Rotamers δ 7.69 (d, *J* = 8.5 Hz, 1H), 7.39 – 7.27 (m, 5H), 6.94 (d, *J* = 8.1 Hz, 1H), 6.89 – 6.76 (m, 2H), 6.63 (tt, *J* = 9.1, 2.3 Hz, 1H), 6.50 – 6.40 (m, 2H), 5.79 (d, *J* = 8.0 Hz, 1H), 5.10 (s, 2H), 4.65 (d, *J* = 16.8 Hz, 1H), 4.61 (s, 1H), 4.30 (s, 1H), 4.28 – 4.11 (m, 6H), 3.93 (d, *J* = 13.5 Hz, 1H), 3.84 – 3.77 (m, 6H), 3.71 (d, *J* = 12.0 Hz, 1H), 3.57 – 3.49 (m, 1H), 3.37 (dd, *J* = 20.9, 14.6 Hz, 1H), 3.06 – 2.82 (m, 4H), 2.19 (d, *J* = 4.9 Hz, 2H), 2.00 – 1.84 (m, 1H), 1.69 (s, 1H), 1.60 –

1.45 (m, 2H), 1.39 (dt, J = 23.3, 7.1 Hz, 8H).
^{13}C NMR (100 MHz, $CDCl_3$) Rotamers δ 171.8, 165.8, 165.7, 164.1, 164.0, 161.7, 161.5, 160.8, 158.3, 156.1, 142.8, 142.7, 136.5, 128.6, 128.5, 128.4, 128.0, 127.9, 116.0, 112.3, 112.2, 112.1, 112.1, 103.9, 101.9, 101.6, 101.4, 98.8, 66.6, 63.0, 63.0, 62.8, 62.7, 55.4, 55.3, 55.2, 55.1, 52.5, 46.4, 42.5, 36.7, 34.3, 33.1, 25.8, 22.4, 16.4, 16.3, 16.2.

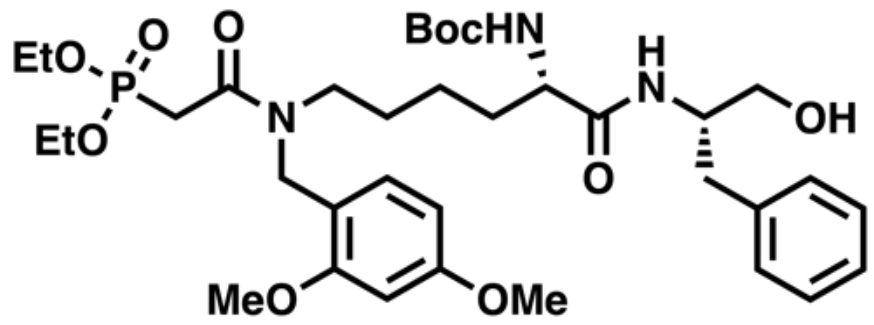

***tert*-butyl ((S)-6-(2-(diethoxyphosphoryl)-N-(2,4-dimethoxybenzyl)acetamido)-1-(((S)-1-hydroxy-3-phenylpropan-2-yl)amino)-1-oxohexan-2-yl)carbamate (S17)**

Following general method 2, afforded **S17** as a white foam. 594 mg yield: 84% over two steps

^{1}H NMR (400 MHz, $CDCl_3$): Rotamers δ7.32-7.23(5H,m), 7.23-7.13(1H,m), 7.01-6.90(1H, d), 6.50-6.40(2H,d), 5.49-5.22(1H,d) 4.67-4.46(1H,m), 4.40-4.22(3H,m), 4.23-4.13(4H,m), 4.11-4.00(1H,q), 3.81-3.77(6H,m), 3.71-3.58(1H,m), 3.80-3.46(1H,m), 3,44-3,14(1H,m), 3.12-2.96(2H,m) 2.95-2.84(2H,q), 1.94-1.81(1H,d), 1.61-1.47(1H,m), 1.45-1.41(9H,s), 1.41-1.29(8H,m)

^{13}C NMR (100 MHz, $CDCl_3$): Rotamers δ172.1, 165.7, 165.6, 160.8, 160.2,158.3, 158.3, 155.5, 138.5, 138.1, 130.0, 129.4, 129.3, 128.5, 128.4, 128.3, 126.3, 126.1, 117.5, 116.2, 104.2, 103.9, 98.7, 98.2, 79.2, 62.8, 62.8, 62.6, 60.3, 55.4, 55.3, 55.3, 55.2, 54.7, 52.9, 47.8, 46.7, 43.2, 42.7, 37.0, 36.9, 34.3, 33.0, 33.0, 28.3, 28.3, 26.1, 22.4, 21.04, 16.43, 16.3

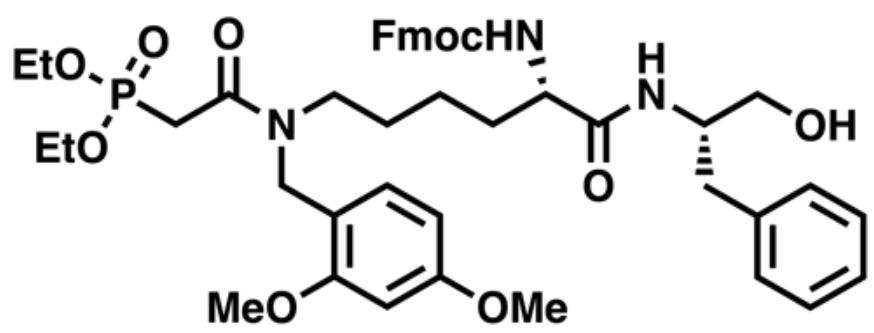

(9H-fluoren-9-yl)methyl((S)-6-(2-(diethoxyphosphoryl)-N-(2,4-dimethoxybenzyl)acetamido)-1-(((S)-1-hydroxy-3-phenylpropan-2-yl)amino)-1-oxohexan-2-yl)carbamate (S18)

Following general method 2 afforded **S18** as a white foam. 539 mg yield: 65% over two steps

^{1}H NMR (400 MHz, $CDCl_3$): Rotamers 7.95-7.71(2H,d), 7.71-7.56(3H,m), 7.46-7.38(2H,q), 7.37-7.31(2H,m), 7.31-7.28(2H,s), 7.29-7.28(2H,s), 7.22-7.14(1H,m), 7.00-6.90(1H,d), 6.56-6.43(2H,m), 5.88-5.73(1H,d), 4.78-4.47(2H,m), 4.43-4.29(3H,m), 4.29-4.17(6H,m), 4.06-3.95(1H,q), 3.87-3.79(6H,m), 3.79-3.63(1H,m), 3.60-3.46(1H,d), 3.46-3.23(1H,m), 3.06-2.84(4H,m), 1.81-1.55(5H,m), 1.55-1.46(2H,m), 1.46-1.32(7H,m)

^{13}C NMR (100 MHz, $CDCl_3$): Rotamers δ171.6, 141.2, 129.4, 128.3, 127.6, 127.0,

125.2, 119.9, 98.8, 60.4, 55.4, 55.2, 53.0, 47.1, 14.2

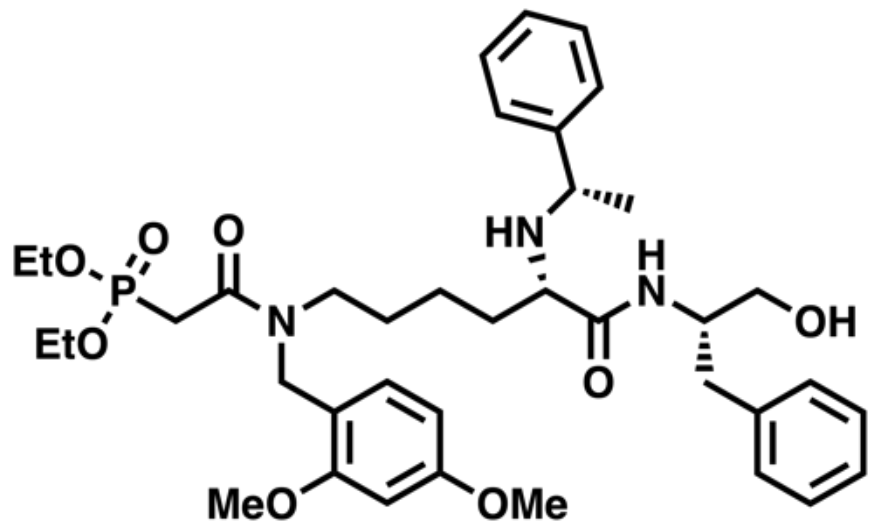

Diethyl(2-((2,4-dimethoxybenzyl)((S)-6-(((S)-1-hydroxy-3-phenylpropan-2-yl)amino)-6-oxo-5-(((S)-1-phenylethyl)amino)hexyl)amino)-2-oxoethyl)phosphonate (S19)

Following general method 2 afforded **S19** as a white foam. 548 mg yield: 74% over two steps.

^{1}H NMR (400 MHz, $CDCl_3$) Rotamers δ 7.54 – 7.47 (d, J = 8.5 Hz, 1H), 7.41 – 7.28 (m, 10H), 7.28 – 7.13 (m, 2H), 6.97 – 6.91 (d, J = 8.0 Hz, 1H), 6.50 – 6.40 (m, 2H), 5.83 – 5.74 (q, J = 6.6 Hz, 1H), 5.70 – 5.63 (d, J = 8.0 Hz, 1H), 4.68 – 4.60 (d, J = 16.8 Hz, 1H), 4.36 – 4.09 (m, 6H), 3.84 – 3.76 (m, 7H), 3.75 – 3.68 (d, J = 11.7 Hz, 1H), 3.58 – 3.49 (d, J = 10.9 Hz, 1H), 3.42 – 3.21 (m, 2H), 3.08 – 2.89 (m, 4H), 1.94 – 1.89 (m, 1H), 1.57 – 1.50 (d, J = 6.5 Hz, 4H), 1.44 – 1.31 (dq, J = 14.4, 7.2 Hz, 9H).

^{13}C NMR (100 MHz, $CDCl_3$) Rotamers δ 129.4, 128.6, 128.4, 128.3, 127.6, 126.1, 125.9, 116.1, 103.9, 98.7, 77.2, 60.4, 55.4, 55.2, 52.9, 22.7, 22.3, 21.0, 16.3, 14.2.

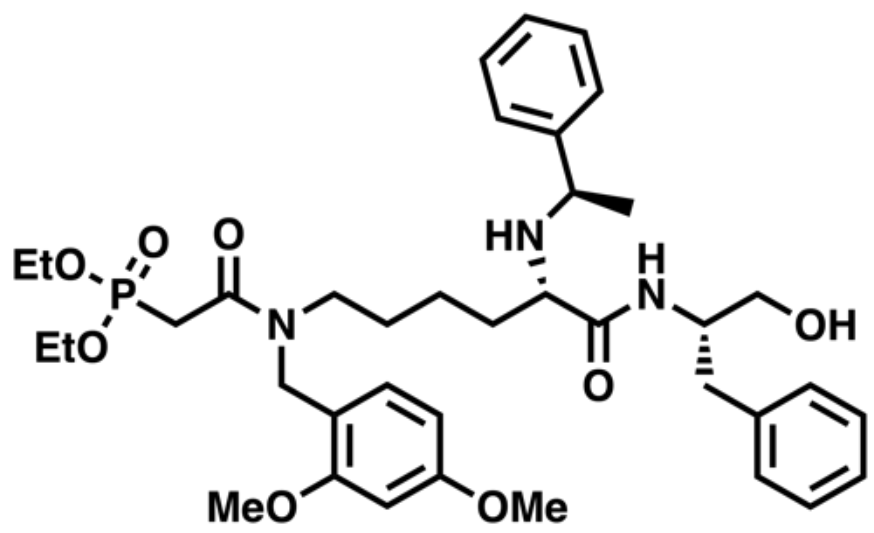

Diethyl(2-((2,4-dimethoxybenzyl)((S)-6-(((R)-1-hydroxy-3-phenylpropan-2-yl)amino)-6-oxo-5-(((S)-1-phenylethyl)amino)hexyl)amino)-2-oxoethyl)phosphonate (S20)

Following general method 2 afforded **S20** as a white foam. 578 mg yield: 78% over two steps.

^{1}H NMR (400 MHz, $CDCl_3$) Rotamers δ 7.42 – 7.31 (q, J = 3.2 Hz, 5H), 7.31 – 7.28 (d, J = 5.0 Hz, 1H), 7.28 – 7.19 (m, 4H), 7.19 – 7.11 (m, 1H), 6.99 – 6.92 (d, J = 7.9 Hz, 1H), 6.51 – 6.41 (m, 2H), 5.82 – 5.73 (q, J = 6.5 Hz, 2H), 4.68 – 4.59 (d, J = 16.8 Hz, 1H), 4.38 – 4.07 (m, 6H), 3.88 – 3.78 (m, 7H), 3.70 – 3.62 (m, 1H), 3.52 – 3.43 (dd, J = 11.7, 4.5 Hz, 1H), 3.41 – 3.28 (dd, J = 21.0, 14.6 Hz, 1H), 3.11 – 2.95 (m, 2H), 2.87 – 2.78 (m, 2H), 2.65 – 2.60 (s, 1H), 1.98 – 1.85 (s, 1H), 1.61 – 1.51 (dd, J = 6.6, 2.7 Hz, 4H), 1.48 – 1.28 (m, 9H).

^{13}C NMR (100 MHz, $CDCl_3$) Rotamers δ 172.1, 160.8, 158.3, 138.5, 129.4, 128.6, 128.4, 128.3, 127.6, 126.1, 125.9, 116.1, 103.9, 98.8, 72.7, 63.0, 60.4, 55.4, 55.2, 55.1, 52.9, 46.6, 42.9, 37.0, 26.0, 25.4, 22.6, 22.4, 21.0, 16.4, 16.3, 14.2.

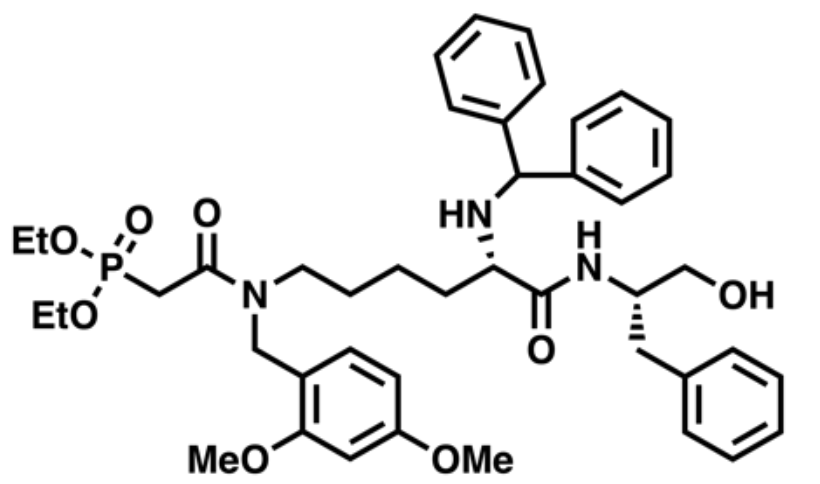

Diethyl (2-(((S)-5-(benzhydrylamino)-6-(((S)-1-hydroxy-3-phenylpropan-2-yl)amino)-6-oxohexyl)(2,4-dimethoxybenzyl)amino)-2-oxoethyl)phosphonate (S21)

Following general method 2, afforded **S21** as a white foam. 630 mg yield: 77% over two steps.

^{1}H NMR (400 MHz, $CDCl_3$) Rotamers δ 7.51 – 7.44 (d, J = 8.4 Hz, 1H), 7.41 – 7.36 (d, J = 1.8 Hz, 2H), 7.36 – 7.29 (m, 9H), 7.29 – 7.25 (dd, J = 6.4, 2.3 Hz, 5H), 7.25 – 7.18 (m, 3H), 7.18 – 7.08 (m, 2H), 6.98 – 6.91 (d, J = 8.1 Hz, 1H), 6.81 – 6.76 (s, 1H), 6.50 – 6.46 (t, J = 2.1 Hz, 2H), 6.46 – 6.41 (m, 1H), 6.03 – 5.97 (d, J = 7.8 Hz, 1H), 4.67 – 4.58 (d, J = 16.9 Hz, 1H), 4.43 – 4.15 (m, 8H), 3.90 – 3.83 (t, J = 6.7 Hz, 1H), 3.83 – 3.75 (m, 8H), 3.71 – 3.63 (d, J = 11.8 Hz, 1H), 3.54 – 3.46 (d, J = 11.5 Hz, 1H), 3.40 – 3.27 (dd, J = 21.0, 14.6 Hz, 1H), 3.11 – 2.92 (m, 2H), 2.91 – 2.82 (dd, J = 10.6, 7.3 Hz, 3H), 2.08 – 2.03 (s, 1H), 1.93 – 1.89 (d, J = 6.1 Hz, 1H), 1.67 – 1.63 (s, 1H), 1.62 – 1.57 (s, 1H), 1.49 – 1.41 (m, 2H), 1.41 – 1.29 (m, 9H).

^{13}C NMR (100 MHz, $CDCl_3$) Rotamers δ 171.6, 165.8, 165.7, 160.8, 158.3, 155.3, 140.8, 138.6, 129.4, 129.3, 128.6, 128.4, 128.4, 128.3, 128.3, 127.8, 127.7, 127.7, 127.0, 126.3, 126.1, 116.1, 104.2, 104.0, 98.7, 98.2, 62.9, 62.9, 62.8, 60.4, 55.4, 55.3, 55.2, 52.9, 46.6, 42.9, 37.0, 33.2, 26.0, 22.4, 21.0, 16.4, 16.3, 14.2.

Synthesis of the Syringolin-B Macrolactam

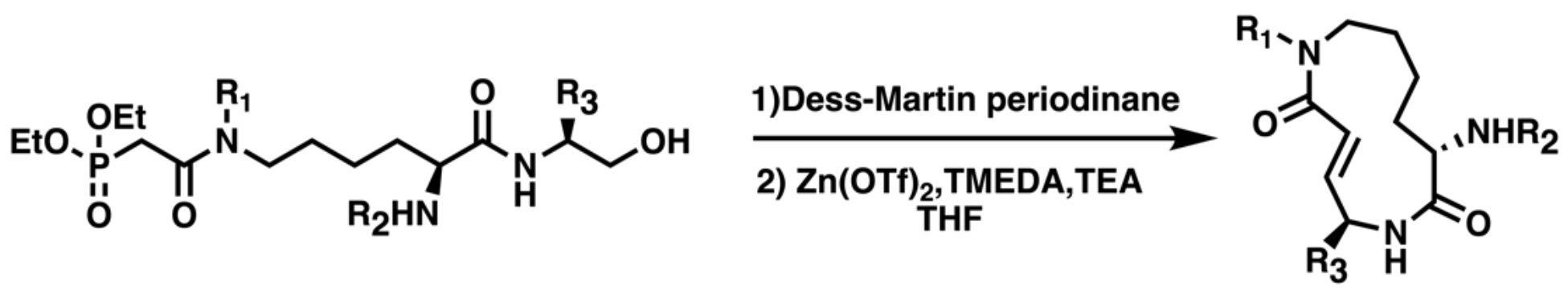

General method 3: The appropriate HWE substrate (0.5 mmol) was added in 10ml DCM, followed by Dess-Martin periodinane (254.4 mg, 0.6 mmol, 1.2 eq.). The suspension was stirred at room temperature for 90 minutes. The solution was diluted in 100ml ethyl acetate, the resulting solution was washed with a 1:1 solution of 5% sodium thiosulfate and sat. sodium bicarbonate (1:1, 3x50 mL). Dry the organic phase with sodium sulfate. The organic phase was dried (Na_2SO_4), filtered, and concentrated. The resulting aldehyde was dissolved in 50ml THF.

$Zn(OTf)_2$ (400 mg,1.1mmol, 2.2 eq.) was added in 50mL THF, followed by TMEDA (70 mg, 0.6 mmol, 1.2 eq.) and TEA (202 mg, 2 mmol, 4 eq.). The suspension was stirred at room temperature for 15 minutes. The previous aldehyde solution was added to the reaction mixture through a dropping funnel in 2h. The reaction mixture

was stirred at room temperature overnight. The solvent was removed *in vacuo, and 100* mL of ethyl acetate was added. The organic phase was washed with 1 M HCl (3 × 100 mL) and brine (100 mL). The organic phase was dried (Na_2SO_4), filtered, and concentrated. The resulting residue was purified by flash column chromatography with DCM and ethyl acetate.

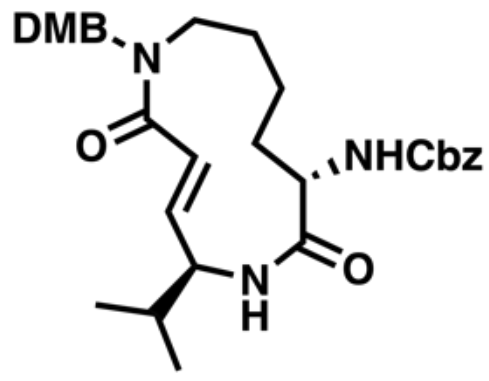

Benzyl ((5S,8S,E)-1-(2,4-dimethoxybenzyl)-5-isopropyl-2,7-dioxo-1,6-diazacyclododec-3-en-8-yl)carbamate (S22).

Following general method 3 afforded **S22** as a white solid. 247.0 mg yield: 92%

^{1}H NMR (400 MHz, $CDCl_3$) δ 7.39 – 7.25 (m, 5H), 7.19 – 7.11 (m, 1H), 7.05 – 6.96 (dd, J = 15.3, 4.8 Hz, 1H), 6.92 – 6.85 (d, J = 8.4 Hz, 1H), 6.46 – 6.35 (m, 3H), 6.06 – 5.99 (d, J = 7.4 Hz, 1H), 5.10 – 5.05 (s, 2H), 4.84 – 4.76 (d, J = 15.0 Hz, 1H), 4.68 – 4.60 (dt, J = 7.5, 3.6 Hz, 1H), 4.46 – 4.36 (q, J = 7.3 Hz, 1H), 4.34 – 4.26 (d, J = 14.9 Hz, 1H), 3.85 – 3.76 (d, J = 1.8 Hz, 6H), 3.61 – 3.50 (m, 1H), 3.17 – 3.06 (dt, J = 15.6, 3.9 Hz, 1H), 2.01 – 1.91 (dt, J = 11.5, 5.1 Hz, 2H), 1.84 – 1.75 (dd, J = 13.7, 6.9 Hz, 2H), 1.48 – 1.35 (m, 1H), 1.31 – 1.19 (dt, J = 17.1, 5.2 Hz, 2H), 1.03 – 0.95 (t, J = 6.7 Hz, 6H).

^{13}C NMR (100 MHz, $CDCl_3$) δ 171.0, 165.7, 160.1, 158.3, 155.7, 144.4, 136.2, 130.1, 128.5, 128.1, 127.7, 120.4, 118.0, 104.3, 98.3, 66.7, 56.7, 55.3, 55.3, 53.7, 44.58, 43.20, 31.6, 31.5, 27.0, 19.6, 19.3, 17.9.

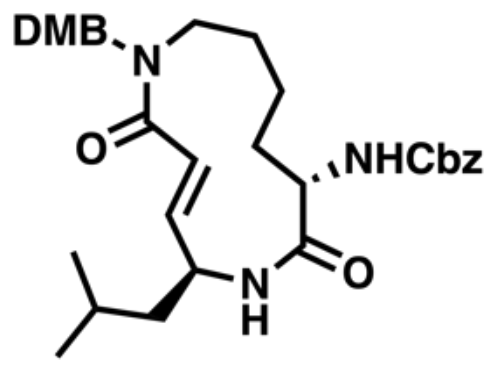

Benzyl ((5*S*,8*S*,*E*)-1-(3,4-dimethylbenzyl)-5-isobutyl-2,7-dioxo-1,6-diazacyclododec-3-en-8-yl)carbamate (S23).

Following general method 3, afforded **S23** as a white solid. 225.9 mg yield: 82%

^{1}H NMR (400 MHz, $CDCl_3$) δ 7.39 – 7.25 (m, 5H), 7.21 – 7.12 (dd, J = 9.7, 7.1 Hz, 1H), 7.08 – 6.96 (dd, J = 15.2, 4.8 Hz, 1H), 6.88 – 6.82 (d, J = 8.1 Hz, 1H), 6.50 – 6.39 (m, 3H), 6.03 – 5.97 (d, J = 7.4 Hz, 1H), 5.11 – 5.09 (s, 1H), 5.09 – 5.02 (d, J = 12.3 Hz, 1H), 4.85 – 4.77 (d, J = 15.0 Hz, 1H), 4.72 – 4.57 (m, 2H), 4.34 – 4.26 (d, J = 15.0 Hz, 1H), 3.81 – 3.76 (s, 6H), 3.60 – 3.49 (m, 1H), 3.16 – 3.06 (dt, J = 15.6, 3.9 Hz, 1H), 2.01 – 1.93 (dd, J = 8.7, 4.5 Hz, 2H), 1.77 – 1.66 (dt, J = 13.5, 6.7 Hz, 2H), 1.54 – 1.34 (m, 3H), 1.32 – 1.20 (dt, J = 12.8, 8.8 Hz, 2H), 0.96 – 0.87 (dd, J = 11.0, 6.6 Hz, 6H).

^{13}C NMR (100 MHz, $CDCl_3$) δ 170.8, 165.8, 160.1, 158.3, 155.7, 145.6, 136.1, 130.1, 128.5, 128.1, 127.8, 119.6, 118.0, 104.3, 98.3, 66.8, 55.3, 55.3, 53.7, 49.1, 44.5, 43.1, 42.7, 31.5, 27.0, 25.2, 22.7, 22.1, 17.9.

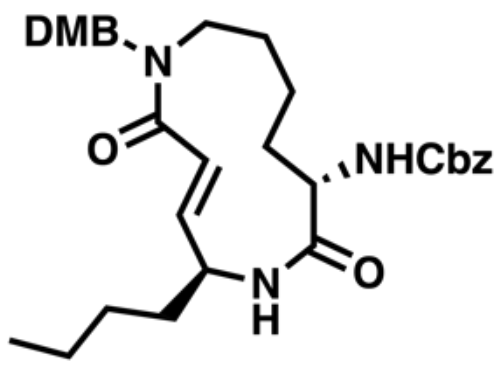

Benzyl ((5S,8S,E)-5-butyl-1-(2,4-dimethoxybenzyl)-2,7-dioxo-1,6-diazacyclododec-3-en-8-yl)carbamate (S24)

Following general method 3 afforded **S24** as a white solid. 231.4 mg yield: 84%

^{1}H NMR (400 MHz, $CDCl_3$) δ 7.39 – 7.25 (m, 5H), 7.20 – 7.11 (m, 1H), 7.06 – 6.96 (dd, J = 15.2, 4.8 Hz, 1H), 6.80 – 6.74 (d, J = 8.0 Hz, 1H), 6.47 – 6.38 (m, 3H), 6.02 – 5.95 (d, J = 7.4 Hz, 1H), 5.18 – 5.03 (d, J = 2.7 Hz, 2H), 4.85 – 4.77 (d, J = 14.9 Hz, 1H), 4.66 – 4.53 (m, 2H), 4.35 – 4.26 (d, J = 14.9 Hz, 1H), 3.85 – 3.76 (d, J = 1.0 Hz, 6H), 3.61 – 3.50 (m, 1H), 3.17 – 3.07 (dt, J = 15.5, 3.9 Hz, 1H), 2.02 – 1.90 (td, J = 11.9, 7.8 Hz, 2H), 1.72 – 1.59 (ddt, J = 13.0, 9.1, 6.1 Hz, 1H), 1.58 – 1.46 (dtd, J = 13.8, 8.8, 5.3 Hz, 1H), 1.45 – 1.20 (m, 7H), 0.93 – 0.85 (t, J = 7.1 Hz, 3H).

^{13}C NMR (100 MHz, $CDCl_3$) δ 170.8, 165.8, 160.1, 158.3, 155.6, 145.4, 136.1, 130.2, 128.5, 128.1, 127.7, 119.6, 118.1, 104.3, 98.3, 66.8, 55.3, 55.3, 53.7, 50.8, 44.5, 43.1, 33.3, 31.5, 28.4, 27.0, 22.3, 17.9, 13.8.

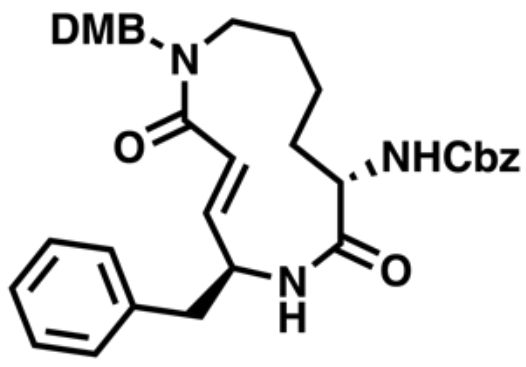

Benzyl ((5*S*,8*S*,*E*)-5-benzyl-1-(3,4-dimethylbenzyl)-2,7-dioxo-1,6-diazacyclododec-3-en-8-yl)carbamate (S25)

Following general method 3, afforded **S25** as a white solid. 243.0 mg yield: 83%

^{1}H NMR (400MHz, $CDCl_3$): δ8.53-8.35(1H,d), 7.40-7.28(8H,m), 7.30-7.20(1H,d), 7.12-7.02(1H,d), 7.00-6.86(1H,d), 6.84-6.72(1H,d),6.59-6.53(1H,d), 6.54-6.43(2H,m), 5.03-4.92(2H,s), 4.70-4.56(2H,d), 4.39-4.25(1H,s), 4.22-4.11(1H,d), 3.81-3.76(3H,s), 3.76-3.72(3H,s), 3.71-3.58(1H,m), 3.30-3.27(2H,s), 3.11-2.88(2H,m), 2.81-2.70(1H,m), 2.25-2.08(1H,m), 1.69-1.55(2H,s), 1.49-1.32(1H,m), 1.27-1.28(1H,m). 1.06-0.91(1H,m)

^{13}C NMR (100 MHz, $CDCl_3$) δ 171.6, 165.8, 160.8, 158.3, 138.6, 136.6, 129.4, 128.6, 128.4, 128.3, 128.0, 127.9, 126.1, 116.1, 103.9, 98.8, 66.6, 63.0, 62.9, 62.8, 55.4, 55.2, 52.9, 46.5, 42.6, 37.0, 33.1, 25.9, 22.4, 16.4.

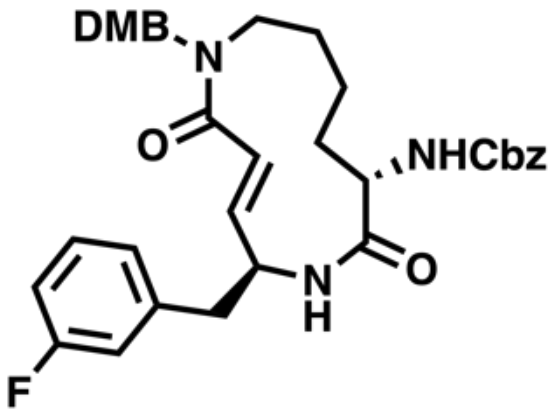

Benzyl ((5*S*,8*S*,*E*)-1-(3,4-dimethylbenzyl)-5-(3-fluorobenzyl)-2,7-dioxo-1,6-diazacyclododec-3-en-8-yl)carbamate (S26)

Following general method 3, afforded **S26** as a white solid. 235.4 mg yield: 78%

^{1}H NMR (400 MHz, $CDCl_3$) δ 7.32 (s, 5H), 7.21 (td, *J* = 7.9, 6.0 Hz, 1H), 7.16 – 7.10 (m, 1H), 7.04 – 6.83 (m, 5H), 6.51 – 6.38 (m, 3H), 5.93 (d, *J* = 7.2 Hz, 1H), 5.08 (s, 2H), 4.92 (s, 1H), 4.79 (d, *J* = 14.9 Hz, 1H), 4.50 (dt, *J* = 7.8, 3.5 Hz, 1H), 4.32 (d, *J* = 15.0 Hz, 1H), 3.77 (d, *J* = 3.3 Hz, 6H), 3.61 – 3.50 (m, 1H), 3.12 (dd, *J* = 15.6, 4.8 Hz, 1H), 2.94 (dd, *J* = 14.3, 6.0 Hz, 1H), 2.71 (dd, *J* = 14.3, 8.8 Hz, 1H), 1.94 (d, *J* = 10.5 Hz, 2H), 1.40 (t, *J* = 9.5 Hz, 1H), 1.32 – 1.16 (m, 2H).

^{13}C NMR (100 MHz, $CDCl_3$) δ 170.9, 165.6, 164.0, 161.6, 160.2, 158.3, 155.5, 144.5, 136.2, 130.2, 130.1, 129.9, 128.5, 128.1, 127.8, 124.4, 120.4, 117.8, 115.9, 115.7, 114.0, 113.8, 104.3, 98.3, 66.8, 55.3, 55.3, 53.6, 51.6, 44.6, 43.4, 38.7, 31.2, 27.0, 17.8.

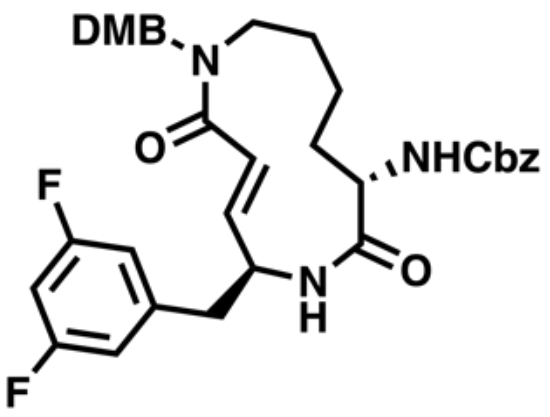

Benzyl ((5*S*,8*S*,*E*)-5-(3,5-difluorobenzyl)-1-(3,4-dimethylbenzyl)-2,7-dioxo-1,6-diazacyclododec-3-en-8-yl)carbamate (S27)

Following general method 3 afforded **S27** as a white solid. 245.6 mg yield: 79%

^{1}H NMR (400 MHz, $CDCl_3$) δ 7.32 (s, 6H), 7.15 (td, *J* = 9.0, 5.9 Hz, 2H), 6.99 (dd, *J* = 15.2, 4.6 Hz, 1H), 6.85 – 6.73 (m, 3H), 6.53 – 6.45 (m, 1H), 6.45 – 6.39 (m, 2H), 5.90 (d, *J* = 7.2 Hz, 1H), 5.08 (s, 2H), 4.79 (d, *J* = 14.9 Hz, 1H), 4.55 – 4.48 (m, 1H), 4.33 (d, *J* = 14.9 Hz, 1H), 3.78 (s, 6H), 3.56 (t, *J* = 13.8 Hz, 1H), 3.14 (dd, *J* = 15.7, 4.6 Hz, 1H), 2.93 (dd, *J* = 14.5, 5.6 Hz, 1H), 2.76 (dd, *J* = 14.5, 9.5 Hz, 1H), 2.00 – 1.89 (m, 2H), 1.41 (s, 1H), 1.25 (d, *J* = 11.3 Hz, 2H).

^{13}C NMR (100 MHz, $CDCl_3$) δ 170.9, 165.5, 160.1, 158.3, 155.5, 144.0, 136.1, 130.1, 128.5, 128.1, 127.8, 120.6, 117.9, 104.3, 103.9, 98.3, 66.8, 60.4, 55.3, 55.3, 53.7, 51.4, 44.5, 43.3, 31.7, 31.3, 26.9, 21.0, 17.8, 14.2.

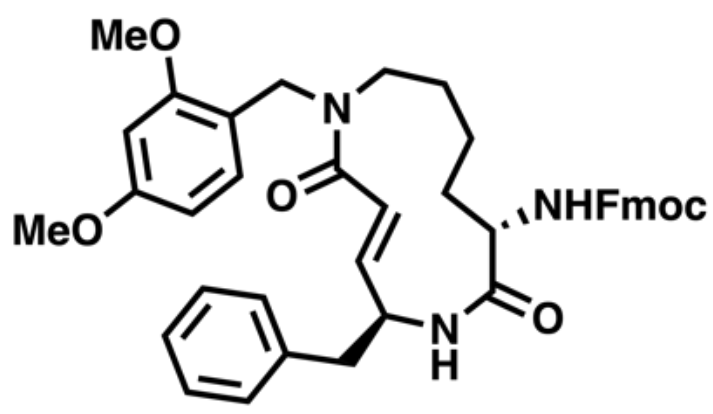

(9H-fluoren-9-yl)methyl ((5S,8S,E)-5-benzyl-1-(2,4-dimethoxybenzyl)-2,7-dioxo-1,6-diazacyclododec-3-en-8-yl)carbamate (S28)

Following general method 3 afforded **S28** as a white solid. 228.4 mg yield: 78%

^{1}H NMR (400MHz, $CDCl_3$): δ7.86-7.70(2H,d), 7.65-7.52(2H,t), 7.45-7.35(2H,t), 7.33-7.20(5H,m), 7.19-7.12(3H,q), 7.07-6.97(1H,q), 6.48-6.41(2H,m), 6.41-6.32(2H,d), 6.03-5.85(1H,d), 5.08-4.91(1H,t), 4.87-4.72(1H,s), 4.58-4.42(4H,m), 4.42-4.28(3H,m), 3.85-3.74(6,s), 3.68-3.44(1H,q), 3.23-3.07(1H,d), 3.05-2.92(1H,q), 2.88-2.68(1H,4), 2.06-1.93(1H,t), 1.90-1.66(3H,m), 1.50-1.32(1H,q), 1.25-1.16(1H,d)

^{13}C NMR (400MHz, $CDCl_3$): δ170.6, 165.5, 160.1, 158.3, 155.4, 144.4, 143.8, 143.6,

141.2, 136.2. 130.2, 128.9, 128.7, 127.7, 127.0, 125.0, 120.2, 120.0, 118.0, 104.3, 98.3, 67.0, 55.3, 55.3, 53.7, 51.5, 47.1, 44.5, 43.2, 39.0, 31.3, 17.7

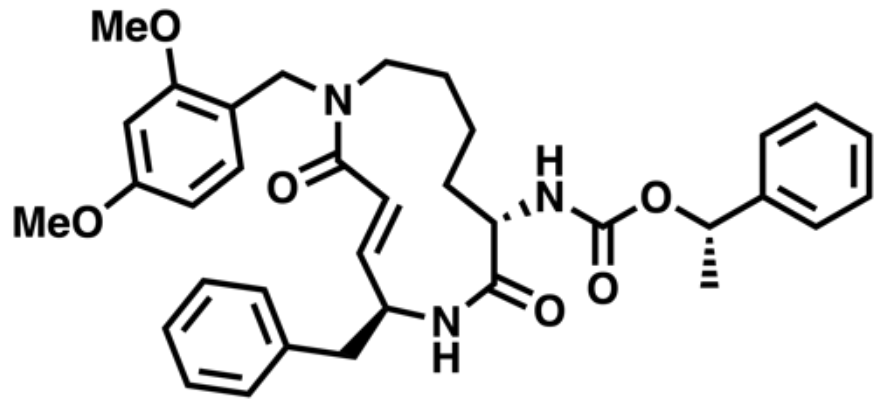

(S)-1-phenylethyl((5S,8S,E)-5-benzyl-1-(2,4-dimethoxybenzyl)-2,7-dioxo-1,6-diazacyclododec-3-en-8-yl)carbamate (S29)

Following general method 3, afforded **S29** as a white solid. 173.7 mg yield: 58%

^{1}H NMR (400MHz, $CDCl_3$): δ7.43-7.26(7H,m), 7.23-7,14(4H,m), 7.04-6.92(1H,q), 6.85-6.67(1H,d), 6.50-6.41(2H,t), 6.40-6.30(1H,d), 5.97-5.85(1H,d), 5.79-5.62(1H,q), 5.06-4.89(1H,s), 4.86-4.71(1H,d), 4.58-4.40(1H,t), 4.40-4.23(1H,d), 3.84-3.73(6H,d), 3.51-3.33(1H,t), 3.16-2.93(2H,m), 2.88-2.70(1H,q), 1.92-1.76(2H,t), 1.76-1.63(1H,t), 1.57-1.44(3H,d), 1.39-1.26(1H,m), 1.24-1.08(2H,q)

^{13}C NMR (400MHz, $CDCl_3$): δ170.8, 165.6, 160.1, 158.3, 155.2, 144.6, 142.1, 136.6, 130.4, 128.9, 128.7, 128.4, 127.7, 127.0, 125.6, 120.2, 118.1, 104.3, 98.3, 73.2, 55.3, 55.3, 53.54, 51.7, 44.4, 43.1, 39.2, 31.4, 26.9, 22.8, 17.8

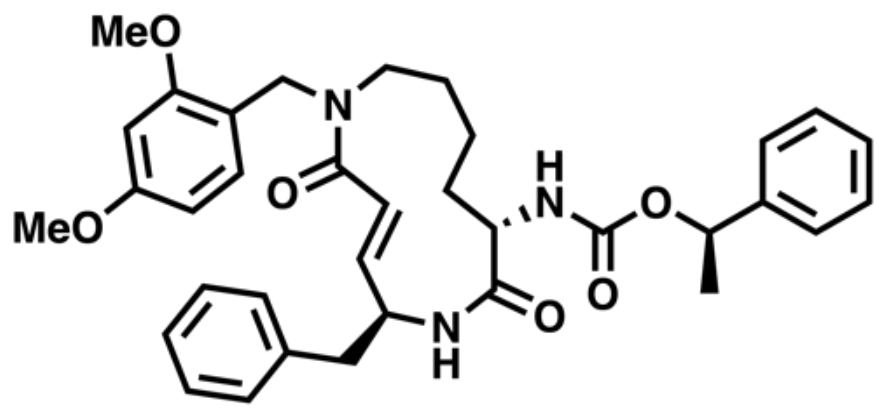

(R)-1-phenylethyl((5S,8S,E)-5-benzyl-1-(2,4-dimethoxybenzyl)-2,7-dioxo-1,6-diazacyclododec-3-en-8-yl)carbamate (S30)

Following general method 3, afforded **S30** as a white solid. 221.6 mg yield: 74%

^{1}H NMR (400MHz, $CDCl_3$): δ7.42-7.21(9H,m), 7.21-7.11(3H,m), 7.10-6.96(1H,1), 6.52-6.41(2H,q), 6.38-6.24(1H,d), 6.01-5.68(3H,m), 5.10-4.89(1H,s), 4.83-4.69(1H,d), 4.44-4.26(2H,m), 3.83-3.78(6H,d), 3.63-3.36(1H,t), 3.26-3.06(1H,t), 3.10-2.90(1H,m), 2.87-2.66(1H,q), 2.06-1.95(1H,t), 1.84-1.71(2H,q), 1.71-1.58(1H,s), 1.57-1.50(3H,d), 1.50-1.36(1H,m), 1.26-1.16(2H,m)

^{13}C NMR (400MHz, $CDCl_3$): δ170.6, 165.4, 160.1, 158.4, 144.2, 142.0, 130.3, 129.0, 128.7, 128.4, 127.7, 127.1, 125.8, 120.3, 118.1, 104.3, 98.3, 73.0, 55.3, 55.3, 53.6, 51.4, 44.5, 43.2, 38.9, 31.4, 26.9, 22.6, 17.7

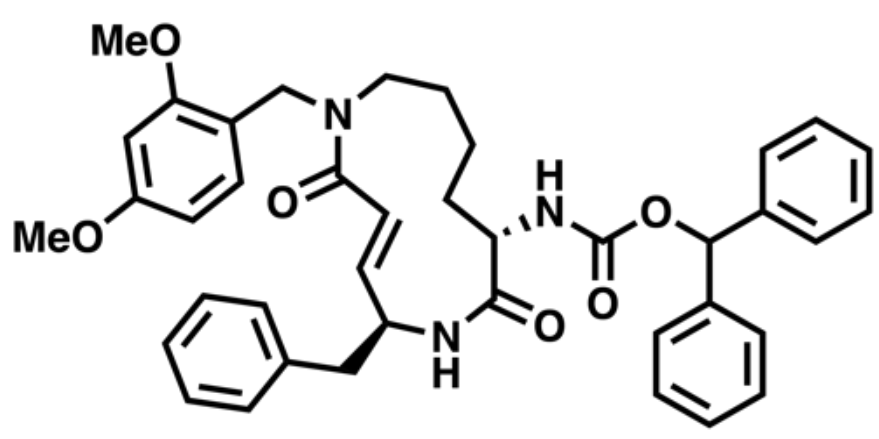

Benzhydryl((5S,8S,E)-5-benzyl-1-(2,4-dimethoxybenzyl)-2,7-dioxo-1,6-diazacyclododec-3-en-8-yl)carbamate (S31)

Following general method 3, afforded **S31** as a white solid. 211.7 mg yield: 64%

^{1}H NMR (400MHz, $CDCl_3$): δ7.42-7.11(17H,m), 7.08-6.97(1H,q), 6.76-6.68(1H,s),

6.50-6.41(2H,q), 6.40-6.28(2H,t), 6.14-5.89(1H,d), 5.02-4.89(1H,s), 4.85-4.72(1H,d), 4.49-4.38(1H,t), 4.38-4.27(1H,t), 3.83-3.75(6H,s), 3.54-3.36(1H,q), 3.20-3.04(1H,d), 3.05-2.89(1H,q), 2.86-2.61(1H,q), 2.03-1.88(1H,m), 1.88-1.64(3H,m), 1.47-1.31(1H,m), 1.26-1.07(2H,m)

^{13}C NMR (400MHz, $CDCl_3$): δ170.6, 165.5, 160.1, 158.4, 154.8, 144.4, 140.4, 140.3, 136.2, 130.3, 128.9, 128.7, 128.5, 127.8, 127.8, 127.0, 126.8, 120.2, 118.0, 104.3, 98.3, 77.8, 55.3, 55.3, 53.7, 51.6, 44.5, 43.2, 39.0, 31.2, 26.9, 17.7

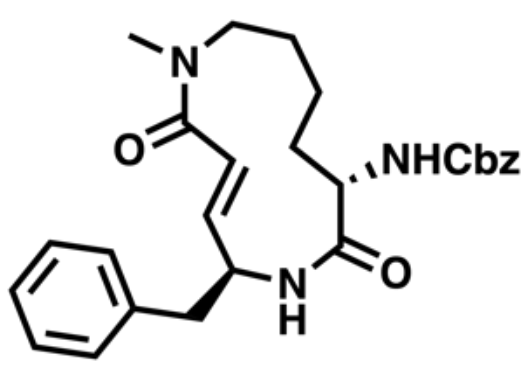

Benzyl((5S,8S,E)-5-benzyl-1-methyl-2,7-dioxo-1,6-diazacyclododec-3-en-8-yl)carbamate (S32)

Following general method 3, afforded **S32** as a white solid. 80.9 mg yield: 36%

^{1}H NMR (400MHz, $CDCl_3$): δ8.65-8.29(1H,d), 7.42-7.27(10H,m), 7.26-7.17(1H,m), 7.14-6.98(1H,d), 6.83-6.68(1H,q), 6.56-6.39(1H,d), 5.10-4.91(2H,d), 4.68-4.52(1H,s), 4.38-4.28(1H,t), 4.37-4.28(1H,t), 3.85-3.59(1H,t), 3.18-3.00(1H,t), 2.98-2.88(1H,q), 2.87-2.80(3H,s),2.80-2.68(1H,q), 2.31-2.09(1H,t), 1.80-1.56(2H,m), 1.55-1.36(1H,s), 1.30-1.20(1H,d), 0.98-0.77(1H,m)

^{13}C NMR (400MHz, $CDCl_3$): δ171.6, 164.9, 155.9,145.7, 138.8, 137.4, 129.3, 128.7, 128.2, 128.1, 126.8, 120.0, 65.8, 60.2, 55.3, 53.6, 52.2, 46.5, 40.6, 40.4, 40.2, 39.9, 39.7, 39.5, 39.3, 38.8, 33.9, 30.7, 26.7, 21.2, 17.8, 14.5

Synthesis of the D-Glu(OtBu) sidechain

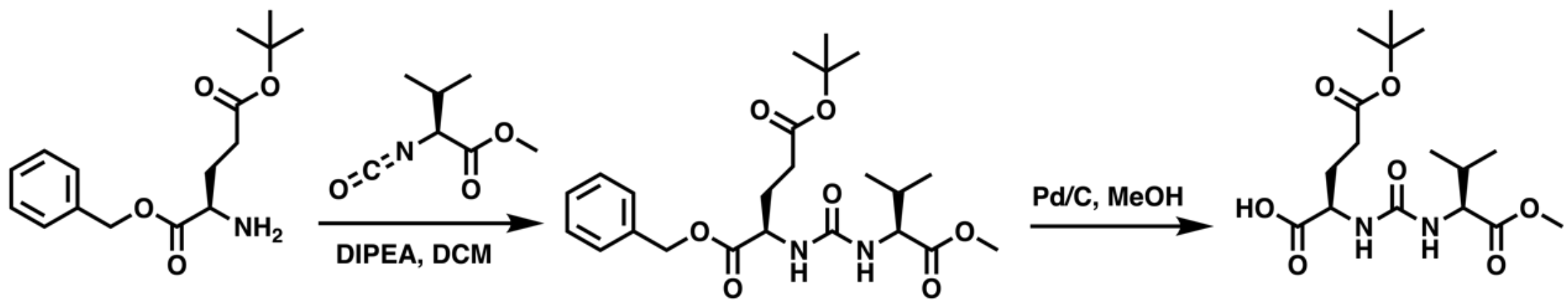

1-benzyl 5-(*tert*-butyl) was mixed with isocyanate(1 eq.) and DIPEA(2 eq.) in DCM, the solution was stirred overnight at room temperature. The solvent was evaporated and redissolved in Ethyl Acetate. The organic phase was washed with 1M HCl(3 times) and brine. The organic phase was dried with sodium sulfate, filtered and the solvent was evaporated. To a solution of the resulting product in methanol(10 mL) and 10% mmol Palladium on activated carbon 10% Pd (50% wet with water for safety). Stir the mixture with bubbling H_2 for 75 minutes. The reaction mixture was filtered through a 0.45 μm syringe filter, followed by a methanol rinse (2 mL). The solvent was removed by evaporation, and the resulting product was used without any further purification.

Synthesis of the Syringolin-Based Proteasome Inhibitor Analogs

General method 4: the appropriate macrolactam (0.1 mmol) was dissolved in

90%TFA and 10% thioanisole, the mixture was stir 4 hours at 50 °C, he solvent was removed by evaporation and diethyl ether was added to the resulting mixture. The resulting suspension was centrifuged, and the supernatant was discarded. The resulting solid was then added to a solution of HATU(38 mg,0.1 mmol, 1 eq.), the corresponding carboxylic sidechain (0.1 mmol, 1 eq.), and DIPEA (35 μL, 0.2 mmol, 2 eq.). The solution was stirred overnight at room temperature. The solvent was removed by evaporation, and the resulting solid was washed with ethyl acetate and acetone, afforded the pure syringolin analogs.

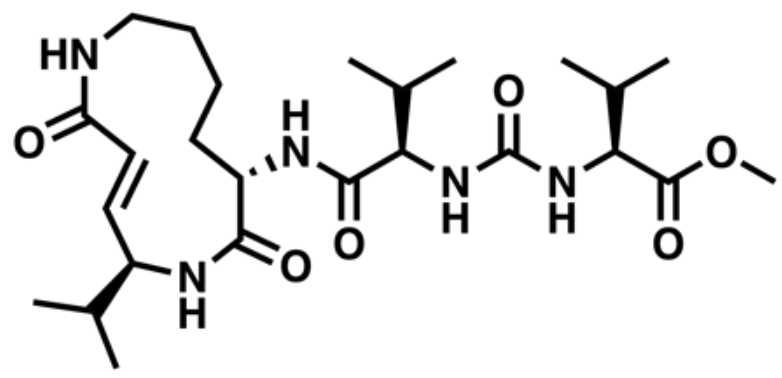

Methyl (((R)-1-(((5S,8S,E)-5-isopropyl-2,7-dioxo-1,6-diazacyclododec-3-en-8-yl)amino)-3-methyl-1-oxobutan-2-yl)carbamoyl)-L-valinate(**A-1**).

Following general method 4 afforded A-1 as white solid. 33 mg yield:65%

^{1}H NMR (600 MHz, MeOD) δ 7.07 (dd, J = 15.5, 5.0 Hz, 1H), 6.60 (dd, J = 14.8, 8.3 Hz, 1H), 6.38 (d, J = 15.5 Hz, 1H), 4.75 – 4.54 (m, 6H), 4.23 (dq, J = 18.6, 5.8 Hz, 2H), 4.09 (t, J = 5.7 Hz, 1H), 3.69 (s, 3H), 3.56 – 3.45 (m, 1H), 3.13 (dt, J = 15.3, 4.1 Hz, 1H), 2.21 (dq, J = 13.4, 6.8 Hz, 1H), 2.15 (dt, J = 13.0, 6.7 Hz, 1H), 2.12 – 2.05 (m, 1H), 1.88 (ddq, J = 33.6, 13.8, 5.9 Hz, 2H), 1.70 – 1.61 (m, 1H), 1.40 (dt, J = 18.4, 9.2 Hz, 2H), 1.07 (dd, J = 18.4, 6.7 Hz, 7H), 0.99 (dd, J = 11.3, 6.7 Hz, 6H), 0.93 (q, J = 5.9 Hz, 6H).

^{13}C NMR (151 MHz, MeOD) δ 173.1, 172.4, 171.5, 168.2, 146.7, 118.9, 59.3, 58.2, 56.9, 51.8, 51.0, 38.4, 31.9, 30.6, 30.2, 29.8, 19.1, 18.4, 18.3, 18.1, 17.0, 16.5, 16.17.

HRMS (ESI) m/z: [M + H]+ Calcd for $(C_{25}H_{44}N_5O_6)^+$: 510.3286; Found 510.3287.

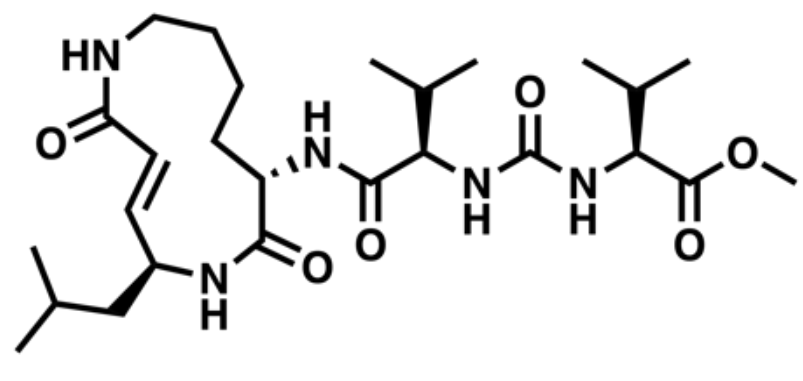

Methyl (((R)-1-(((5S,8S,E)-5-isobutyl-2,7-dioxo-1,6-diazacyclododec-3-en-8-yl)amino)-3-methyl-1-oxobutan-2-yl)carbamoyl)-L-valinate(**A-2**).

Following general method 4 afforded **A-2** as white solid. 39 mg yield:75%

^{1}H NMR (600 MHz, MeOD) δ 7.03 (dd, J = 15.4, 4.9 Hz, 1H), 6.60 (dd, J = 13.9, 8.4 Hz, 1H), 6.39 (d, J = 15.4 Hz, 1H), 4.70 (d, J = 3.9 Hz, 1H), 4.64 (s, 2H), 4.58 (dt, J = 9.8, 5.6 Hz, 1H), 4.22 (t, J = 5.8 Hz, 1H), 4.09 (d, J = 5.3 Hz, 1H), 3.69 (s, 3H), 3.55 – 3.47 (m, 1H), 3.33 (s, 2H), 3.12 (dt, J = 15.7, 3.9 Hz, 1H), 2.21 (h, J = 6.7 Hz, 1H), 2.17 – 2.06 (m, 2H), 1.91 (ddt, J = 14.5, 11.1, 5.3 Hz, 1H), 1.79 (dp, J = 13.4, 6.8 Hz, 1H), 1.50 (dt, J = 8.1, 4.4 Hz, 2H), 1.46 – 1.35 (m, 2H), 1.31 (s, 1H), 1.10 (q, J = 12.5

Hz, 1H), 1.02 – 0.91 (m, 18H).
^{13}C NMR (151 MHz, MeOD) δ 173.1, 172.4, 171.5, 168.2, 159.0, 147.5, 118.2, 59.3, 58.2, 51.8, 51.0, 48.9, 41.7, 38.3, 30.6, 30.2, 29.8, 29.8, 25.0, 21.8, 20.6, 18.4, 18.1, 17.1, 16.6, 16.1.
HRMS (ESI) m/z: [M + H]+ Calcd for $(C_{26}H_{46}N_5O_6)^+$: 524.3443; Found 524.3458.

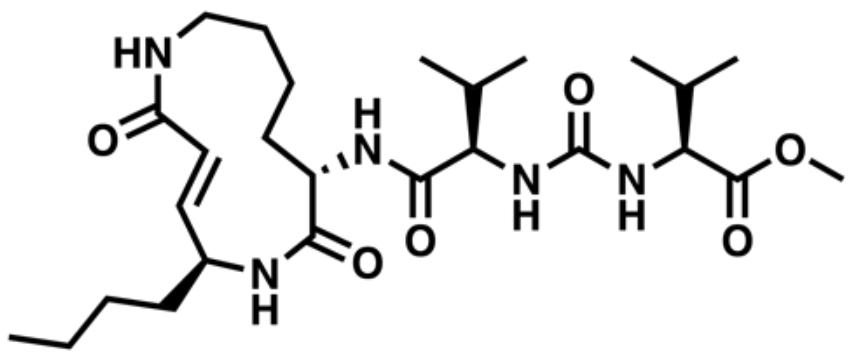

Methyl (((R)-1-(((5S,8S,E)-5-butyl-2,7-dioxo-1,6-diazacyclododec-3-en-8-yl)amino)-3-methyl-1-oxobutan-2-yl)carbamoyl)-L-valinate(**A-3**).
Following general method 4 afforded **A-3** as white solid. 35 mg yield:73%
^{1}H NMR (600 MHz, MeOD) δ 7.03 (ddd, J = 13.7, 8.6, 4.7 Hz, 1H), 6.60 (dd, J = 14.1, 8.4 Hz, 1H), 6.39 (d, J = 15.4 Hz, 1H), 4.72 – 4.65 (m, 2H), 4.64 (s, 2H), 4.47 (dt, J = 10.3, 5.4 Hz, 1H), 4.22 (dd, J = 14.0, 5.4 Hz, 1H), 4.09 (t, J = 5.7 Hz, 1H), 3.71 (d, J = 19.3 Hz, 3H), 3.51 (t, J = 13.7 Hz, 1H), 3.37 (s, 3H), 3.13 (dd, J = 15.5, 4.4 Hz, 1H), 2.21 (h, J = 6.7 Hz, 1H), 2.17 – 2.05 (m, 2H), 1.95 – 1.86 (m, 1H), 1.70 (dt, J = 14.9, 5.2 Hz, 1H), 1.65 (s, 1H), 1.58 (td, J = 9.0, 4.3 Hz, 1H), 1.51 (t, J = 8.4 Hz, 1H), 1.45 – 1.35 (m, 5H), 1.10 (q, J = 12.4 Hz, 1H), 0.96 (ddt, J = 25.9, 12.9, 6.6 Hz, 15H).
^{13}C NMR (151 MHz, MeOD) δ 173.1, 171.5, 168.2, 159.0, 147.4, 118.2, 58.2, 51.0, 50.7, 48.4, 48.0, 47.8, 47.7, 47.6, 47.4, 47.3, 47.1, 38.3, 32.5, 30.6, 30.2, 29.8, 28.54, 22.04, 18.4, 18.1, 17.0, 16.5, 16.1, 12.8.
HRMS (ESI) m/z: [M + H]+ Calcd for $(C_{26}H_{46}N_5O_6)^+$: 524.3443; Found 524.3458.

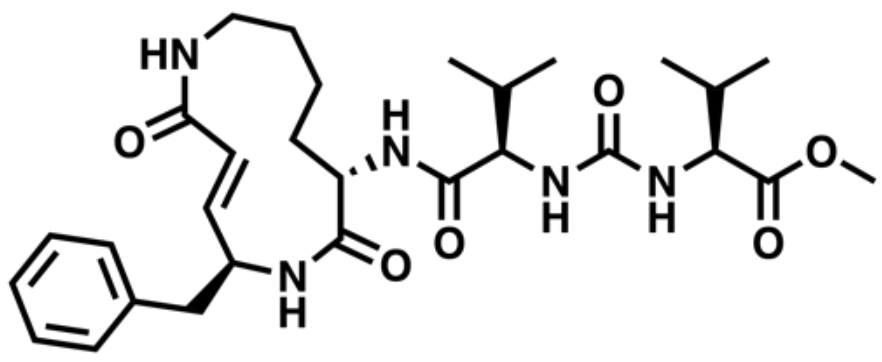

Methyl (((R)-1-(((5S,8S,E)-5-benzyl-2,7-dioxo-1,6-diazacyclododec-3-en-8-yl)amino)-3-methyl-1-oxobutan-2-yl)carbamoyl)-L-valinate(**A-4**).
Following general method 4 afforded **A-4** as white solid. 40 mg yield:71%
1H NMR (400 MHz, DMSO) δ 8.48 (d, J = 8.2 Hz, 1H), 7.88 (d, J = 8.1 Hz, 1H), 7.44 – 7.27 (m, 5H), 7.22 (ddt, J = 8.5, 6.3, 2.8 Hz, 1H), 6.77 (dd, J = 15.3, 4.7 Hz, 1H), 6.50 (d, J = 9.0 Hz, 1H), 6.33 (d, J = 15.4 Hz, 1H), 6.29 (d, J = 8.8 Hz, 1H), 4.60 (s, 1H), 4.11 (ddd, J = 21.0, 8.9, 5.5 Hz, 2H), 3.59 (s, 3H), 3.01 – 2.87 (m, 2H), 2.72 (dd, J = 13.9, 9.6 Hz, 1H), 2.15 (t, J = 13.3 Hz, 1H), 2.02 – 1.82 (m, 2H), 1.55 (s, 1H), 1.45 (s, 1H), 1.34 – 1.20 (m, 2H), 0.96 (q, J = 12.5 Hz, 1H), 0.87 – 0.79 (m, 9H), 0.75 (d, J = 6.9 Hz, 3H).
13C NMR (100 MHz, DMSO) δ 173.5, 171.6, 171.4, 166.1, 157.8, 145.8, 138.8,

129.3, 128.8, 126.8, 119.8, 57.9, 57.8, 52.2, 51.9, 51.4, 38.7, 38.4, 31.7, 31.0, 30.7, 19.5, 19.4, 18.1, 17.9, 17.7.
HRMS (ESI) m/z: [M + H]+ Calcd for $(C_{29}H_{44}N_5O_6)^+$: 558.3286; Found 558.3297.

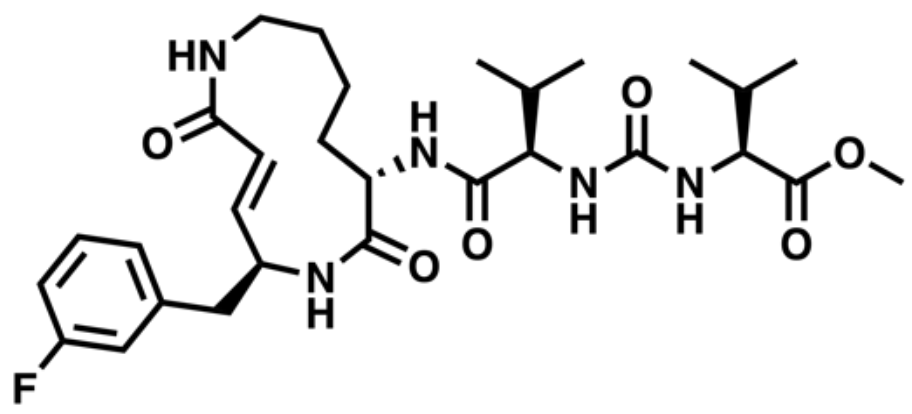

Methyl (((R)-1-(((5S,8S,E)-5-(3-fluorobenzyl)-2,7-dioxo-1,6-diazacyclododec-3-en-8-yl)amino)-3-methyl-1-oxobutan-2-yl)carbamoyl)-L-valinate(**A-5**).
Following general method 4 afforded **A-5** as a white solid. 25 mg yield:43%
^{1}H NMR (400 MHz, DMSO) δ 8.47 (d, J = 8.2 Hz, 1H), 7.89 (d, J = 8.1 Hz, 1H), 7.45 – 7.29 (m, 2H), 7.20 – 7.12 (m, 2H), 7.05 (td, J = 8.6, 2.5 Hz, 1H), 6.78 (dd, J = 15.3, 4.6 Hz, 1H), 6.50 (d, J = 9.0 Hz, 1H), 6.37 – 6.26 (m, 2H), 4.66 – 4.57 (m, 2H), 4.11 (ddd, J = 22.4, 8.9, 5.5 Hz, 2H), 3.59 (s, 3H), 3.28 (d, J = 13.1 Hz, 1H), 2.96 (dd, J = 14.0, 6.1 Hz, 2H), 2.71 (dd, J = 14.0, 10.0 Hz, 1H), 2.20 – 2.07 (m, 1H), 1.92 (dh, J = 26.3, 6.7 Hz, 2H), 1.55 (s, 1H), 1.46 (s, 1H), 1.35 – 1.21 (m, 2H), 0.96 (q, J = 12.5 Hz, 1H), 0.88 – 0.78 (m, 9H), 0.75 (d, J = 6.8 Hz, 3H).
^{13}C NMR (100 MHz, DMSO) δ 173.5, 171.6, 171.4, 166.1, 163.7, 161.3, 157.8, 145.5, 141.8, 141.7, 130.6, 130.6, 125.5, 119.9, 116.2, 116.0, 113.8, 113.5, 57.9, 57.83, 52.05, 51.9, 51.4, 38.4, 38.3, 31.7, 31.0, 30.7, 19.5, 19.4, 18.1, 17.9, 17.7.
HRMS (ESI) m/z: [M + H]+ Calcd for $(C_{29}H_{43}FN_5O_6)^+$: 576.3192; Found 576.3230.

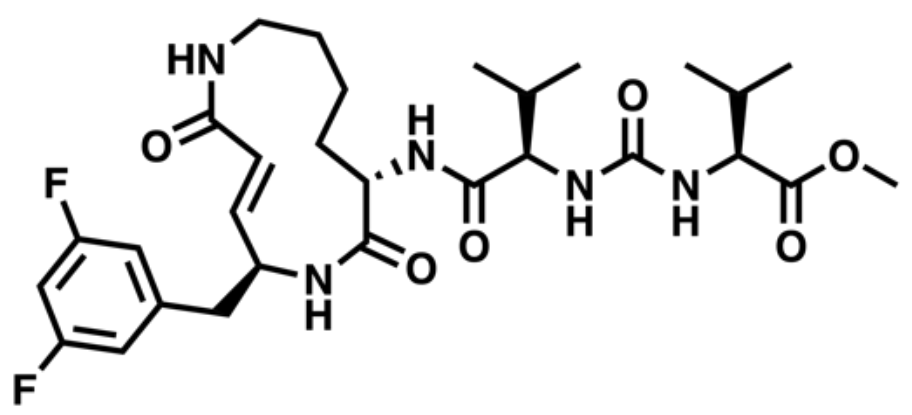

Methyl (((R)-1-(((5S,8S,E)-5-(3,5-difluorobenzyl)-2,7-dioxo-1,6-diazacyclododec-3-en-8-yl)amino)-3-methyl-1-oxobutan-2-yl)carbamoyl)-L-valinate(**A-6**).
Following general method 4 afforded **A-6** as a white solid. 29 mg yield:49%
^{1}H NMR (400 MHz, DMSO) δ 8.49 (d, J = 8.3 Hz, 1H), 7.91 (d, J = 8.1 Hz, 1H), 7.49 – 7.40 (m, 2H), 7.26 – 7.16 (m, 1H), 7.07 (td, J = 8.5, 2.6 Hz, 1H), 6.71 (dd, J = 15.3, 4.7 Hz, 1H), 6.51 (d, J = 8.9 Hz, 1H), 6.35 (d, J = 15.3 Hz, 1H), 6.30 (d, J = 8.8 Hz, 1H), 4.62 (d, J = 14.0 Hz, 2H), 4.11 (ddd, J = 23.8, 8.9, 5.5 Hz, 2H), 3.60 (s, 3H), 2.94 (td, J = 14.9, 8.9 Hz, 2H), 2.82 – 2.72 (m, 1H), 2.17 (t, J = 13.2 Hz, 1H), 1.92 (ddt, J = 32.1, 13.3, 6.6 Hz, 2H), 1.54 (s, 1H), 1.45 (s, 1H), 1.35 – 1.24 (m, 2H), 0.97 (t, J = 12.9 Hz, 1H), 0.87 – 0.79 (m, 9H), 0.76 (d, J = 6.8 Hz, 3H).
^{13}C NMR (100 MHz, DMSO) δ 173.5, 171.6, 171.5, 157.8, 144.8, 104.2, 57.9, 57.80, 51.9, 50.7, 31.7, 31.0, 30.6, 19.5, 19.4, 18.1, 17.9.
HRMS (ESI) m/z: [M + H]+ Calcd for $(C_{29}H_{42}F_2N_5O_6)^+$: 594.3098; Found 594.3090.

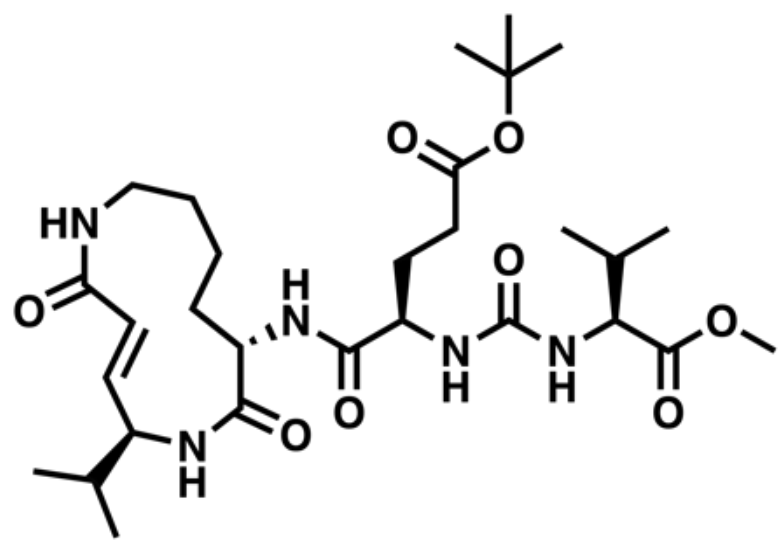

tert-butyl (R)-5-(((5S,8S,E)-5-isopropyl-2,7-dioxo-1,6-diazacyclododec-3-en-8-yl)amino)-4-(3-((S)-1-methoxy-3-methyl-1-oxobutan-2-yl)ureido)-5-oxopentanoate(**B-1**).

Following general method 4 afforded **B-1** as a white solid. 36.8 mg yield:62%

^{1}H NMR (400 MHz, DMSO) δ 8.29 (d, J = 8.2 Hz, 1H), 7.91 (d, J = 7.9 Hz, 1H), 7.37 (t, J = 7.1 Hz, 1H), 6.79 (dd, J = 15.3, 4.9 Hz, 1H), 6.49 (d, J = 8.8 Hz, 1H), 6.40 (d, J = 8.1 Hz, 1H), 6.22 (d, J = 15.3 Hz, 1H), 4.66 (dt, J = 7.7, 3.6 Hz, 1H), 4.26 (td, J = 7.9, 5.5 Hz, 1H), 4.18 – 4.02 (m, 2H), 3.61 (s, 3H), 3.32 – 3.21 (m, 1H), 2.95 (d, J = 14.4 Hz, 1H), 2.15 (q, J = 8.2 Hz, 2H), 2.06 (d, J = 13.6 Hz, 1H), 2.03 – 1.91 (m, 1H), 1.82 – 1.73 (m, 2H), 1.71 – 1.52 (m, 2H), 1.38 (s, 9H), 1.29 (s, 1H), 1.24 (d, J = 14.7 Hz, 1H), 0.95 (dd, J = 11.4, 6.6 Hz, 6H), 0.84 (dd, J = 11.2, 6.8 Hz, 6H).

^{13}C NMR (100 MHz, DMSO) δ 173.4, 172.1, 171.6, 171.2, 166.2, 157.6, 145.3, 120.1, 80.0, 58.1, 56.5, 52.3, 51.9, 51.6, 38.4, 31.6, 31.6, 30.9, 30.7, 30.6, 29.4, 28.2, 20.3, 19.7, 19.46, 18.2, 17.6.

HRMS (ESI) m/z: [M + H]+ Calcd for $(C_{29}H_{50}N_5O_8)^+$: 596.3654; Found 596.3669.

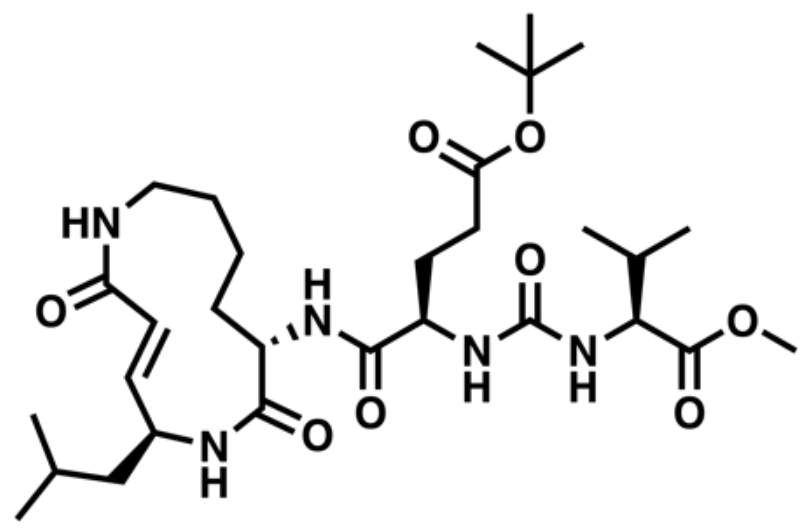

tert-butyl (R)-5-(((5S,8S,E)-5-isobutyl-2,7-dioxo-1,6-diazacyclododec-3-en-8-yl)amino)-4-(3-((S)-1-methoxy-3-methyl-1-oxobutan-2-yl)ureido)-5-oxopentanoate(**B-2**).

Following general method 4 afforded **B-2** as a white solid. 29 mg yield:47%

^{1}H NMR (400 MHz, DMSO) δ 8.36 (d, J = 8.0 Hz, 1H), 7.90 (d, J = 7.8 Hz, 1H), 7.35 (t, J = 7.1 Hz, 1H), 6.78 (dd, J = 15.2, 4.8 Hz, 1H), 6.49 (d, J = 8.8 Hz, 1H), 6.40 (d, J = 8.1 Hz, 1H), 6.25 (d, J = 15.3 Hz, 1H), 4.61 – 4.55 (m, 1H), 4.42 – 4.34 (m, 1H), 4.26 (q, J = 7.4 Hz, 1H), 4.06 (dd, J = 8.7, 5.3 Hz, 1H), 3.61 (s, 3H), 2.95 (d, J = 14.4 Hz, 1H), 2.14 (q, J = 11.1 Hz, 4H), 1.96 (dt, J = 13.3, 6.6 Hz, 1H), 1.82 – 1.74 (m, 1H), 1.73 – 1.64 (m, 2H), 1.64 – 1.56 (m, 1H), 1.41 (s, 2H), 1.38 (s, 10H), 1.28 (d, J = 13.5 Hz, 2H), 1.04 – 0.75 (m, 15H).

^{13}C NMR (100 MHz, DMSO) δ 173.4, 172.1, 171.6, 171.1, 166.2, 157.6, 146.5, 119.4, 80.0, 58.1, 52.3, 52.0, 51.6, 48.7, 41.9, 38.4, 31.6, 30.9, 30.7, 30.4, 29.4, 28.20, 25.34, 23.30, 22.0, 19.4, 18.2, 17.6.

HRMS (ESI) m/z: [M + H]+ Calcd for $(C_{30}H_{52}N_5O_8)^+$: 610.3810; Found 610.3825.

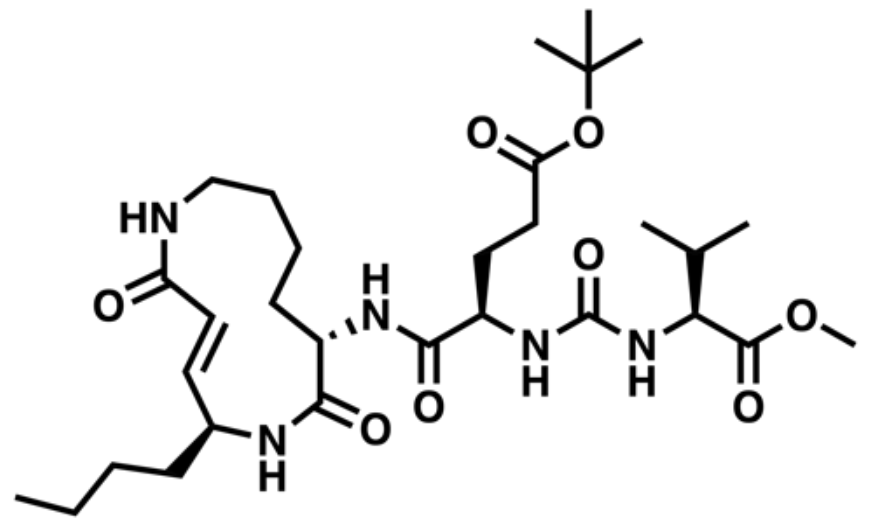

tert-butyl (R)-5-(((5S,8S,E)-5-butyl-2,7-dioxo-1,6-diazacyclododec-3-en-8-yl)amino)-4-(3-((S)-1-methoxy-3-methyl-1-oxobutan-2-yl)ureido)-5-oxopentanoate(**B-3**).

Following general method 4 afforded **B-3** as a white solid. 25 mg yield:41%

^{1}H NMR (400 MHz, DMSO) δ 8.36 (d, J = 8.0 Hz, 1H), 7.90 (d, J = 7.9 Hz, 1H), 7.35 (t, J = 7.0 Hz, 1H), 6.79 (dd, J = 15.3, 4.7 Hz, 1H), 6.49 (d, J = 8.8 Hz, 1H), 6.40 (d, J = 8.1 Hz, 1H), 6.24 (d, J = 15.3 Hz, 1H), 4.59 (dd, J = 8.0, 4.1 Hz, 1H), 4.33 – 4.21 (m, 2H), 4.06 (dd, J = 8.8, 5.4 Hz, 1H), 3.61 (s, 3H), 3.26 (s, 1H), 3.00 – 2.91 (m, 1H), 2.16 (t, J = 8.0 Hz, 2H), 2.09 (t, J = 12.6 Hz, 1H), 1.97 (dq, J = 13.2, 6.7 Hz, 1H), 1.79 (dq, J = 13.6, 7.1 Hz, 1H), 1.65 (dt, J = 14.2, 7.6 Hz, 2H), 1.58 (s, 1H), 1.54 (s, 1H), 1.45 (s, 2H), 1.38 (s, 10H), 1.30 (dt, J = 9.8, 6.7 Hz, 6H), 0.98 (t, J = 12.3 Hz, 1H), 0.91 – 0.82 (m, 9H).

^{13}C NMR (100 MHz, DMSO) δ 173.4, 172.1, 171.6, 171.2, 166.2, 157.6, 146.3, 119.3, 80.0, 58.1, 52.3, 51.9, 51.6, 50.5, 38.4, 32.7, 31.6, 30.9, 30.7, 30.4, 29.4, 28.8, 28.2, 22.3, 19.46, 18.2, 17.6, 14.3.

HRMS (ESI) m/z: [M + H]+ Calcd for $(C_{30}H_{52}N_5O_8)^+$: 610.3810; Found 610.3825.

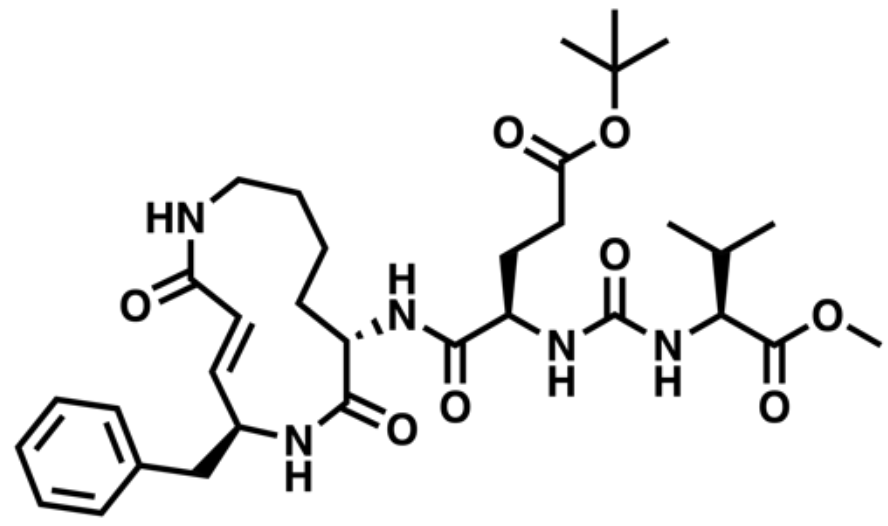

tert-butyl (R)-5-(((5S,8S,E)-5-benzyl-2,7-dioxo-1,6-diazacyclododec-3-en-8-yl)amino)-4-(3-((S)-1-methoxy-3-methyl-1-oxobutan-2-yl)ureido)-5-oxopentanoate(**B-4**).

Following general method 4 afforded **B-4** as a white solid. 35 mg yield:54%

^{1}H NMR (400 MHz, DMSO) δ 8.50 (d, J = 8.2 Hz, 1H), 7.89 (d, J = 7.9 Hz, 1H), 7.40 (d, J = 7.0 Hz, 1H), 7.31 (d, J = 5.4 Hz, 4H), 7.22 (ddd, J = 10.5, 5.9, 3.1 Hz, 1H), 6.78 (dd, J = 15.3, 4.7 Hz, 1H), 6.47 (d, J = 8.8 Hz, 1H), 6.39 – 6.29 (m, 2H), 4.61 (s,

1H), 4.59 – 4.52 (m, 1H), 4.28 – 4.18 (m, 1H), 4.04 (dd, J = 8.8, 5.4 Hz, 1H), 3.59 (s, 3H), 3.00 (s, 1H), 2.97 – 2.87 (m, 1H), 2.76 – 2.66 (m, 1H), 2.15 (q, J = 9.4 Hz, 3H), 1.96 (dq, J = 13.4, 6.8 Hz, 1H), 1.77 (h, J = 7.2 Hz, 1H), 1.63 (dt, J = 14.1, 7.7 Hz, 2H), 1.37 (s, 9H), 1.31 (s, 1H), 1.25 (d, J = 7.0 Hz, 3H), 0.97 (t, J = 13.2 Hz, 1H), 0.83 (dd, J = 11.5, 6.8 Hz, 6H).

^{13}C NMR (100 MHz, DMSO) δ 173.4, 172.1, 171.6, 171.2, 166.1, 157.6, 145.8, 138.8, 129.3, 128.7, 126.8, 119.8, 80.0, 58.0, 52.3, 51.9, 51.6, 38.7, 38.3, 31.6, 30.91, 30.72, 30.4, 29.3, 28.1, 19.4, 18.2, 17.6.

HRMS (ESI) m/z: [M + H]+ Calcd for $(C_{33}H_{50}N_5O_8)^+$: 644.3654; Found 644.3688.

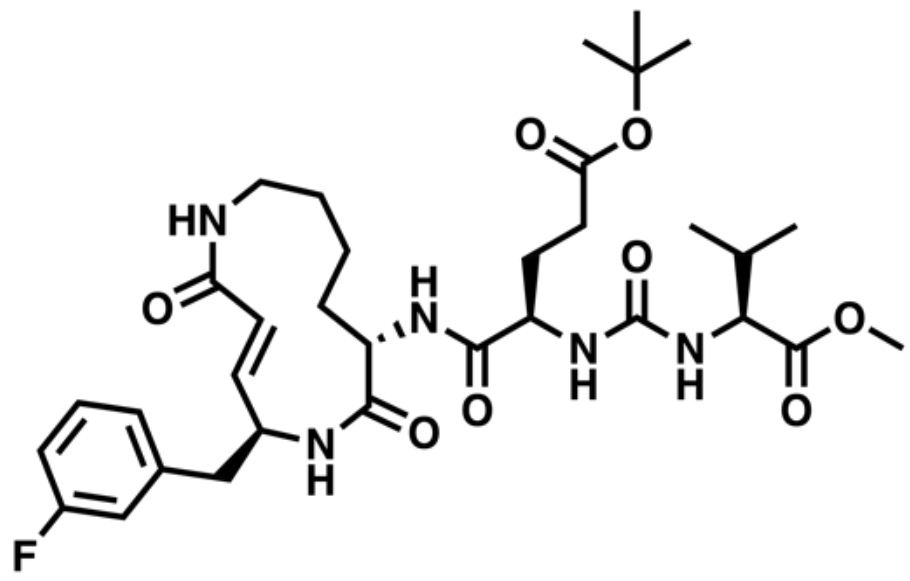

tert-butyl (R)-5-(((5S,8S,E)-5-(3-fluorobenzyl)-2,7-dioxo-1,6-diazacyclododec-3-en-8-yl)amino)-4-(3-((S)-1-methoxy-3-methyl-1-oxobutan-2-yl)ureido)-5-oxopentanoate(**B-5**). Following general method 4 afforded **B-5** as a white solid. 40 mg yield:60%

^{1}H NMR (400 MHz, DMSO) δ 8.49 (d, J = 8.2 Hz, 1H), 7.90 (d, J = 7.9 Hz, 1H), 7.45 – 7.31 (m, 2H), 7.20 – 7.12 (m, 2H), 7.05 (td, J = 8.9, 2.6 Hz, 1H), 6.78 (dd, J = 15.3, 4.7 Hz, 1H), 6.47 (d, J = 8.8 Hz, 1H), 6.39 – 6.28 (m, 2H), 4.62 (s, 1H), 4.55 (s, 1H), 4.23 (q, J = 7.3 Hz, 1H), 4.04 (dd, J = 8.8, 5.3 Hz, 1H), 3.59 (s, 3H), 3.01 – 2.87 (m, 2H), 2.70 (dd, J = 15.0, 11.3 Hz, 2H), 2.12 (q, J = 8.4 Hz, 3H), 1.95 (dt, J = 13.4, 6.7 Hz, 1H), 1.76 (t, J = 7.0 Hz, 1H), 1.62 (dd, J = 14.2, 7.5 Hz, 2H), 1.46 (s, 2H), 1.37 (s, 9H), 1.26 (q, J = 11.4 Hz, 5H), 0.96 (t, J = 12.4 Hz, 1H), 0.83 (dd, J = 11.6, 6.8 Hz, 6H).

^{13}C NMR (100 MHz, DMSO) δ 173.4, 172.1, 171.6, 171.3, 166.1, 157.6, 145.5, 141.7, 130.6, 130.5, 125.5, 119.9, 116.2, 116.0, 113.7, 80.0, 58.1, 52.3, 52.1, 51.9, 51.65, 38.27, 31.6, 30.9, 30.7, 30.4, 29.3, 28.1, 19.4, 18.2, 17.6.

HRMS (ESI) m/z: [M + H]+ Calcd for $(C_{33}H_{49}FN_5O_8)^+$: 662.3560; Found 662.3594.

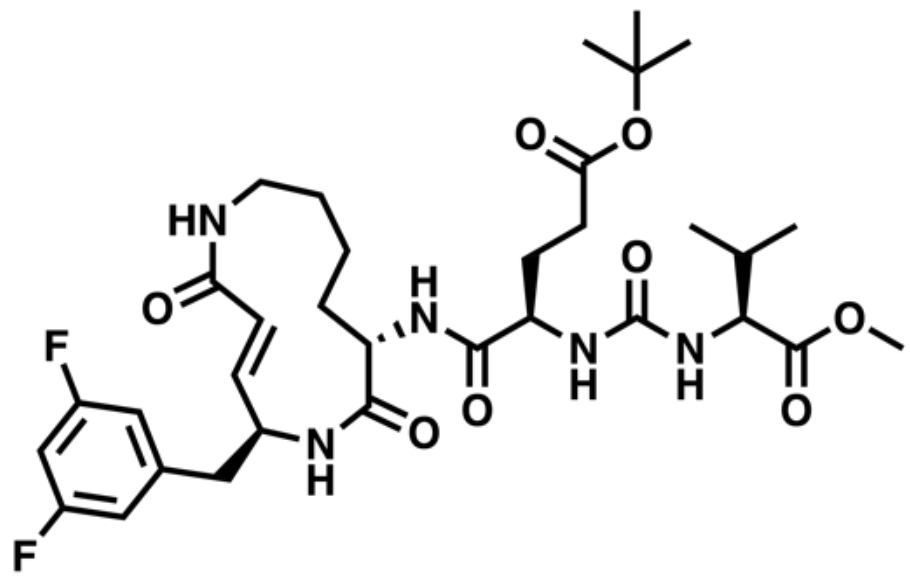

tert-butyl (R)-5-(((5S,8S,E)-5-(3,5-difluorobenzyl)-2,7-dioxo-1,6-diazacyclododec-3-en-8-yl)amino)-4-(3-((S)-1-methoxy-3-methyl-1-oxobutan-2-yl)ureido)-5-oxopentanoate(**B-6**). Following general method 4, afforded **B-6** as a white solid. 39 mg yield:57%

^{1}H NMR (400 MHz, DMSO) δ 8.52 (d, J = 8.3 Hz, 1H), 7.92 (d, J = 7.8 Hz, 1H), 7.44 (q, J = 8.5 Hz, 2H), 7.25 – 7.16 (m, 1H), 7.06 (s, 1H), 6.72 (dd, J = 15.2, 4.7 Hz, 1H), 6.48 (d, J = 8.8 Hz, 1H), 6.40 – 6.32 (m, 2H), 4.63 (s, 1H), 4.54 (d, J = 7.6 Hz, 1H), 4.24 (q, J = 7.2 Hz, 1H), 4.05 (dd, J = 8.8, 5.4 Hz, 1H), 3.59 (s, 3H), 3.13 (d, J = 7.4 Hz, 1H), 3.02 – 2.87 (m, 2H), 2.81 – 2.67 (m, 1H), 2.16 (q, J = 11.8 Hz, 3H), 1.96 (h, J = 6.9 Hz, 1H), 1.77 (dq, J = 14.0, 7.2 Hz, 1H), 1.63 (dq, J = 15.9, 8.2 Hz, 2H), 1.48 – 1.43 (m, 1H), 1.37 (s, 9H), 1.31 (s, 1H), 1.26 (d, J = 7.0 Hz, 4H), 0.96 (t, J = 13.3 Hz, 1H), 0.83 (dd, J = 11.5, 6.7 Hz, 6H).

^{13}C NMR (100 MHz, DMSO) δ 173.4, 172.1, 171.6, 171.4, 166.1, 157.6, 144.8, 132.7, 120.4, 112.0, 104.4, 104.1, 80.0, 58.0, 52.3, 51.9, 51.7, 50.7, 38.3, 31.6, 31.36, 30.92, 30.7, 30.3, 29.3, 28.1, 19.4, 18.2, 17.6.

HRMS (ESI) m/z: [M + H]+ Calcd for $(C_{33}H_{48}F_2N_5O_8)^+$: 680.3465; Found 680.3489.

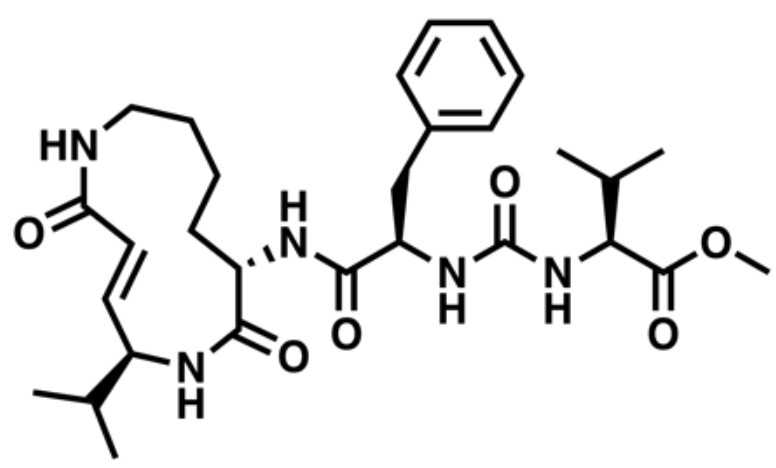

methyl (((R)-1-(((5S,8S,E)-5-isopropyl-2,7-dioxo-1,6-diazacyclododec-3-en-8-yl)amino)-1-oxo-3-phenylpropan-2-yl)carbamoyl)-L-valinate(**C-1**).

Following general method 4 afforded **C-1** as a white solid. 25 mg yield:45%

^{1}H NMR (400 MHz, DMSO) δ 8.25 (d, J = 8.3 Hz, 1H), 7.87 (d, J = 8.1 Hz, 1H), 7.36 (t, J = 7.1 Hz, 1H), 7.27 – 7.11 (m, 5H), 6.78 (dd, J = 15.4, 4.8 Hz, 1H), 6.47 (d, J = 8.9 Hz, 1H), 6.37 (d, J = 8.3 Hz, 1H), 6.18 (d, J = 15.3 Hz, 1H), 4.63 (dt, J = 7.7, 3.6 Hz, 1H), 4.55 (q, J = 7.5 Hz, 1H), 4.12 (td, J = 7.9, 4.7 Hz, 1H), 4.03 (dd, J = 8.8, 5.3 Hz, 1H), 3.60 (s, 3H), 3.24 (q, J = 12.0 Hz, 1H), 2.97 – 2.81 (m, 2H), 2.76 (dd, J = 13.1, 7.4 Hz, 1H), 2.03 – 1.86 (m, 2H), 1.76 (h, J = 6.8 Hz, 1H), 1.35 (d, J = 13.6 Hz, 2H), 1.16 (t, J = 13.5 Hz, 1H), 0.94 (dd, J = 10.7, 6.7 Hz, 6H), 0.79 (t, J = 6.5 Hz, 6H).

^{13}C NMR (100 MHz, DMSO) δ 173.4, 171.2, 171.2, 166.1, 157.3, 145.3, 138.0, 129.8, 128.3, 126.5, 120.1, 57.9, 56.4, 54.3, 51.9, 51.4, 39.3, 38.4, 31.6, 30.9, 30.7, 20.31, 19.78, 19.4, 18.1, 17.4.

HRMS (ESI) m/z: [M + H]+ Calcd for $(C_{29}H_{44}N_5O_6)^+$: 558.3286; Found 558.3297.

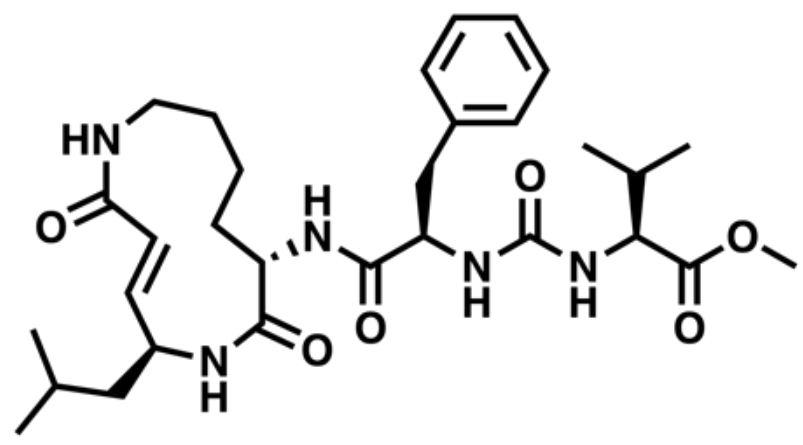

methyl (((R)-1-(((5S,8S,E)-5-isobutyl-2,7-dioxo-1,6-diazacyclododec-3-en-8-yl)amino)-1-oxo-3-phenylpropan-2-yl)carbamoyl)-L-valinate(**C-2**).

Following general method 4, afforded **C-2** as a white solid. 28 mg yield:49%

^{1}H NMR (400 MHz, DMSO) δ 8.31 (d, J = 8.0 Hz, 1H), 7.86 (d, J = 8.0 Hz, 1H), 7.34 (t, J = 7.1 Hz, 1H), 7.27 – 7.21 (m, 2H), 7.18 (dd, J = 9.5, 6.5 Hz, 4H), 6.77 (dd, J = 15.2, 4.7 Hz, 1H), 6.47 (d, J = 8.9 Hz, 1H), 6.36 (d, J = 8.3 Hz, 1H), 6.21 (d, J = 15.3 Hz, 1H), 4.55 (s, 2H), 4.36 (t, J = 6.7 Hz, 1H), 4.03 (dd, J = 8.9, 5.4 Hz, 1H), 3.60 (s, 3H), 3.23 (s, 1H), 2.94 (s, 1H), 2.92 – 2.81 (m, 2H), 2.81 – 2.71 (m, 2H), 2.06 – 1.86 (m, 2H), 1.68 (dt, J = 13.4, 6.8 Hz, 1H), 1.38 (t, J = 7.4 Hz, 4H), 1.18 (d, J = 13.9 Hz, 1H), 0.99 (s, 1H), 0.89 (dd, J = 22.1, 6.6 Hz, 6H), 0.79 (t, J = 6.4 Hz, 7H).

^{13}C NMR (100 MHz, DMSO) δ 173.4, 171.2, 166.2, 157.3, 146.5, 129.8, 128.3, 126.5, 58.0, 54.3, 51.9, 48.7, 41.9, 30.9, 25.3, 23.3, 22.0, 19.4, 18.2.

HRMS (ESI) m/z: [M + H]+ Calcd for $(C_{30}H_{46}N_5O_6)^+$: 572.3443; Found 572.3457.

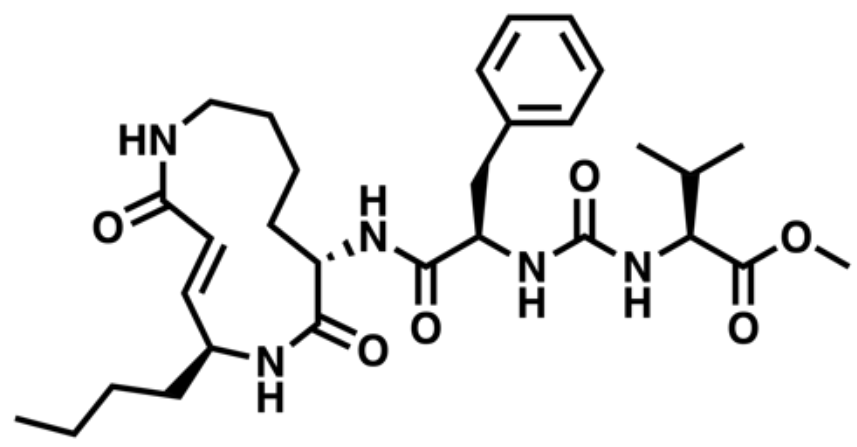

methyl (((R)-1-(((5S,8S,E)-5-butyl-2,7-dioxo-1,6-diazacyclododec-3-en-8-yl)amino)-1-oxo-3-phenylpropan-2-yl)carbamoyl)-L-valinate(**C-3**).

Following general method 4, afforded **C-3** as a white solid. 29 mg yield:50%

^{1}H NMR (400 MHz, DMSO) δ 8.32 (d, J = 8.1 Hz, 1H), 7.87 (d, J = 8.0 Hz, 1H), 7.34 (t, J = 6.9 Hz, 1H), 7.27 – 7.21 (m, 2H), 7.21 – 7.12 (m, 3H), 6.77 (dd, J = 15.3, 4.7 Hz, 1H), 6.47 (d, J = 8.8 Hz, 1H), 6.36 (d, J = 8.3 Hz, 1H), 6.19 (d, J = 15.3 Hz, 1H), 4.60 – 4.49 (m, 2H), 4.29 (s, 1H), 4.03 (dd, J = 8.8, 5.4 Hz, 1H), 3.60 (s, 3H), 3.23 (d, J = 11.8 Hz, 1H), 2.97 – 2.71 (m, 3H), 2.05 – 1.86 (m, 2H), 1.56 (d, J = 9.4 Hz, 1H), 1.39 (s, 1H), 1.33 (d, J = 8.2 Hz, 2H), 1.32 – 1.24 (m, 3H), 1.22 – 1.14 (m, 1H), 0.99 (s, 1H), 0.87 (q, J = 7.6 Hz, 4H), 0.79 (t, J = 6.4 Hz, 6H).

^{13}C NMR (100 MHz, DMSO) δ 173.4, 171.3, 171.2, 166.2, 157.3, 146.3, 138.0, 129.8, 128.3, 126.5, 119.3, 57.9, 54.3, 51.9, 51.5, 50.4, 38.3, 32.7, 30.9, 30.7, 30.6, 28.81, 22.37, 19.4, 18.1, 17.4, 14.2.

HRMS (ESI) m/z: [M + H]+ Calcd for $(C_{30}H_{46}N_5O_6)^+$: 572.3443; Found 572.3457.

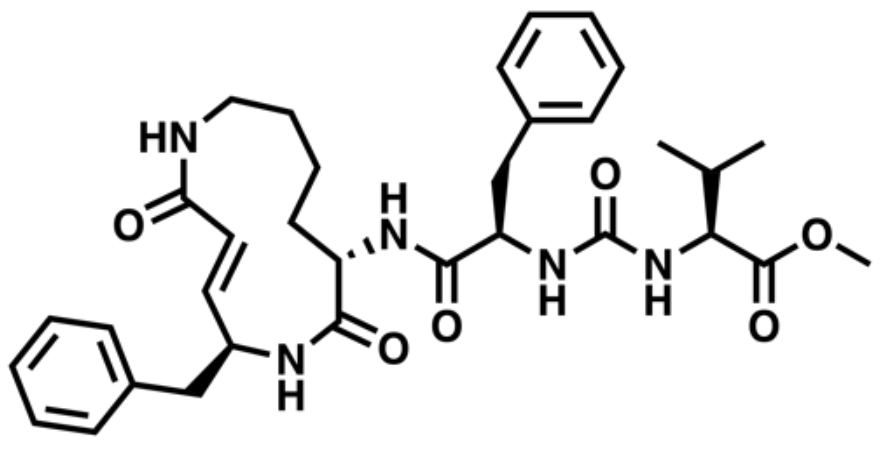

methyl (((R)-1-(((5S,8S,E)-5-benzyl-2,7-dioxo-1,6-diazacyclododec-3-en-8-yl)amino)-1-oxo-3-phenylpropan-2-yl)carbamoyl)-L-valinate(**C-4**).

Following general method 4, afforded **C-4** as a white solid. 30 mg yield:49%

^{1}H NMR (400 MHz, DMSO) δ 8.44 (d, J = 8.2 Hz, 1H), 7.85 (d, J = 8.1 Hz, 1H), 7.39 (t, J = 7.1 Hz, 1H), 7.35 – 7.25 (m, 5H), 7.25 – 7.10 (m, 7H), 6.76 (dd, J = 15.3, 4.6 Hz, 1H), 6.45 (d, J = 8.9 Hz, 1H), 6.36 – 6.25 (m, 2H), 4.60 (s, 1H), 4.52 (q, J = 7.3 Hz, 2H), 4.01 (dd, J = 8.8, 5.4 Hz, 1H), 3.58 (s, 3H), 3.29 (s, 1H), 3.00 – 2.87 (m, 3H), 2.83 (dd, J = 13.5, 6.4 Hz, 1H), 2.79 – 2.64 (m, 3H), 2.11 – 1.99 (m, 1H), 1.91 (dq, J = 13.3, 6.8 Hz, 1H), 1.37 (s, 1H), 1.18 (t, J = 13.6 Hz, 1H), 1.01 (s, 1H), 0.86 (d, J = 13.1 Hz, 1H), 0.78 (t, J = 6.8 Hz, 6H).

^{13}C NMR (100 MHz, DMSO) δ 173.4, 171.2, 166.1, 157.3, 145.8, 138.8, 137.9, 129.7, 129.3, 128.7, 128.3, 126.8, 126.5, 119.7, 58.0, 54.3, 52.2, 51.9, 51.4, 38.7, 38.34, 30.90, 30.8, 30.5, 19.4, 18.1, 17.4.

HRMS (ESI) m/z: [M + H]+ Calcd for $(C_{33}H_{44}N_5O_6)^+$: 606.3286; Found 680.3283.

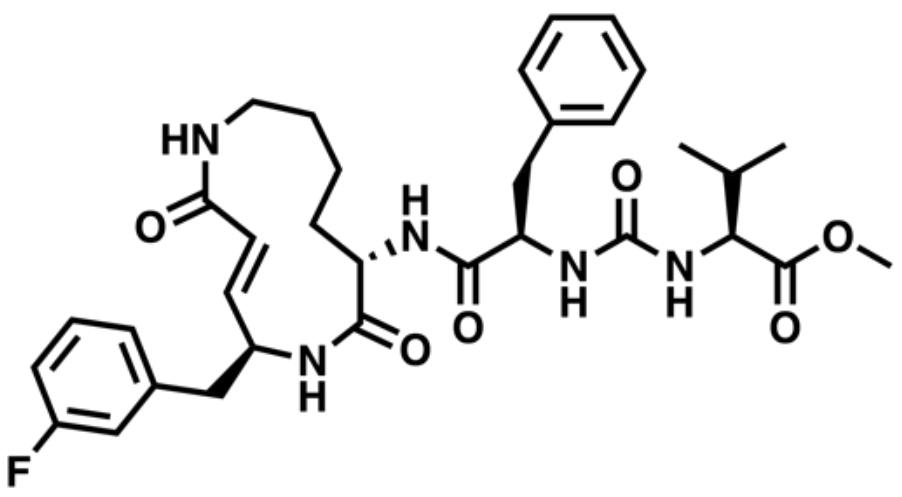

methyl (((R)-1-(((5S,8S,E)-5-(3-fluorobenzyl)-2,7-dioxo-1,6-diazacyclododec-3-en-8-yl)amino)-1-oxo-3-phenylpropan-2-yl)carbamoyl)-L-valinate(**C-5**).

Following general method 4 afforded C-4 as a white solid. 25 mg yield:40%

^{1}H NMR (400 MHz, DMSO) δ 8.43 (d, J = 8.2 Hz, 1H), 7.85 (d, J = 8.0 Hz, 1H), 7.37 (dt, J = 21.7, 7.3 Hz, 2H), 7.18 (dd, J = 17.5, 7.0 Hz, 8H), 7.05 (t, J = 8.8 Hz, 1H), 6.77 (dd, J = 15.4, 4.6 Hz, 1H), 6.45 (d, J = 8.9 Hz, 1H), 6.30 (dd, J = 15.2, 11.7 Hz, 2H), 4.61 (s, 1H), 4.52 (q, J = 7.2 Hz, 2H), 4.01 (dd, J = 8.8, 5.4 Hz, 1H), 3.58 (s, 3H), 3.27 (s, 1H), 3.00 – 2.63 (m, 6H), 2.01 (d, J = 14.0 Hz, 1H), 1.91 (q, J = 6.6 Hz, 1H), 1.37 (d, J = 14.2 Hz, 2H), 1.24 – 1.13 (m, 1H), 1.02 (s, 1H), 0.78 (t, J = 7.1 Hz, 6H).

^{13}C NMR (100 MHz, DMSO) δ 173.4, 171.3, 166.1, 163.7, 157.3, 145.5, 141.7, 137.9, 130.6, 130.5, 129.7, 128.3, 126.5, 125.4, 119.9, 116.2, 116.0, 113.7, 58.0, 54.32, 52.04, 51.9, 51.4, 38.3, 38.2, 30.8, 19.4, 18.1.

HRMS (ESI) m/z: [M + H]+ Calcd for $(C_{33}H_{43}FN_5O_6)^+$: 624.3192; Found 624.3231.

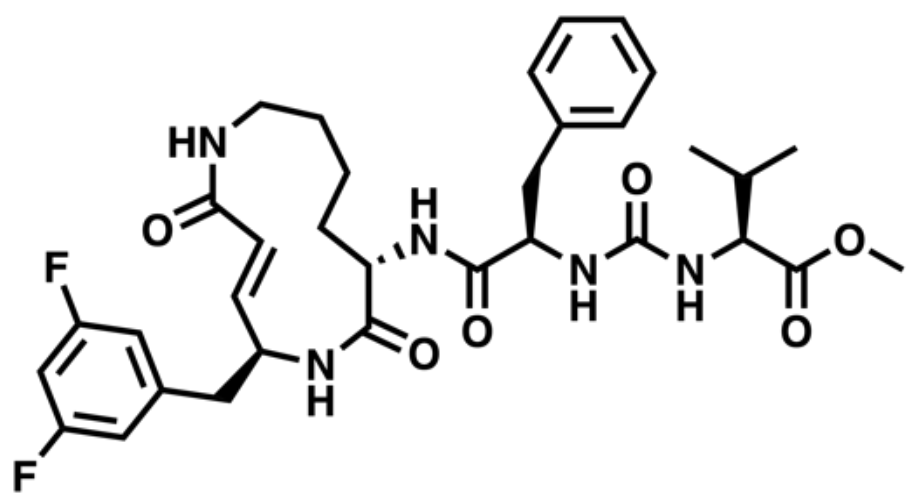

methyl (((R)-1-(((5S,8S,E)-5-(3,5-difluorobenzyl)-2,7-dioxo-1,6-diazacyclododec-3-en-8-yl)amino)-1-oxo-3-phenylpropan-2-yl)carbamoyl)-L-valinate(**C-6**).

Following general method 4 afforded **C-6** as a white solid. 30 mg yield:46%

^{1}H NMR(DMSO, 400 MHz): δ = 8.45 (d, 1H, J=8.4 Hz), 7.88 (d, 1H, J=8.0 Hz), 7.48 – 7.37 (m, 2H), 7.25 – 7.11 (m, 6H), 7.06 (td, 1H, J=8.5, 2.6 Hz), 6.71 (dd, 1H, J=15.3, 4.6 Hz), 6.45 (d, 1H, J=8.9 Hz), 6.37 – 6.27 (m, 2H), 4.63 (d, 1H, J=7.9 Hz), 4.52 (t, 2H, J=7.4 Hz), 4.01 (dd, 1H, J=8.8, 5.4 Hz), 3.59 (s, 3H), 3.26 (t, 1H, J=10.2 Hz), 2.94 (dd, 2H, J=14.5, 6.8 Hz), 2.90 – 2.79 (m, 1H), 2.79 – 2.69 (m, 2H), 2.06 (t, 1H, J=13.5 Hz), 1.91 (dq, 1H, J=13.3, 6.7 Hz), 1.37 (d, 1H, J=14.2 Hz), 1.20 (d, 1H, J=14.0 Hz), 1.02 (s, 1H), 0.84 (d, 1H, J=12.4 Hz), 0.78 (t, 6H, J=6.9 Hz)

^{13}C NMR (100 MHz, DMSO) δ 173.4, 171.3, 171.3, 166.0, 160.3, 157.3, 144.8, 137.9, 132.6, 132.5, 129.7, 128.3, 126.5, 121.7, 120.4, 112.0, 111.8, 104.4, 104.1, 58.0, 54.2, 51.9, 51.5, 50.7, 38.3, 31.3, 30.9, 30.7, 30.4, 19.4, 18.1, 17.4.

HRMS (ESI) m/z: [M + H]+ Calcd for $(C_{33}H_{42}F_2N_5O_6)^+$: 642.3098; Found 642.3101.

Condition Screen for the Deprotection of Macrolactam

Table S1 Conditions that been used in the deprotection of macrolactam

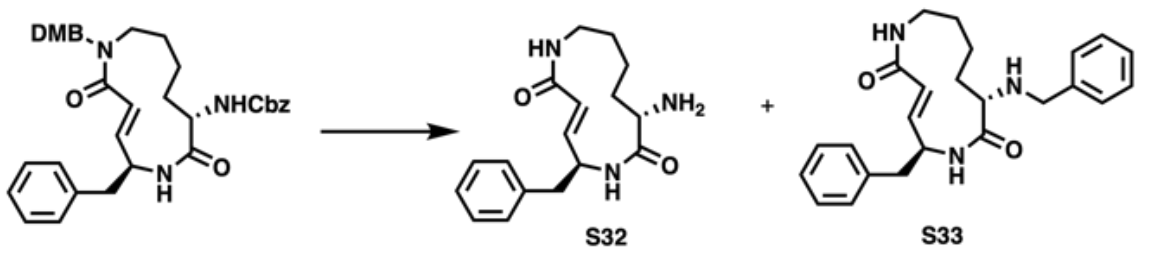

Condition	Reagent/Temperature	Isolated Yield **S32**
1	50%TFA in DCM,10eq Et_3SiH,40°C,overnight	81%
2	33% HBr in Acetic Acid,45mins	40%
3	95% TFA in Water, 10eq TIPS,45°C,overnight	~100% + **S33** (Trace amount)
4	90%TFA, 10%Thioanisole, 60°C,2h	~100%

General Procedure for Syringolin Analog Solid Phase Synthesis

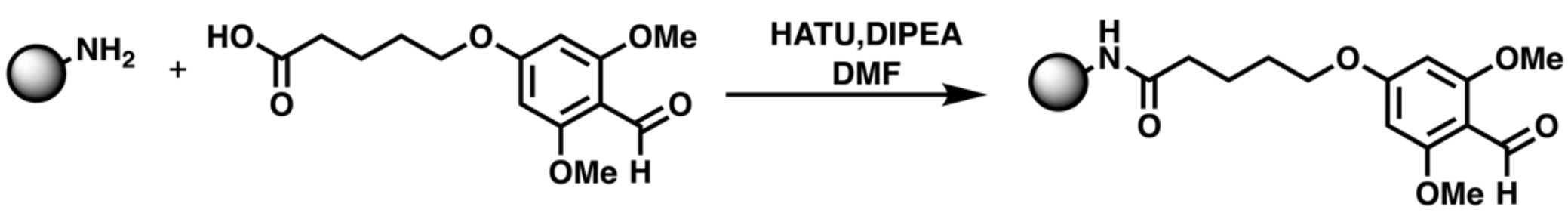

The corresponding resin was weighed in the container. The resin was swelled in DMF (1 g resin/10 mL) for 1 h. The solvent was removed, and the resin was treated

with a 0.2 M mixture of 5-(4-formyl-3,5-dimethoxyphenoxy)pentanoic acid, HATU(3eq),DIPEA(6eq) in DMF and the reaction was agitated for 2h. The solvent was removed and wash with DMF(3 times),MeOH(3 times), and DCM (3 times).

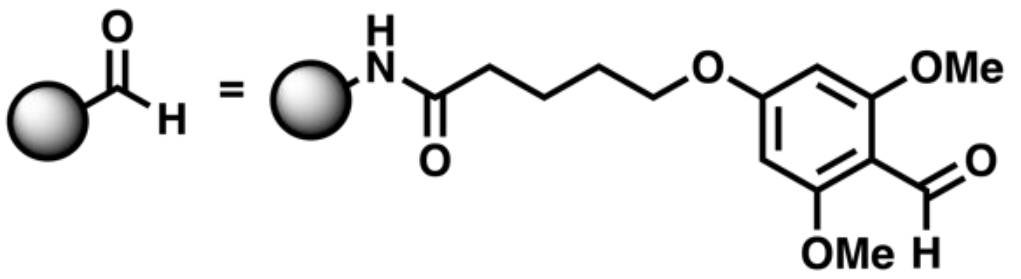

The resin was treated with 10ml of mixture of 1%AcOH/DMF and 10 eq. of Fmoc-Lys-OAllyl, the mixture was agitated for 1. Add 20 eq. $NaCNBH_3$ to the mixture and the reaction was agitated overnight. The solvent was removed, and the resin was washed with DMF(3 times),MeOH(3 times), and DCM (3 times).

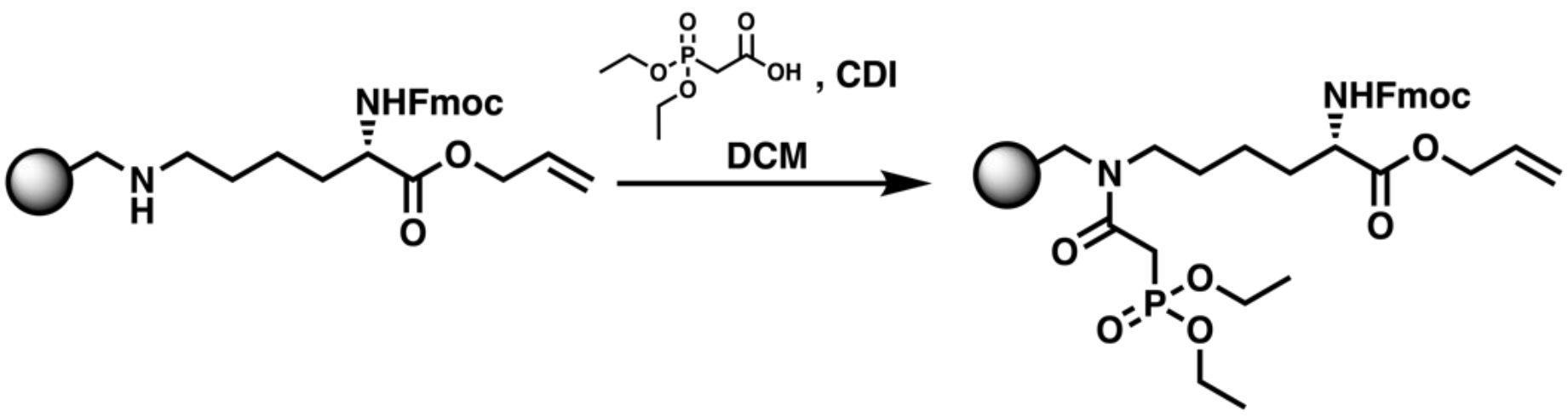

2-(diethoxyphosphoryl)acetic acid(10 eq) was dissolved in 10ml in DCM, CDI(10 eq) was added in the solution and stirred for 20mins. The resin was treated by the aforementioned mixture and agitated for 2h. The solvent was removed, and the resin was washed with DMF(3 times),MeOH(3 times), and DCM (3 times).

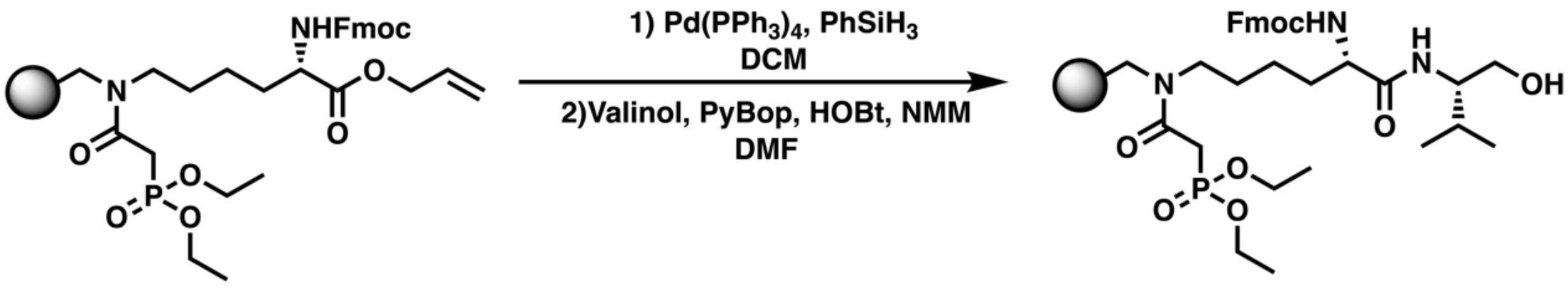

$Pd(PPh_3)_4$ (0.1 eq) and of $PhSiH_3$ (5 eq) were dissolved in 5ml DCM. The resin was treated by the aforementioned mixture and agitated for 15min. Repeat. The solvent was removed, and the resin was washed with DMF(3 times),MeOH(3 times), and DCM (3 times). Then, the resin was treated with 0.2M of mixture of valinol(4 eq) HOBt(4 eq), PyBop(4 eq), NMM(8 eq), and the reaction was agitated for 2h. The solvent was removed, and the resin was washed with DMF(3 times),MeOH(3 times), and DCM (3 times).

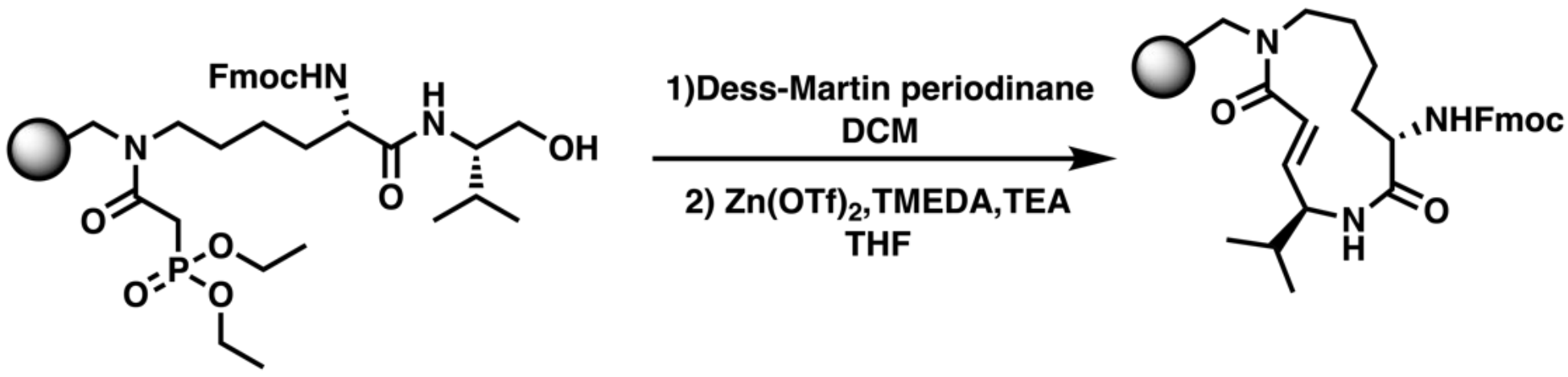

Dess-Martin periodinane(5 eq) was dissolve in DCM/DMF (10:1). The resin was treated by aforementioned mixture and was agitated for 2.5h. The solvent was removed, and the resin was washed with DMF(3 times),MeOH(3 times), and DCM (3 times). Repeat. Then, $Zn(OTf)_2$ (2.2 eq), TMEDA(1.2 eq), and TEA(4 eq) was added in THF. The suspension was vortexed for 15min and added to resin. The reaction was agitated overnight. The resin was washed with DMF(3 times),MeOH(3 times), and DCM (3 times).

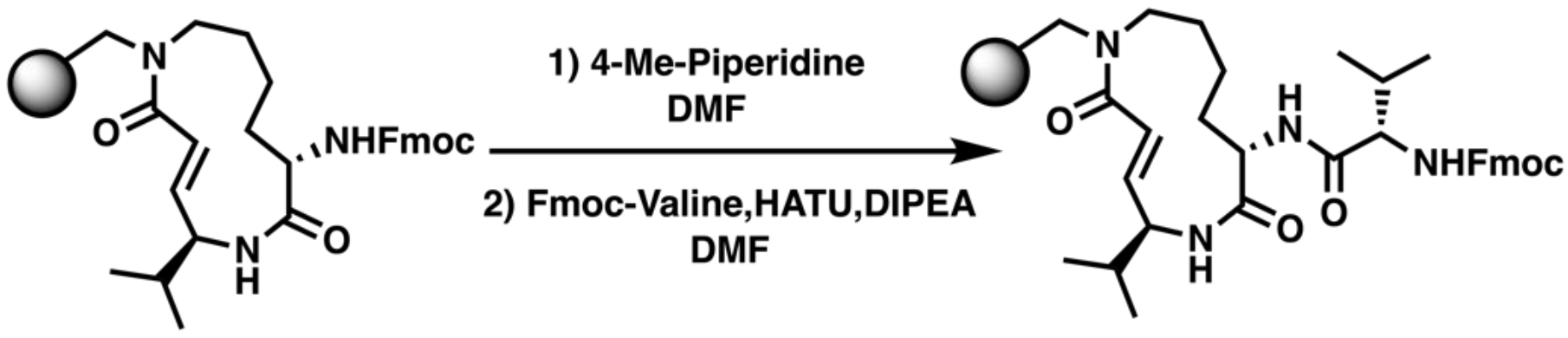

The resin was treated with 20% 4-methyl-piperidine in DMF, and the reaction was agitated for 15 minutes. The solvent was removed, and the resin was washed with DMF(3 times),MeOH(3 times), and DCM (3 times). Repeat. The resin was treated by the 0.2 mixture of Fmoc-valine(3 eq), HATU(3 eq) and DIPEA(6 eq) in DMF and the reaction was agitated for 2h. The solvent was removed, and the resin was washed with DMF(3 times),MeOH(3 times), and DCM (3 times).

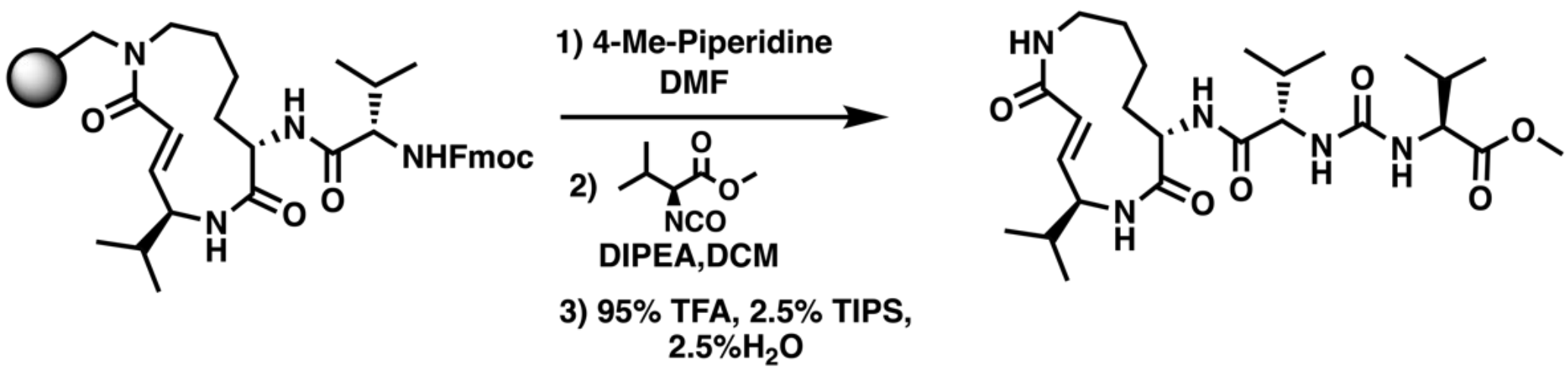

The resin was treated with 20% 4-methyl-piperidine in DMF, and the reaction was agitated for 15 minutes. The solvent was removed, and the resin was washed with DMF(3 times),MeOH(3 times), and DCM (3 times). Repeat. Then, the resin was treated by the mixture of methyl (S)-2-isocyanato-3-methylbutanoate (3 eq) and DIPEA(6 eq) in DCM, the reaction was agitated for 4h.

Resin cleavage is achieved by the treatment of mixture of 95%TFA, 2.5% TIPS, 2.5% water and was agitated for 2h. The solution was filtered and evaporated. The final product was purified by prep-TLC with DCM:MeOH(10:1), afforded the product as a white solid(Yield: 30%).^{1}H NMR is agreement with literature[1].

Figure S1. Lower loading improves the efficiency of macrocyclization in SPPS. Left: Macrocyclization contact in MHBA(0.6 mmol/g) resin. Right: Macrocyclization contact in Tentagel® resin (loading 0.26 millimole/g).

List of Molecular Formula Strings

S1:
NCCCC[C@@H](C(OC(C)(C)C)=O)NC(OCC1=CC=CC=C1)=O

S2:
O=C(OC(C)(C)C)[C@H](CCCCNS(=O)(C1=C([N+]([O-])=O)C=CC=C1)=O)NC(OCC2=CC=CC=C2)=O

S3:
O=C(OC(C)(C)C)[C@H](CCCCN(C)S(=O)(C1=C([N+]([O-])=O)C=CC=C1)=O)NC(OCC2=CC=CC=C2)=O

S4:
O=C(OC(C)(C)C)[C@H](CCCCNC)NC(OCC1=CC=CC=C1)=O

S5:
OC([C@@H](NC(OCC1=CC=CC=C1)=O)CCCCNCC2=CC=C(OC)C=C2OC)=O

S6:
O=C(ON1C(CCC1=O)=O)O[C@@H](C)C2=CC=CC=C2

S7:
O=C(ON1C(CCC1=O)=O)O[C@H](C)C2=CC=CC=C2

S8:
O=C(ON1C(CCC1=O)=O)OC(C2=CC=CC=C2)C3=CC=CC=C3

S9:
OC([C@@H](NC(OCC1=CC=CC=C1)=O)CCCCN(C(CP(OCC)(OCC)=O)=O)CC2=C(OC)C=C(OC)C=C2)=O

S10:
O=C(N[C@@H](CC1=CC=CC=C1)CO)[C@@H](NC(OCC2=CC=CC=C2)=O)CCCCNC(CP(OCC)(OCC)=O)=O

S11:
O=C(N[C@@H](C(C)C)CO)[C@@H](NC(OCC1=CC=CC=C1)=O)CCCCN(CC2=CC=C(OC)C=C2OC)C(CP(OCC)(OCC)=O)=O

S12:

O=C(N[C@@H](CC(C)C)CO)[C@@H](NC(OCC1=CC=CC=C1)=O)CCCCN(CC2=CC=C(OC)C=C2OC)C(CP(OCC)(OCC)=O)=O

S13:

O=C(N[C@@H](CCCC)CO)[C@@H](NC(OCC1=CC=CC=C1)=O)CCCCN(CC2=CC=C(OC)C=C2OC)C(CP(OCC)(OCC)=O)=O

S14:

O=C(N[C@@H](CC1=CC=CC=C1)CO)[C@@H](NC(OCC2=CC=CC=C2)=O)CCCCN(CC3=CC=C(OC)C=C3OC)C(CP(OCC)(OCC)=O)=O

S15:

O=C(N[C@@H](CC1=CC=CC(F)=C1)CO)[C@@H](NC(OCC2=CC=CC=C2)=O)CCCCN(CC3=CC=C(OC)C=C3OC)C(CP(OCC)(OCC)=O)=O

S16:

O=C(N[C@@H](CC1=CC(F)=CC(F)=C1)CO)[C@@H](NC(OCC2=CC=CC=C2)=O)CCCCN(CC3=CC=C(OC)C=C3OC)C(CP(OCC)(OCC)=O)=O

S17:

O=C(N[C@@H](CC1=CC=CC=C1)CO)[C@@H](NC(OC(C)(C)C)=O)CCCCN(C(CP(OCC)(OCC)=O)=O)CC2=C(OC)C=C(OC)C=C2

S18:

O=C(N[C@@H](CC1=CC=CC=C1)CO)[C@@H](NC(OCC2C3=C(C4=C2C=CC=C4)C=CC=C3)=O)CCCCN(C(CP(OCC)(OCC)=O)=O)CC5=C(OC)C=C(OC)C=C5

S19:

O=C(N[C@@H](CC1=CC=CC=C1)CO)[C@@H](N[C@H](C2=CC=CC=C2)C)CCCCN(C(CP(OCC)(OCC)=O)=O)CC3=C(OC)C=C(OC)C=C3

S20:

O=C(N[C@@H](CC1=CC=CC=C1)CO)[C@@H](N[C@@H](C2=CC=CC=C2)C)CCCCN(C(CP(OCC)(OCC)=O)=O)CC3=C(OC)C=C(OC)C=C3

S21:

O=C(N[C@@H](CC1=CC=CC=C1)CO)[C@@H](NC(C2=CC=CC=C2)C3=CC=CC=C3)CCCCN(C(CP(OCC)(OCC)=O)=O)CC4=C(OC)C=C(OC)C=C4

S22:

O=C(/C=C/[C@H](C(C)C)N1)N(CC2=C(OC)C=C(OC)C=C2)CCCC[C@H](NC(OCC3=CC=CC=C3)=O)C1=O

S23:

O=C(/C=C/[C@H](CC(C)C)N1)N(CC2=C(OC)C=C(OC)C=C2)CCCC[C@H](NC(OCC3=CC=CC=C3)=O)C1=O

S24:

O=C(/C=C/[C@H](CCCC)N1)N(CC2=C(OC)C=C(OC)C=C2)CCCC[C@H](NC(OCC3=CC=CC=C3)=O)C1=O

S25:

O=C(/C=C/[C@H](CC1=CC=CC=C1)N2)N(CC3=C(OC)C=C(OC)C=C3)CCCC[C@H](NC(OCC4=CC=CC=C4)=O)C2=O

S26:

O=C(/C=C/[C@H](CC1=CC=CC(F)=C1)N2)N(CC3=C(OC)C=C(OC)C=C3)CCCC[C@H](NC(OCC4=CC=CC=C4)=O)C2=O

S27:
O=C(/C=C/[C@H](CC1=CC(F)=CC(F)=C1)N2)N(CC3=C(OC)C=C(OC)C=C3)CCCC[C@H](NC(OCC4=CC=CC=C4)=O)C2=O
S28:
O=C(/C=C/[C@H](CC1=CC=CC=C1)N2)N(CC3=C(OC)C=C(OC)C=C3)CCCC[C@H](NC(OCC4C5=C(C6=C4C=CC=C6)C=CC=C5)=O)C2=O
S29:
O=C(/C=C/[C@H](CC1=CC=CC=C1)N2)N(CC3=C(OC)C=C(OC)C=C3)CCCC[C@H](NC(O[C@@H](C)C4=CC=CC=C4)=O)C2=O
S30:
O=C(/C=C/[C@H](CC1=CC=CC=C1)N2)N(CC3=C(OC)C=C(OC)C=C3)CCCC[C@H](NC(O[C@H](C)C4=CC=CC=C4)=O)C2=O
S31:
O=C(/C=C/[C@H](CC1=CC=CC=C1)N2)N(CC3=C(OC)C=C(OC)C=C3)CCCC[C@H](NC(OC(C4=CC=CC=C4)C5=CC=CC=C5)=O)C2=O
S32:
O=C(/C=C/[C@H](CC1=CC=CC=C1)N2)N(C)CCCC[C@H](NC(OCC3=CC=CC=C3)=O)C2=O

Syringolin B methyl ester:
O=C(/C=C/[C@H](C(C)C)N1)NCCCC[C@H](NC([C@H](C(C)C)NC(N[C@@H](C(C)C)C(OC)=O)=O)=O)C1=O
A-1:
O=C(/C=C/[C@H](C(C)C)N1)NCCCC[C@H](NC([C@@H](C(C)C)NC(N[C@@H](C(C)C)C(OC)=O)=O)=O)C1=O
A-2:
O=C(/C=C/[C@H](CC(C)C)N1)NCCCC[C@H](NC([C@@H](C(C)C)NC(N[C@@H](C(C)C)C(OC)=O)=O)=O)C1=O
A-3:
O=C(/C=C/[C@H](CCCC)N1)NCCCC[C@H](NC([C@@H](C(C)C)NC(N[C@@H](C(C)C)C(OC)=O)=O)=O)C1=O
A-4:
O=C(/C=C/[C@H](CC1=CC=CC=C1)N2)NCCCC[C@H](NC([C@@H](C(C)C)NC(N[C@@H](C(C)C)C(OC)=O)=O)=O)C2=O
A-5:
O=C(/C=C/[C@H](CC1=CC=CC(F)=C1)N2)NCCCC[C@H](NC([C@@H](C(C)C)NC(N[C@@H](C(C)C)C(OC)=O)=O)=O)C2=O
A-6:
O=C(/C=C/[C@H](CC1=CC(F)=CC(F)=C1)N2)NCCCC[C@H](NC([C@@H](C(C)C)NC(N[C@@H](C(C)C)C(OC)=O)=O)=O)C2=O
B-1:
O=C(/C=C/[C@H](C(C)C)N1)NCCCC[C@H](NC([C@@H](CCC(OC(C)(C)C)=O)NC(N[C@@H](C(C)C)C(OC)=O)=O)=O)C1=O
B-2:

O=C(/C=C/[C@H](CC(C)C)N1)NCCCC[C@H](NC([C@@H](CCC(OC(C)(C)C)=O)N
C(N[C@@H](C(C)C)C(OC)=O)=O)=O)C1=O

B-3:
O=C(/C=C/[C@H](CCCC)N1)NCCCC[C@H](NC([C@@H](CCC(OC(C)(C)C)=O)NC(
N[C@@H](C(C)C)C(OC)=O)=O)=O)C1=O

B-4:
O=C(/C=C/[C@H](CC1=CC=CC(F)=C1)N2)NCCCC[C@H](NC([C@@H](CCC(OC(C
)(C)C)=O)NC(N[C@@H](C(C)C)C(OC)=O)=O)=O)C2=O

B-5:
O=C(/C=C/[C@H](CC1=CC=CC(F)=C1)N2)NCCCC[C@H](NC([C@@H](CCC(OC(C
)(C)C)=O)NC(N[C@@H](C(C)C)C(OC)=O)=O)=O)C2=O

B-6:
O=C(/C=C/[C@H](CC1=CC(F)=CC(F)=C1)N2)NCCCC[C@H](NC([C@@H](CCC(O
C(C)(C)C)=O)NC(N[C@@H](C(C)C)C(OC)=O)=O)=O)C2=O

C-1:
O=C(/C=C/[C@H](C(C)C)N1)NCCCC[C@H](NC([C@@H](CC2=CC=CC=C2)NC(N[
C@@H](C(C)C)C(OC)=O)=O)=O)C1=O

C-2:
O=C(/C=C/[C@H](CC(C)C)N1)NCCCC[C@H](NC([C@@H](CC2=CC=CC=C2)NC(N
[C@@H](C(C)C)C(OC)=O)=O)=O)C1=O

C-3:
O=C(/C=C/[C@H](CCCC)N1)NCCCC[C@H](NC([C@@H](CC2=CC=CC=C2)NC(N[
C@@H](C(C)C)C(OC)=O)=O)=O)C1=O

C-4:
O=C(/C=C/[C@H](CC1=CC=CC=C1)N2)NCCCC[C@H](NC([C@@H](CC3=CC=CC
=C3)NC(N[C@@H](C(C)C)C(OC)=O)=O)=O)C2=O

C-5:
O=C(/C=C/[C@H](CC1=CC=CC(F)=C1)N2)NCCCC[C@H](NC([C@@H](CC3=CC=
CC=C3)NC(N[C@@H](C(C)C)C(OC)=O)=O)=O)C2=O

C-6:
O=C(/C=C/[C@H](CC1=CC(F)=CC(F)=C1)N2)NCCCC[C@H](NC([C@@H](CC3=C
C=CC=C3)NC(N[C@@H](C(C)C)C(OC)=O)=O)=O)C2=O

Procedure for Parasitemia Assay

P. falciparum culturing

All experiments in this study were performed using *P. falciparum* strain W2 (MRA-157). Parasites were cultured at 2% hematocrit in $RPMI_c$ supplemented with 0.25% Albumax, 2g/L sodium bicarbonate, 0.1mM hypoxanthine, 25mM HEPES (pH 7.4), and 50μg/L gentamycin. Incubators were maintained at 37°C, 5% O_2, and 5% CO_2. Parasitemia was maintained below 5% to ensure the health and active replication of parasites.

Dose Response Assay

All compounds were resuspended in pure DMSO at 10mM and stored at -20°C in

10μL aliquots. A 200μM solution of the compounds was then generated and serially diluted √10-fold into a 2% DMSO in 1X PBS solution to maintain the solvent concentration across all wells. Cultures were initiated at 0.5% hematocrit and 0.8% rings, and 190 μL was dispensed into rows of a 96-well U-bottom culture plate. 10μL of the dilution series was then pipetted to mix with the culture, resulting in a 20-fold dilution ranging from 10uM to 1nM. Plates were incubated at 37°C, 5% O_2, and 5% CO_2 for 72 hours, fixed in 1:1 in 2% Paraformaldehyde (PFA) for 1 hour at room temperature, and then stained overnight in 50nM YOYO-1. To quantify parasitemia, each well plate was run on a BDFACSSymphony A1 flow cytometer with a BB15A laser. Each sample was run in technical and biological triplicates, with a collection threshold of 50,000 events. Gating and IC_{50} calculations were then carried out using FlowJo and GraphPad Prism.

Kinetic Evaluation of Syringolin-based Proteasome Inhibitors

Materials

Fluorogenic substrates Suc-LLVY-amc, Z-VLR-amc, Z-LLE-amc, and Ac-nPnD-amc were obtained from Cayman Chemical or AdipoGen, while Ac-FNKL-amc was custom-synthesized by GenScript. Substrates and inhibitors were dissolved in DMSO at concentrations of 10 or 20 mM and stored at −20 °C. Human constitutive proteasome (c20S, E-360-050) was purchased from R&D Systems (Minneapolis, MN, USA) and stored at −80 °C.

Expression and purification of recombinant Pf20S proteasome

Recombinant Pf20S was expressed using the baculovirus–insect cell system, as previously described[2, 3]. Cells were harvested 72 h post-infection, lysed in 50 mM Tris-HCl (pH 7.5), 150 mM NaCl, 1 mM DTT, and 1 mM EDTA, and clarified by centrifugation. The proteasome complexes were purified using tandem Strep-Tactin XT affinity columns, eluted with 50 mM biotin in the same buffer, and further separated by size-exclusion chromatography on a Superose 6 Increase column equilibrated in 50 mM HEPES (pH 7.5), 150 mM NaCl, and 1 mM DTT. Enzymatically active fractions were pooled, concentrated, and stored at –80 °C until use.

Inhibition assays

Initially, a single inhibitor concentration was tested, which demonstrated inhibition of β5 activity. Subsequently, kinetic inhibition (Ki) studies were performed with β5 only. For preincubation assays, 12.5 μM inhibitor was incubated (30 min, room temperature) with either 1 nM human c20S or 1 nM Pf20S, DMSO alone was used as a control. c20S was activated for 1 h at room temperature with 100 nM PA28α, and assays were performed in 50 mM HEPES (pH 7.5) and 1 mM DTT. Substrate hydrolysis was monitored using Suc-LLVY-amc (β5, 65 μM), Z-VLR-amc (β2, 30 μM), and Z-LLE-amc (β1, 80 μM). In parallel, Pf20S was activated for 1 h at room temperature with 200 nM PA28α, and assays were performed in 50 mM HEPES (pH 7.5), 5 mM MgCl2, 1 mM DTT, and 0.01% BSA. Substrate hydrolysis was monitored

using Ac-FNKL-amc (β5, 50 μM), Z-VLR-amc (β2, 25 μM), and Ac-nPnD-amc (β1, 60 μM).

For K_i determination, inhibitor concentrations ranging from 0.603 to 3.125 μM and DMSO control were pre-mixed with substrate, and enzyme was added last. Fluorescence (RFU) was immediately monitored for 3 h. Apparent first-order rate constants (k_{obs}) were calculated and plotted against inhibitor concentration to derive K_i values. All reactions were performed in a final volume of 8 µL per well. Inhibitors were dispensed using an Echo650 liquid handler (Beckman Coulter, Brea, CA, USA). Pre–steady-state kinetic measurements were carried out for the irreversible inhibitor, and data were analyzed using GraphPad Prism v10.4.1. All assays were performed in triplicate in 384-well black plates (Greiner Bio-One, Monroe, NC, USA) at 37 °C. Fluorescence was recorded with a Synergy HTX Multi-Mode Microplate Reader (BioTek, Winooski, VT, USA) using excitation at 360 nm and emission at 460 nm.

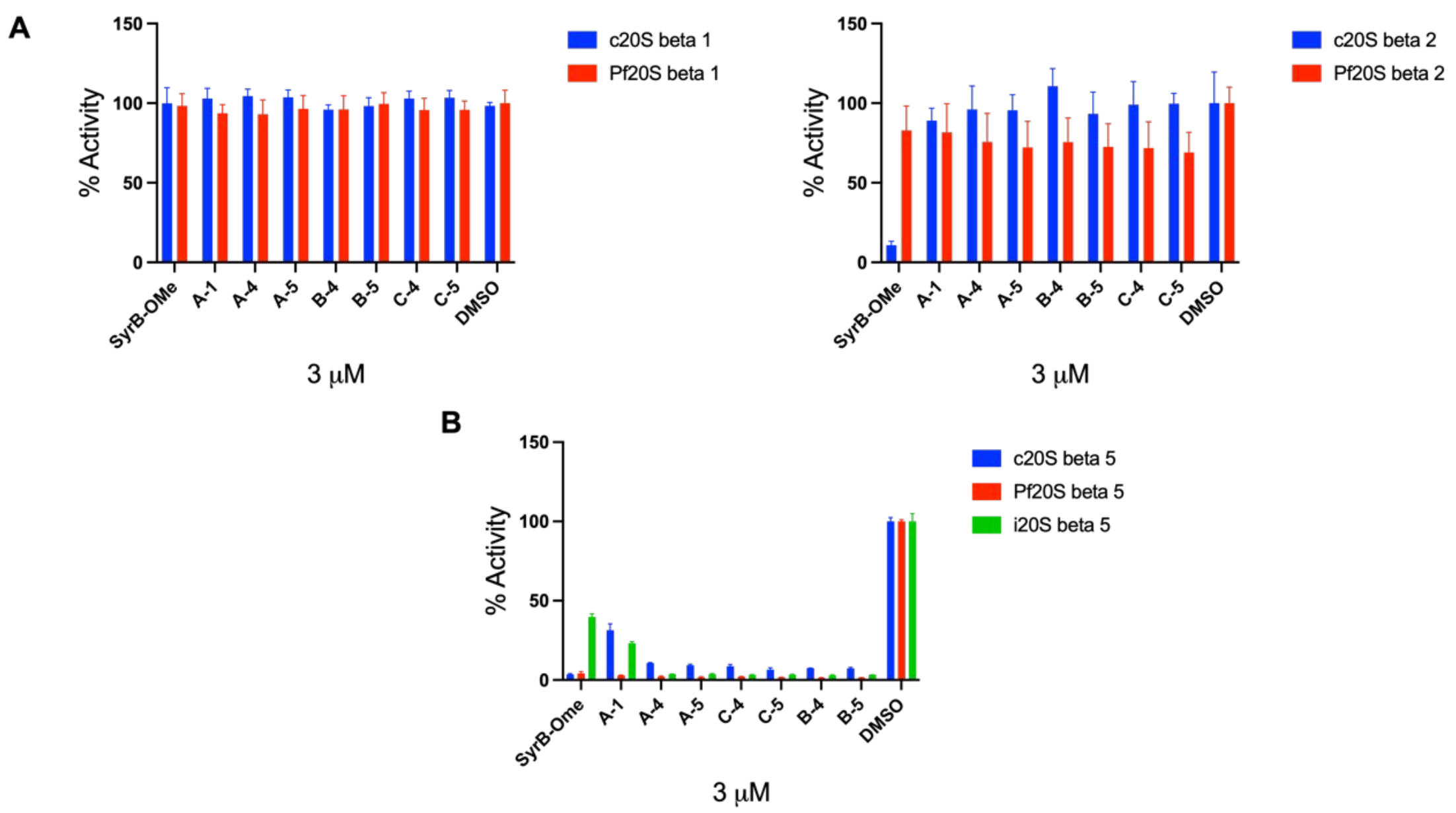

Figure S2. Single-dose inhibition data for (A) syringolin B methyl ester and analogs thereof against the b1, b2, and b5 activities of the human constitutive proteasome and for (B) syringolin B methyl ester, A1, A4, A5, C4, C-5, B-4, and B-5 against the b5 activity of the both the human constitutive- and immune-proteasomes.

Table S2. Kinetic Evaluation of Syringolin Analogs

i20S beta 5	SyrB-OMe	A-1	A-4	A-5	C-4	C-5	B-4	B-5
Ki (nM)	3186.50	958.50	4.92	8.08	5.23	6.11	5.37	6.81
Ki (SE)	1683.00	111.45	1.38	1.66	1.45	1.21	1.25	1.17
kinact/Ki ($M^{-1}s^{-1}$)	173.48	408.56	32821.87	26588.24	32676.86	35384.62	41489.76	37900.15
SE	113.14	52.87	9475.26	5700.65	9353.72	7294.25	10017.90	6795.85

Pf20S beta 5	SyrB-OMe	A-1	A-4	A-5	C-4	C-5	B-4	B-5
Ki (nM)	479.09	412.55	55.40	33.89	18.39	6.70	61.95	15.00
Ki (SE)	100.36	38.36	12.17	12.29	3.64	2.63	8.44	2.16
kinact/Ki ($M^{-1}s^{-1}$)	933.43	1570.74	8620.94	10740.34	23647.55	53305.28	23129.86	48200.00
SE	205.96	159.45	2011.54	4099.20	4991.91	21830.90	3855.69	7651.49

c20S beta 5	SyrB-OMe	A-1	A-4	A-5	C-4	C-5	B-4	B-5
Ki (nM)	598.00	>3000	611.50	150.90	118.90	463.55	43.41	126.00
Ki (SE)	90.20		54.55	46.47	69.90	111.10	17.83	21.69
kinact/Ki ($M^{-1}s^{-1}$)	1397.16	N/A	704.33	1744.86	1557.61	1180.46	5418.11	4112.70
SE	228.46		68.19	559.60	950.34	323.41	2319.71	798.72

Procedure for Human Toxicity Assay

HFF cells were resuspended in phenol red-free DMEM (Gibco) supplemented with 10% Animal Serum Complex (Gemini Bio Products) and 100 units/mL Penicillin-Streptomycin (Thermo Scientific), and seeded into 96-well black/clear bottom plates (Advanced TC™, Greiner) at a density of 400,000 cells/mL (50 μL per well).[4] Cell density was determined using a Cellometer® Auto T4 cell counter. After a 3-hour adhesion period at 37 °C in a humidified incubator with 5% CO_2, 50 μL of the same medium containing serial dilutions of the test compounds (final concentrations: 0–500 μM; 1% v/v DMSO) was added to each well, bringing the total volume to 100 μL. Plates were incubated for an additional 24 hours under the same conditions. Then, 20 μL of CellTiter-Blue™ Reagent (Promega) was added to each well, and the plates were returned to the incubator for 3 hours to allow for the conversion of resazurin to resorufin. Fluorescence was measured using a Cytation 3 plate reader (BioTek) at excitation/emission wavelengths of 560/590 nm.[5]

Cell viability in each well was normalized to the DMSO-treated control (100% viability) and 0.1% (v/v) SDS-treated cells (0% viability), and fitted to the following equation[6]:

$$\text{Cell Viability} = \text{Bottom} + \frac{\text{Top} - \text{Bottom}}{1 + (\frac{x}{CC_{50}})^{H}} \qquad \text{(eq. 1)}$$

where *Bottom* and *Top* were constrained to 0% and 100%, respectively; *x* represents the final compound concentration (in molar units); and *H* is the Hill slope.

To minimize edge effects, cells were seeded only in the center wells of each plate. All experiments were performed in biological triplicate.

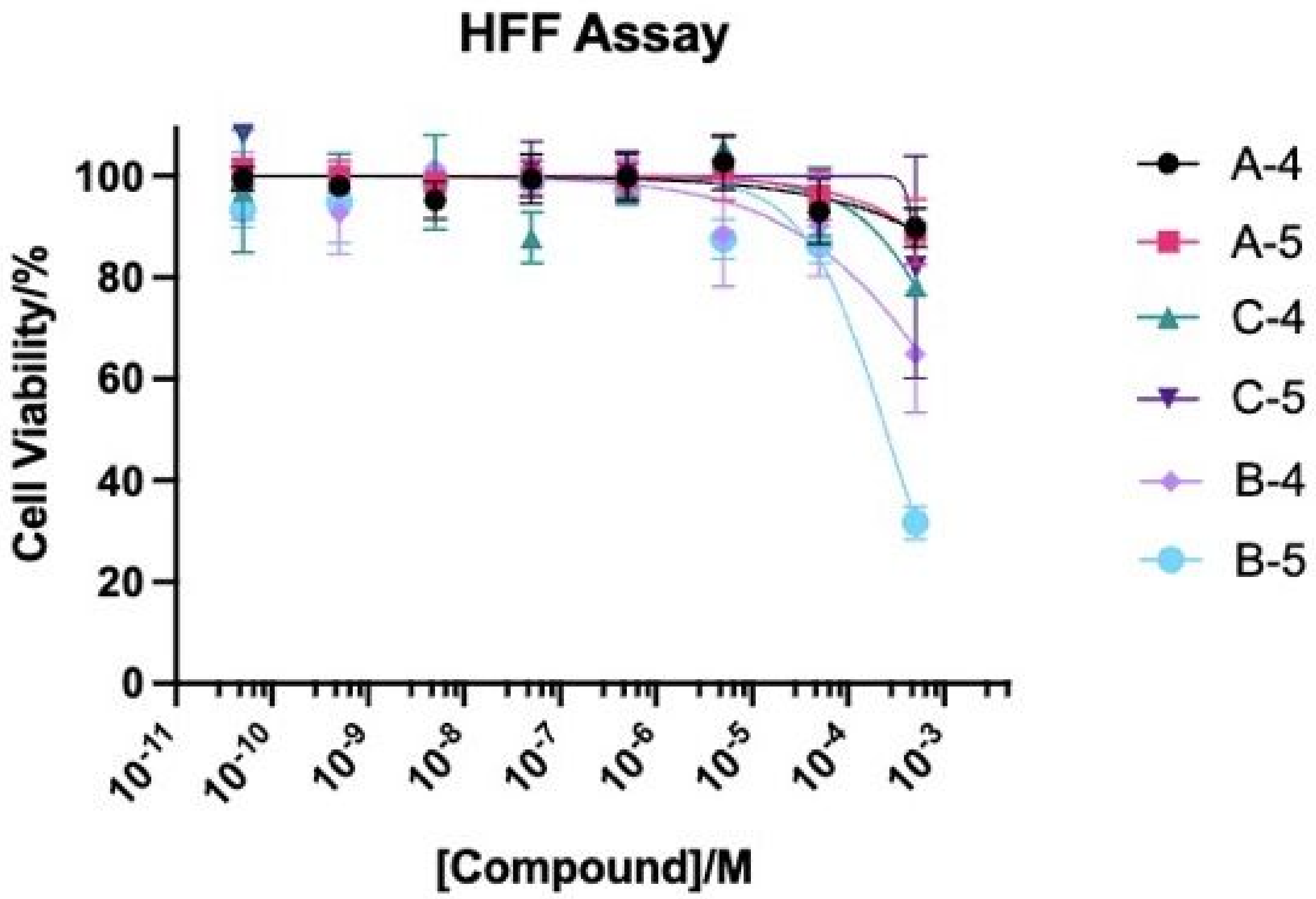

Figure S3. The compounds exhibited no to low toxicity toward HHFs.

Table S3. Summarized CC_{50} of SyrB Compound Series

**Name**	**CC_{50} / μM**
A-4	> 500
A-5	> 500
C-4	> 500
C-5	> 500
B-4	> 500
B-5	246 ± 33

Cryo-EM method

Cryo-EM sample preparation and data collection

The *Pf*20S•**C-5** adduct was prepared by incubating 2.5 mg/mL *Pf*20S proteasome with 100 μM **C-5** for 1 hour at room temperature in vitrification buffer (50 mM HEPES pH 7.5, 1 mM DTT, 1% DMSO). Afterwards, 3 μL of sample was applied to a non-glow-discharged graphene oxide (GO)-coated holey carbon grid (Quantifoil R1.2/1.3

on a gold 300 mesh support), which were prepared in house as previously described[7]. After 30 s, grids were blotted for 4 s at room temperature and 100% humidity using a FEI Vitrobot Mark IV (Thermo Fisher Scientific), followed by plunge freezing in liquid ethane. A total of 14,303 super-resolution movies were collected at a nominal magnification of 105,000× (physical pixel size: 0.417 Å/pixel) on a Titan Krios (Thermo Fisher Scientific, Waltham, MA) operated at 300 kV and equipped with a K3 direct electron detector and BioQuantum energy filter (Gatan, Inc., Pleasanton, CA) set to a slit width of 20 eV. A defocus range of −0.8 to −1.8 μm was used with a total exposure time of 2.024 s fractionated into 0.025-s subframes. The total dose for each movie was 46 electrons/Å^2. Movies were motion corrected using MotionCor2 [8] in Scipion [9] and were Fourier cropped by a factor of 2 to a final pixel size of 0.834 Å/pixel.

The h20S•**C-5** adduct was prepared by incubating 0.35 mg/mL h20S proteasome with 100 μM C-5 for 1 hour at room temperature in vitrification buffer (50 mM Tris pH 7.5, 100 mM NaCl, 1 mM EDTA, 1% DMSO). Afterwards, 3 μL of sample was applied to a non-glow-discharged graphene oxide (GO)-coated holey carbon grid (Quantifoil R1.2/1.3 on a gold 300 mesh support), which were prepared in house as previously described [7]. After 30 s, grids were blotted for 4 s at room temperature and 100% humidity using a FEI Vitrobot Mark IV (Thermo Fisher Scientific), followed by plunge freezing in liquid ethane. A total of 3,670 movies were collected at a nominal magnification of 130,000× (physical pixel size: 0.927 Å/pixel) on a Titan Krios G4 (Thermo Fisher Scientific, Waltham, MA) operated at 300 kV and equipped with a Falcon 4i direct electron detector and Selectris X energy filter (Thermo Fisher Scientific, Waltham, MA) set to a slit width of 20 eV. A defocus range of −0.8 to −1 μm was used with a total exposure time of 3.05 s fractionated into 936 EER frames grouped into 40 subframes. The total dose for each movie was 45 electrons/Å^2. Movies were motion corrected using MotionCor2 [8] in Scipion [9].

Cryo-EM data processing

For both datasets, all image processing was performed in CryoSPARC v4[10].

For the *Pf*20S•**C-5** dataset, motion-corrected and dose-weighted micrographs were subject to Patch CTF estimation and curated to low-resolution micrographs (CTF fit resolution > 4 Å), or those with significant ice contamination, resulting in 13,263 remaining micrographs. Particles were picked using Blob Picker and extracted with a box size of 400 pixels binned to 100 pixels. Two rounds of reference-free 2D classification were performed to remove contaminants, incompletely assembled or misassembled 20S proteasomes, and low-resolution classes, resulting in 52,291 remaining particles. These were subject to *ab initio* model generation (k=3) and heterogenous refinement using the *ab initio* models as references, resulting in low-resolution classes that lacked features of mature 20S proteasomes. Therefore, another round of 2D classification was performed on the individual particle stacks per 3D class. The first class primarily had side view 20S particles, the second class top view 20S particles, and the third class misassembled asymmetric 20S particles. Particles from the highest-resolution 2D classes in the former two 3D classes were

pooled and re-extracted with a box size of 400 pixels binned to 300 pixels, giving a

	*Pf*20S•**C-5**	h20S•**C-5**
**PDB accession code**	9YUY	9YUZ
**EMDB accession code**	EMD-73509	EMD-73510
**Data collection and processing**		
Microscope and camera	Krios, Gatan K3	Krios G4, Falcon 4i
Nominal magnification	105,000	130,000
Voltage (kV)	300	300

stack of 36,364 particles. This stack was subject to one round of homogenous refinement, followed by one round of non-uniform refinement (imposing C2 symmetry with global CTF and per-particle defocus refinement) to give the final *Pf*20S•**C-5** map with an estimated global resolution of 2.58 Å. To assess the stoichiometry of the liganded complex, we performed non-uniform refinement without imposing symmetry, giving a slightly lower-resolution (2.82 Å) map that nevertheless displayed full occupancy at both beta 5 sites, with small-molecule resolution comparable to that of the surrounding protein.

For the h20S•**C-5** dataset, motion-corrected and dose-weighted micrographs were subject to Patch CTF estimation and curated to low-resolution micrographs (CTF fit resolution > 4 Å), or those with significant ice contamination, resulting in 3,118 remaining micrographs. Particles were picked using Template Picker (using 2D classes from a previous in-house h20S dataset as templates) and extracted with a box size of 400 pixels binned to 100 pixels. Two rounds of reference-free 2D classification were performed to remove contaminants and low-resolution classes, resulting in 81,101 remaining particles. These were subject to *ab initio* model generation (k=3) and heterogenous refinement using the *ab initio* models as references. Particles from the highest-resolution 3D class were re-extracted with a box size of 400 pixels without binning, giving a stack of 50,158 particles. This stack was subject to one round of homogenous refinement, followed by one round of non-uniform refinement (imposing C2 symmetry with global CTF and per-particle defocus refinement) to give the final h20S•**C-5** map with an estimated global resolution of 2.51 Å.

Model building

For each refined map, initial models were constructed by fitting a published apo 20S proteasome model (*Pf*20S: PDB 6MUW; h20S: PDB 7NAN) to the map using UCSF ChimeraX [11]. CIF restraint files of both the reduced **C-5** species and the Thr•**C-5** conjugate were generated using ACEDRG [12] within the CCP4 i2 suite [13]. The reduced C-5 species was fitted into the density and linked to the Thr1 sidechain oxygen using COOT [14]. Each bound model underwent iterative rounds of refinement in Phenix, COOT, and ISOLDE [15]. A CIF restraint file for the Thr:**C-5** conjugate and a definition file specifying the link between Thr1 and **C-5** were provided as input for Phenix. The refined models were deposited to the Protein Data Bank with accession codes 9YUY for *Pf*20S•**C-5** and 9YUZ for h20S•**C-5**.

Data acquisition software	SerialEM	EPU
Exposure navigation	Image shift	Image shift
Electron exposure ($e^{-}/Å^2$)	46	45
Defocus range (μm)	-0.8 to -1.8	-0.8 to -1.0
Pixel size (Å)	0.834	0.927
Symmetry imposed	C2	C2
Initial particle images (no.)	52,291	81,101
Final particle images (no.)	36,364	50,210
Map resolution (Å)	2.6	2.5
FSC threshold	0.143	0.143
Map resolution range (Å)	2.5 to 41	2.3 to 44
**Refinement**		
Model resolution (Å)	2.8	2.7
FSC threshold	0.5	0.5
Map sharpening B factor ($Å^2$)	-65	-51
**Model composition**		
Non-hydrogen atoms	49,902	46,426
Protein residues	6,284	6,246
Ligands	2	2
**B factors ($Å^2$)**		
Protein	52.9	42.4
Ligands	37.0	42.8
**R.M.S. deviations**		
Bond lengths (Å)	0.024	0.004
Bond angles (°)	1.978	0.589
**Validation**		
MolProbity score	1.51	1.72
Clash score	3.83	6.73
Rotamer outliers (%)	1.59	2.84
**Ramachandran plot**		
Favored (%)	96.91	98.03
Allowed (%)	2.97	1.97
Outliers (%)	0.13	0

Table S4. Cryo-EM data collection, refinement, and validation statistics.

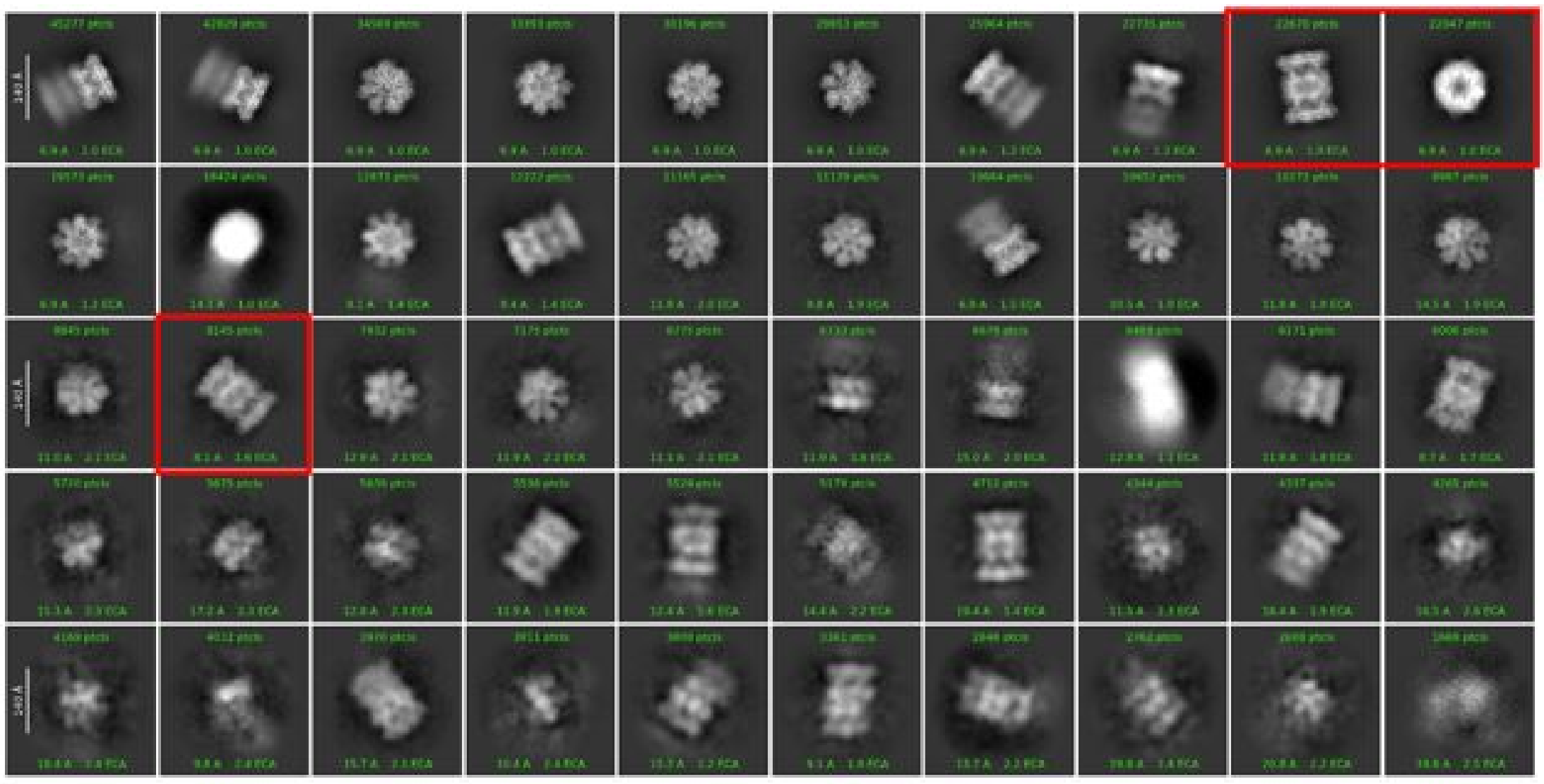

Figure S4. 2D class averages of the *Pf*20S•**C-5** dataset after 2 rounds of reference-free 2D classification, indicating that most of the particles are incompletely assembled or misassembled asymmetric proteasomes. Particles in the highlighted averages, which had features of mature 20S proteasomes, were selected for *ab initio* map generation.

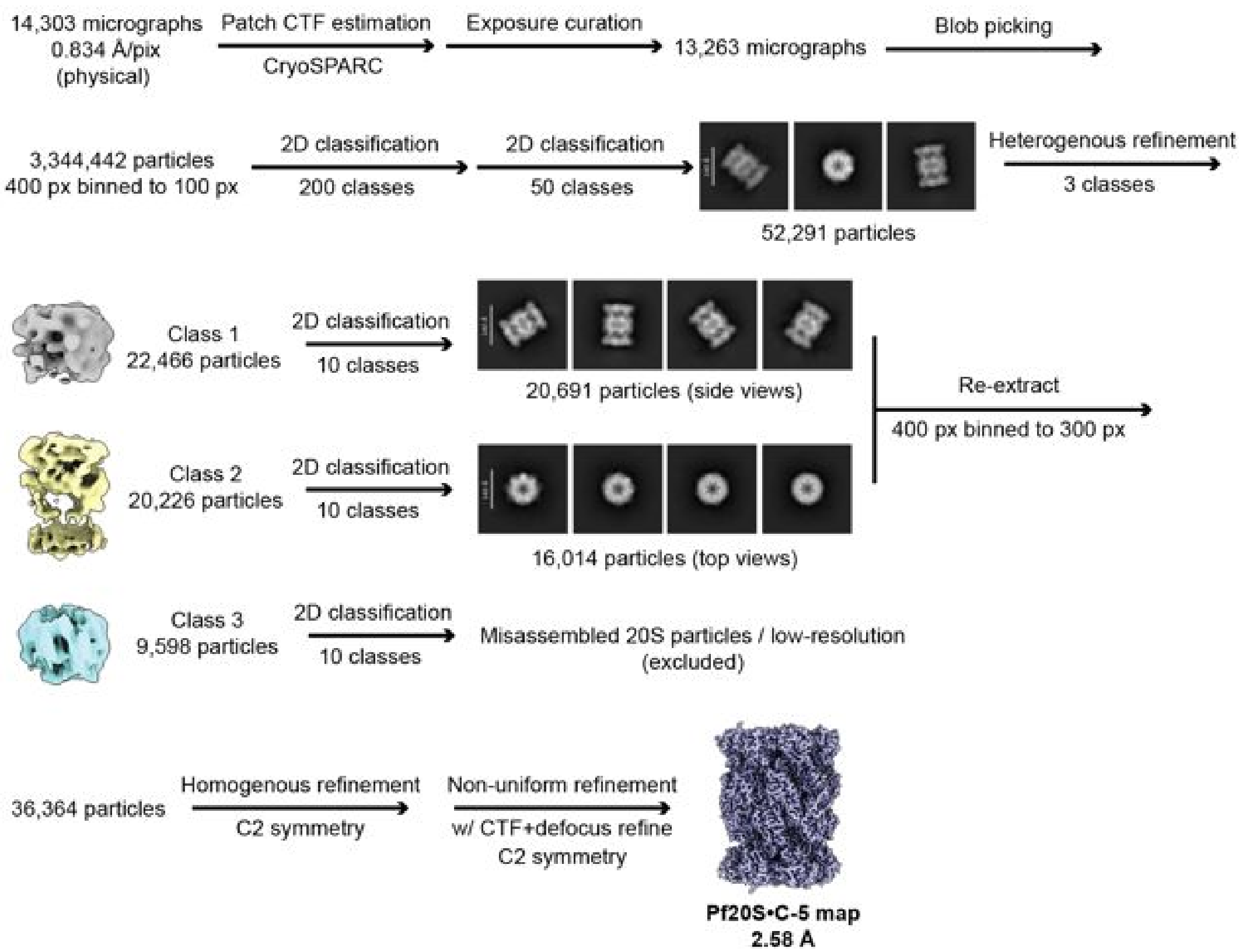

Figure S5. Cryo-EM data processing flowchart for the *Pf* 20S•**C-5** complex.

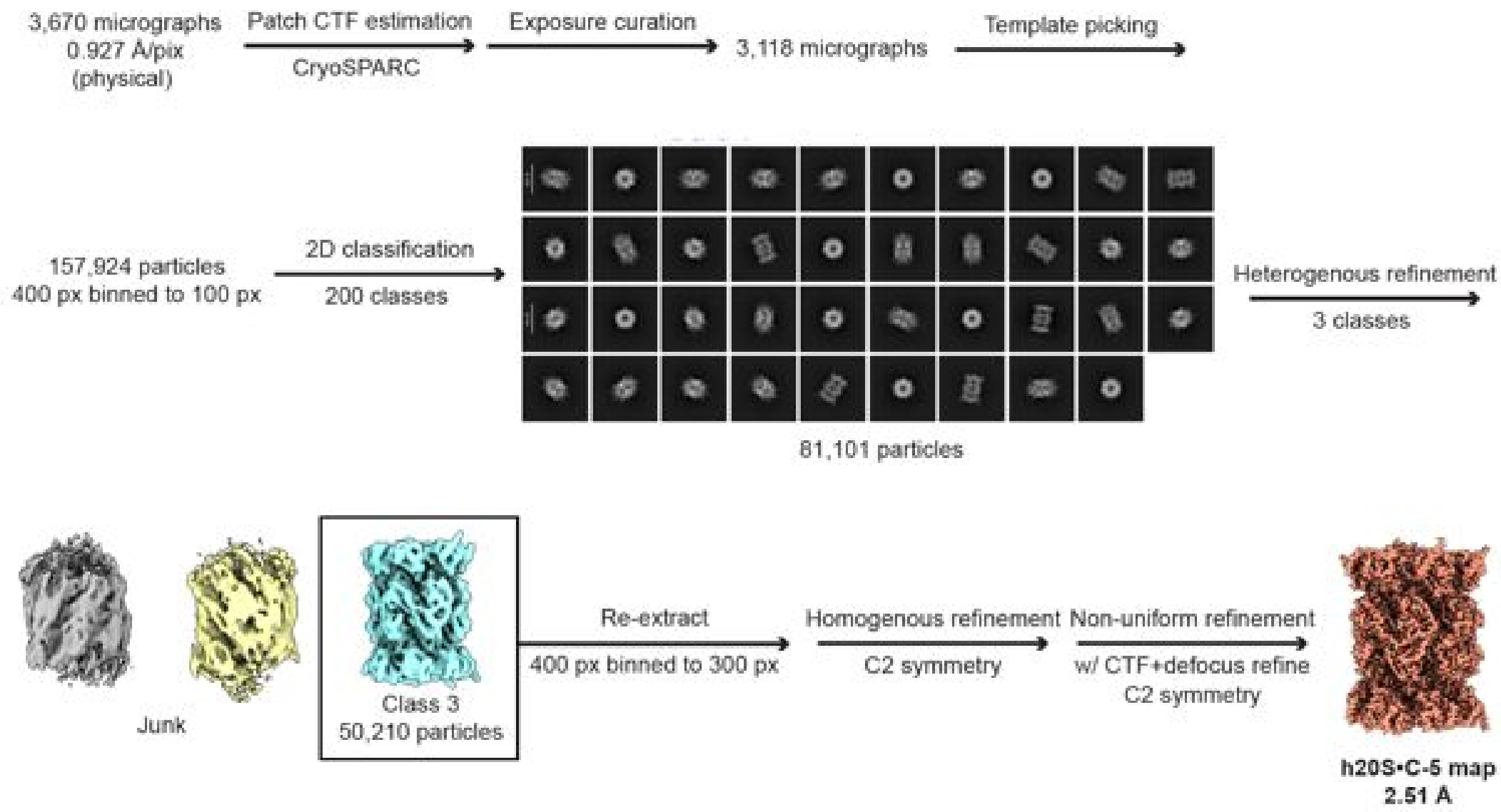

Figure S6. Cryo-EM data processing flowchart for the h20S•**C-5** complex.

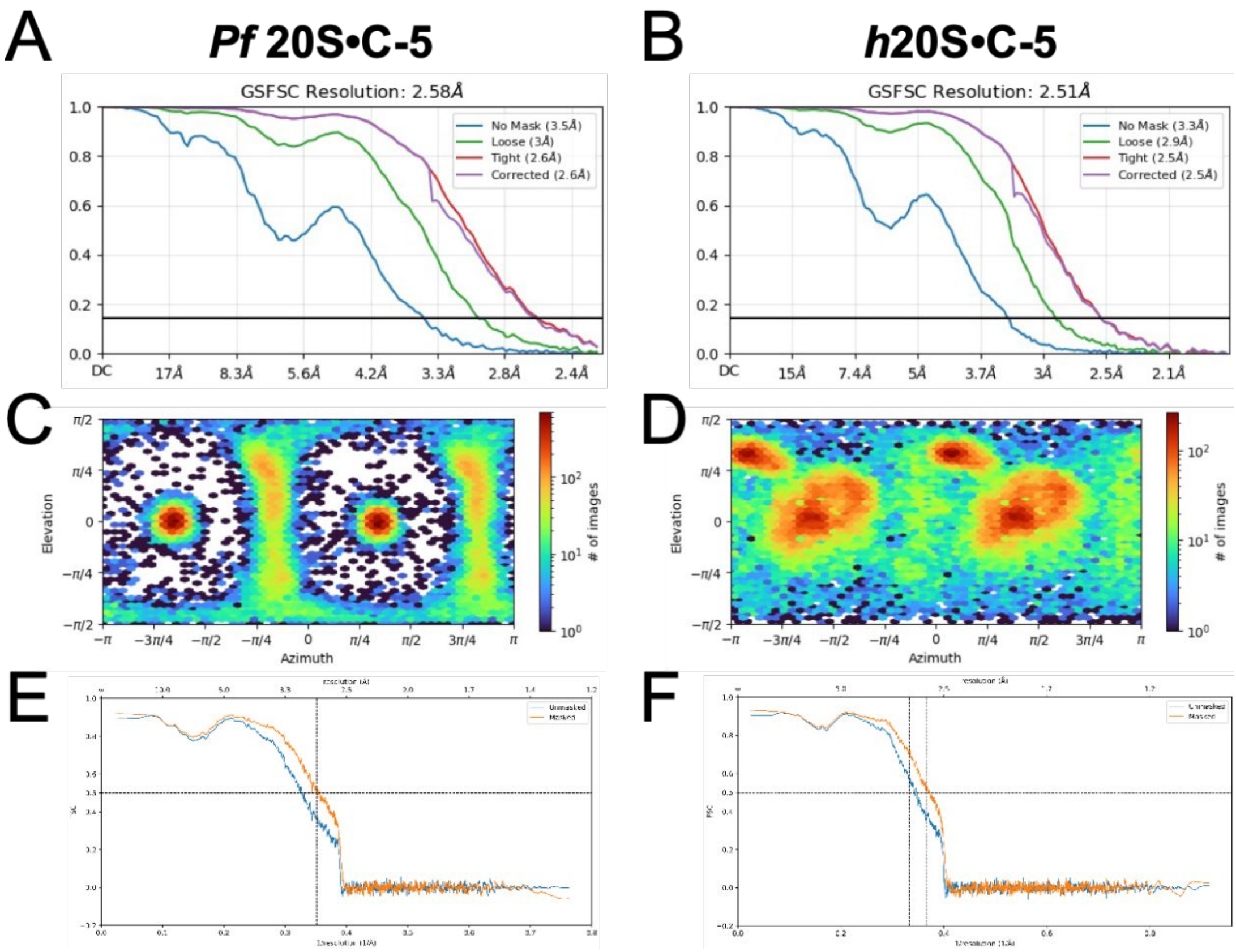

Figure S7. Cryo-EM data quality. (A-B) Fourier Shell Correlation (FSC) curves for finalized maps (C-D) Viewing direction distributions for particle stacks in finalized

maps. (E-F) Map-to model FSC curves.

UPLC-MS for Syringolin Analogs

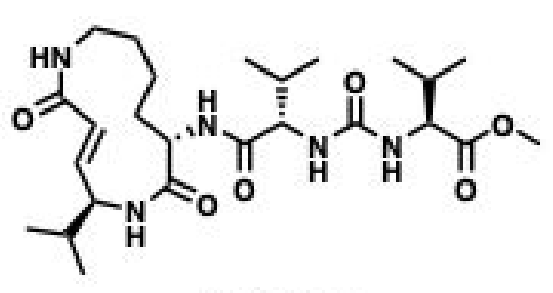

SyrB-OMe

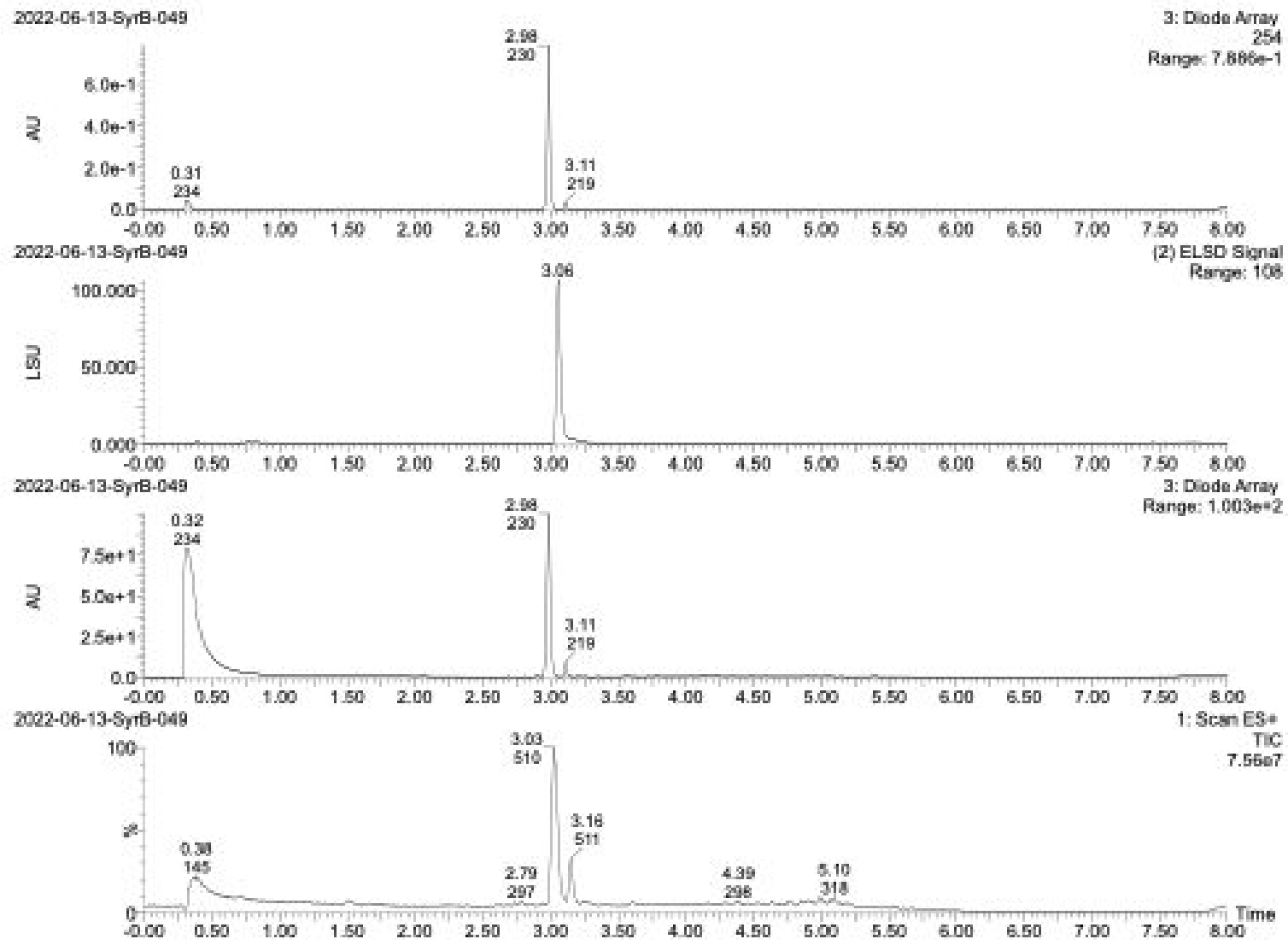

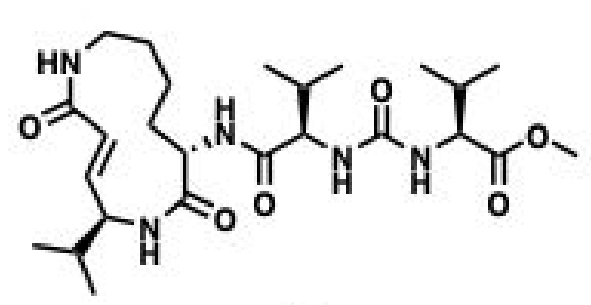

A-1

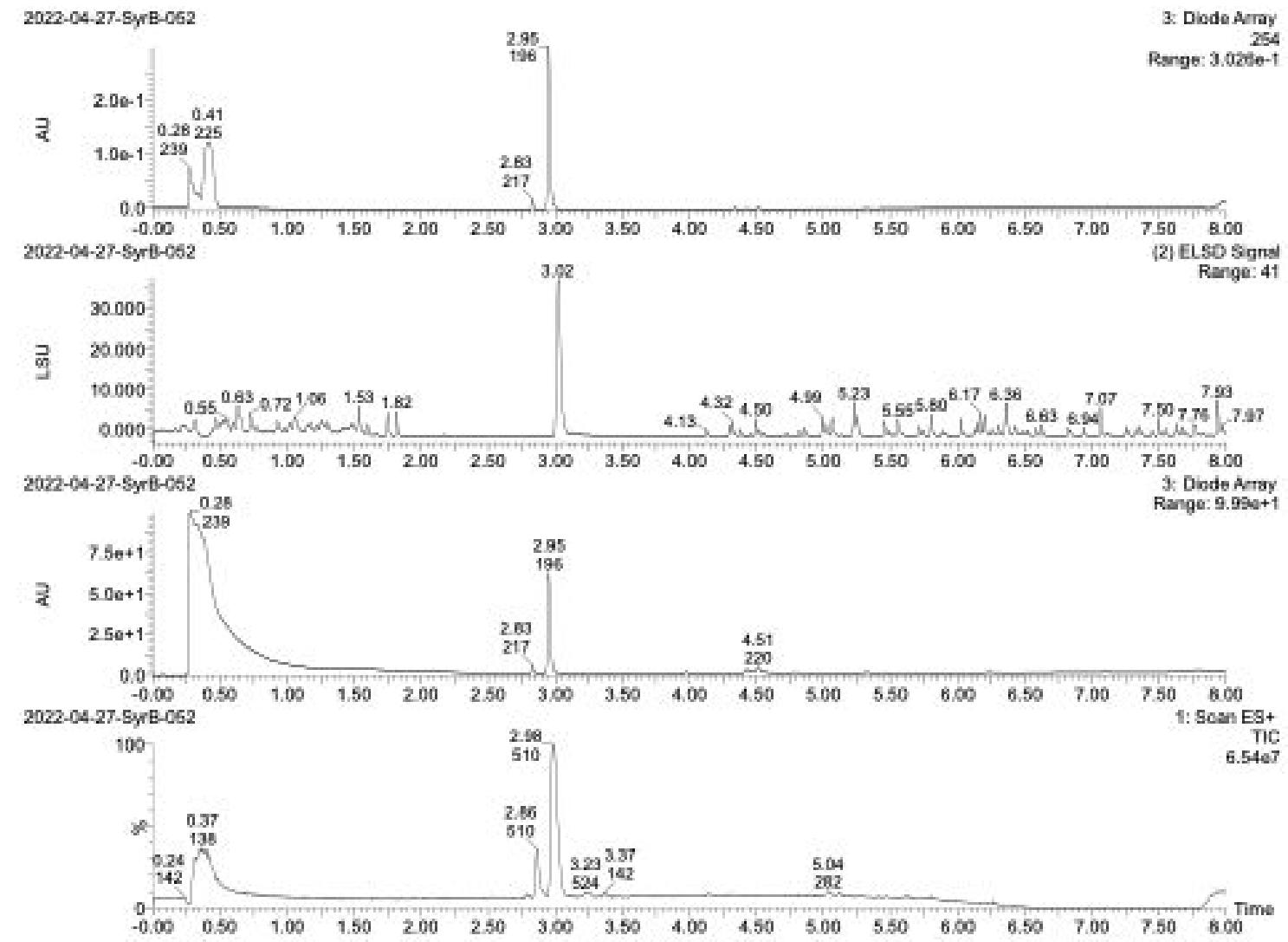

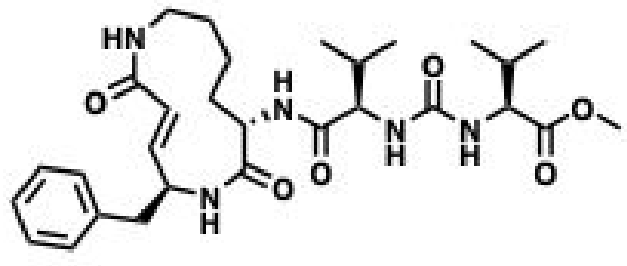

A-4

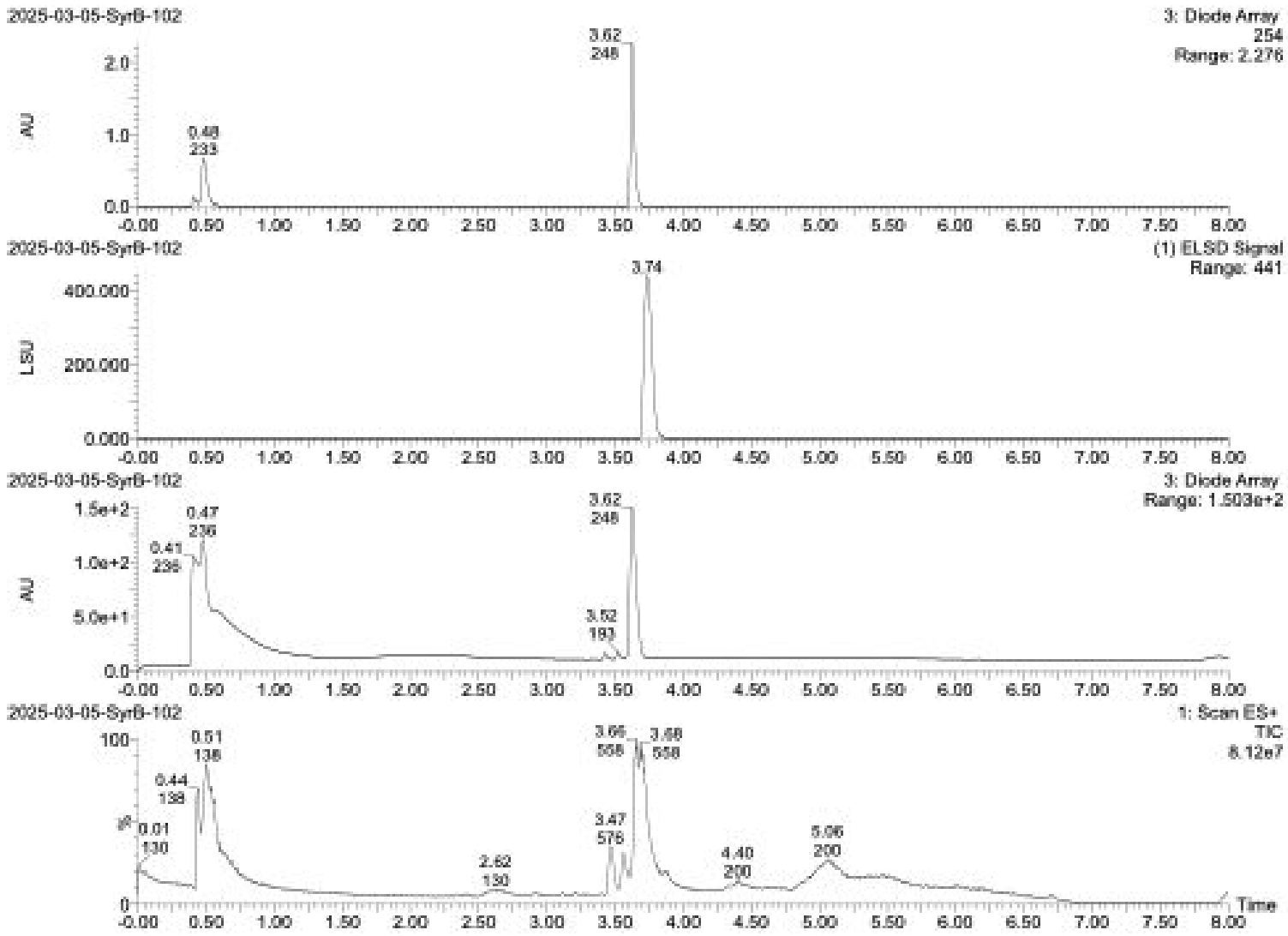
2025-03-05-SyrB-102
3: Diode Array
254
Range: 2.276
3.62
248
0.48
233
AU
(1) ELSD Signal
Range: 441
3.74
LSU
3: Diode Array
Range: 1.503e+2
0.47
236
0.41
236
3.52
193
1: Scan ES+
TIC
8.12e7
0.51
138
0.44
138
0.01
130
2.62
130
3.66
558
3.68
558
3.47
576
4.40
200
5.06
200
Time

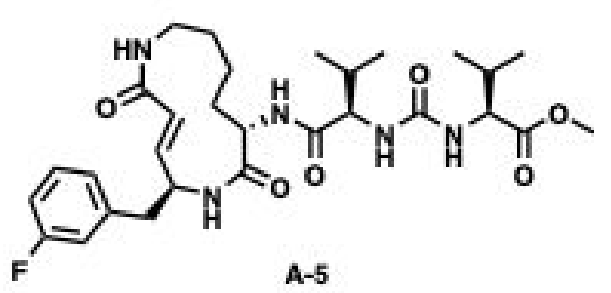

A-5

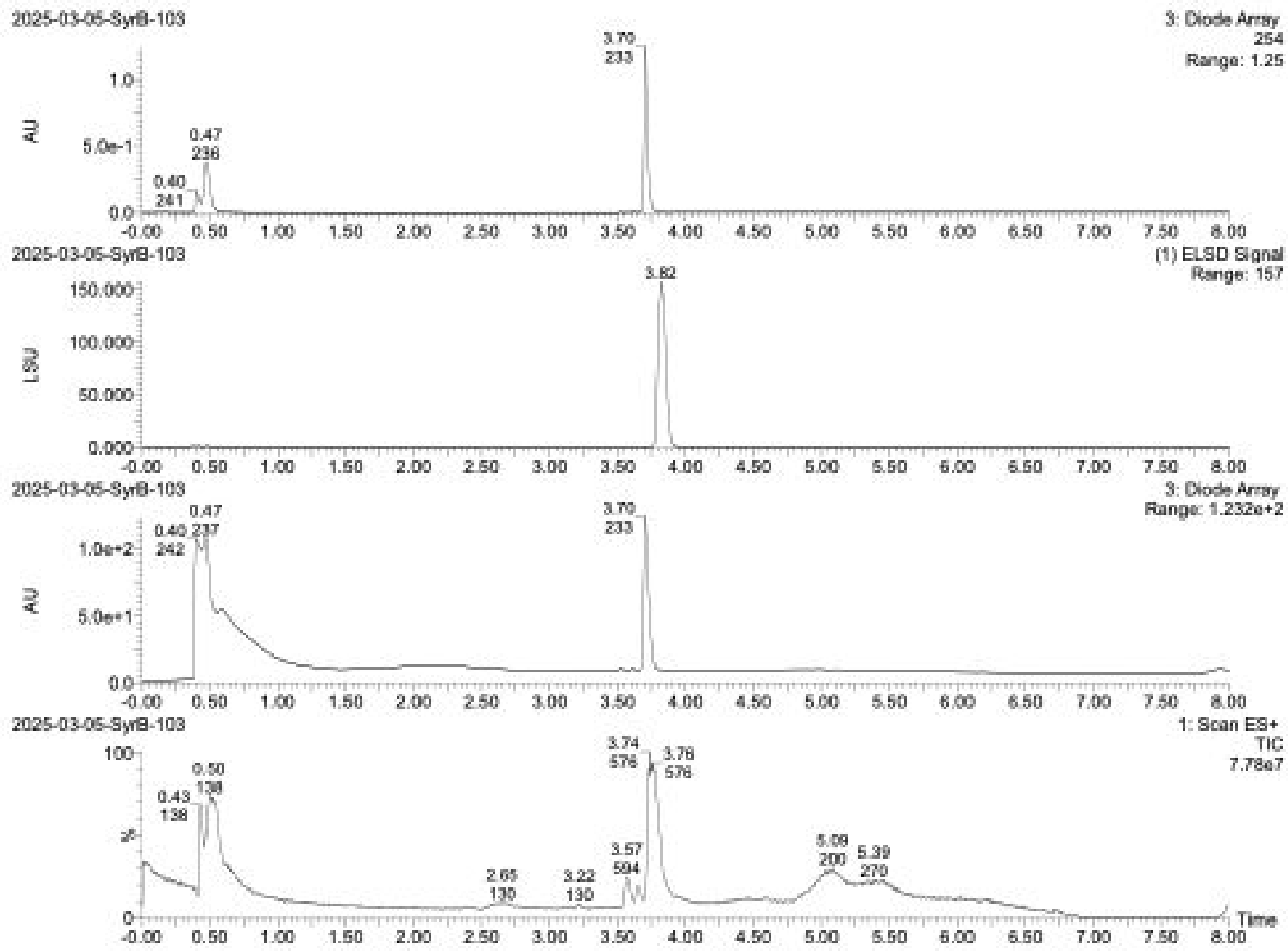
2025-03-05-SyrB-103
3: Diode Array
254
Range: 1.25
3.70
233
0.47
236
0.40
241
AU
(1) ELSD Signal
Range: 157
3.82
LSU
3: Diode Array
Range: 1.232e+2
0.40
242
0.47
237
1: Scan ES+
TIC
7.78e7
0.50
138
0.43
138
2.65
130
3.22
130
3.74
576
3.76
576
3.57
594
5.09
200
5.39
270
Time

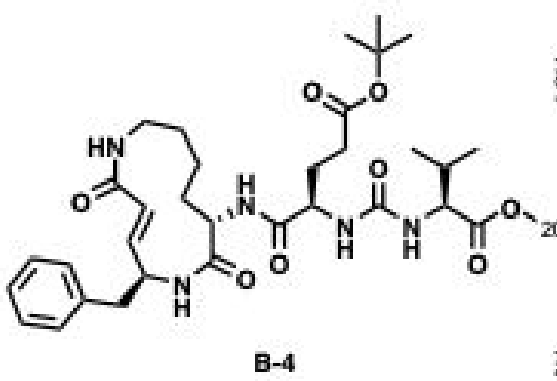

B-4

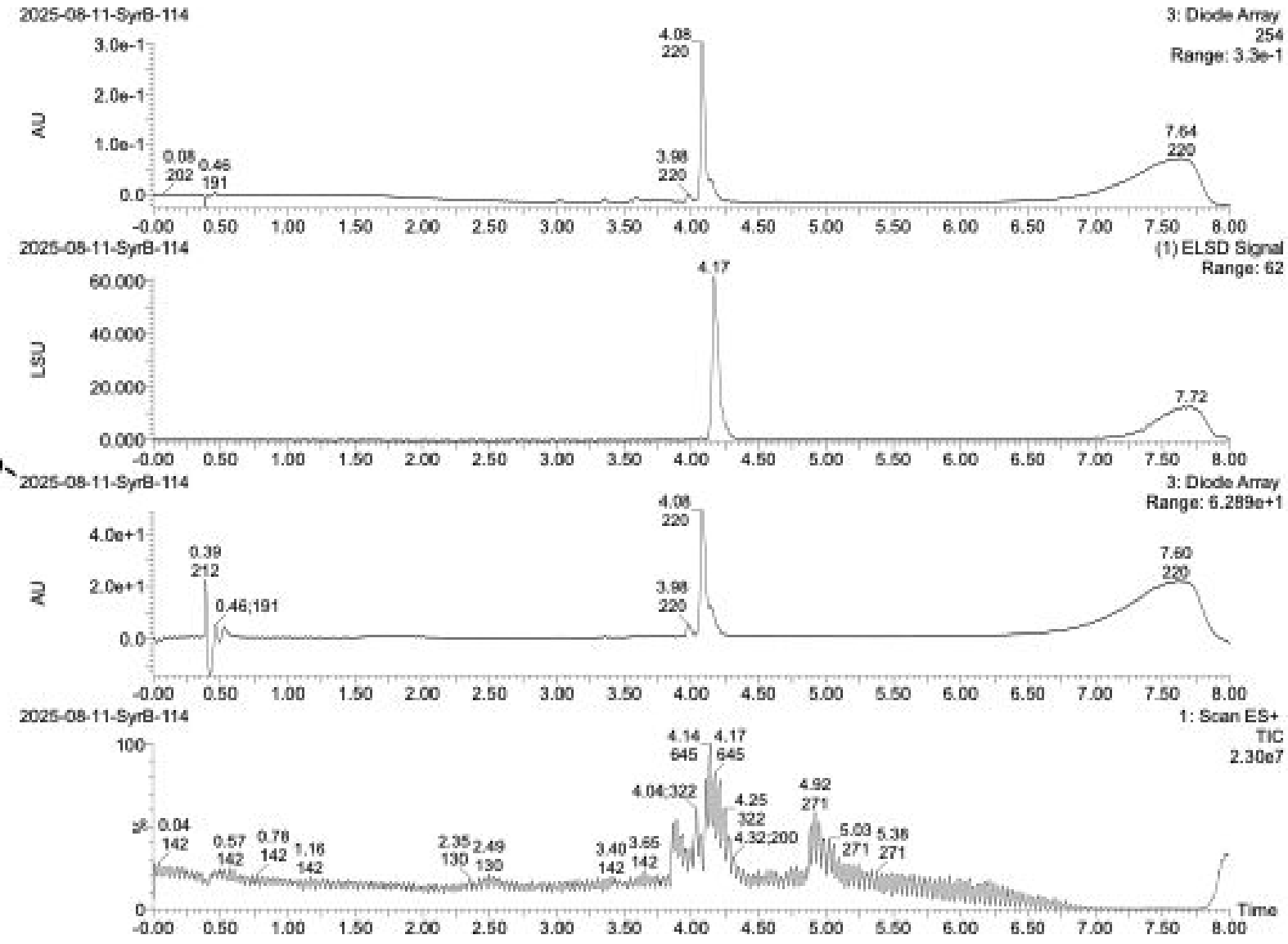
2025-08-11-SyrB-114
3: Diode Array
254
Range: 3.3e-1
AU
4.08
220
7.64
220
(1) ELSD Signal
Range: 62
LSU
4.17
7.72
3: Diode Array
Range: 6.289e+1
7.60
220
1: Scan ES+
TIC
2.30e7
4.14
645
4.17
645
4.92
271
Time

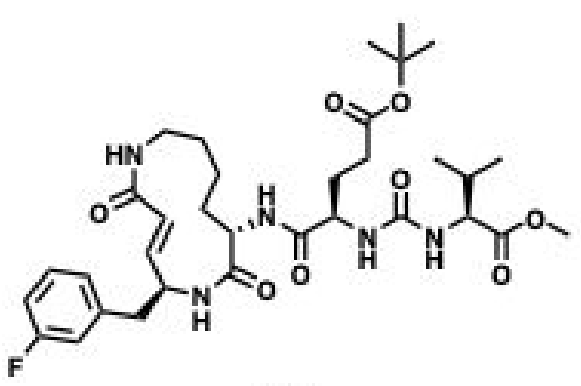

B-5

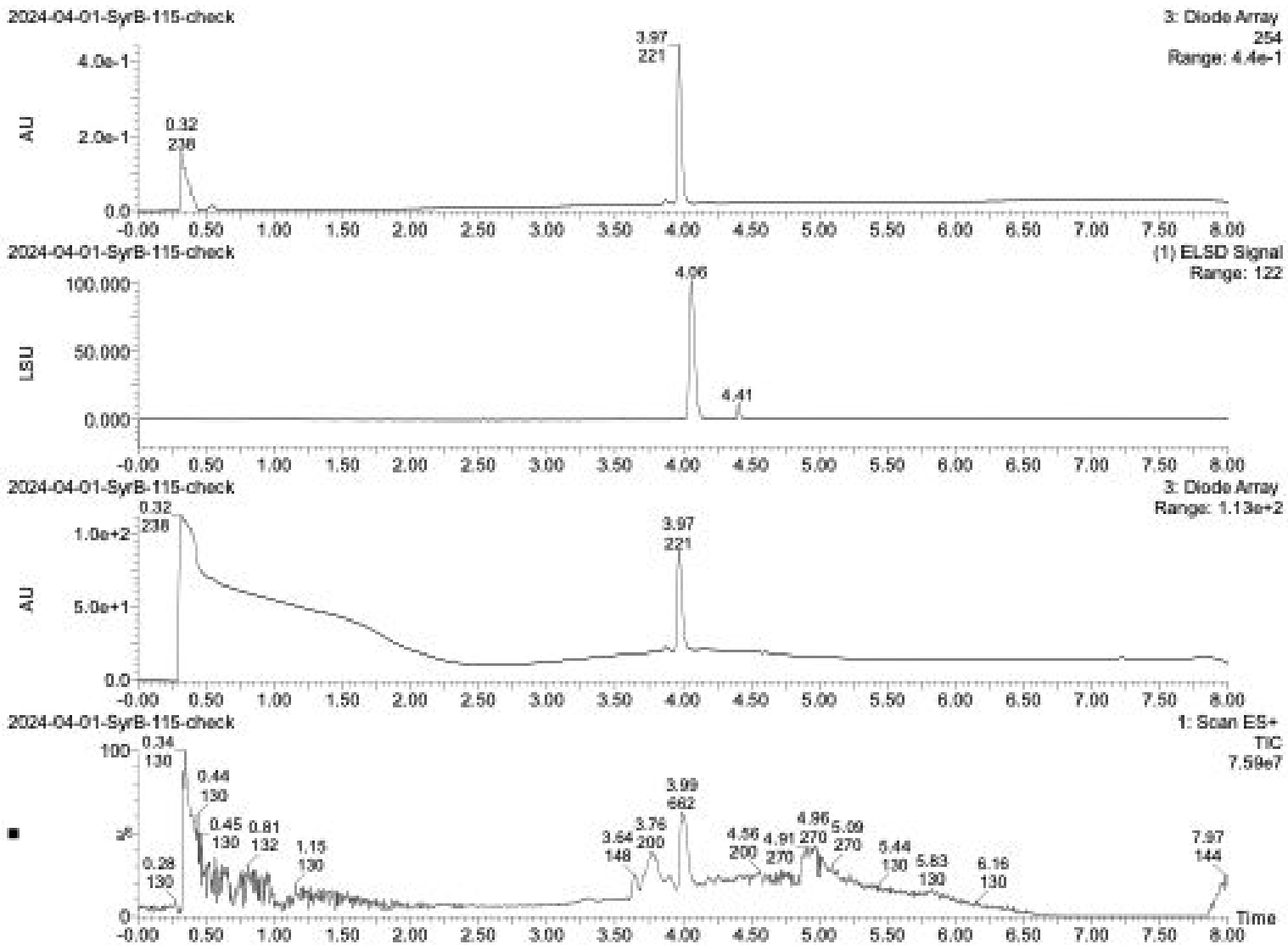
2024-04-01-SyrB-115-check
3: Diode Array
254
Range: 4.4e-1
AU
3.97
221
(1) ELSD Signal
Range: 122
LSU
4.06
4.41
3: Diode Array
Range: 1.13e+2
1: Scan ES+
TIC
7.59e7
3.99
662
Time

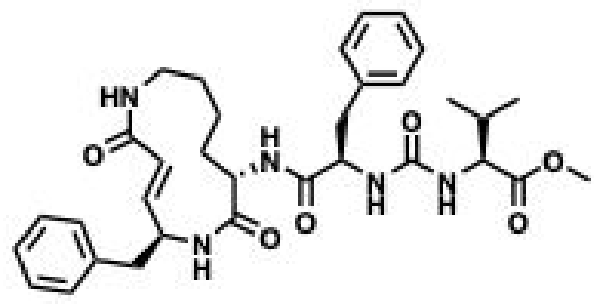
HN
O
NH
H
N
O
O
N
H
N
H
O
O
O

C-4

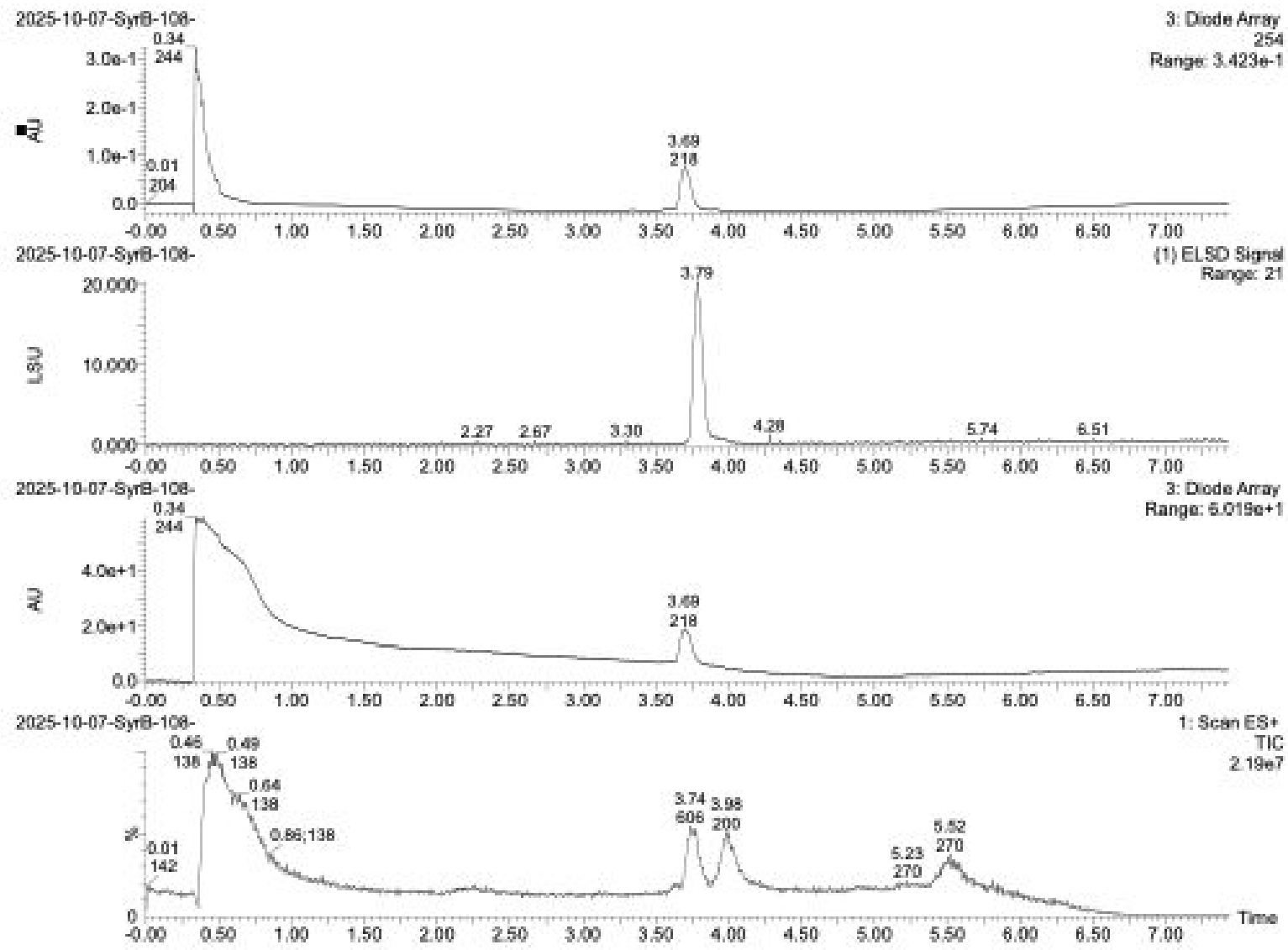
2025-10-07-SyrB-108-
3: Diode Array
254
Range: 3.423e-1
AU
0.34
244
0.01
204
3.69
218
2025-10-07-SyrB-108-
(1) ELSD Signal
Range: 21
LSU
3.79
2.27
2.67
3.30
4.28
5.74
6.51
2025-10-07-SyrB-108-
3: Diode Array
Range: 6.019e+1
AU
0.34
244
3.69
218
2025-10-07-SyrB-108-
1: Scan ES+
TIC
2.19e7
0.46
138
0.49
138
0.64
138
0.86;138
0.01
142
3.74
606
3.98
200
5.23
270
5.52
270
Time

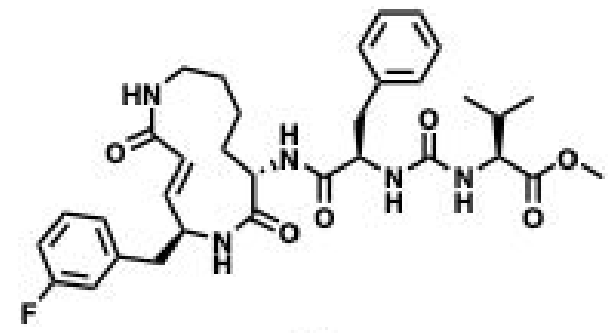
HN
O
NH
H
N
O
O
N
H
N
H
O
O
O
F

C-5

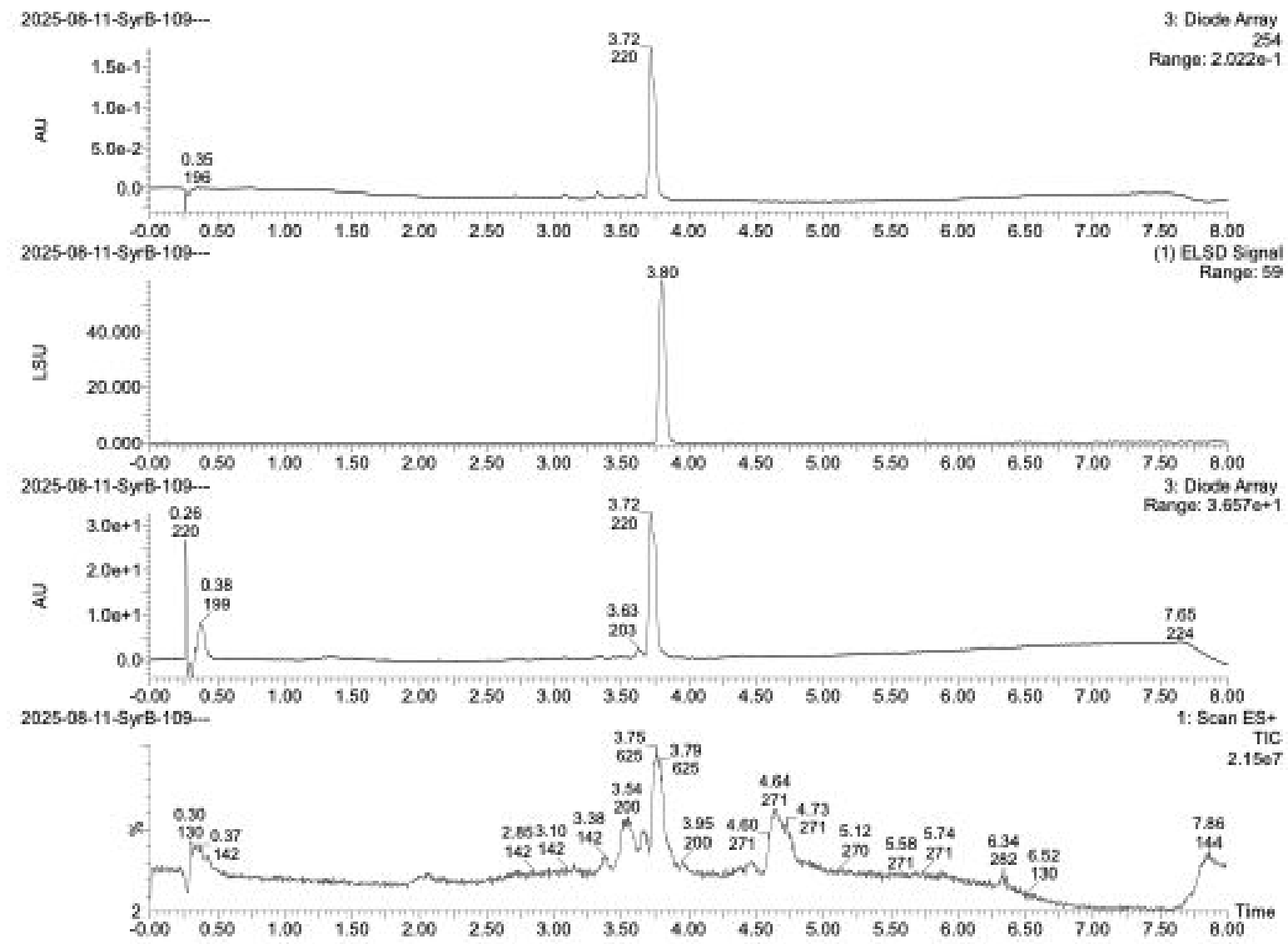
2025-08-11-SyrB-109---
3: Diode Array
254
Range: 2.022e-1
AU
3.72
220
0.35
196
2025-08-11-SyrB-109---
(1) ELSD Signal
Range: 59
LSU
3.80
2025-08-11-SyrB-109---
3: Diode Array
Range: 3.657e+1
AU
0.26
220
0.38
199
3.63
203
3.72
220
7.65
224
2025-08-11-SyrB-109---
1: Scan ES+
TIC
2.15e7
0.30
130
0.37
142
2.85
142
3.10
142
3.38
142
3.54
200
3.75
625
3.79
625
3.95
200
4.60
271
4.64
271
4.73
271
5.12
270
5.58
271
5.74
271
6.34
282
6.52
130
7.86
144
Time

^{1}H and ^{13}C NMR Spectrum

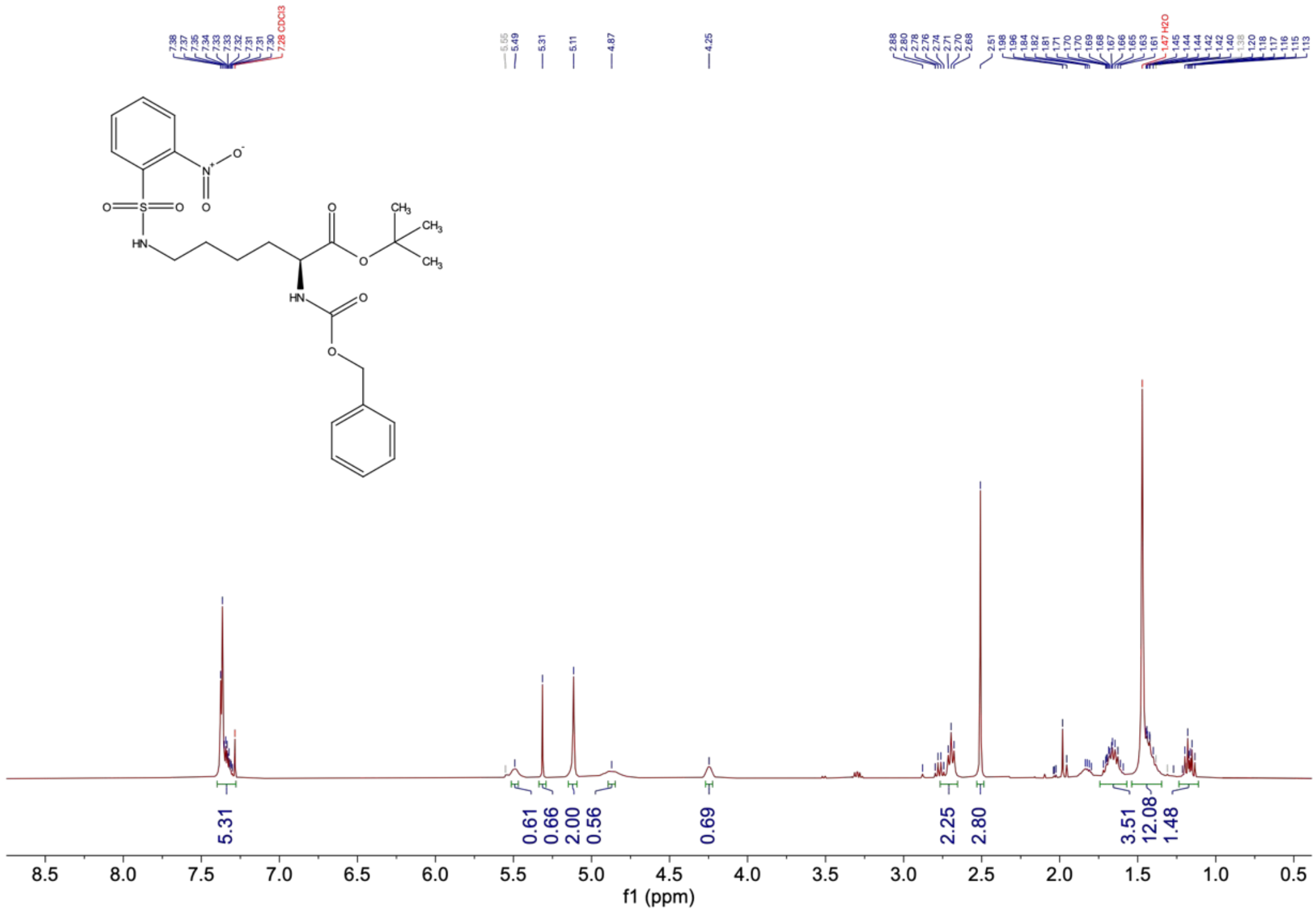

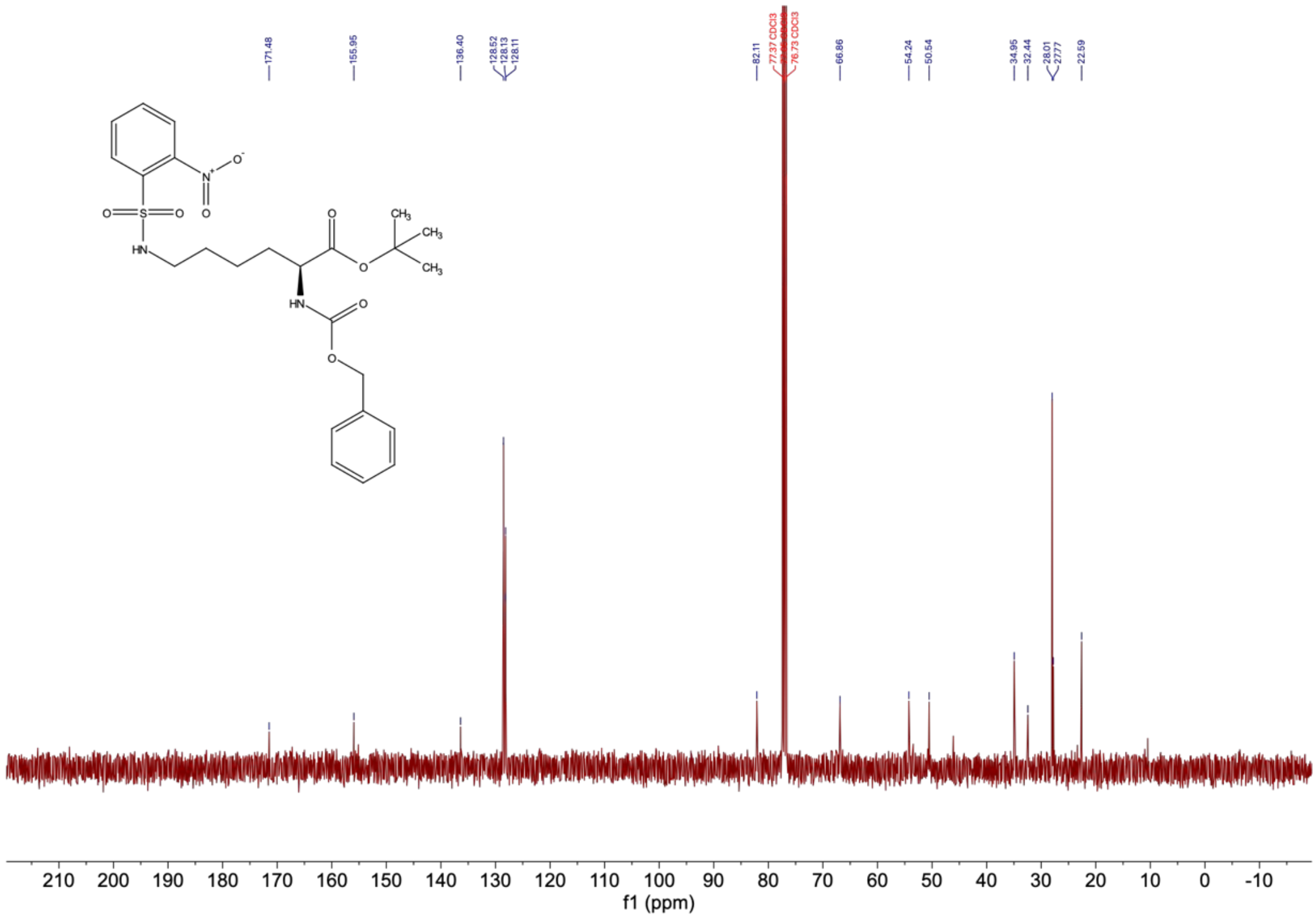

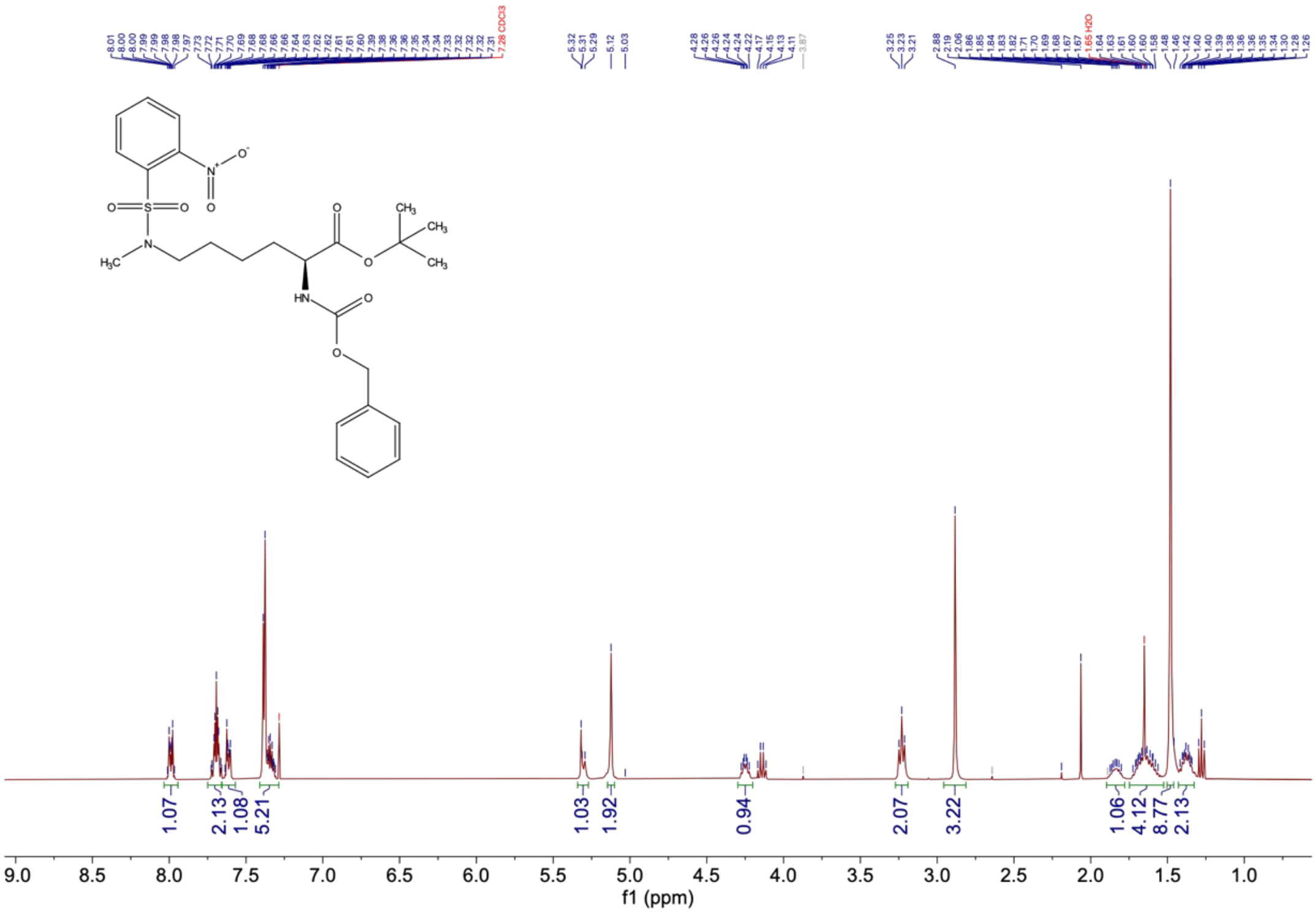

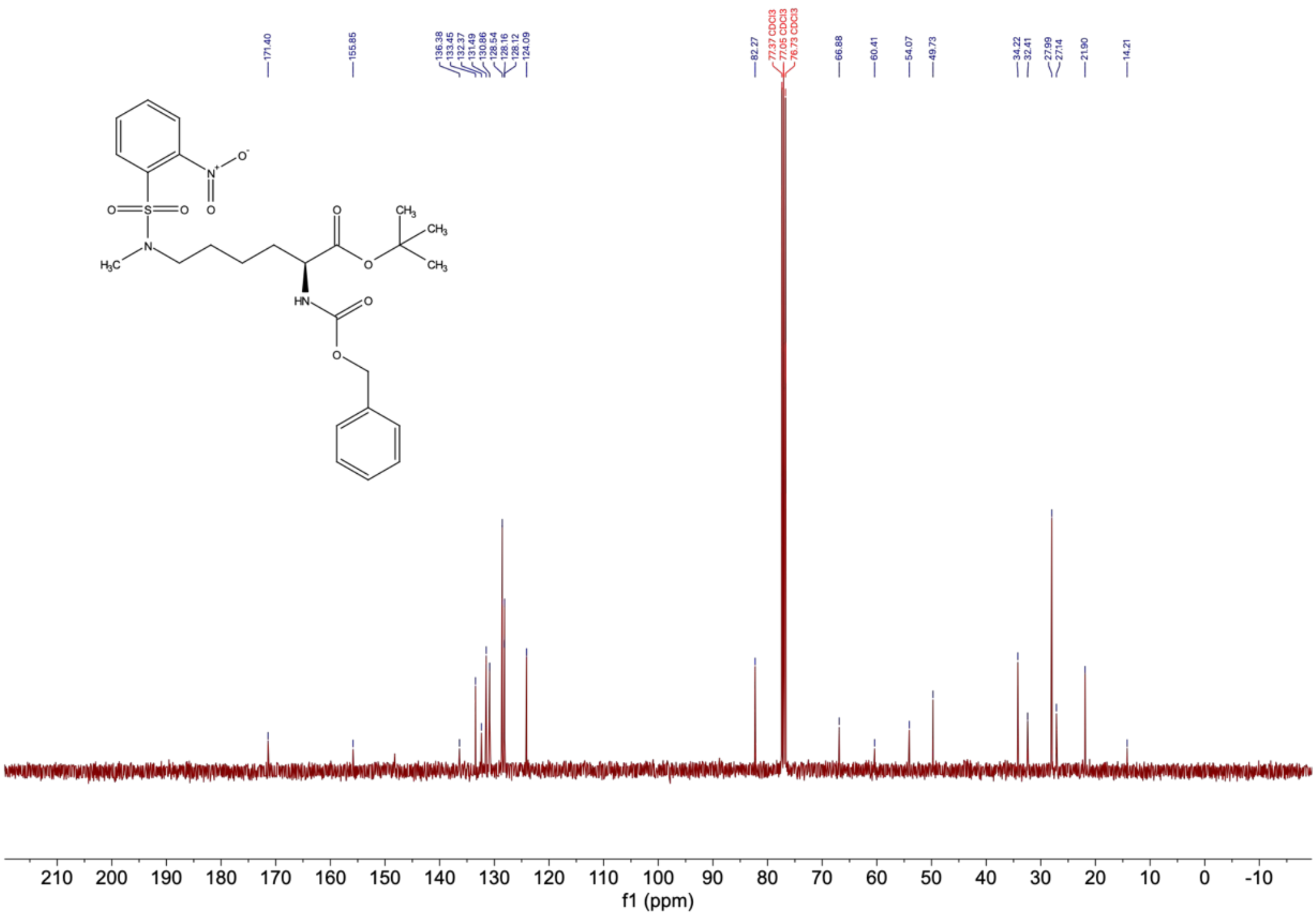

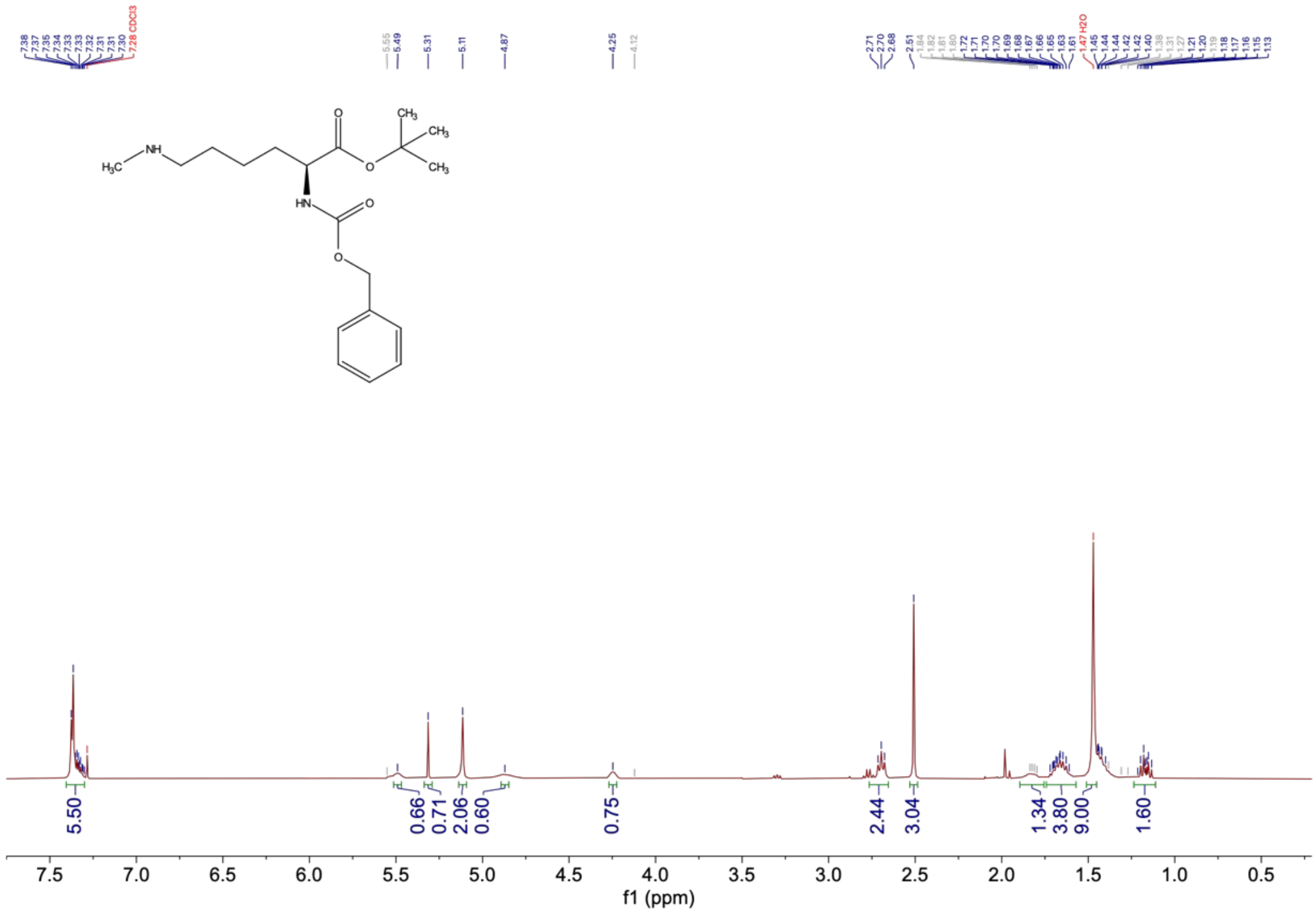

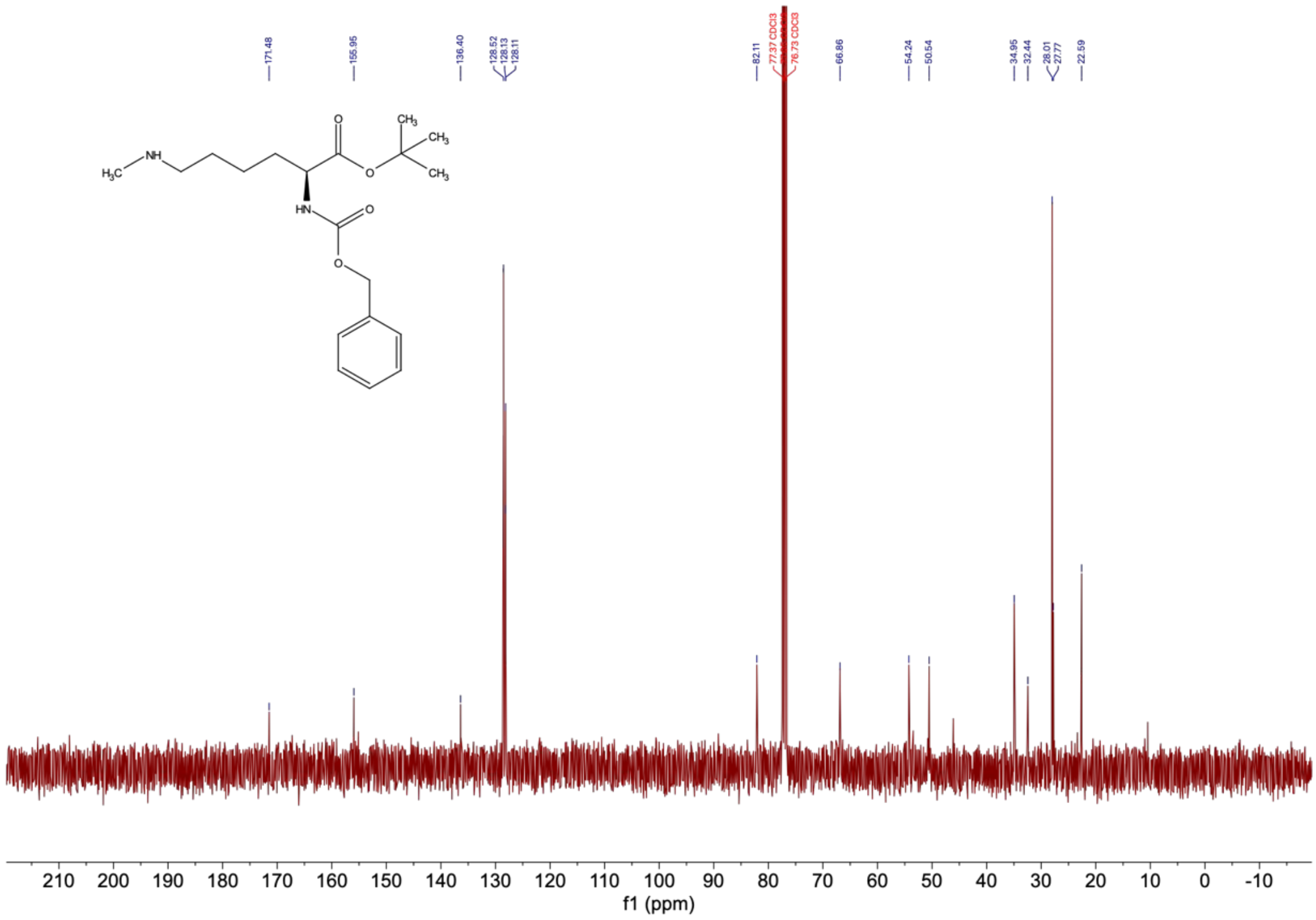

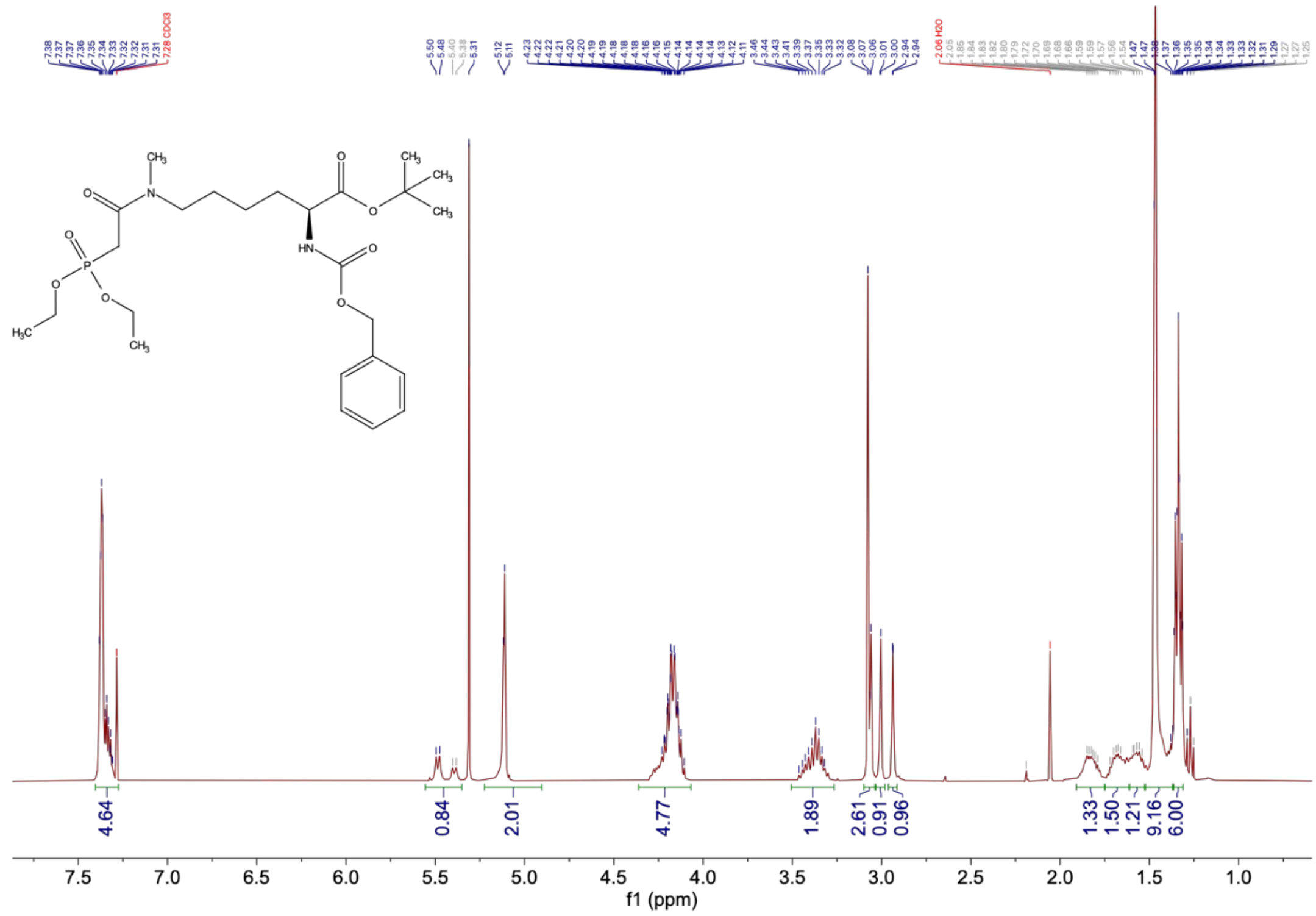

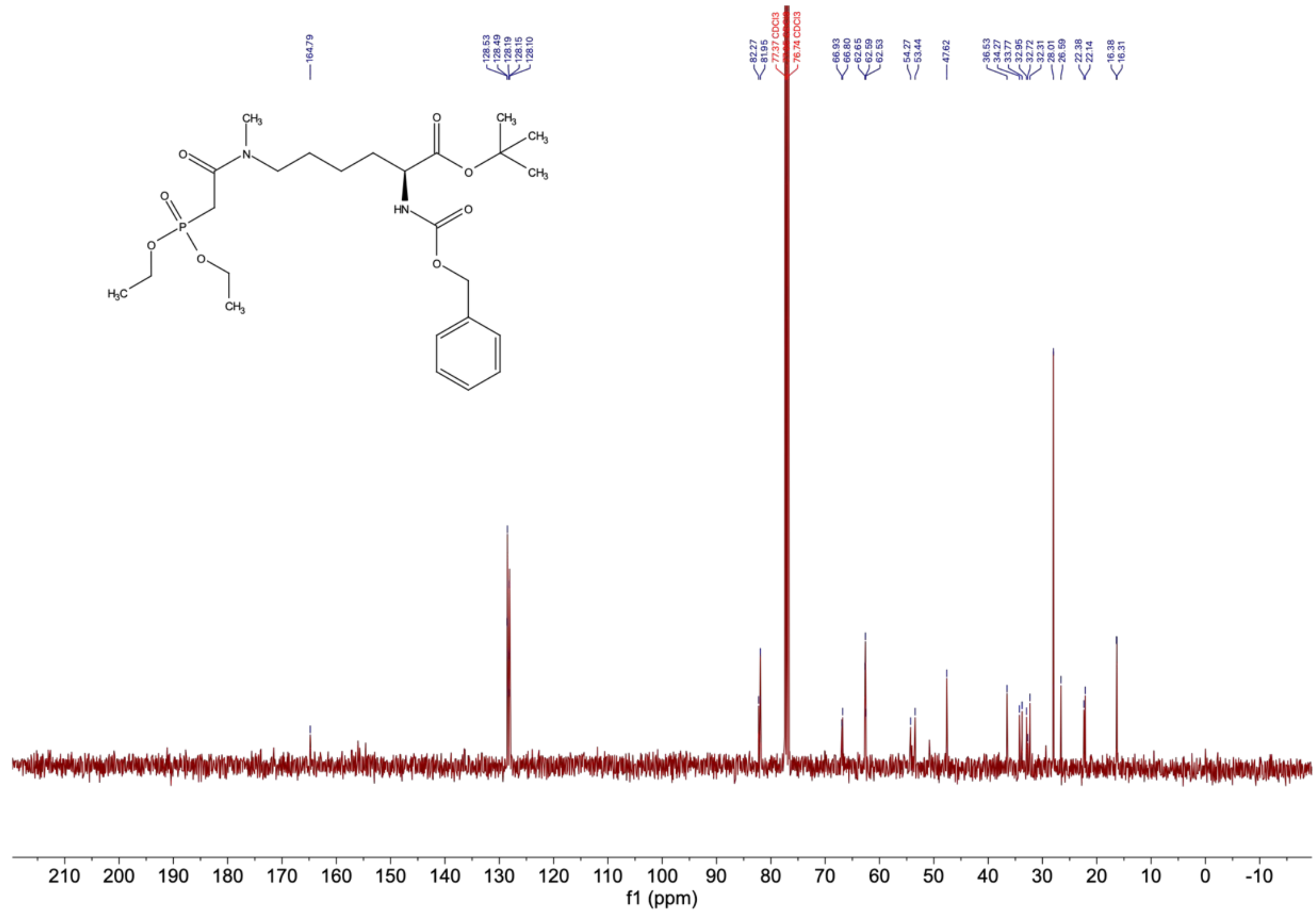

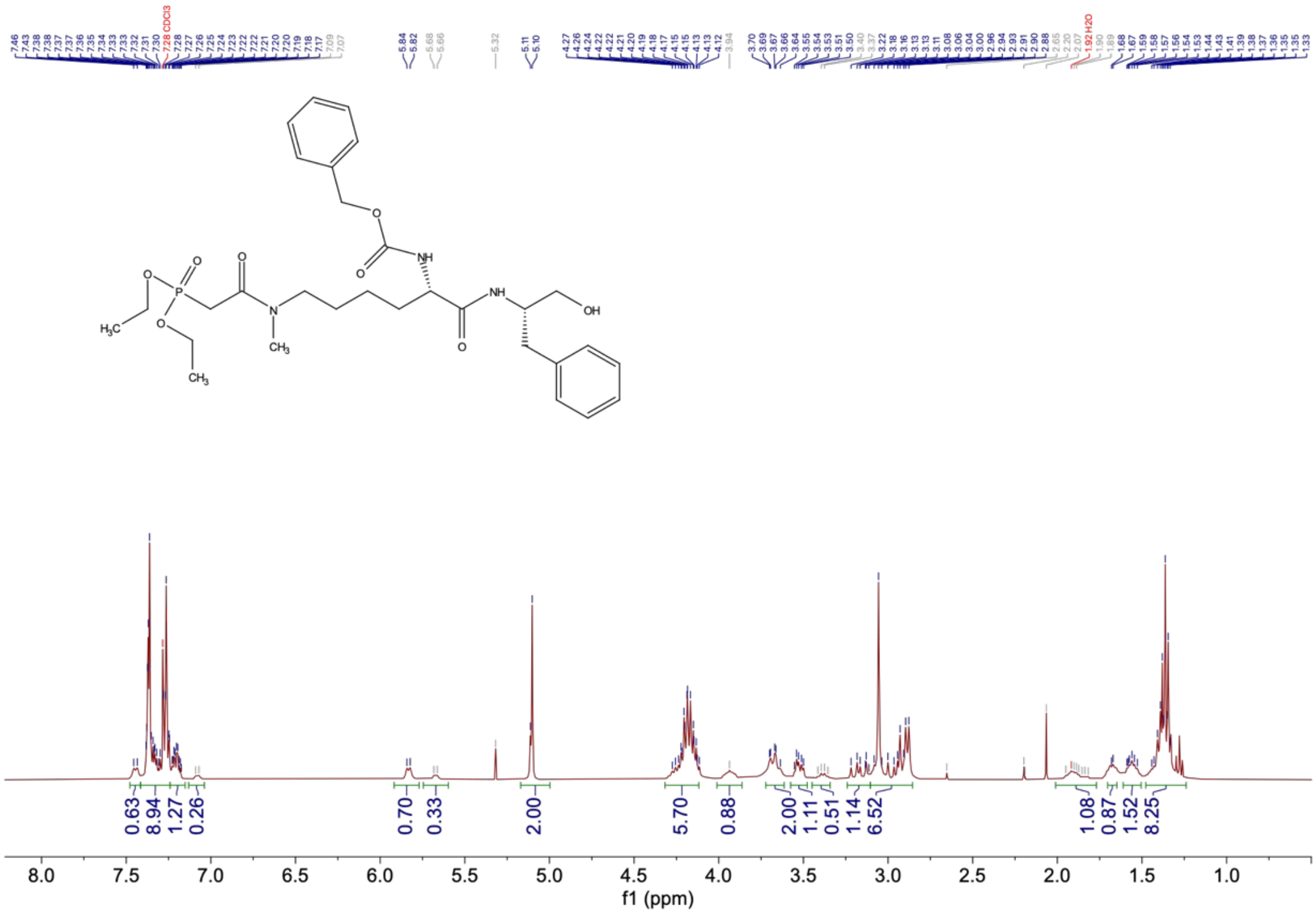

NH
O
O
P
N
CH3
H3C
CH3
OH
0.63
8.94
1.27
0.26
0.70
0.33
2.00
5.70
0.88
2.00
1.11
0.51
1.14
6.52
1.08
0.87
1.52
8.25
7.28 CDCl3
1.92 H2O
f1 (ppm)

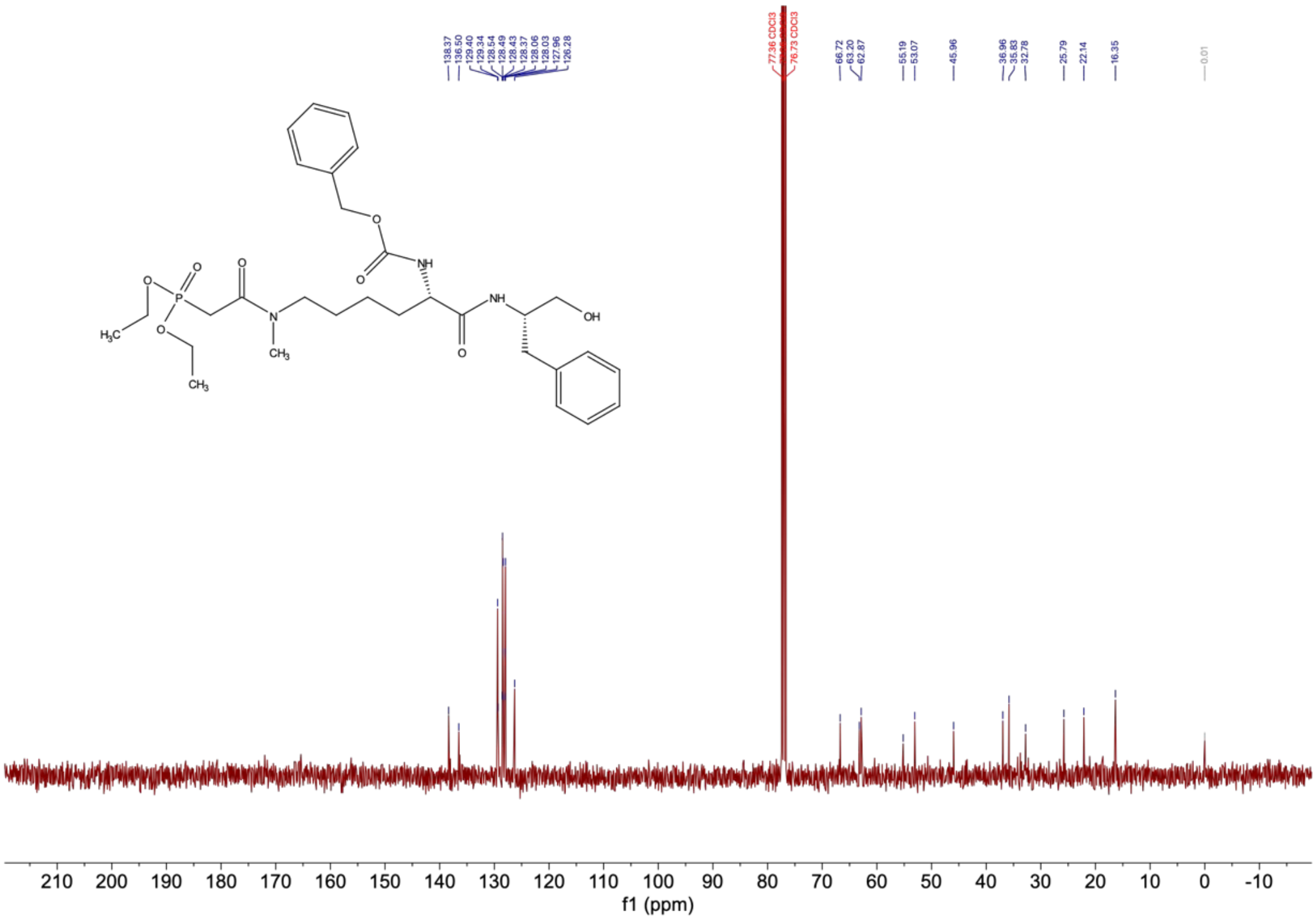

NH
O
O
P
N
CH3
H3C
CH3
OH
77.36 CDCl3
76.73 CDCl3
f1 (ppm)

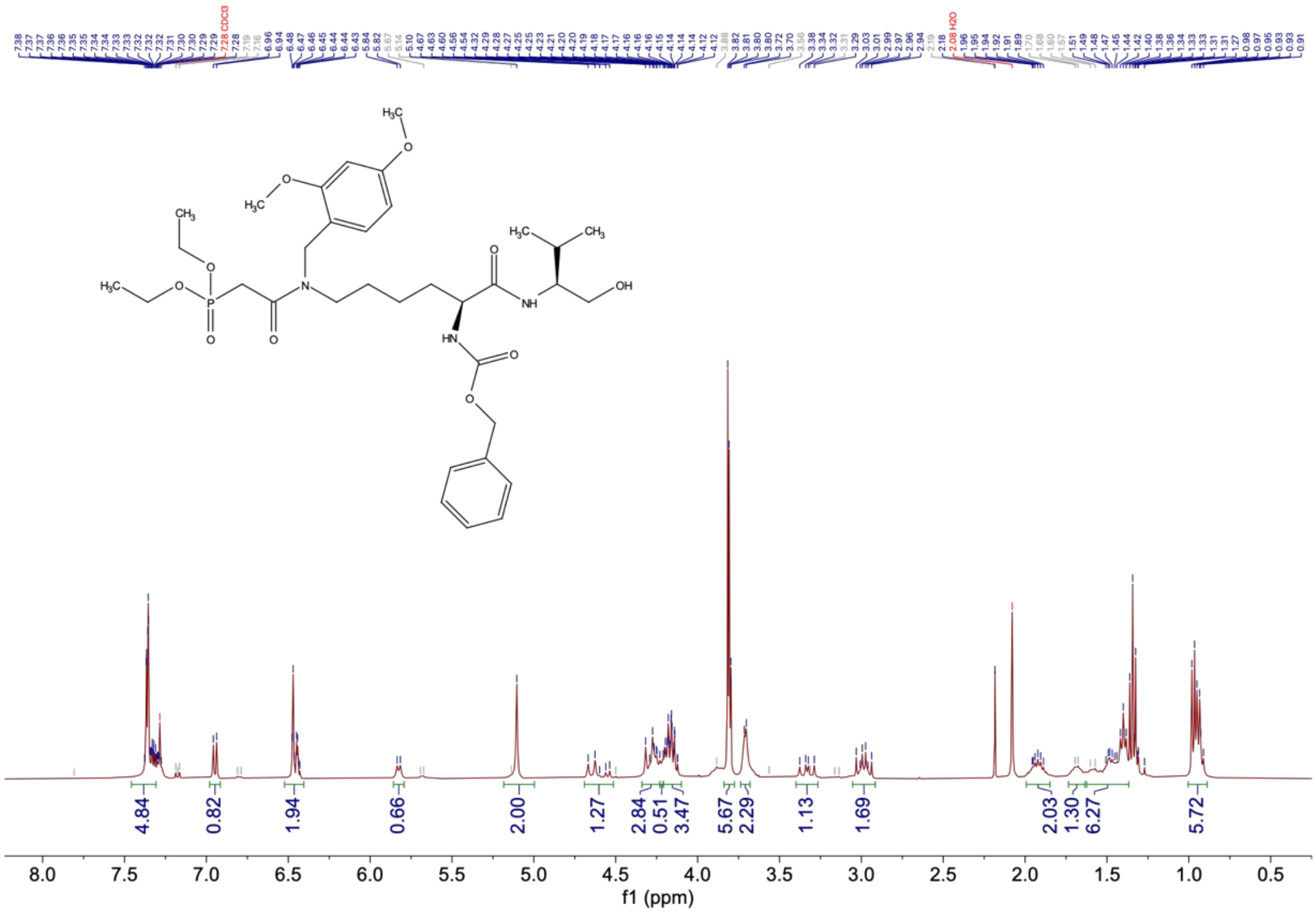

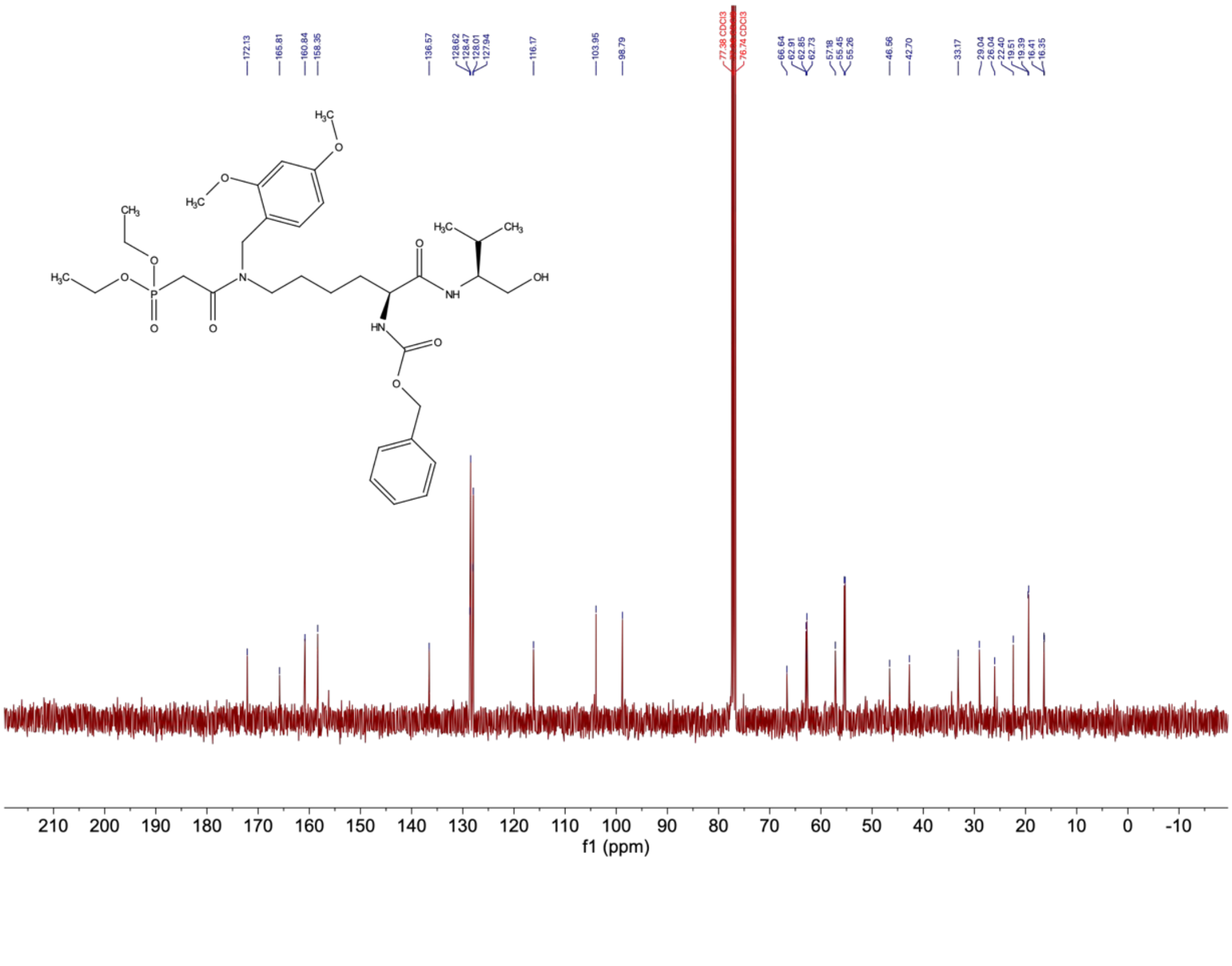

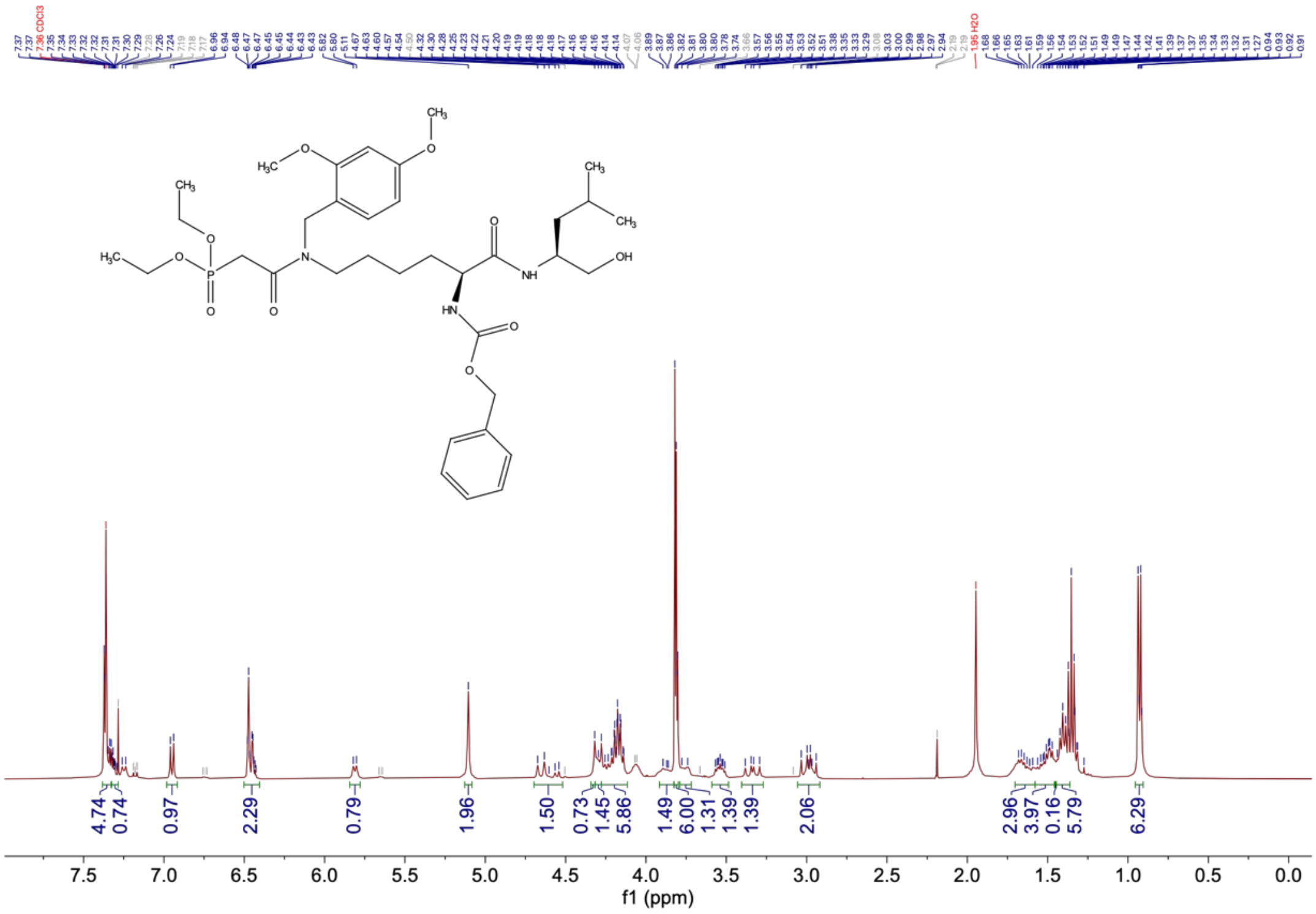
4.74
0.74
0.97
2.29
0.79
1.96
1.50
0.73
1.45
5.86
1.49
6.00
1.31
1.39
1.39
2.06
2.96
3.97
0.16
5.79
6.29
7.5 7.0 6.5 6.0 5.5 5.0 4.5 4.0 3.5 3.0 2.5 2.0 1.5 1.0 0.5 0.0
f1 (ppm)

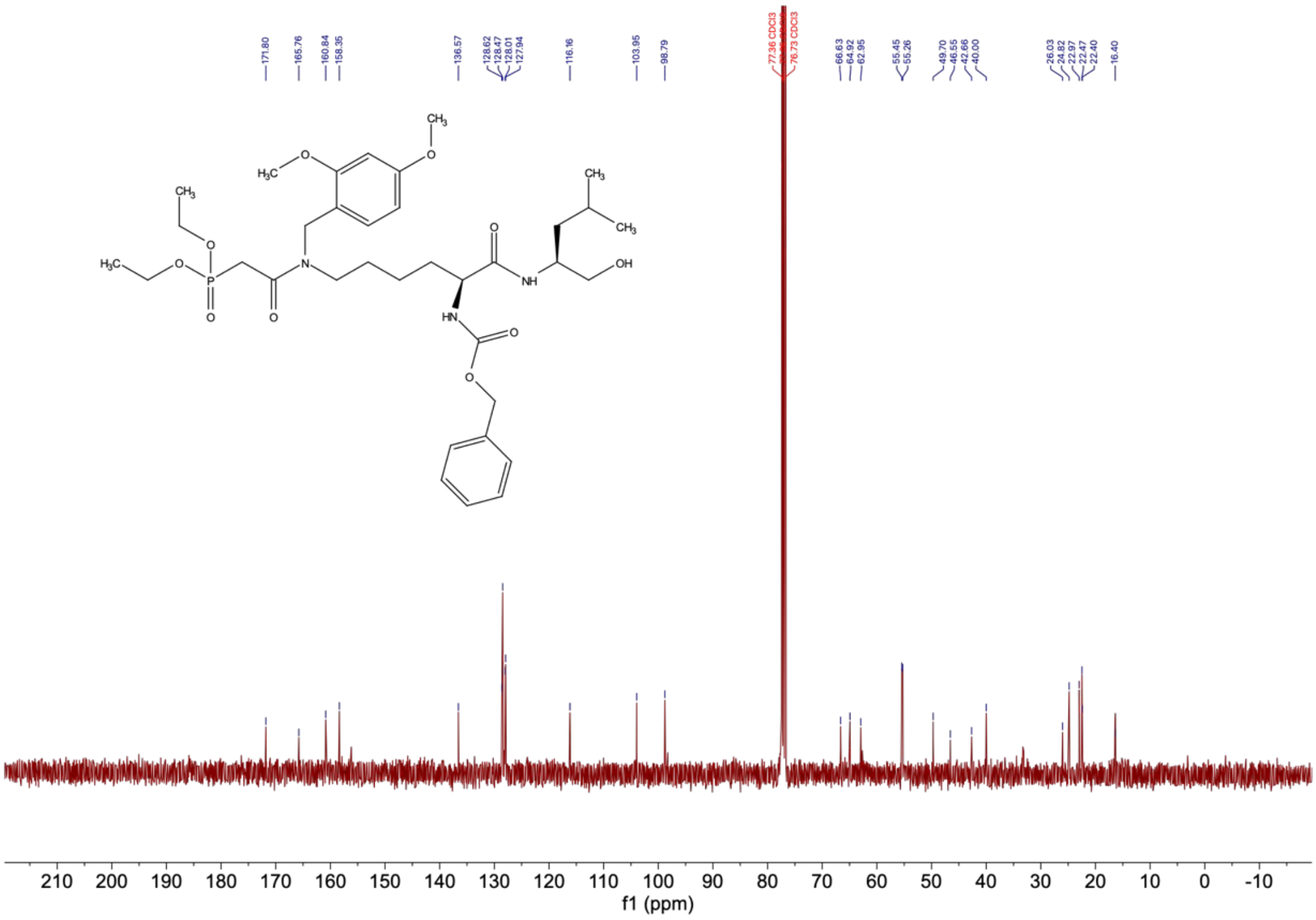
171.80
165.76
160.84
158.35
136.57
128.62
128.47
128.01
127.94
116.16
103.95
98.79
77.36 CDCl3
76.73 CDCl3
66.63
64.92
62.95
55.45
55.26
49.70
46.55
42.66
40.00
26.03
24.82
22.97
22.47
22.40
16.40
210 200 190 180 170 160 150 140 130 120 110 100 90 80 70 60 50 40 30 20 10 0 -10
f1 (ppm)

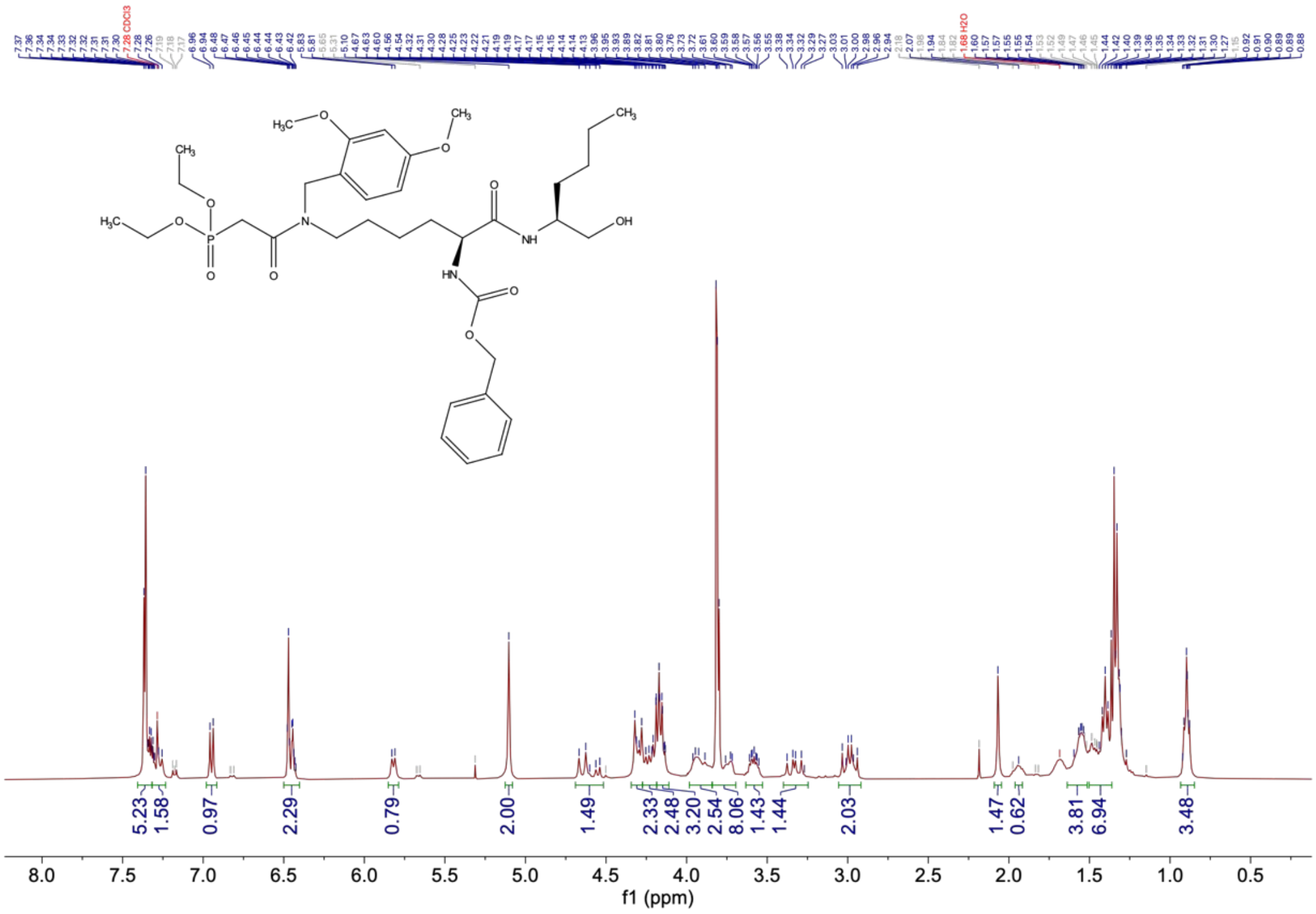

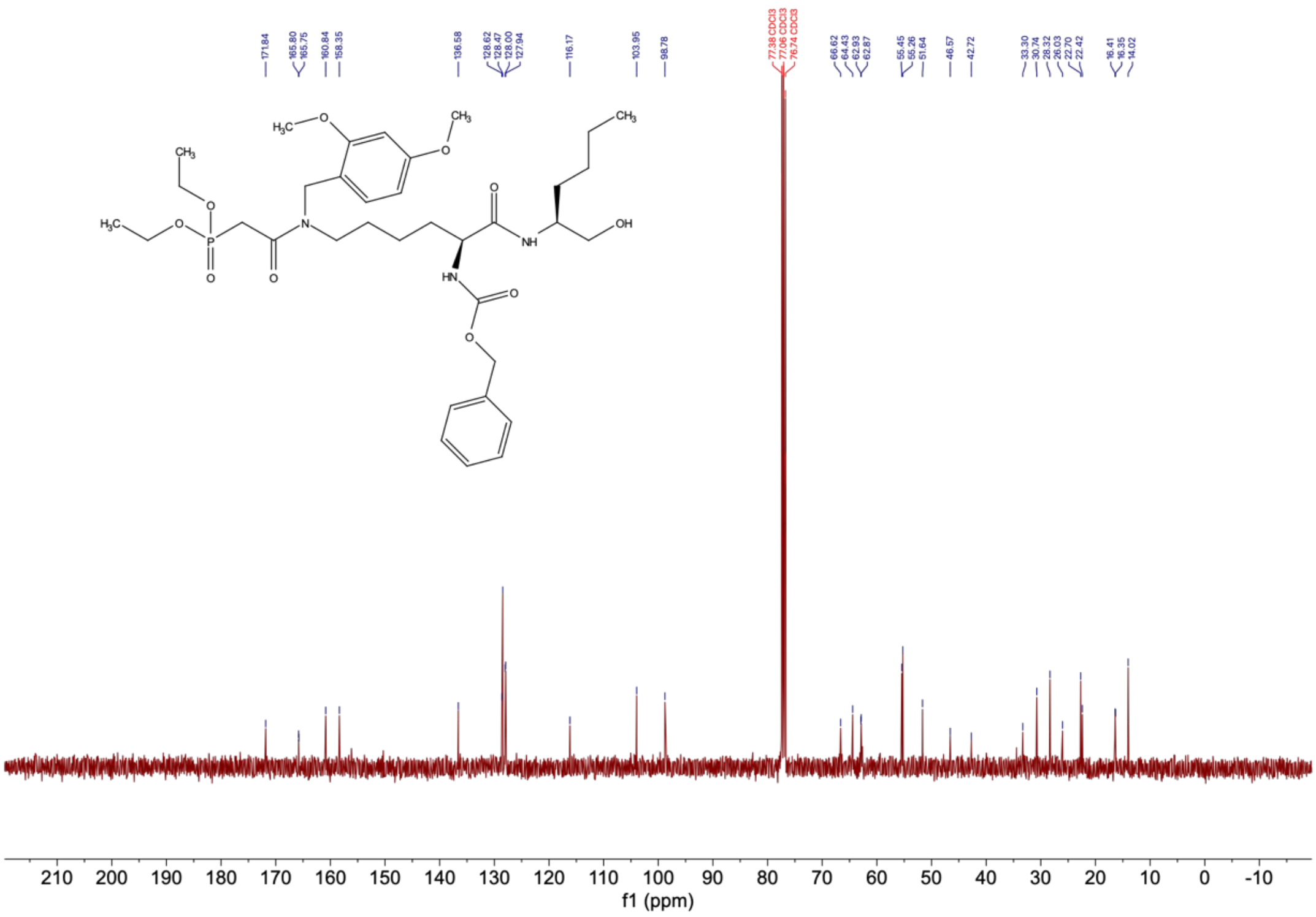

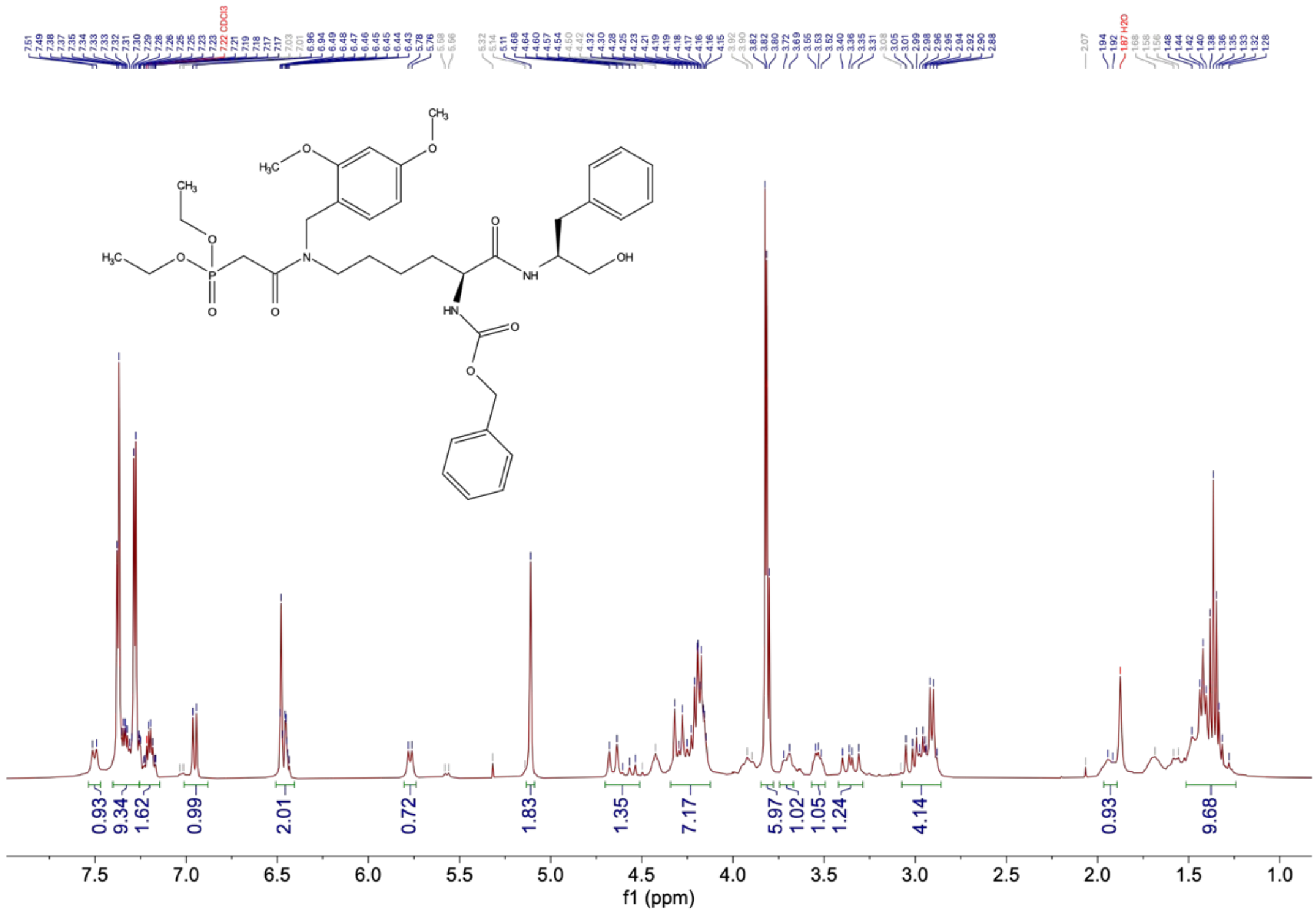

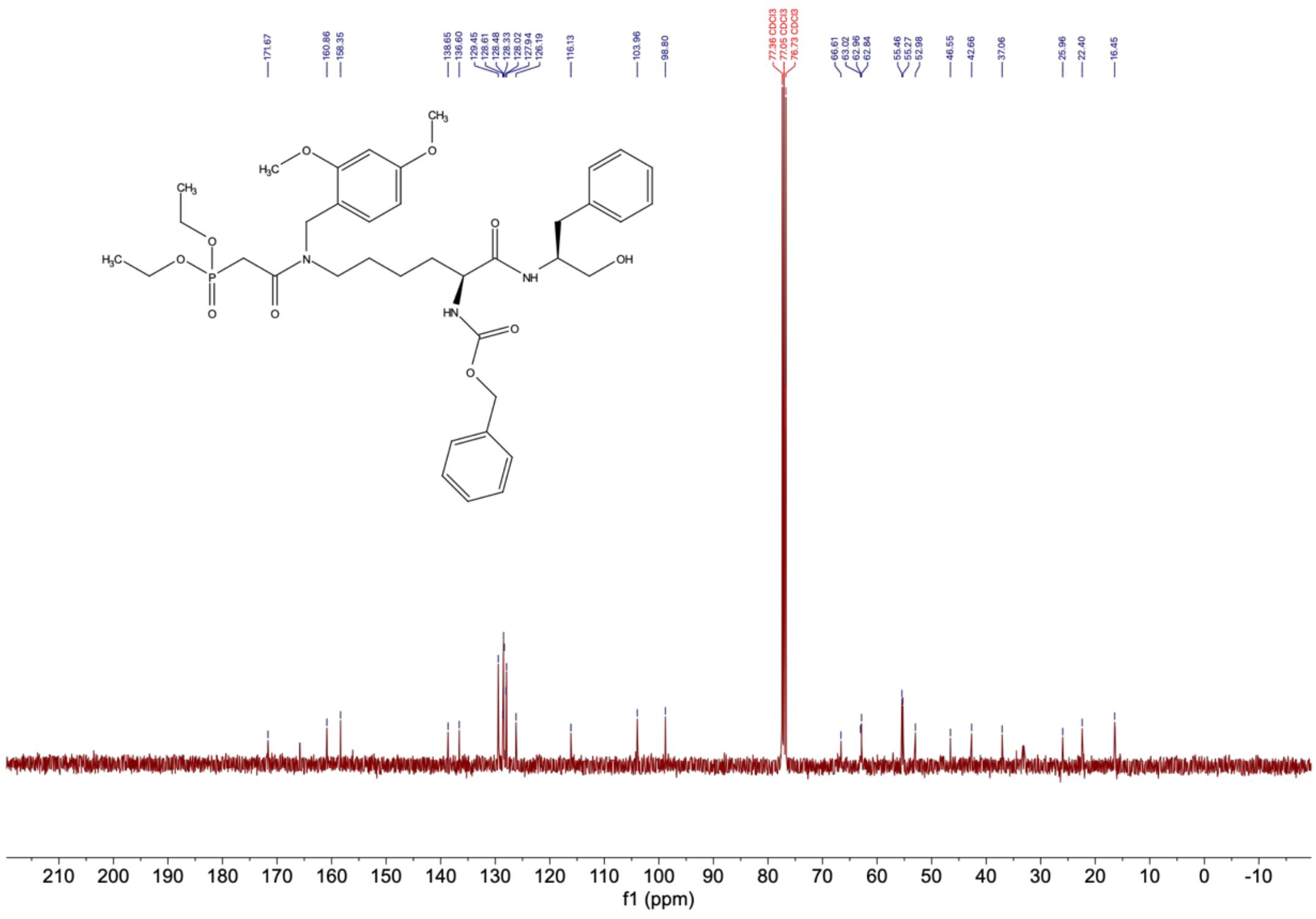

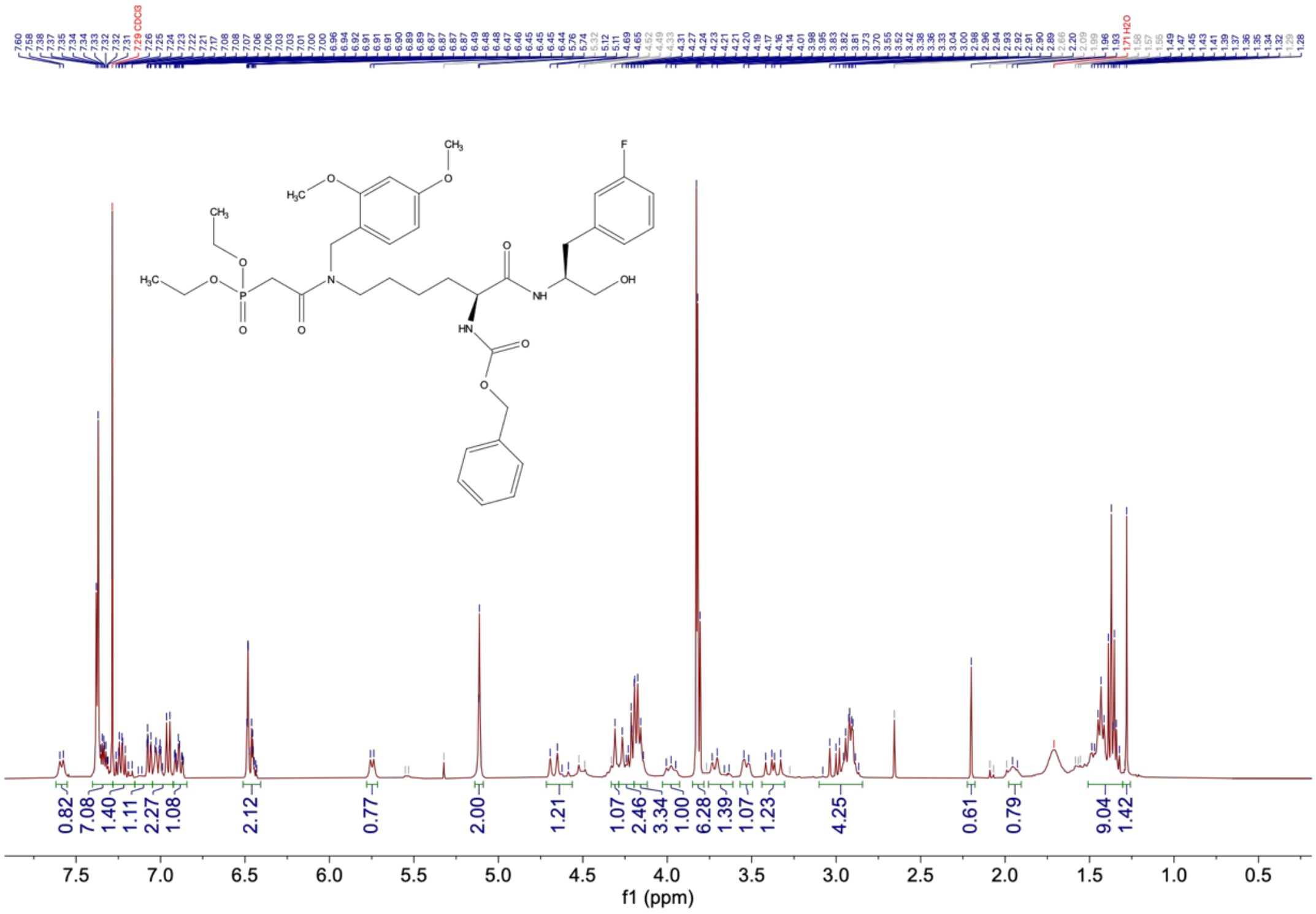

0.82 7.08 1.40 1.11 2.27 1.08 2.12 0.77 2.00 1.21 1.07 2.46 3.34 1.00 6.28 1.39 1.07 1.23 4.25 0.61 0.79 9.04 1.42
7.5 7.0 6.5 6.0 5.5 5.0 4.5 4.0 3.5 3.0 2.5 2.0 1.5 1.0 0.5
f1 (ppm)

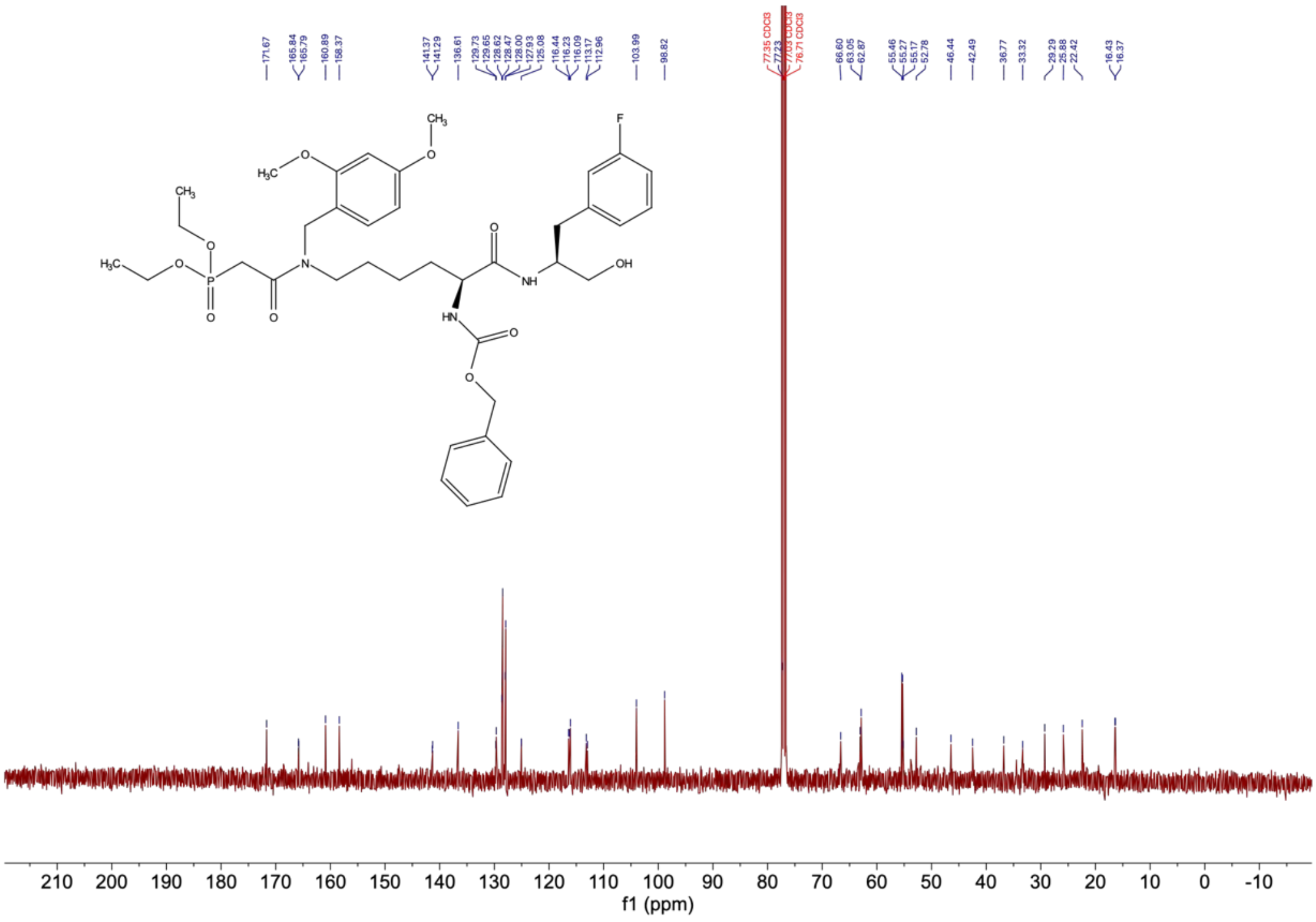

171.67 165.84 165.79 160.89 158.37 141.37 141.29 136.61 129.73 129.65 128.62 128.47 128.00 127.93 125.08 116.44 116.23 116.09 113.17 112.96 103.99 98.82
77.35 CDCl3 77.23 77.03 CDCl3 76.71 CDCl3
66.60 63.05 62.87 55.46 55.27 55.17 52.78 46.44 42.49 36.77 33.32 29.29 25.88 22.42 16.43 16.37
210 200 190 180 170 160 150 140 130 120 110 100 90 80 70 60 50 40 30 20 10 0 -10
f1 (ppm)

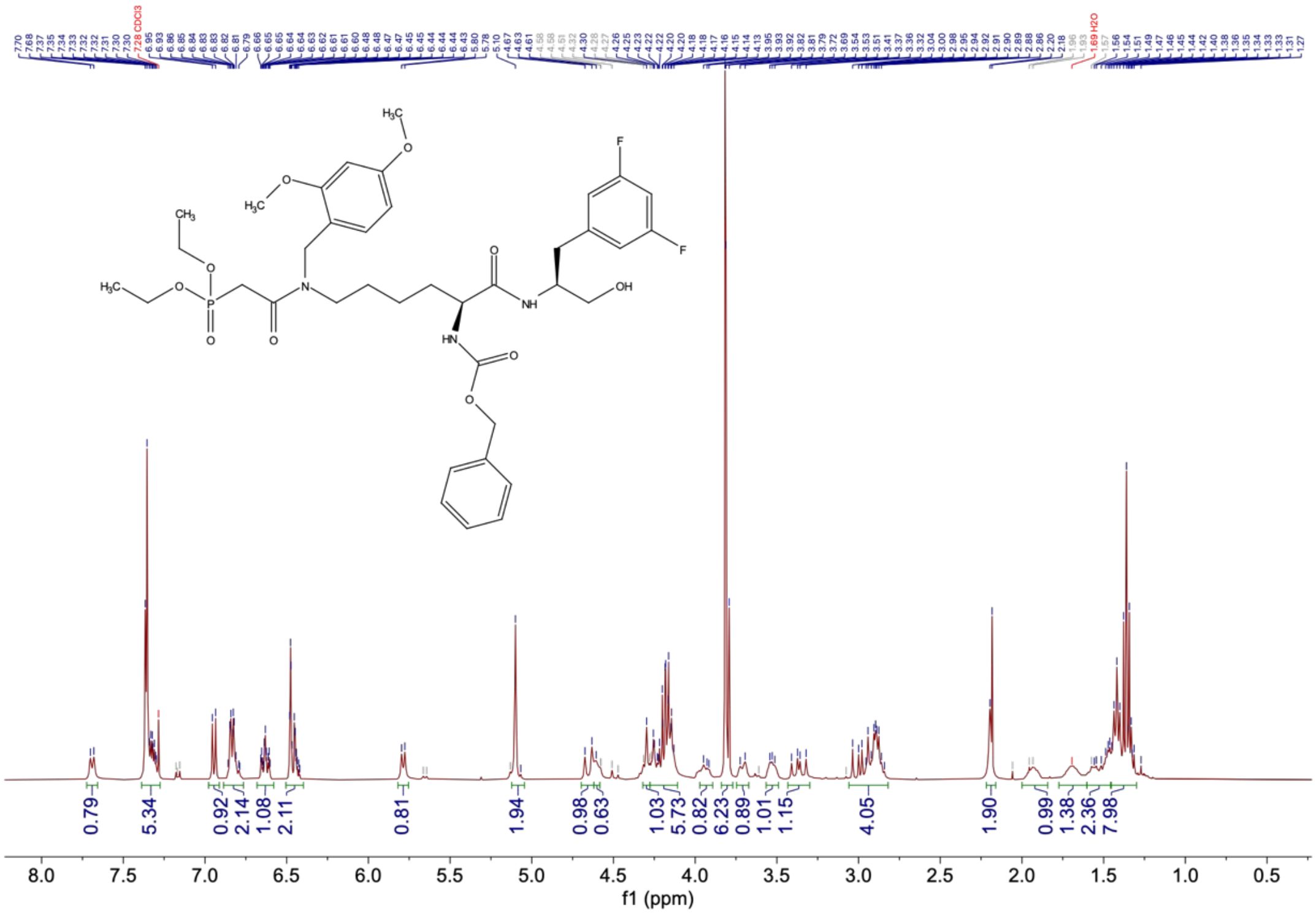
0.79
5.34
0.92
2.14
1.08
2.11
0.81
1.94
0.98
0.63
1.03
5.73
0.82
6.23
0.89
1.01
1.15
4.05
1.90
0.99
1.38
2.36
7.98
f1 (ppm)

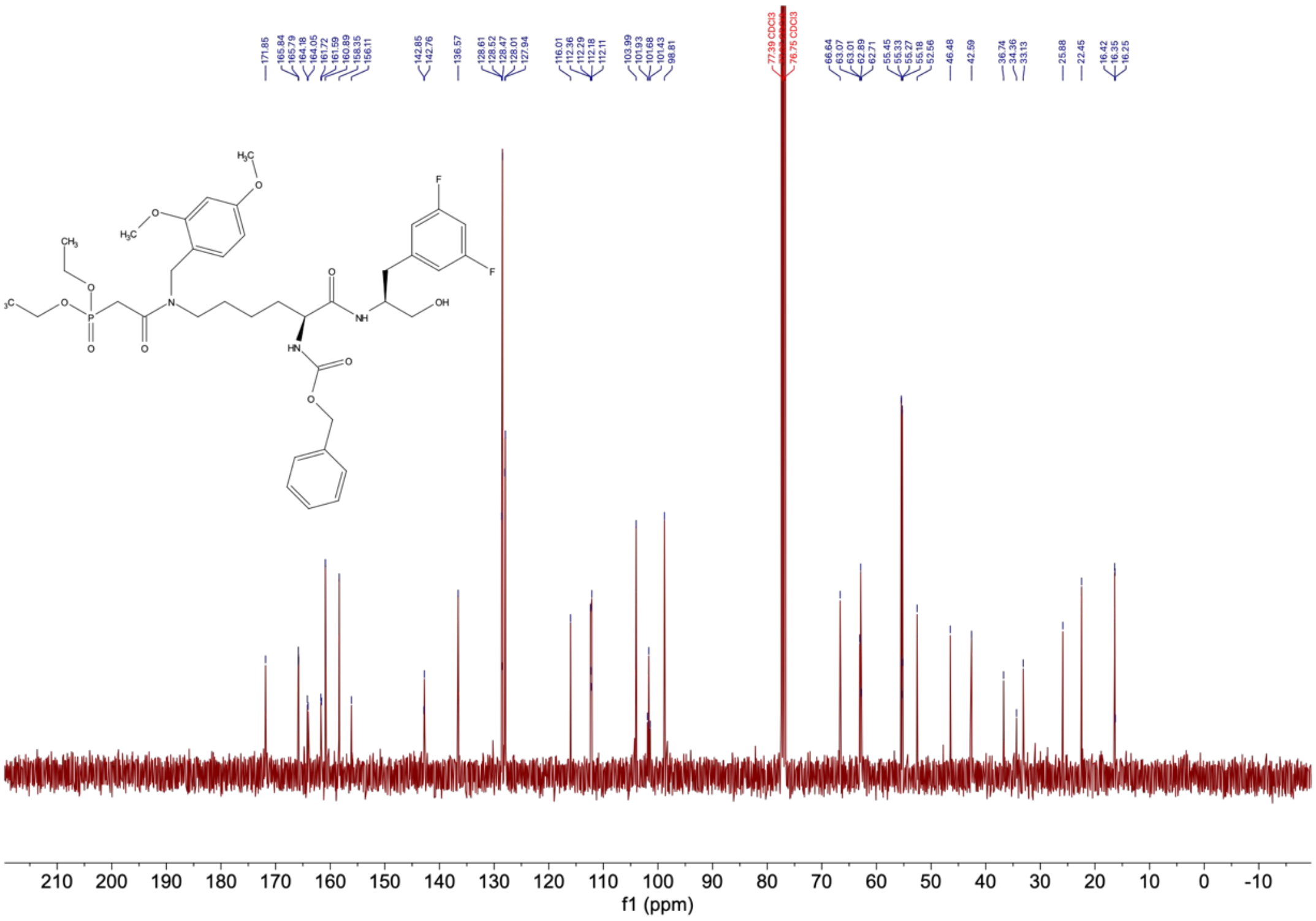
f1 (ppm)

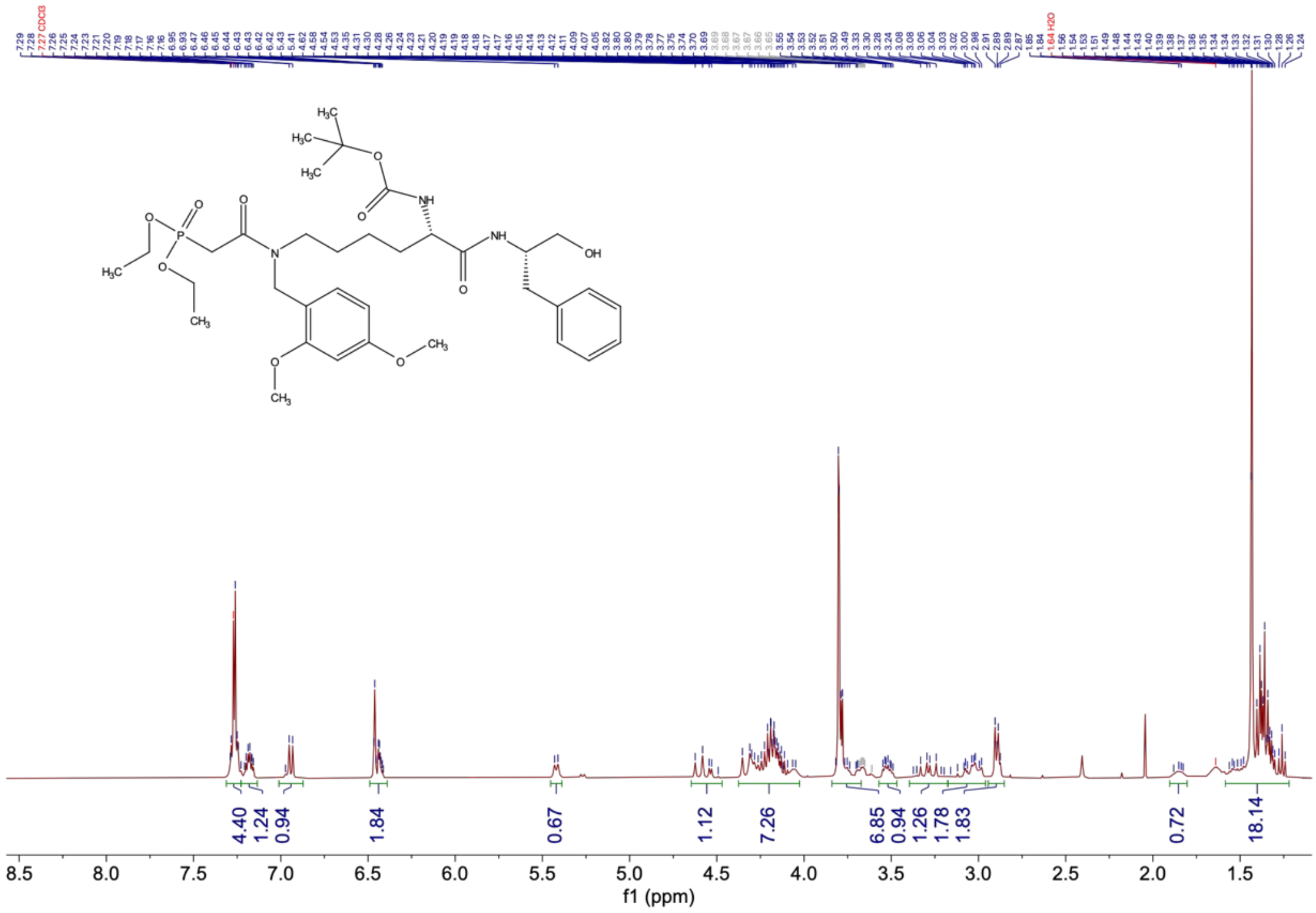

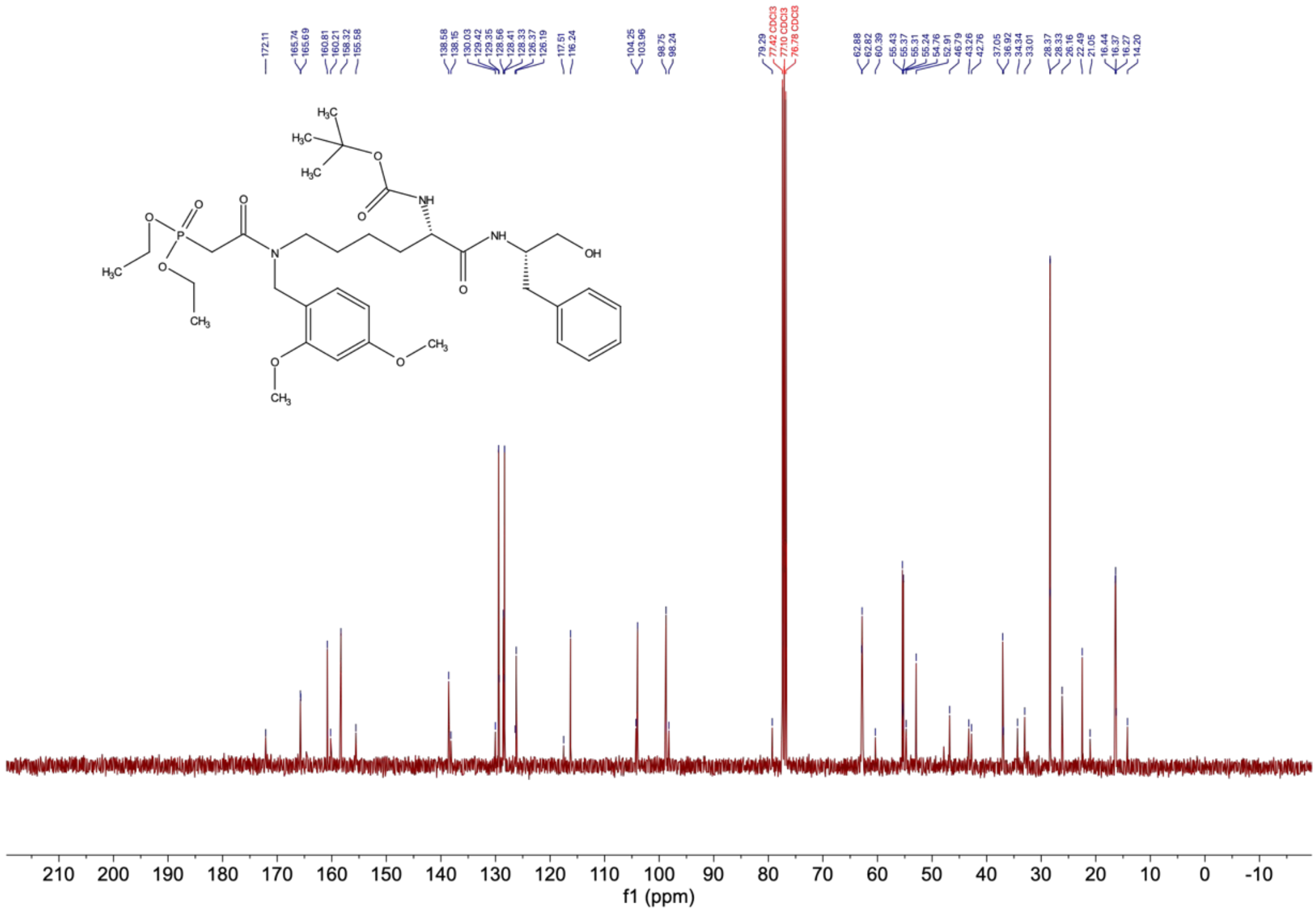

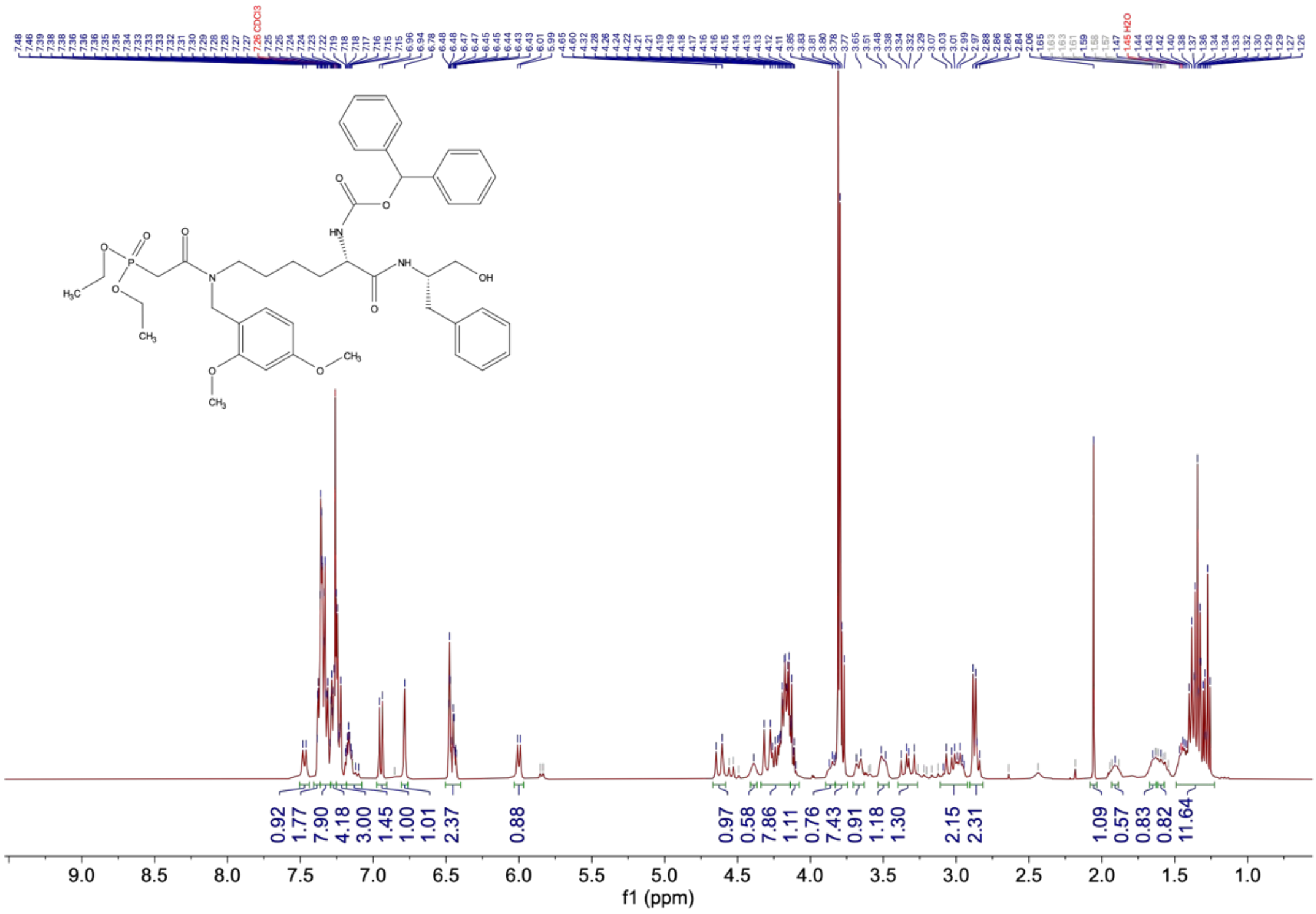

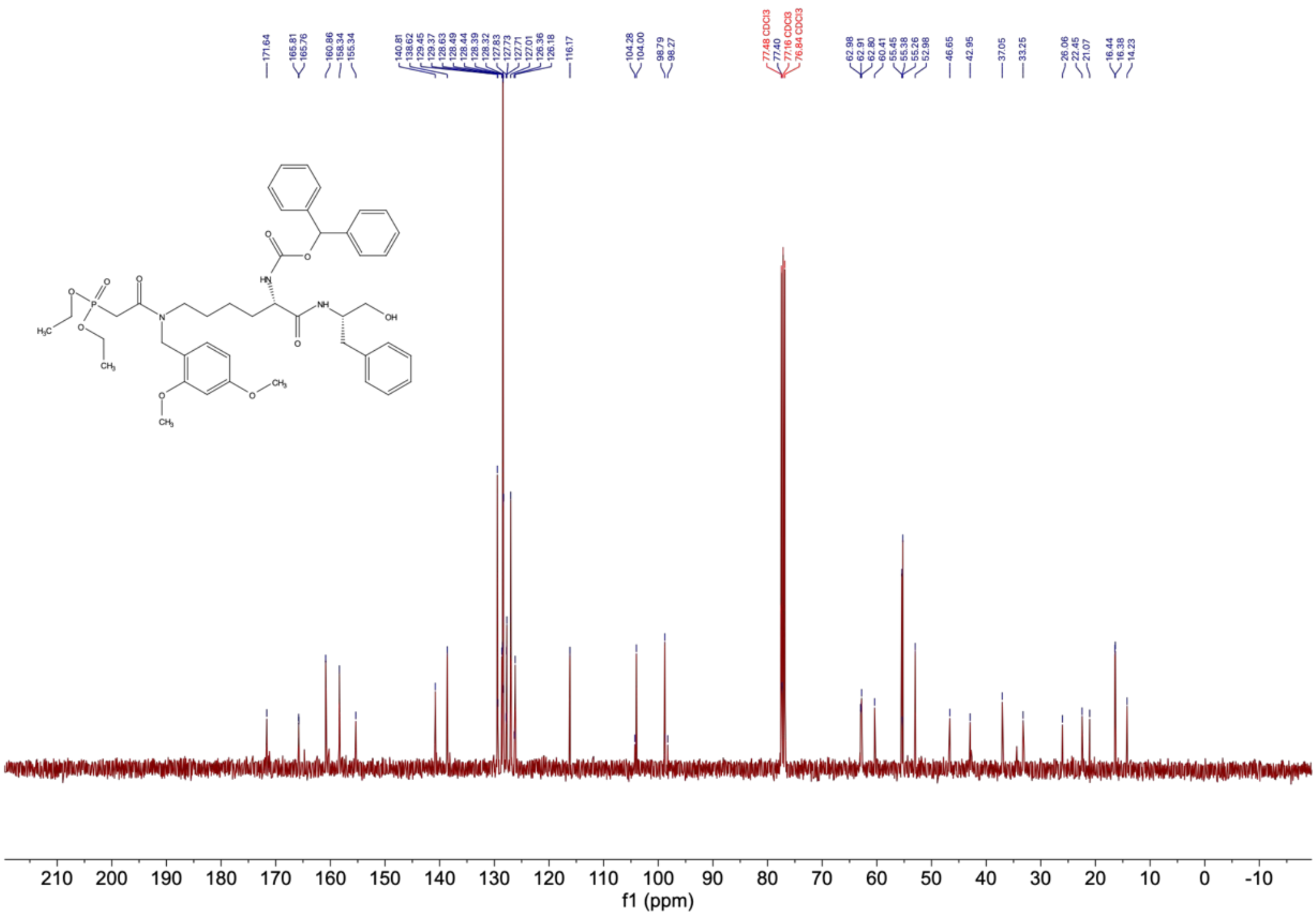

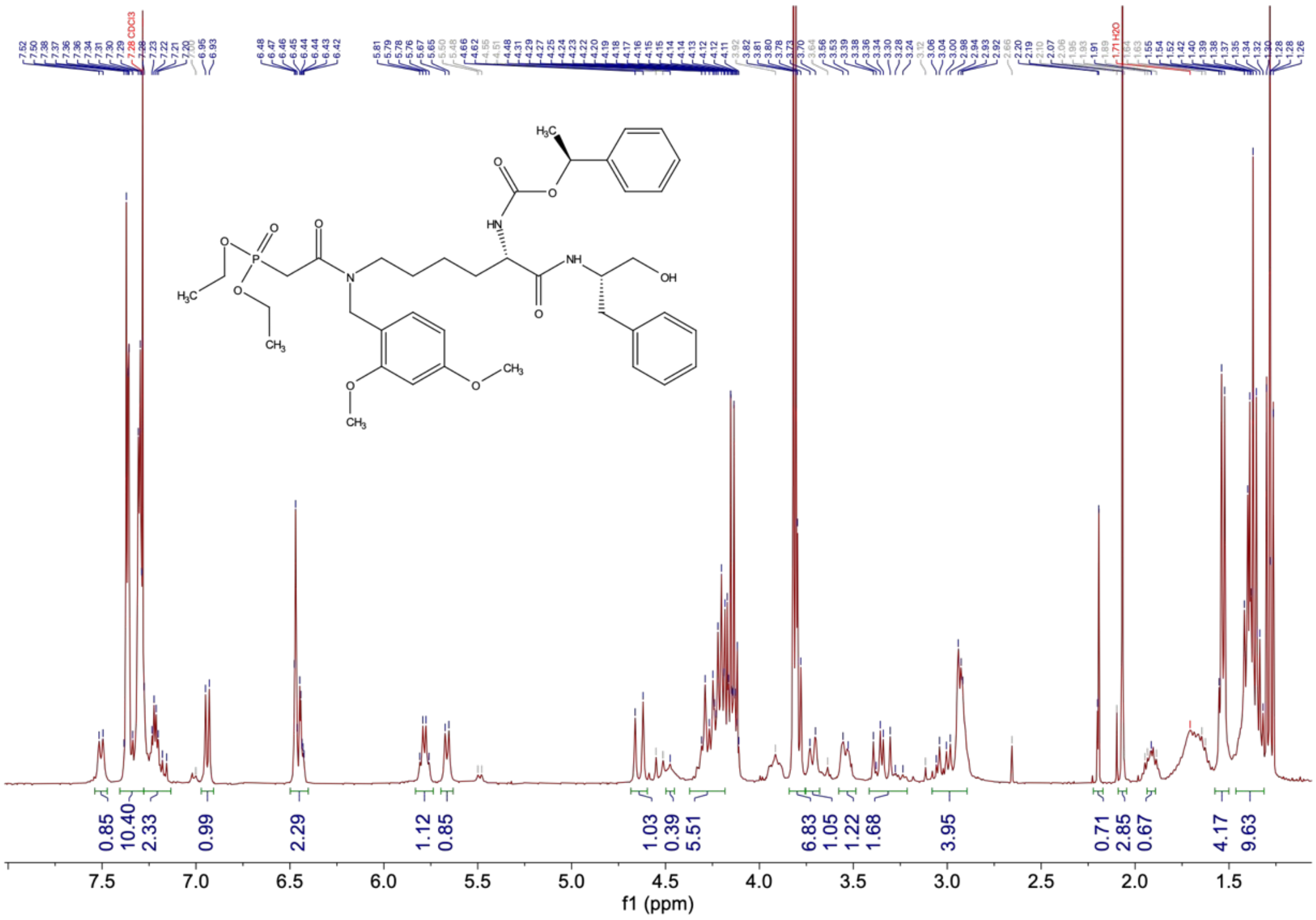

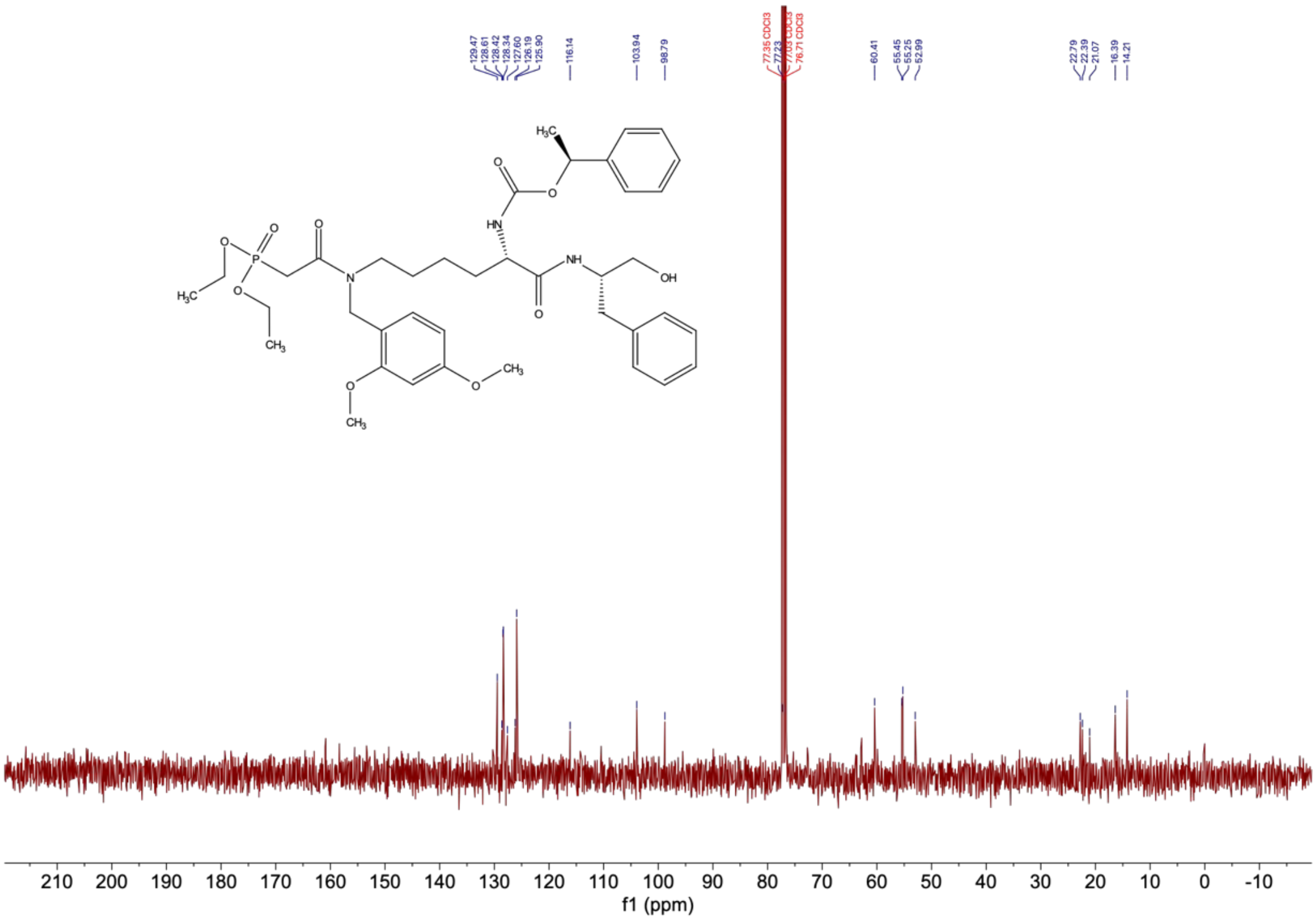

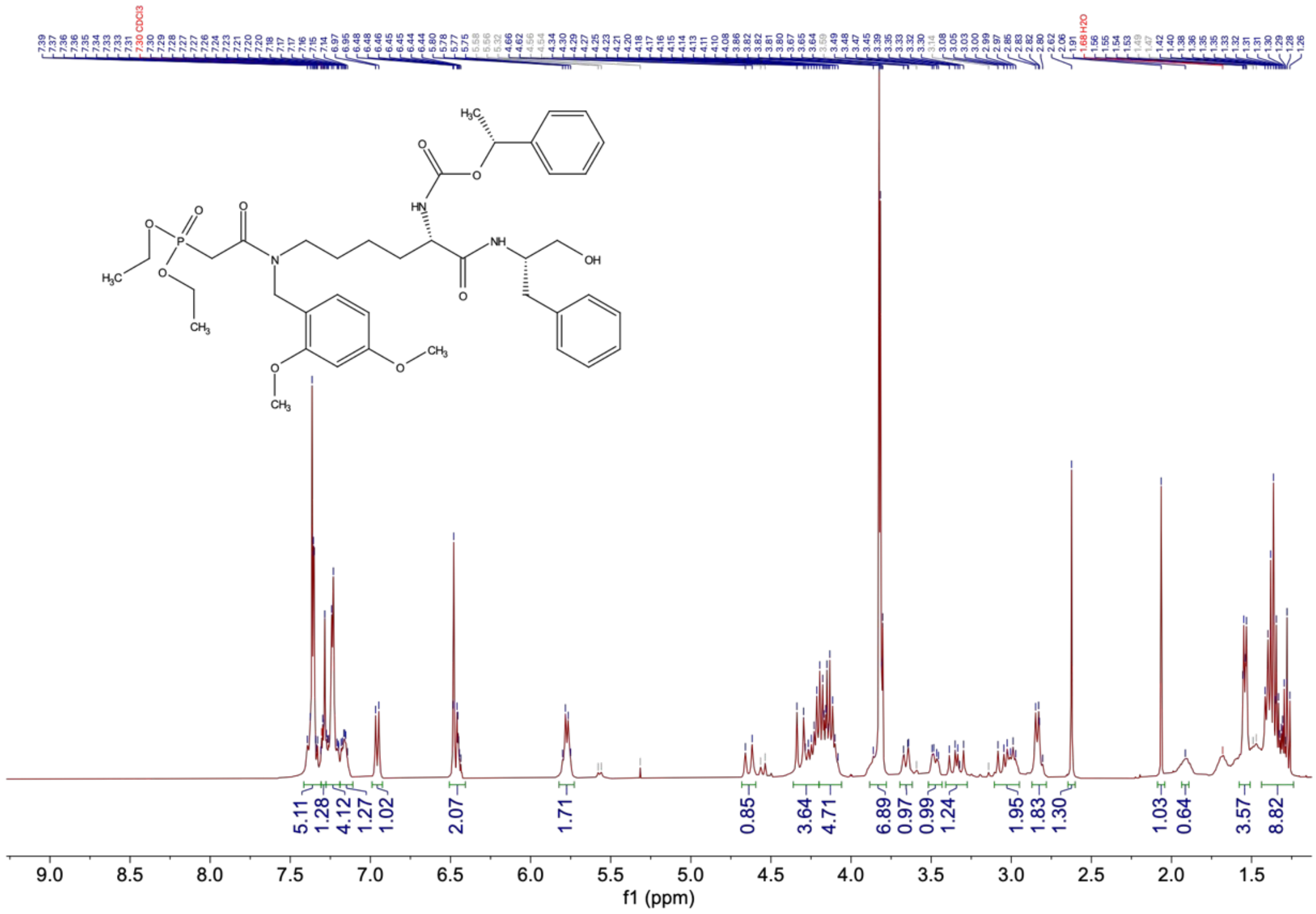

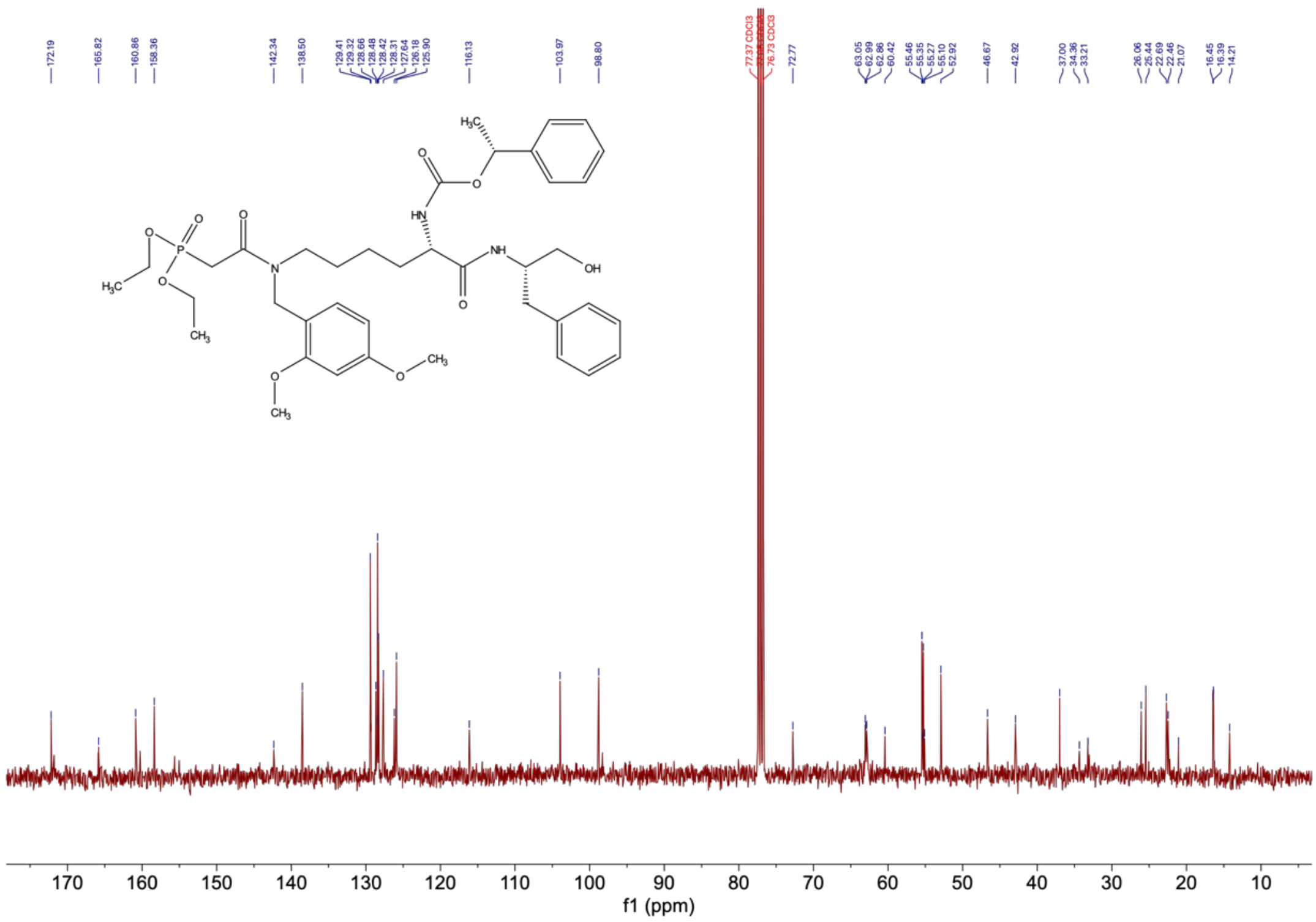

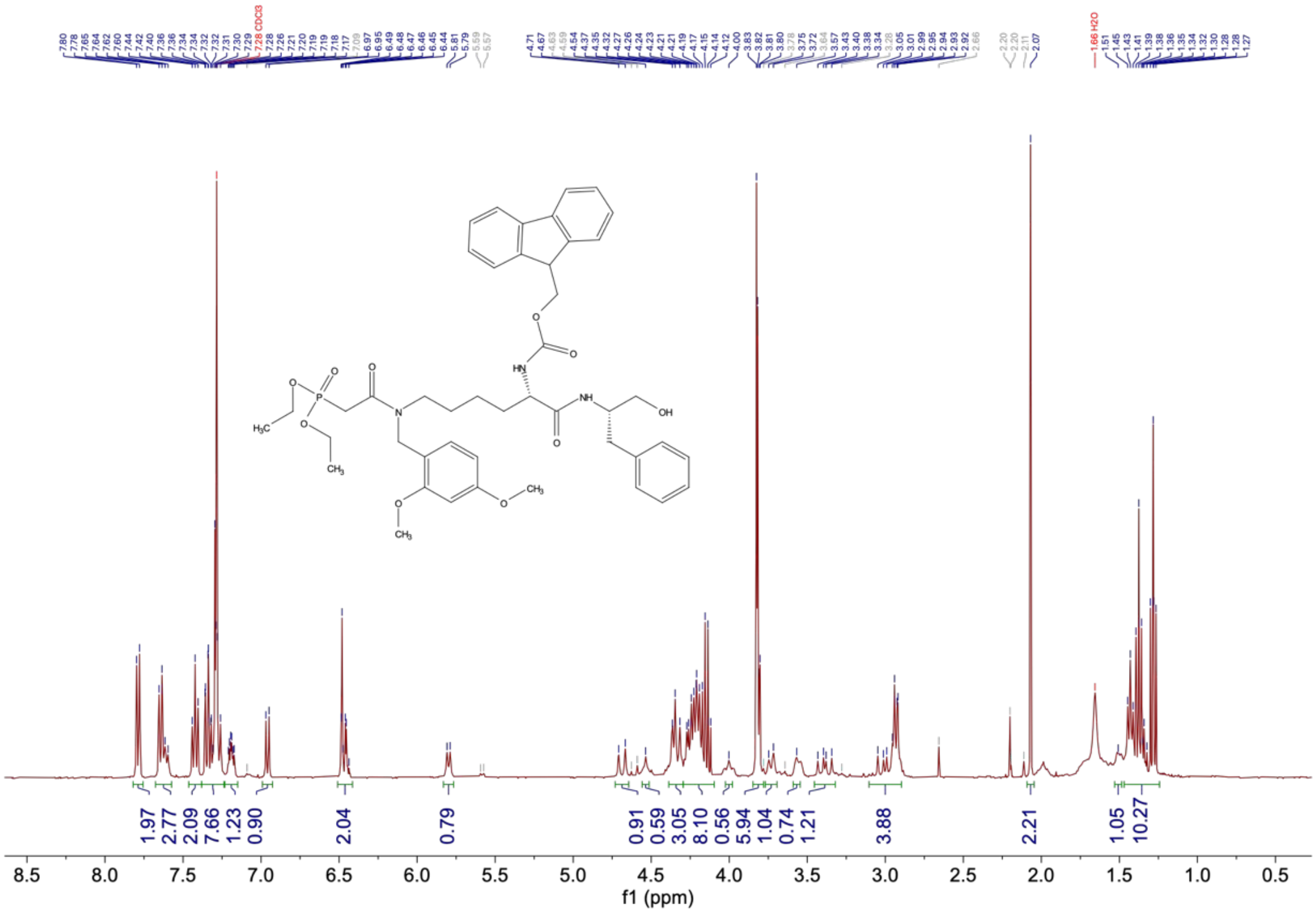
1.97
2.77
2.09
7.66
1.23
0.90
2.04
0.79
0.91
0.59
3.05
8.10
0.56
5.94
1.04
0.74
1.21
3.88
2.21
1.05
10.27
f1 (ppm)

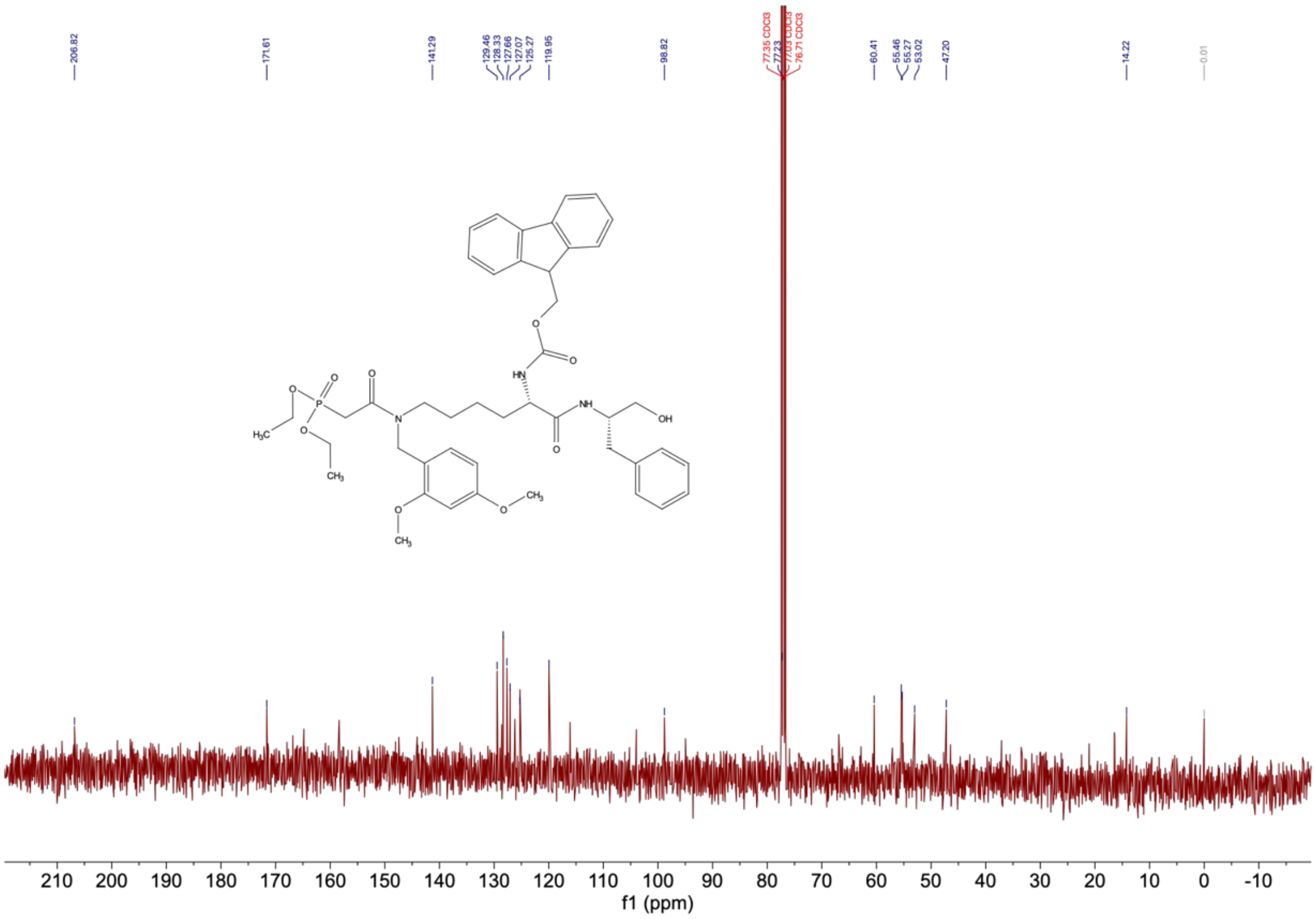
f1 (ppm)

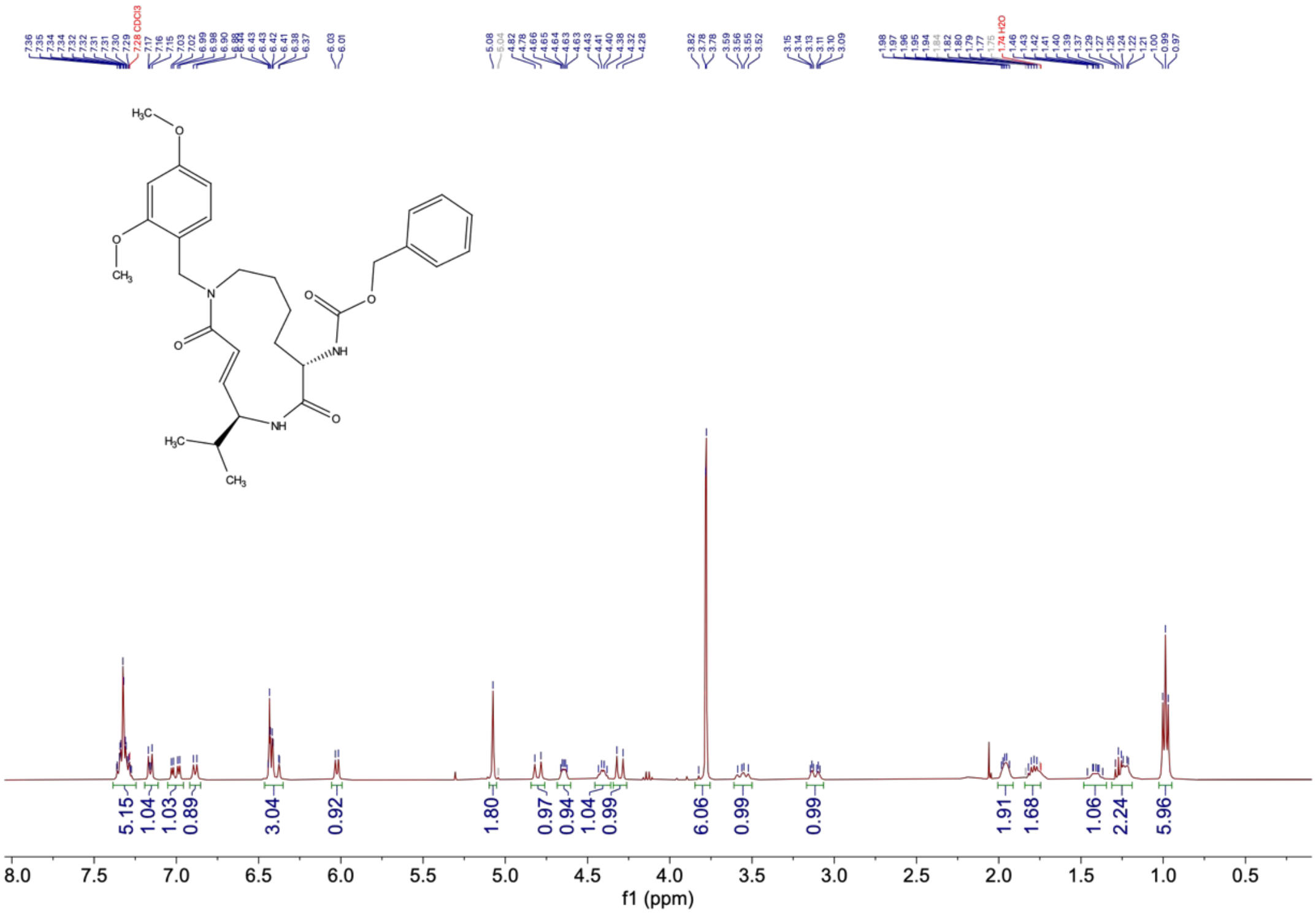

7.36
7.35
7.34
7.32
7.32
7.31
7.31
7.30
7.29
7.28 CDCl3
7.17
7.16
7.15
7.03
7.02
6.99
6.98
6.43
6.43
6.42
6.41
6.38
6.37
6.03
6.01
5.08
5.04
4.82
4.78
4.66
4.65
4.64
4.63
4.63
4.43
4.41
4.40
4.38
4.32
4.28
3.82
3.78
3.78
3.59
3.56
3.55
3.52
3.15
3.14
3.13
3.11
3.10
3.09
1.98
1.97
1.96
1.95
1.94
1.84
1.82
1.80
1.79
1.77
1.75
1.74 H2O
1.46
1.43
1.42
1.41
1.40
1.39
1.37
1.29
1.27
1.25
1.24
1.22
1.21
1.00
0.99
0.97
H3C
O
O
CH3
N
O
O
NH
O
O
NH
O
H3C
CH3
5.15
1.04
1.03
0.89
3.04
0.92
1.80
0.97
0.94
1.04
0.99
6.06
0.99
0.99
1.91
1.68
1.06
2.24
5.96
8.0
7.5
7.0
6.5
6.0
5.5
5.0
4.5
4.0
3.5
3.0
2.5
2.0
1.5
1.0
0.5
f1 (ppm)

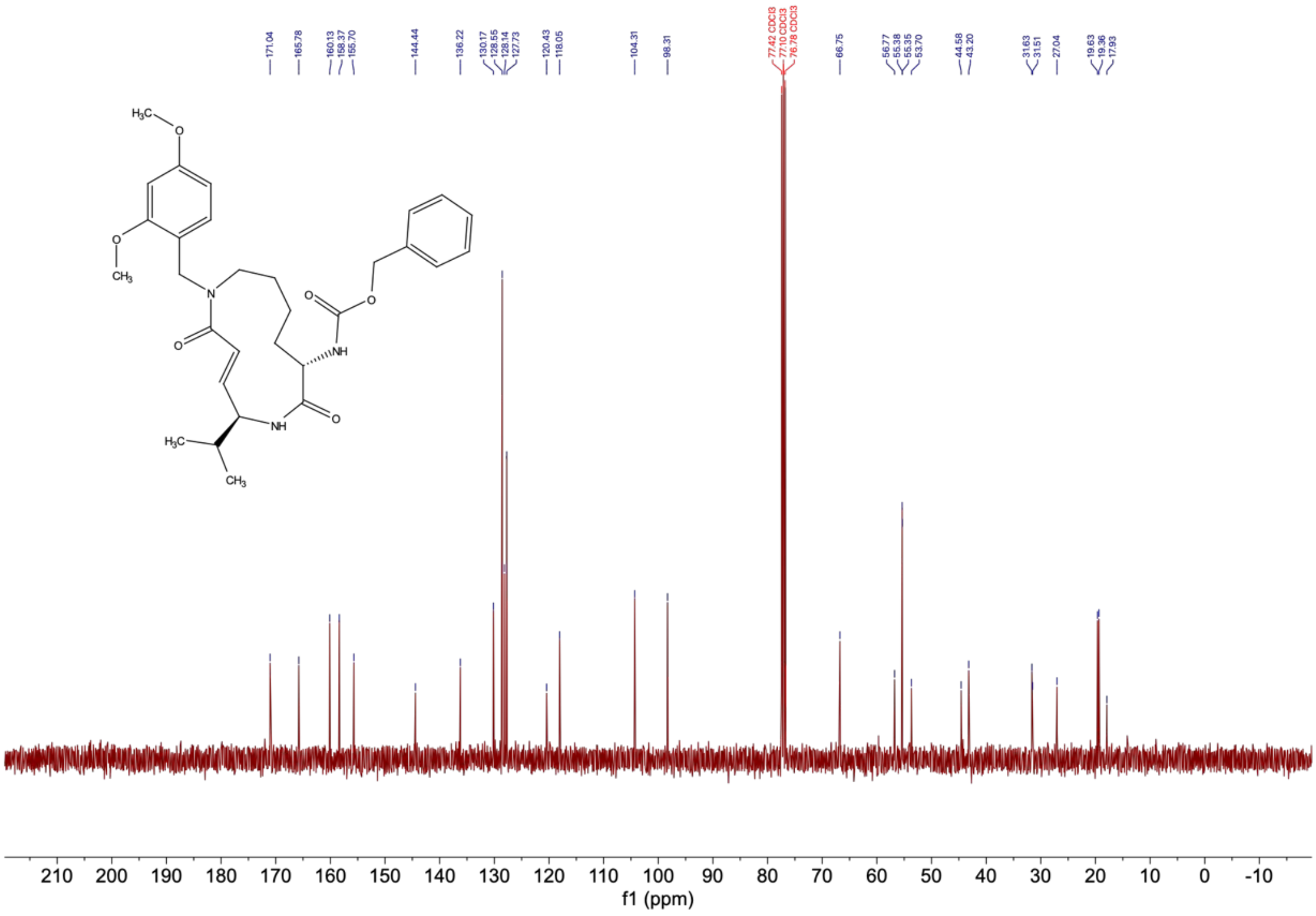

171.04
165.78
160.13
158.37
155.70
144.44
136.22
130.17
128.55
128.14
127.73
120.43
118.05
104.31
98.31
77.42 CDCl3
77.10 CDCl3
76.78 CDCl3
66.75
56.77
55.38
55.35
53.70
44.58
43.20
31.63
31.51
27.04
19.63
19.36
17.93
H3C
O
O
CH3
N
O
O
NH
O
O
NH
O
H3C
CH3
210
200
190
180
170
160
150
140
130
120
110
100
90
80
70
60
50
40
30
20
10
0
-10
f1 (ppm)

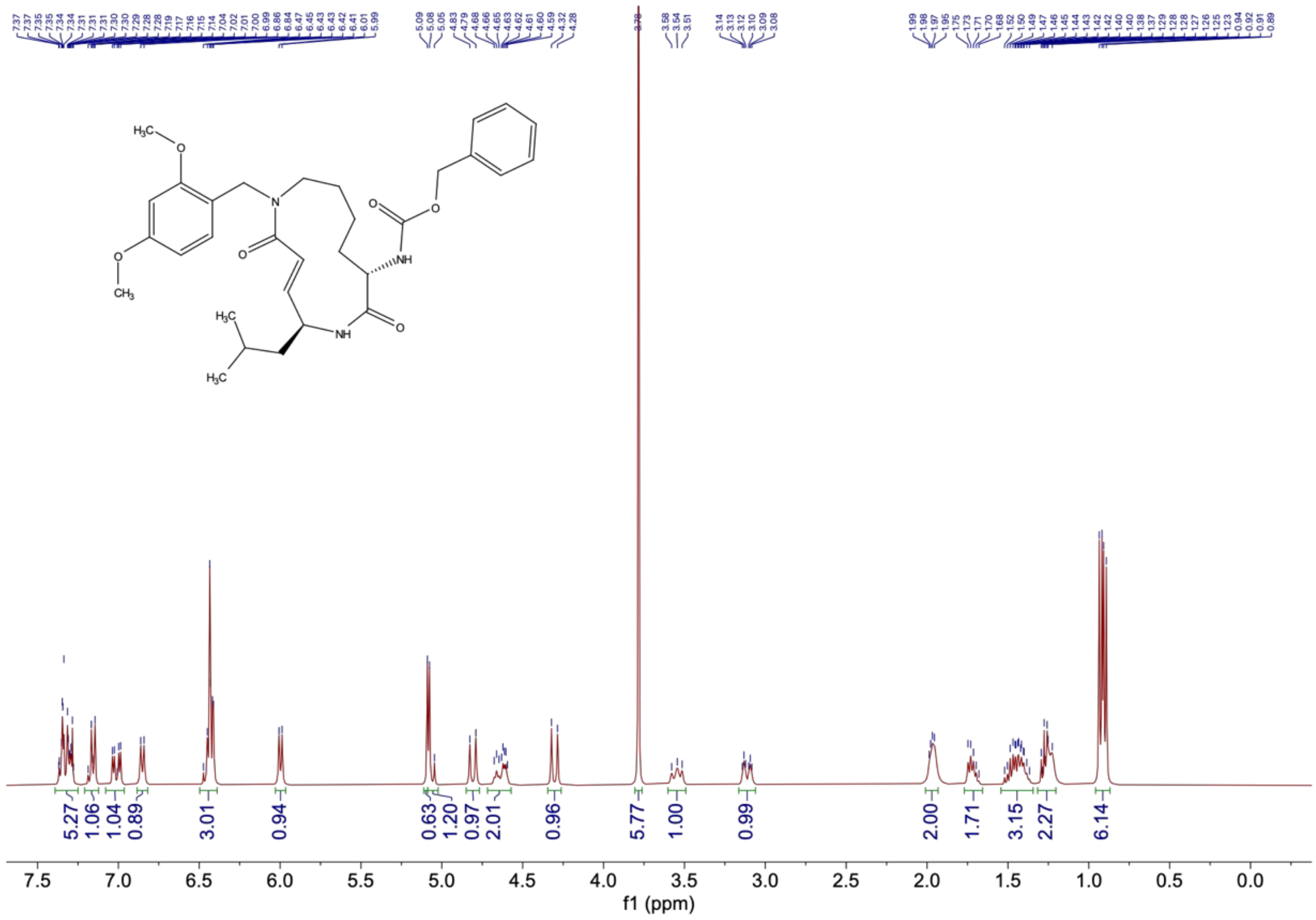

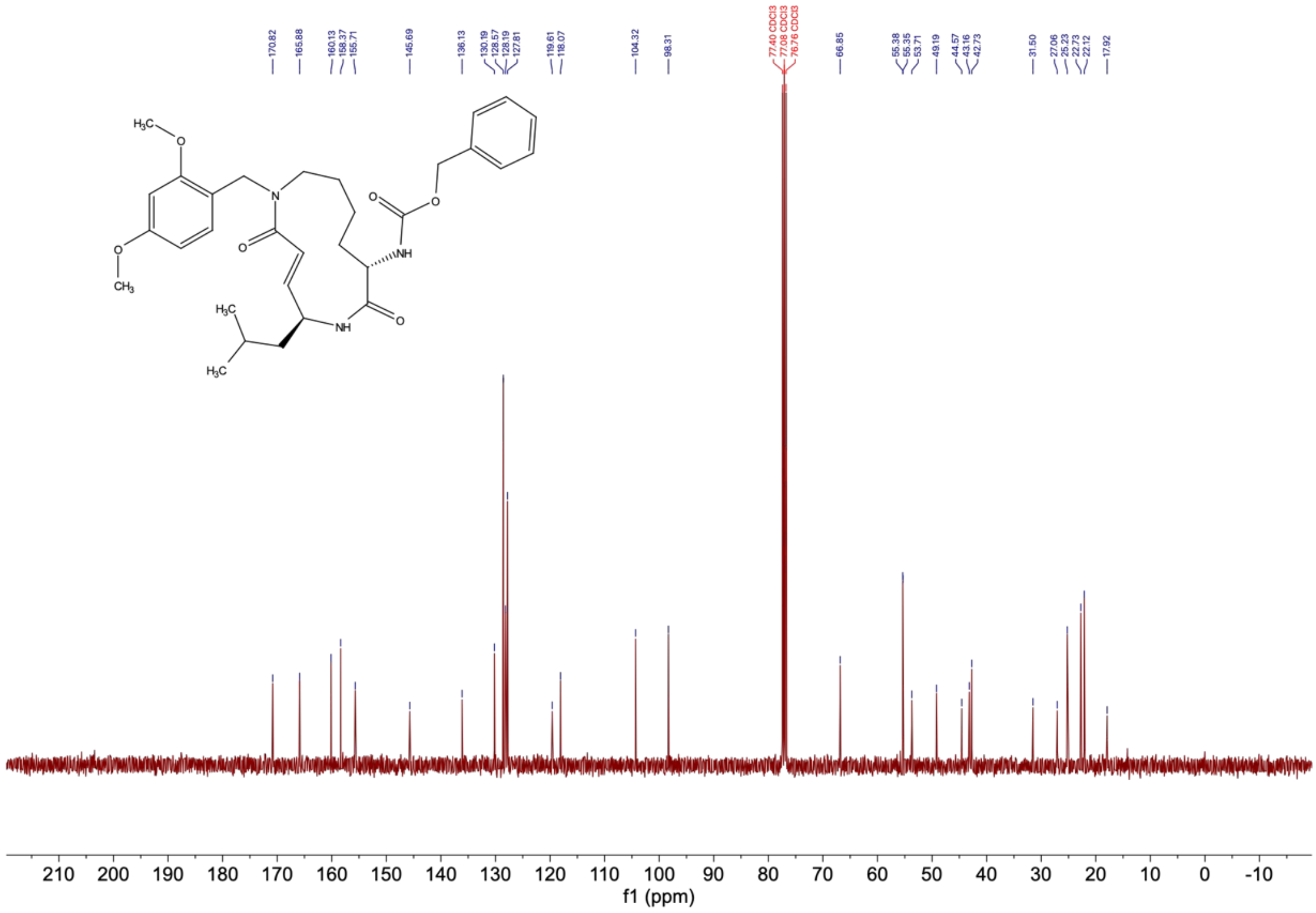

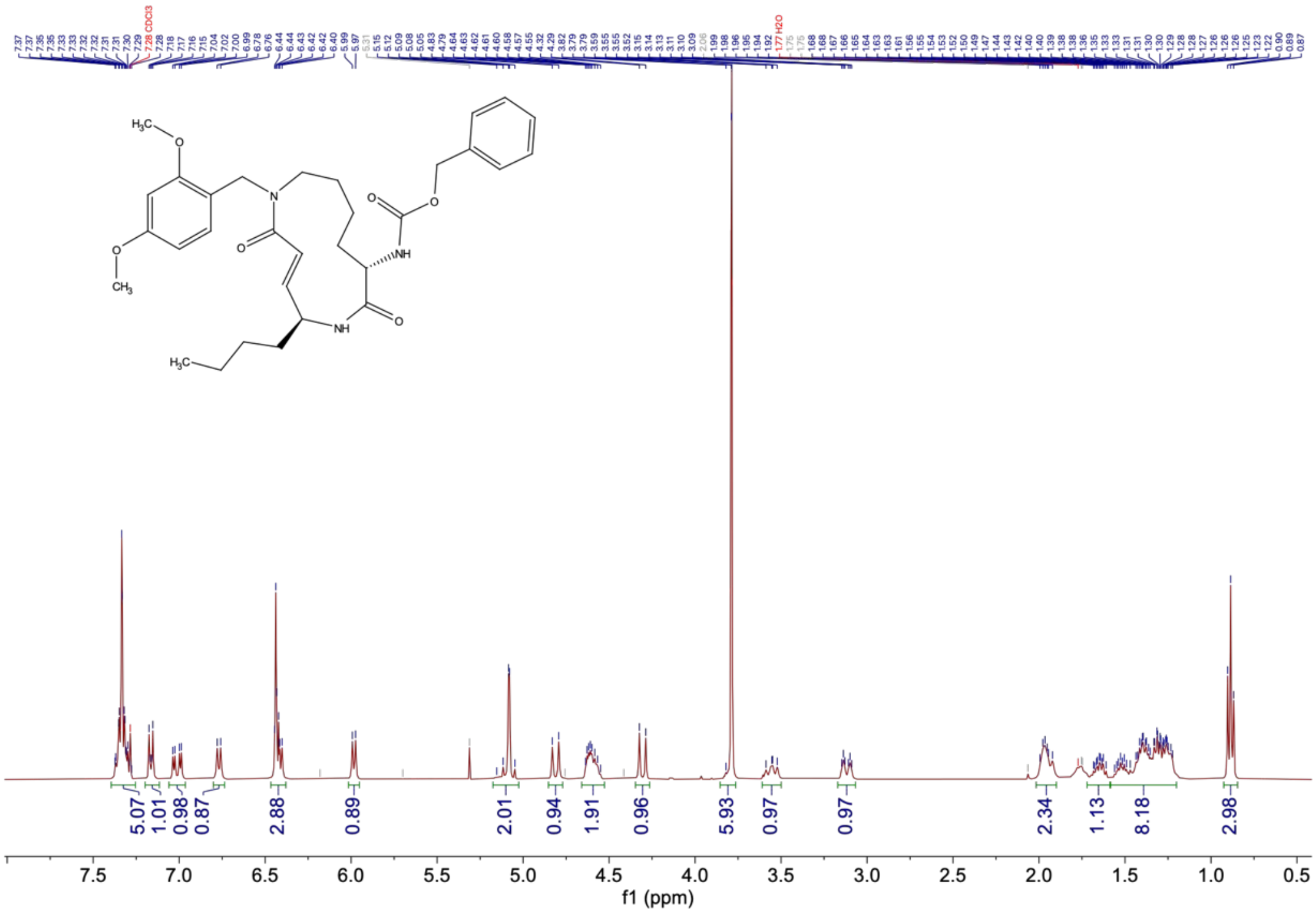

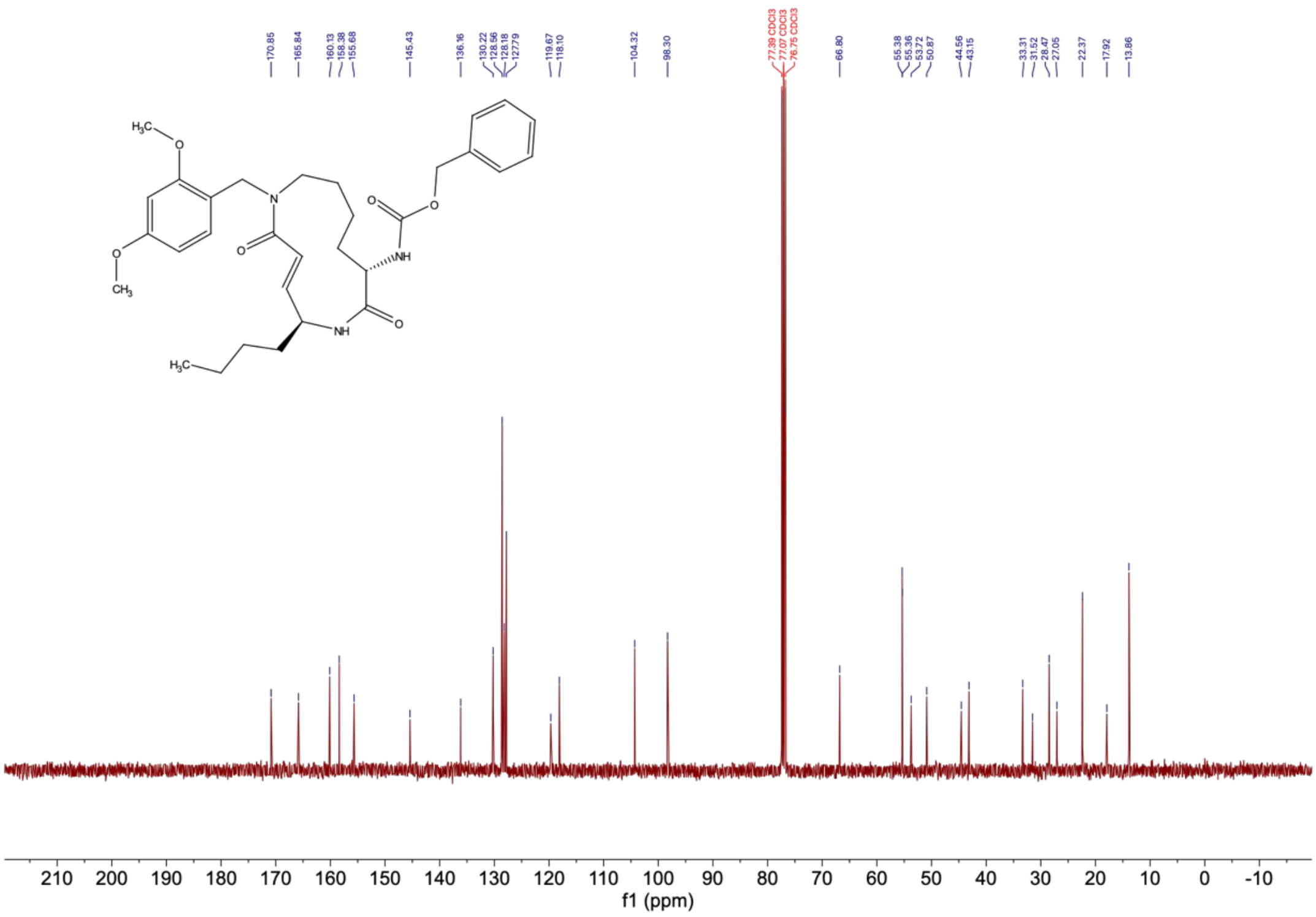

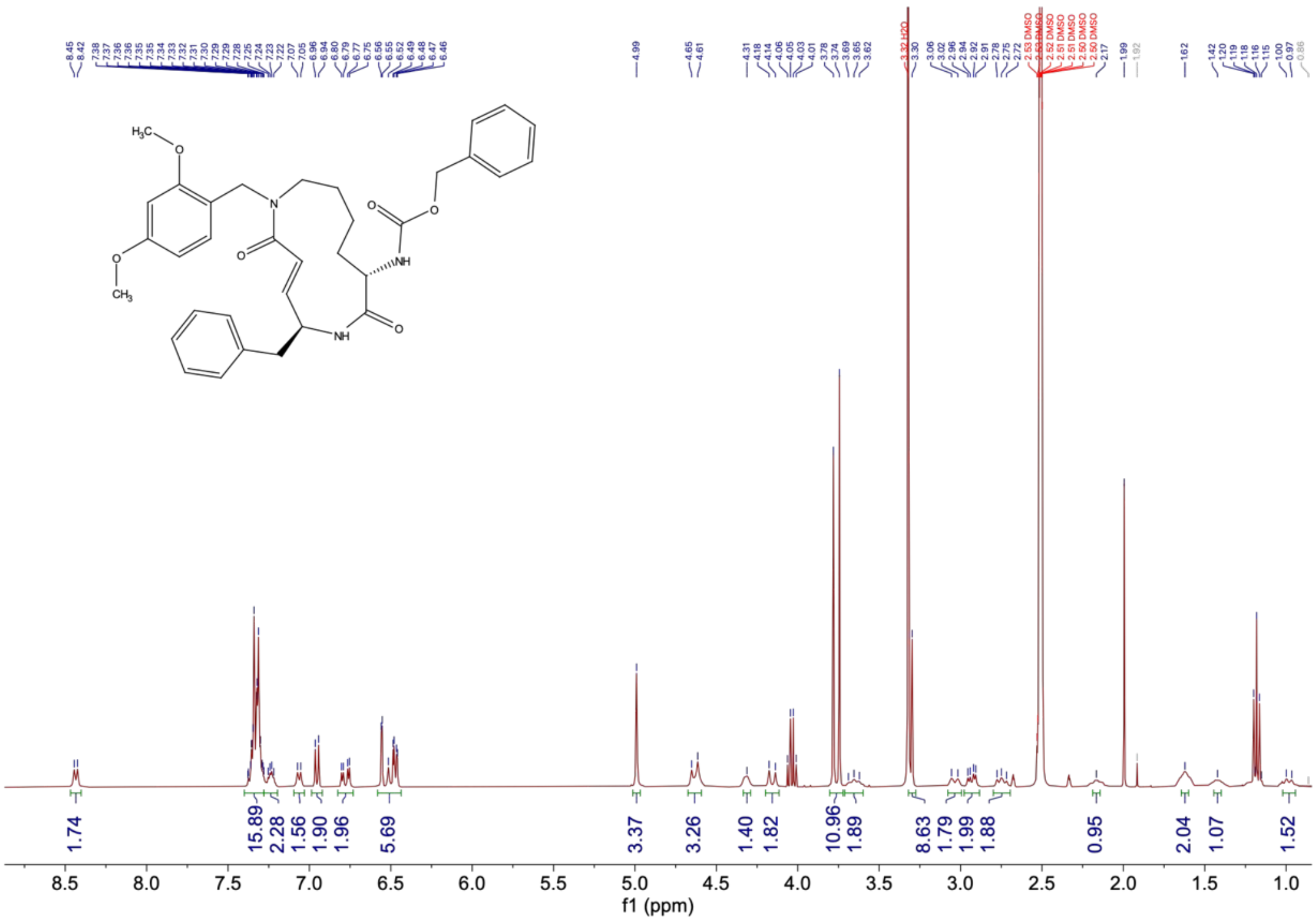
1.74
15.89
2.28
1.56
1.90
1.96
5.69
3.37
3.26
1.40
1.82
10.96
1.89
8.63
1.79
1.99
1.88
0.95
2.04
1.07
1.52
8.5 8.0 7.5 7.0 6.5 6.0 5.5 5.0 4.5 4.0 3.5 3.0 2.5 2.0 1.5 1.0
f1 (ppm)

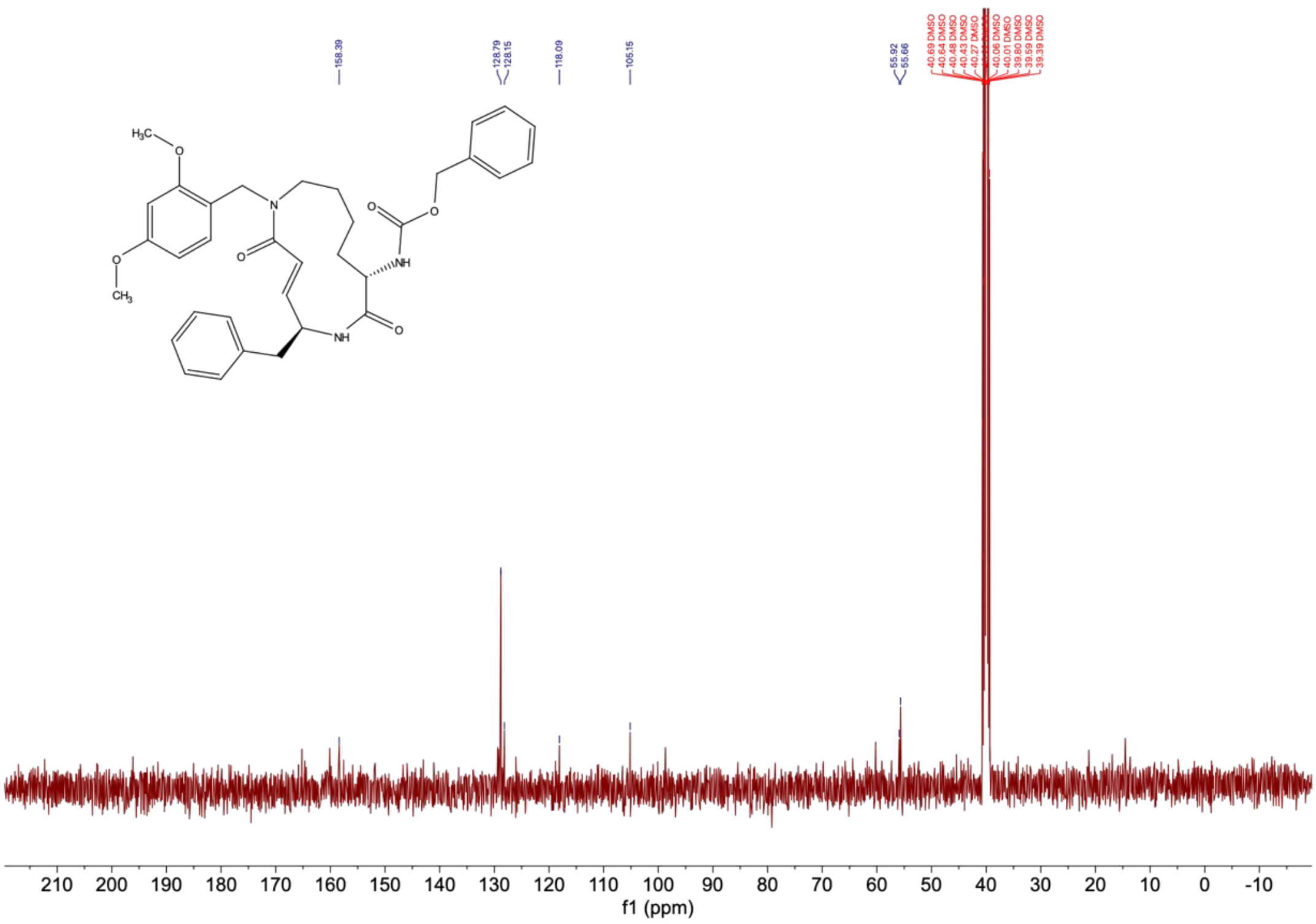
158.39
128.79
128.15
118.09
105.15
55.92
55.66
210 200 190 180 170 160 150 140 130 120 110 100 90 80 70 60 50 40 30 20 10 0 -10
f1 (ppm)

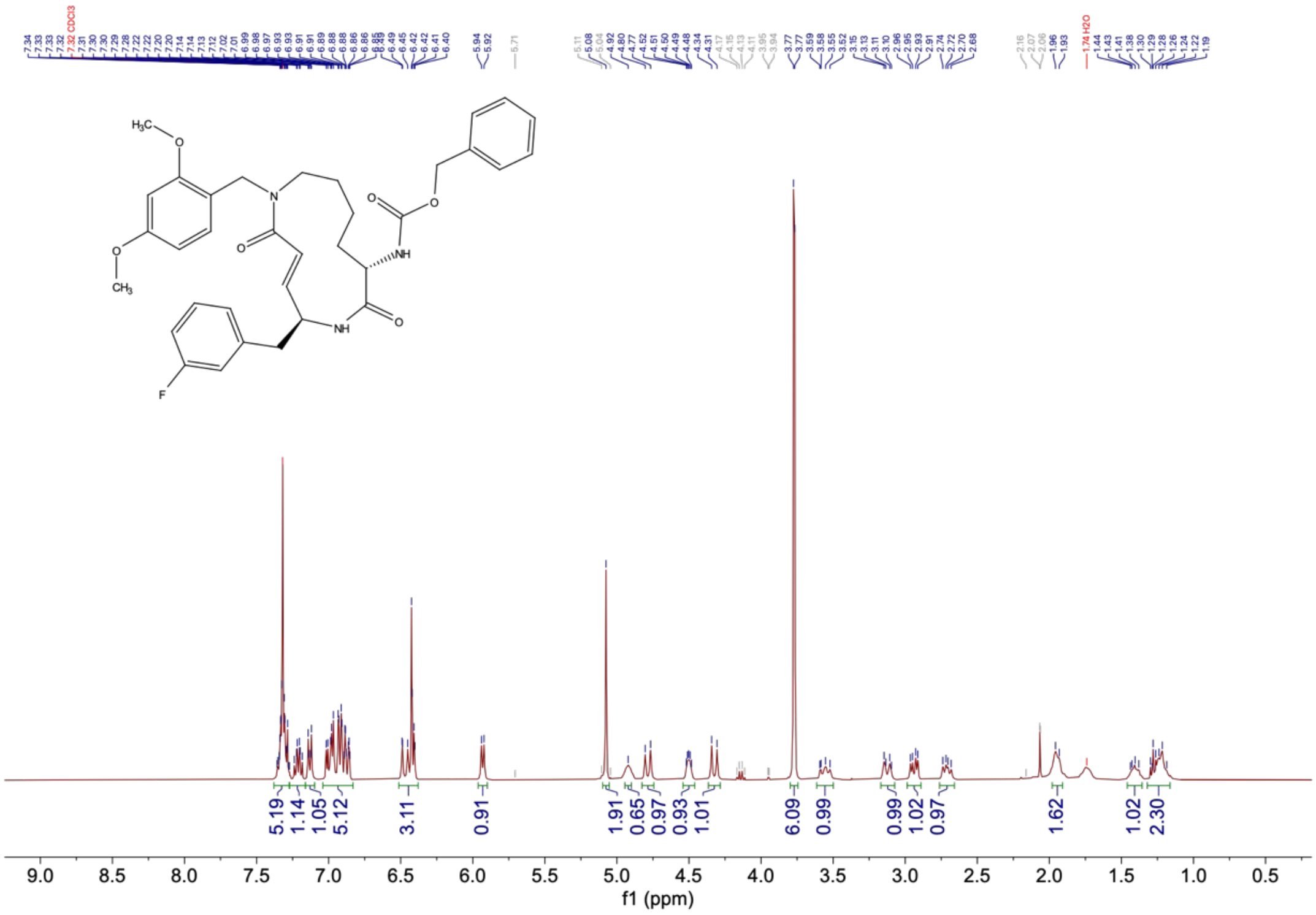

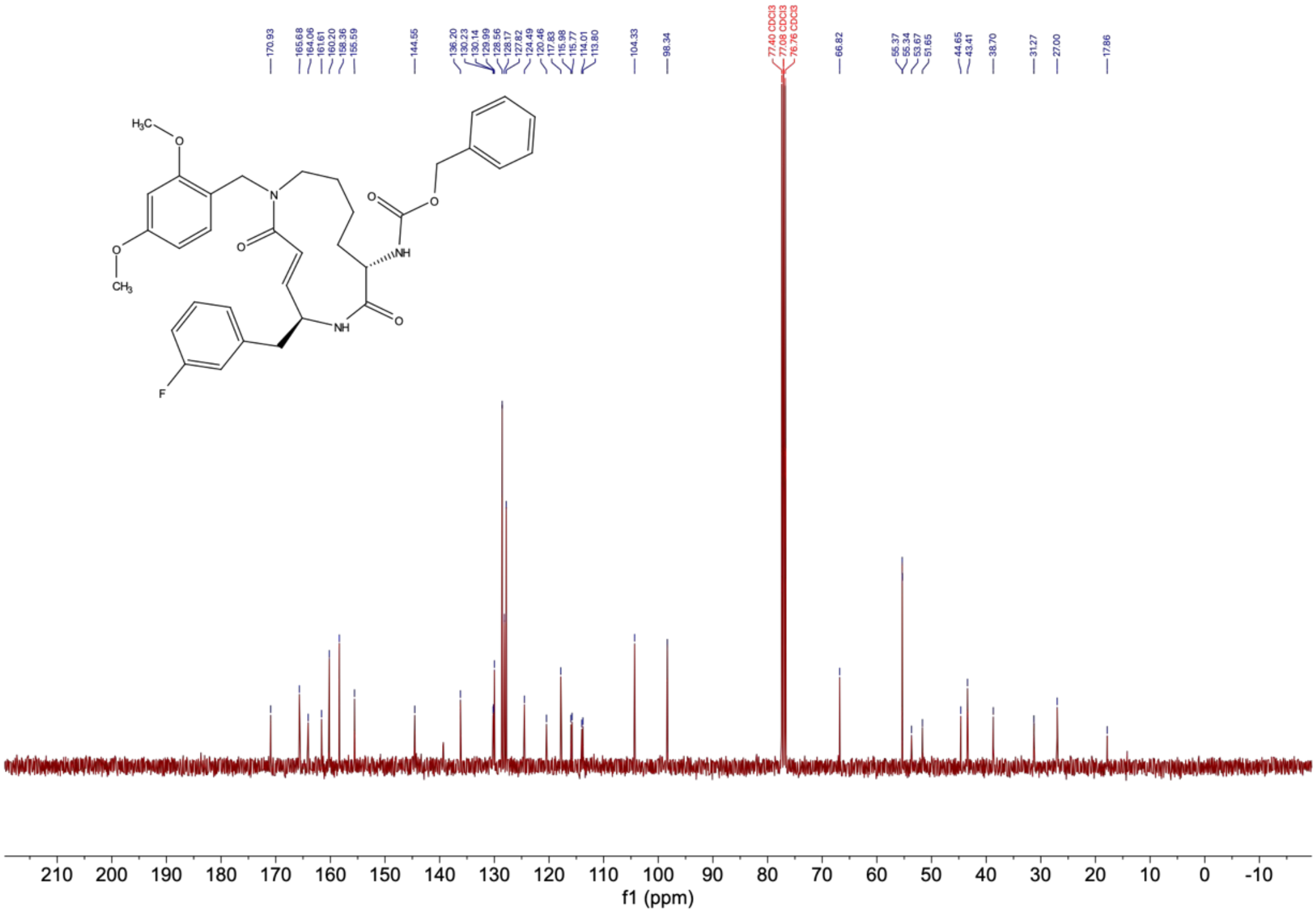

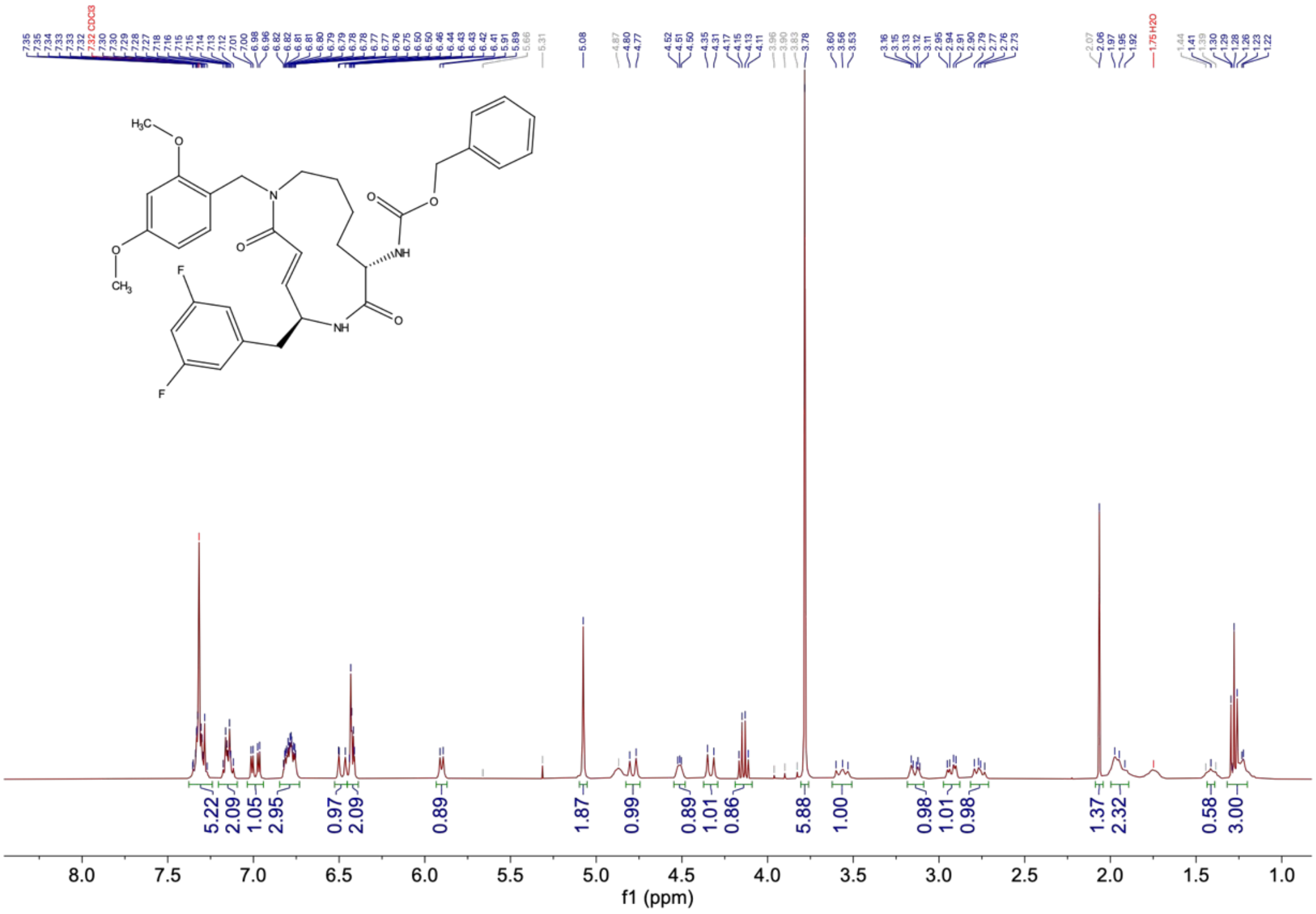

H3C
O
N
O
O
O
NH
O
CH3
F
F
NH
O
O
f1 (ppm)

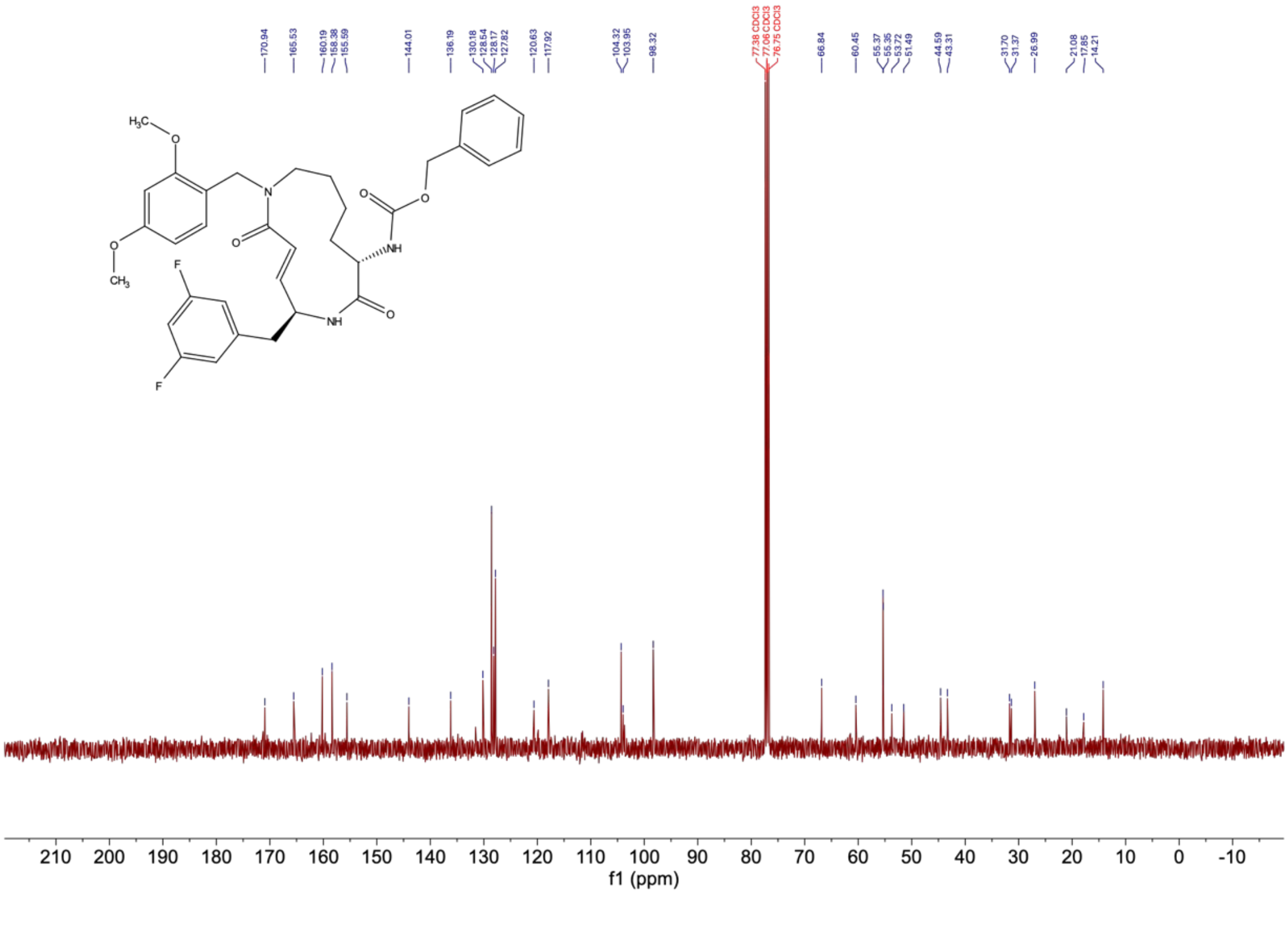

H3C
O
N
O
O
O
NH
O
CH3
F
F
NH
O
O
f1 (ppm)

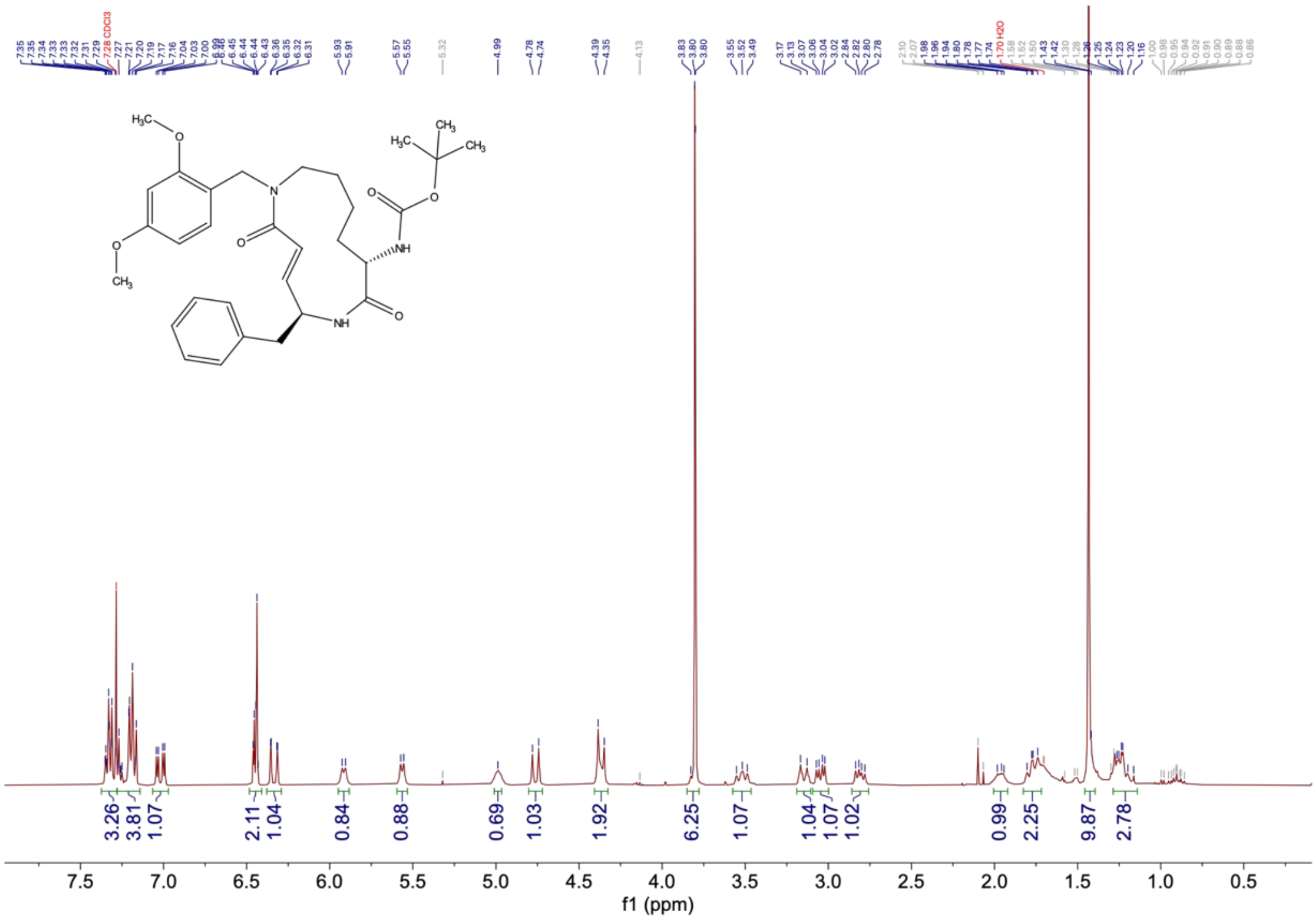

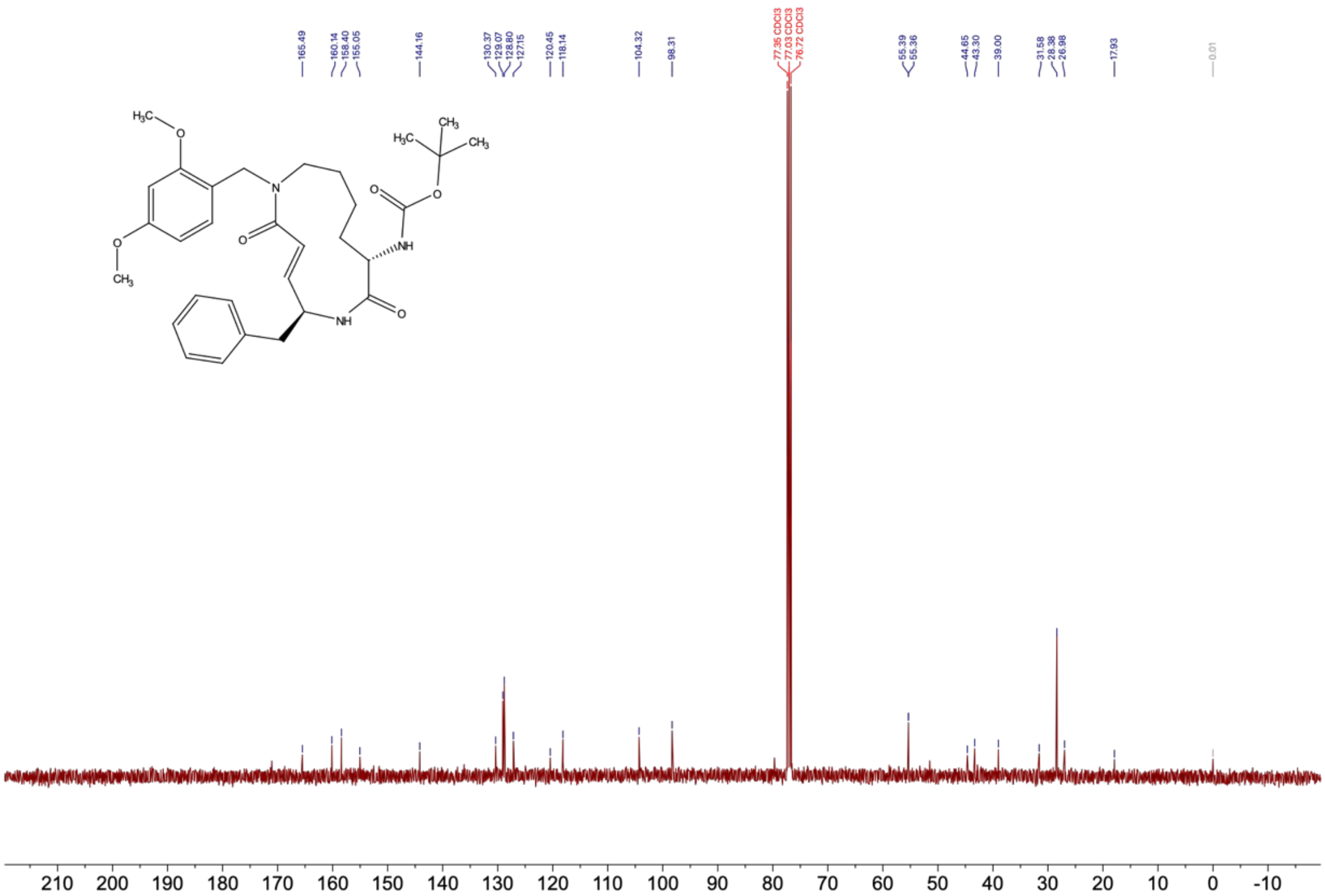

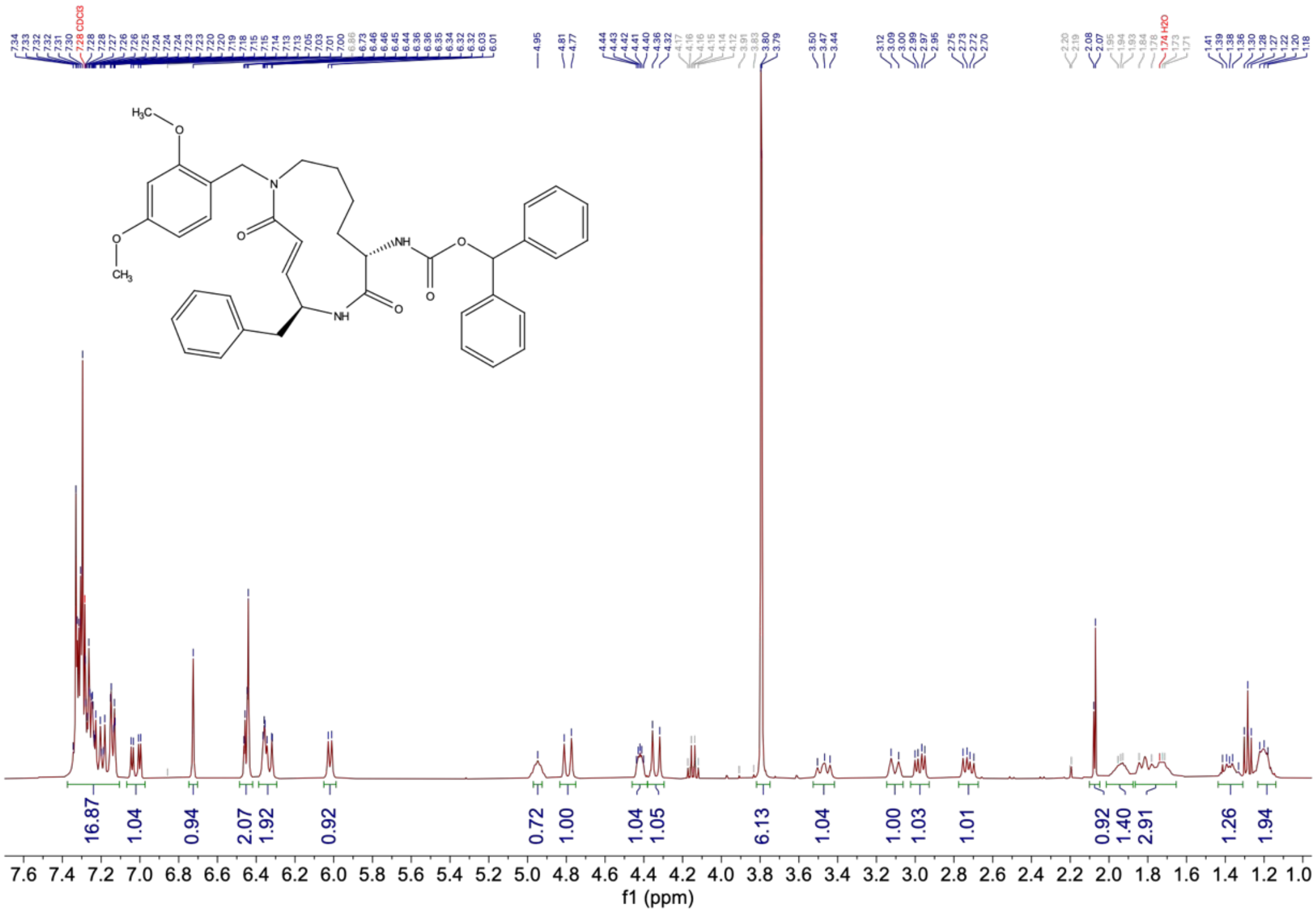

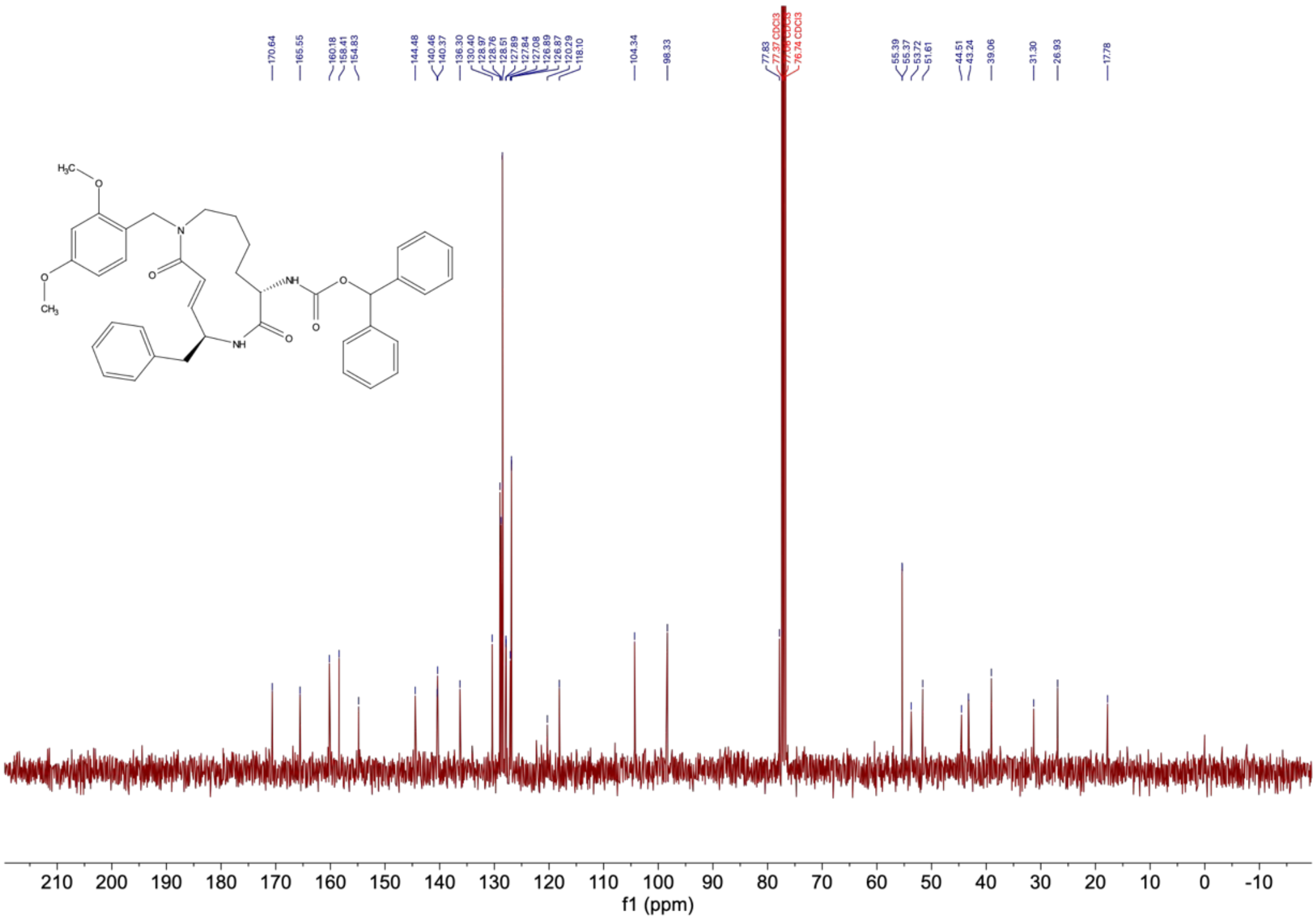

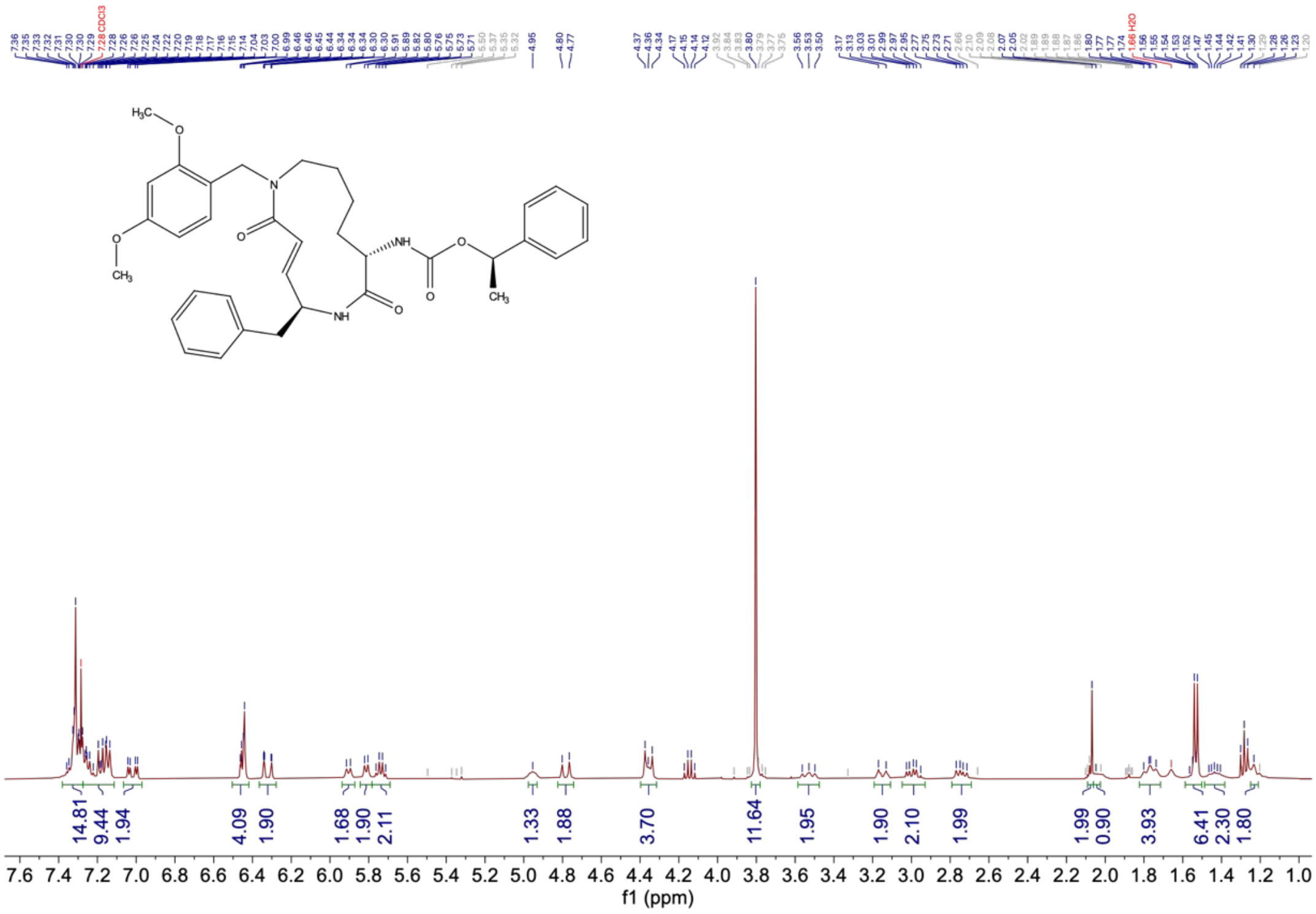

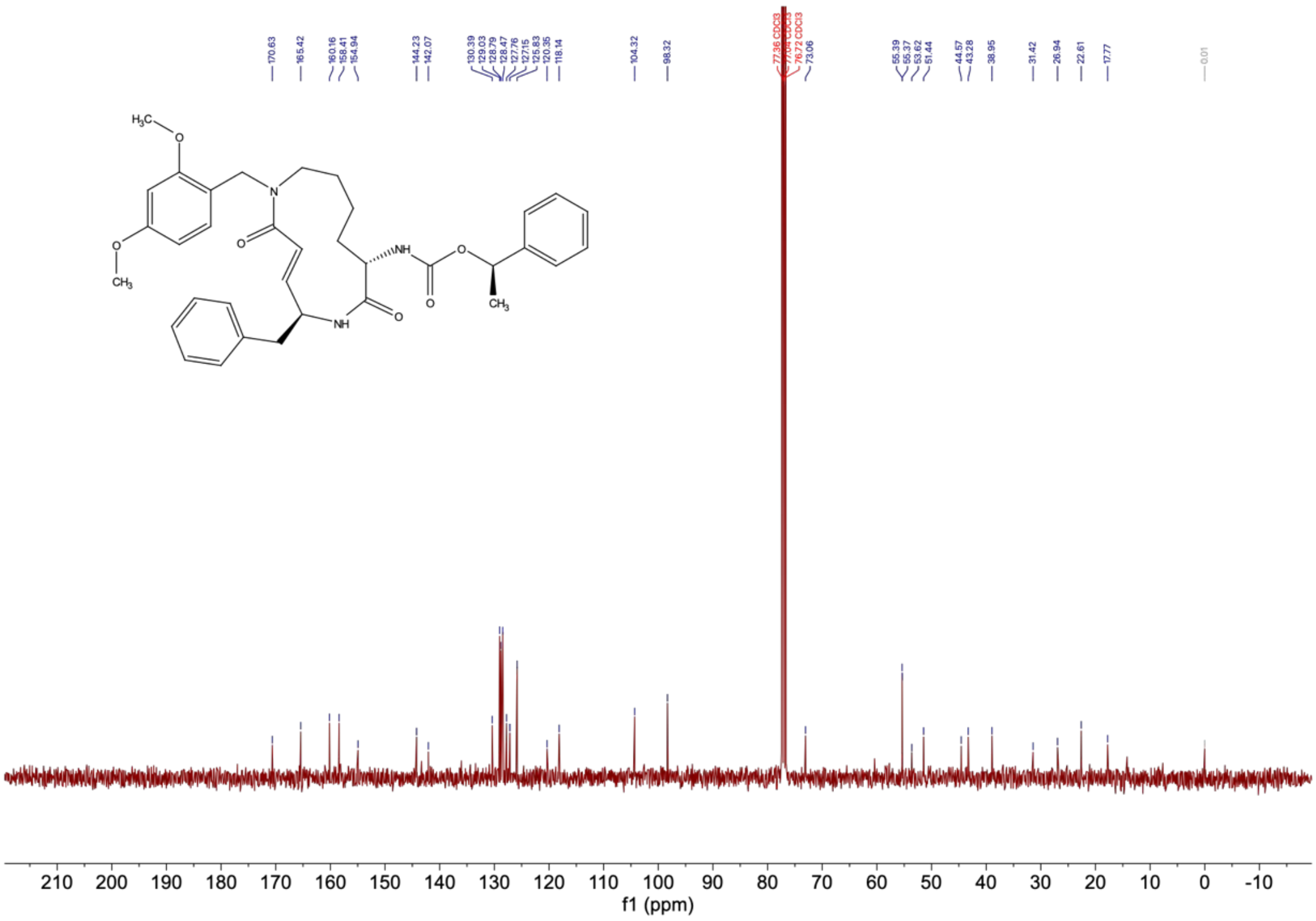

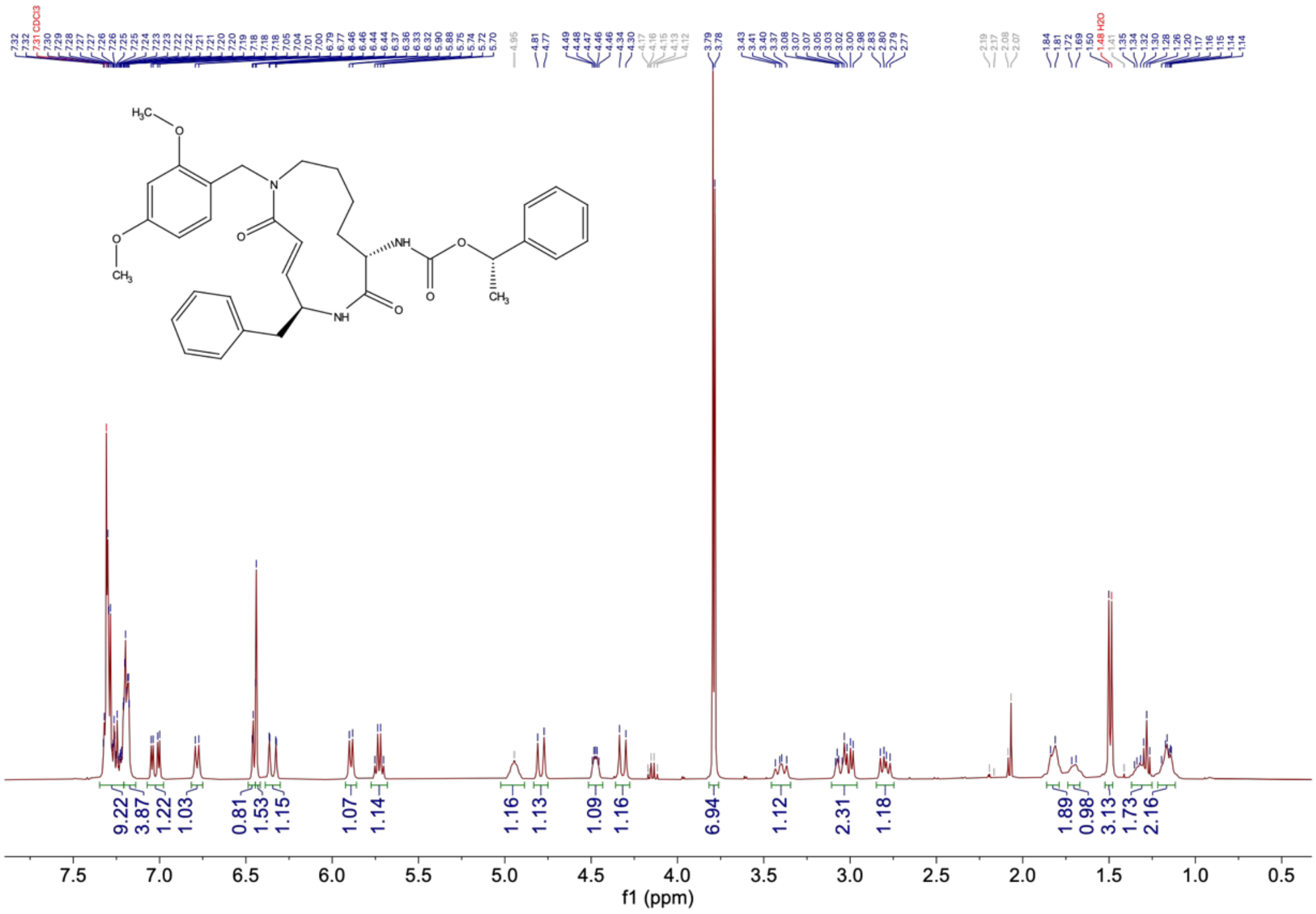

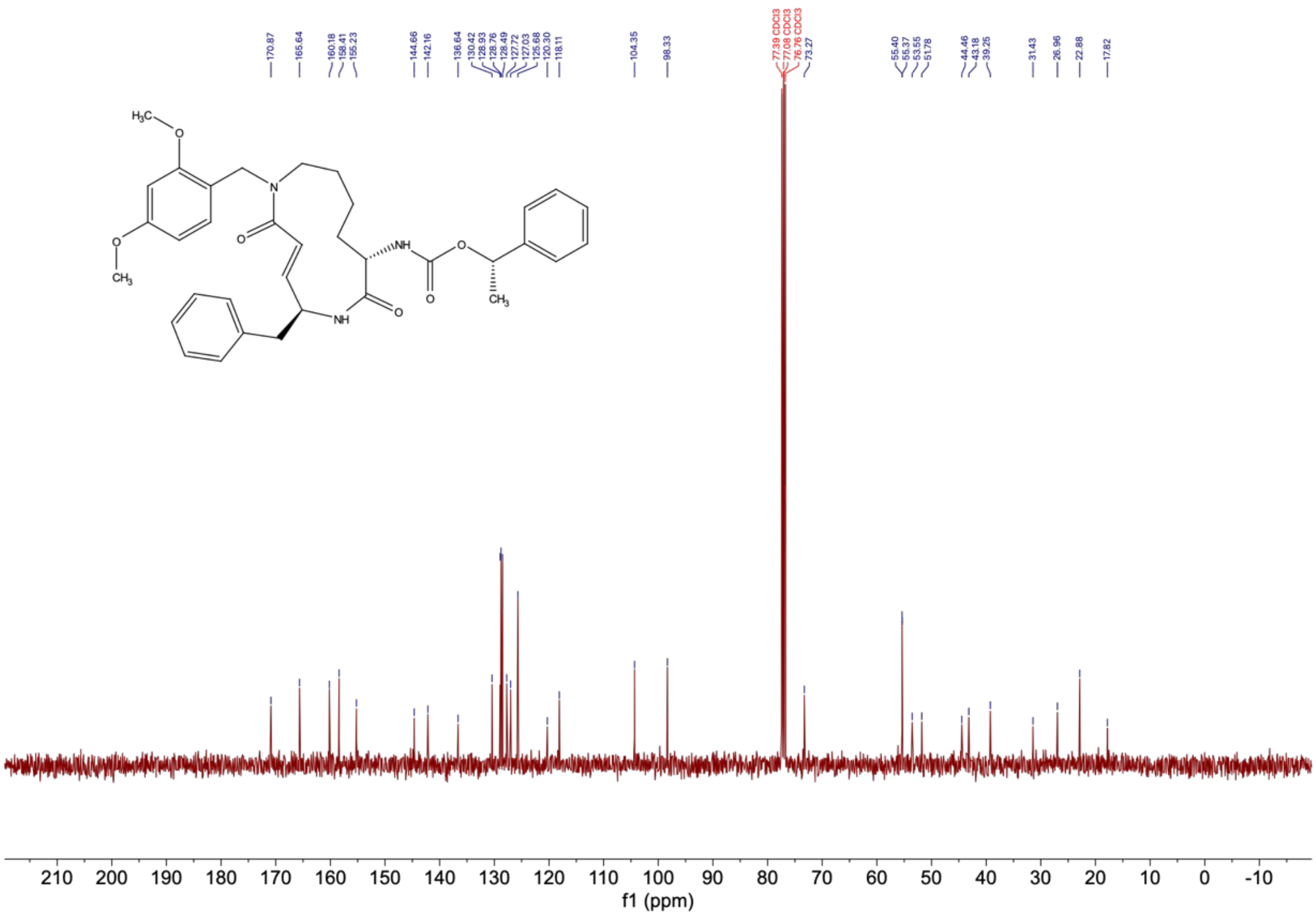

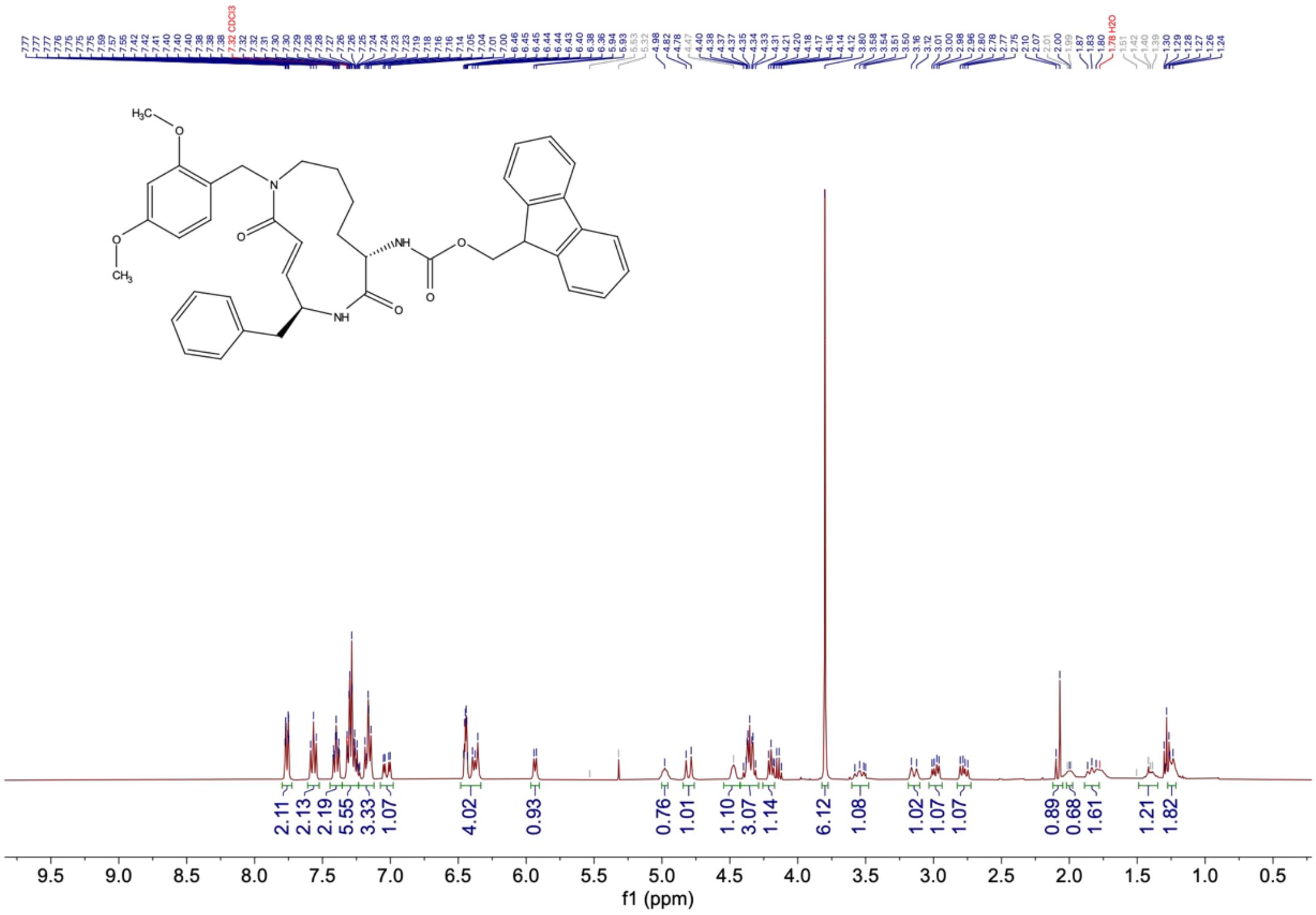
H3C
CH3
N
O
NH
O
O
NH
O
2.11
2.13
2.19
5.55
3.33
1.07
4.02
0.93
0.76
1.01
1.10
3.07
1.14
6.12
1.08
1.02
1.07
1.07
0.89
0.68
1.61
1.21
1.82
f1 (ppm)

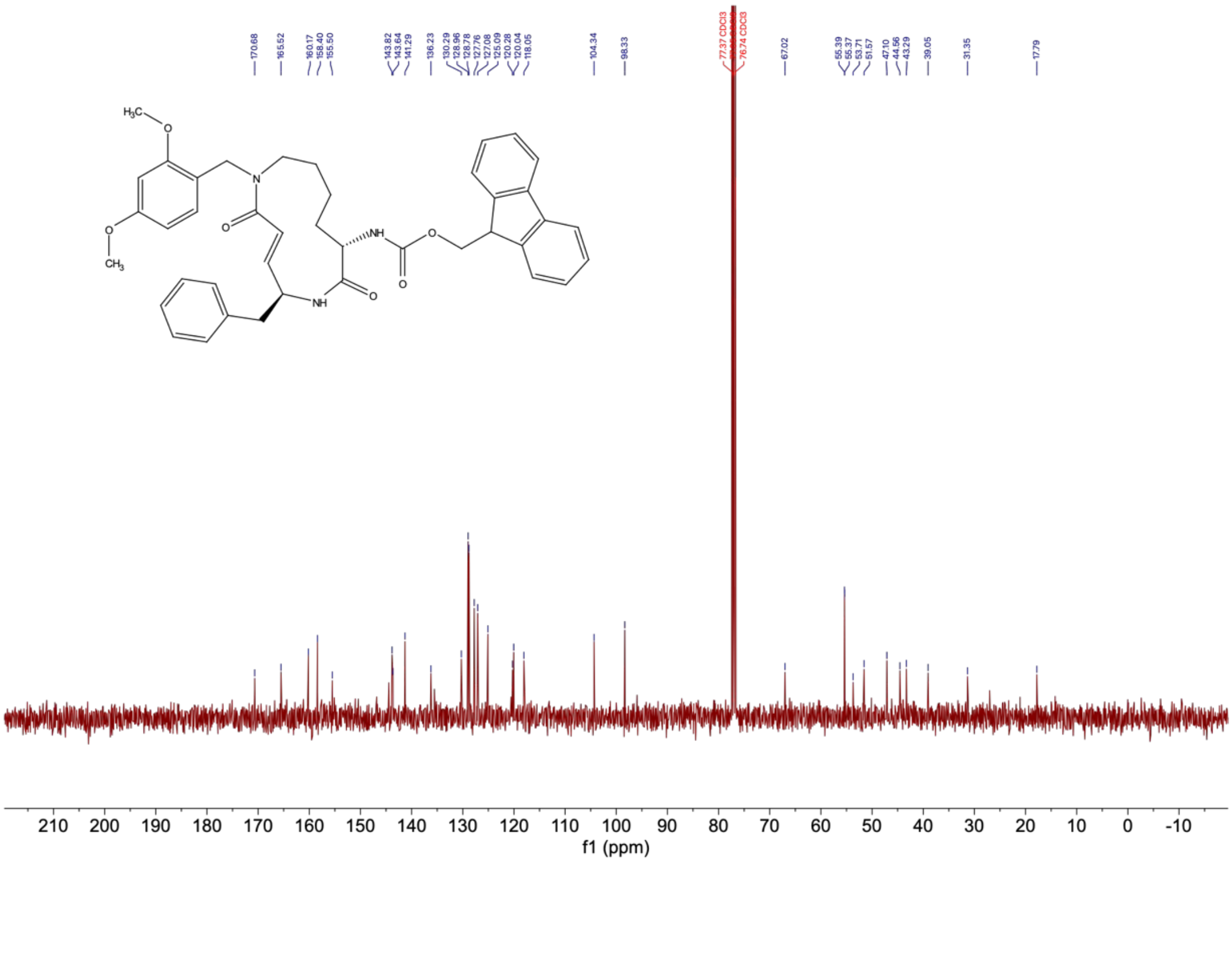
170.68
165.52
160.17
158.40
155.50
143.82
143.64
141.29
136.23
130.29
128.96
128.78
127.76
127.08
125.09
120.28
120.04
118.05
104.34
98.33
77.37 CDCl3
76.74 CDCl3
67.02
55.39
55.37
53.71
51.57
47.10
44.56
43.29
39.05
31.35
17.79
H3C
CH3
N
O
NH
O
O
NH
O
f1 (ppm)

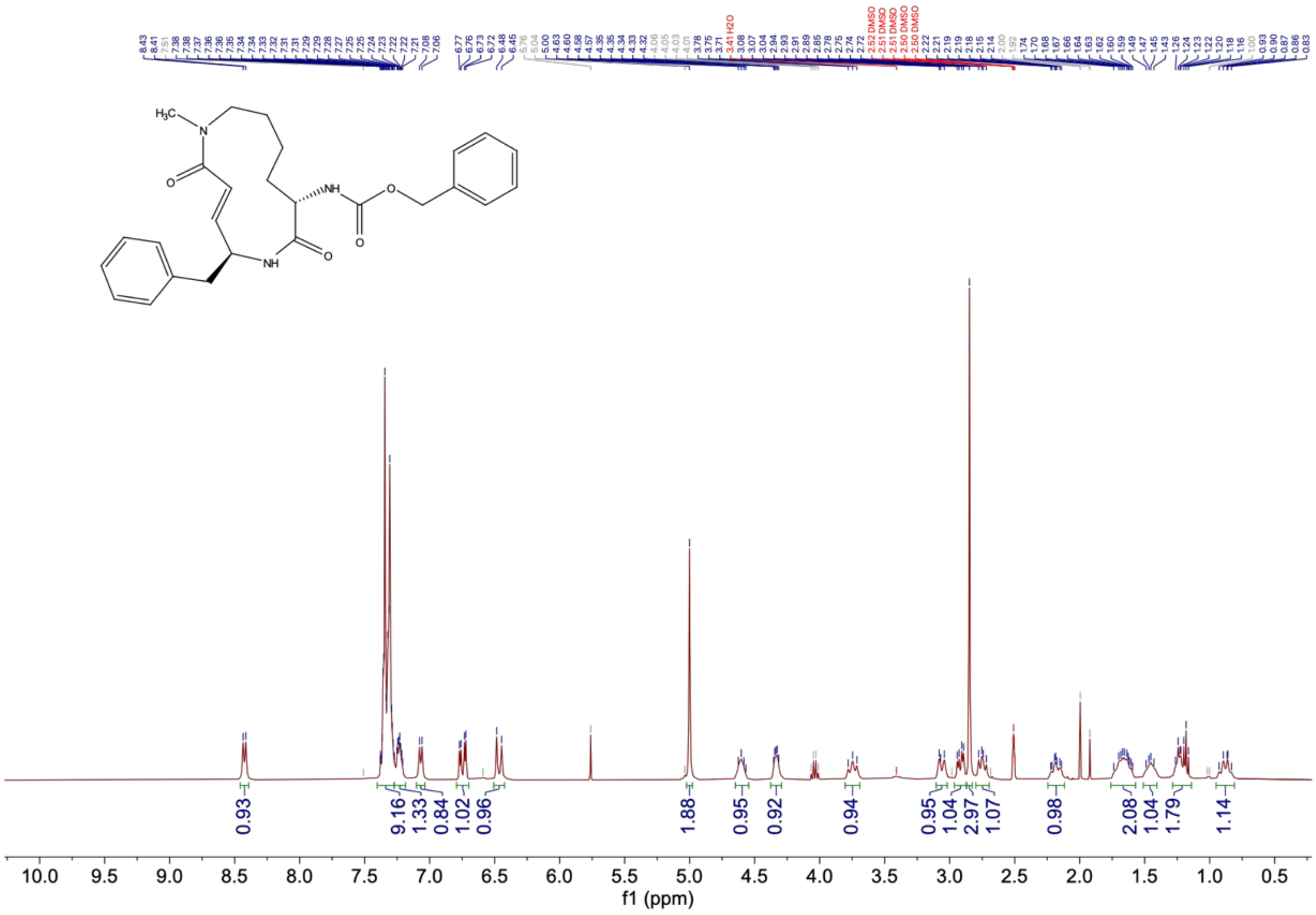
H3C
N
O
NH
O
NH
O
O
0.93
9.16
1.33
0.84
1.02
0.96
1.88
0.95
0.92
0.94
0.95
1.04
2.97
1.07
0.98
2.08
1.04
1.79
1.14
10.0 9.5 9.0 8.5 8.0 7.5 7.0 6.5 6.0 5.5 5.0 4.5 4.0 3.5 3.0 2.5 2.0 1.5 1.0 0.5
f1 (ppm)

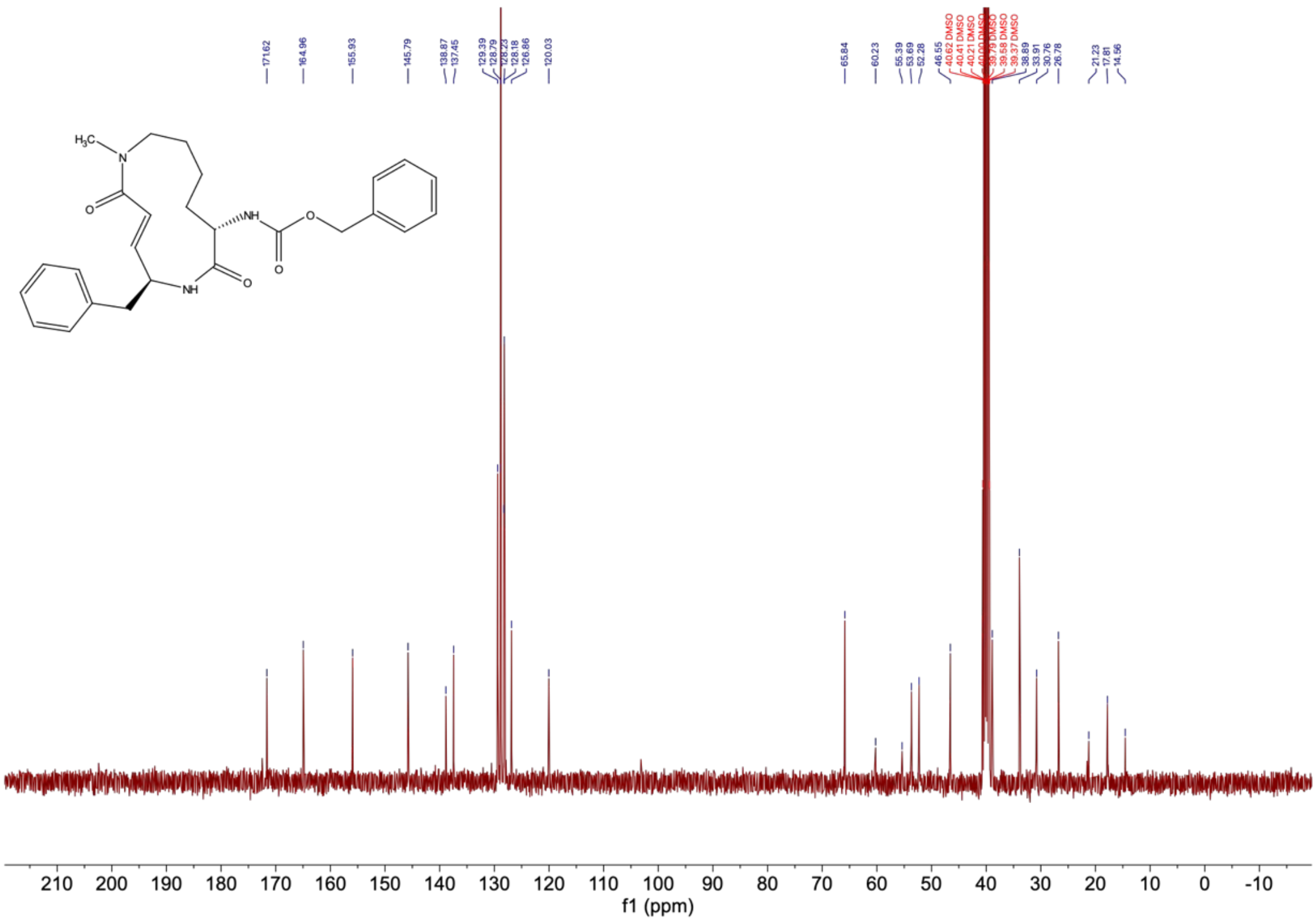
H3C
N
O
NH
O
NH
O
O
210 200 190 180 170 160 150 140 130 120 110 100 90 80 70 60 50 40 30 20 10 0 -10
f1 (ppm)

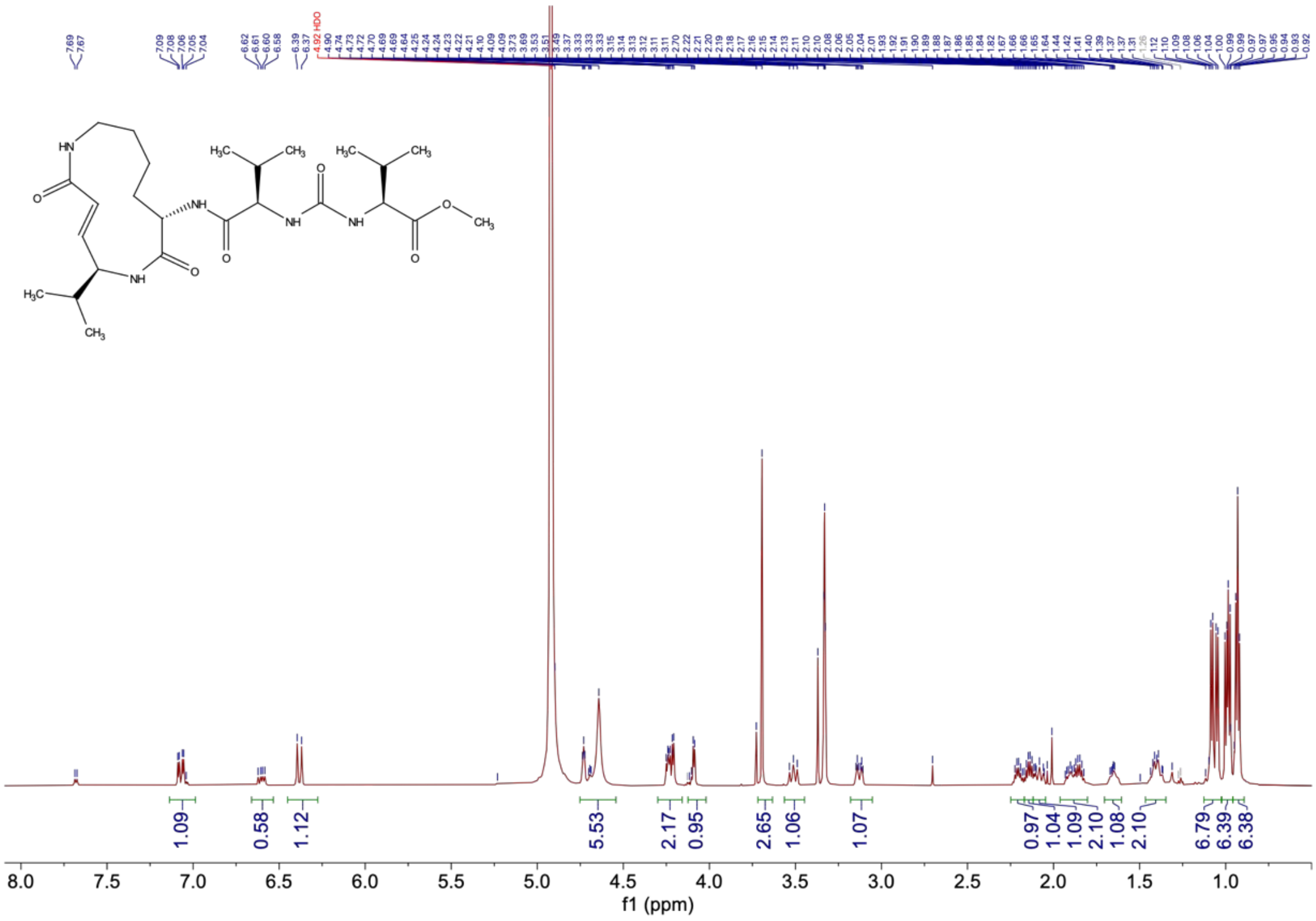
1.09
0.58
1.12
5.53
2.17
0.95
2.65
1.06
1.07
0.97
1.04
1.09
2.10
1.08
2.10
6.79
6.39
6.38
8.0 7.5 7.0 6.5 6.0 5.5 5.0 4.5 4.0 3.5 3.0 2.5 2.0 1.5 1.0
f1 (ppm)

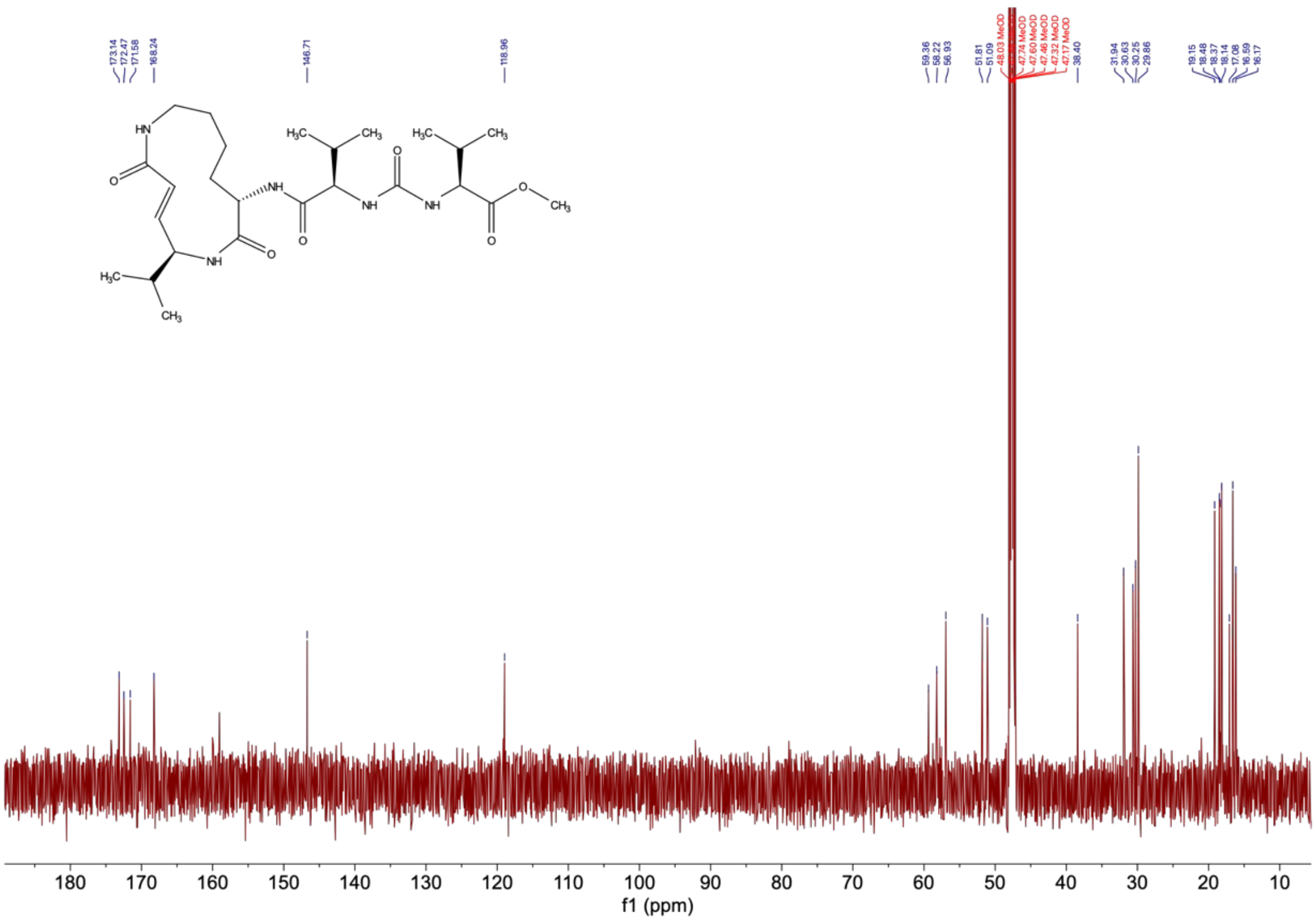
180 170 160 150 140 130 120 110 100 90 80 70 60 50 40 30 20 10
f1 (ppm)

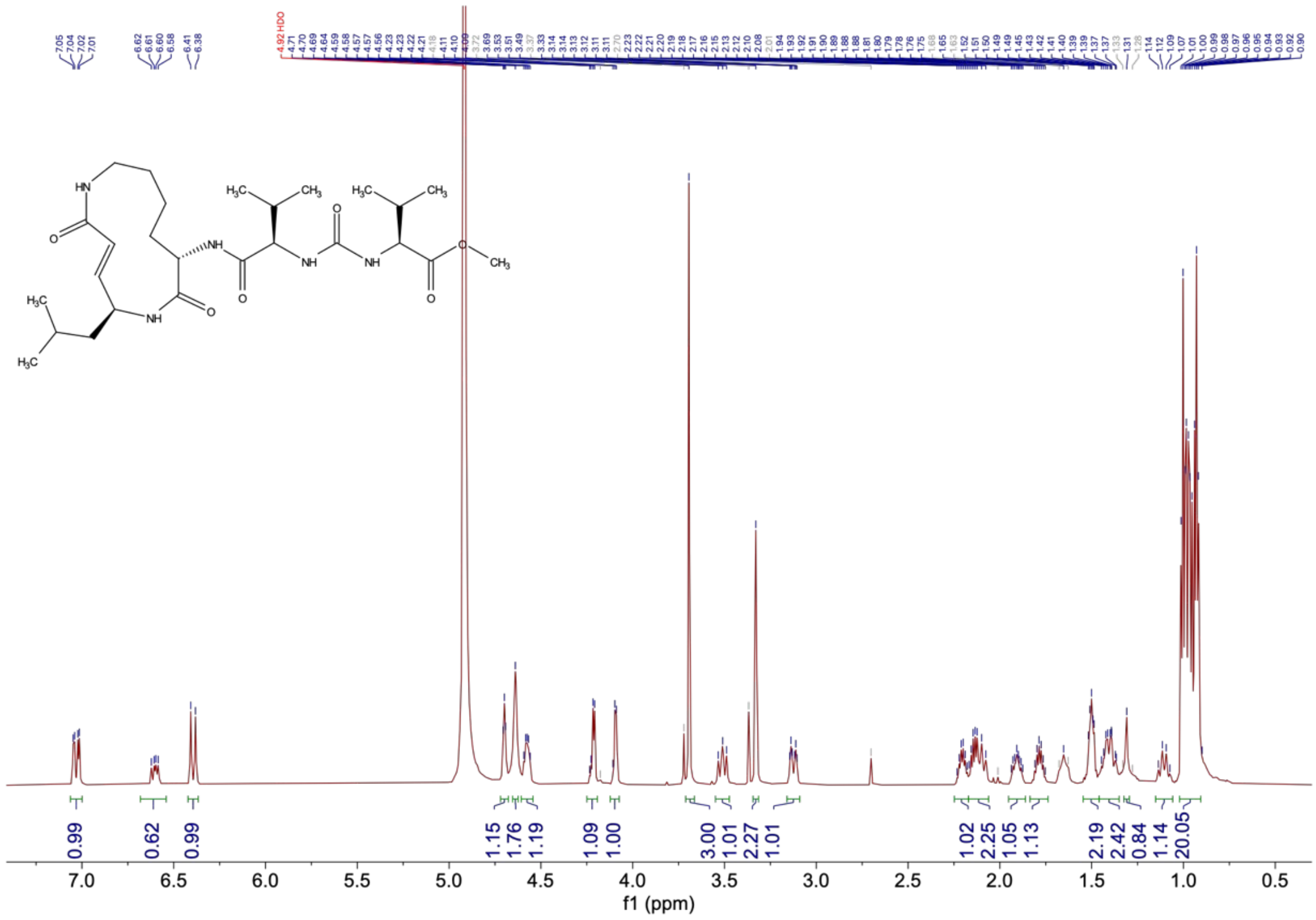

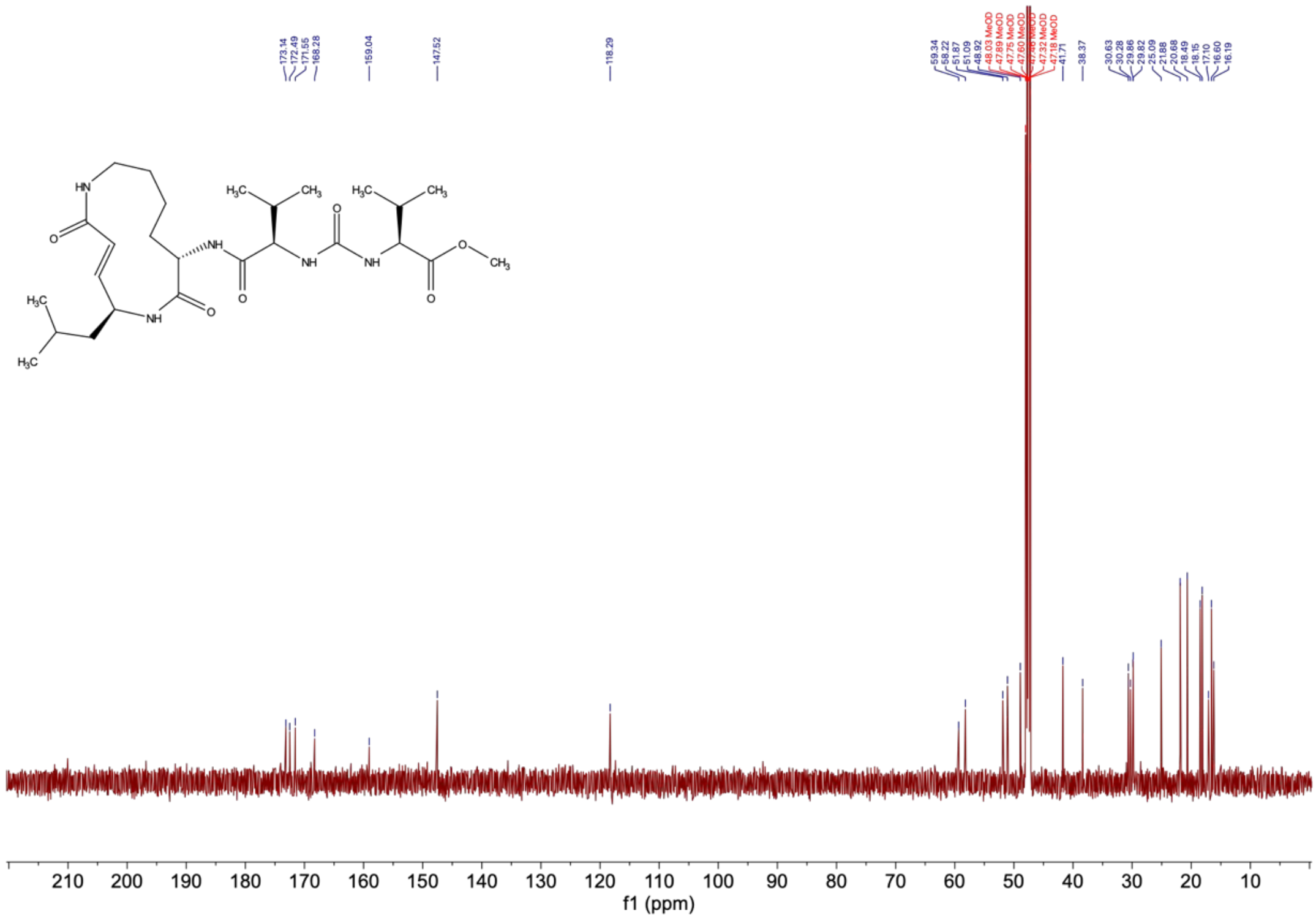

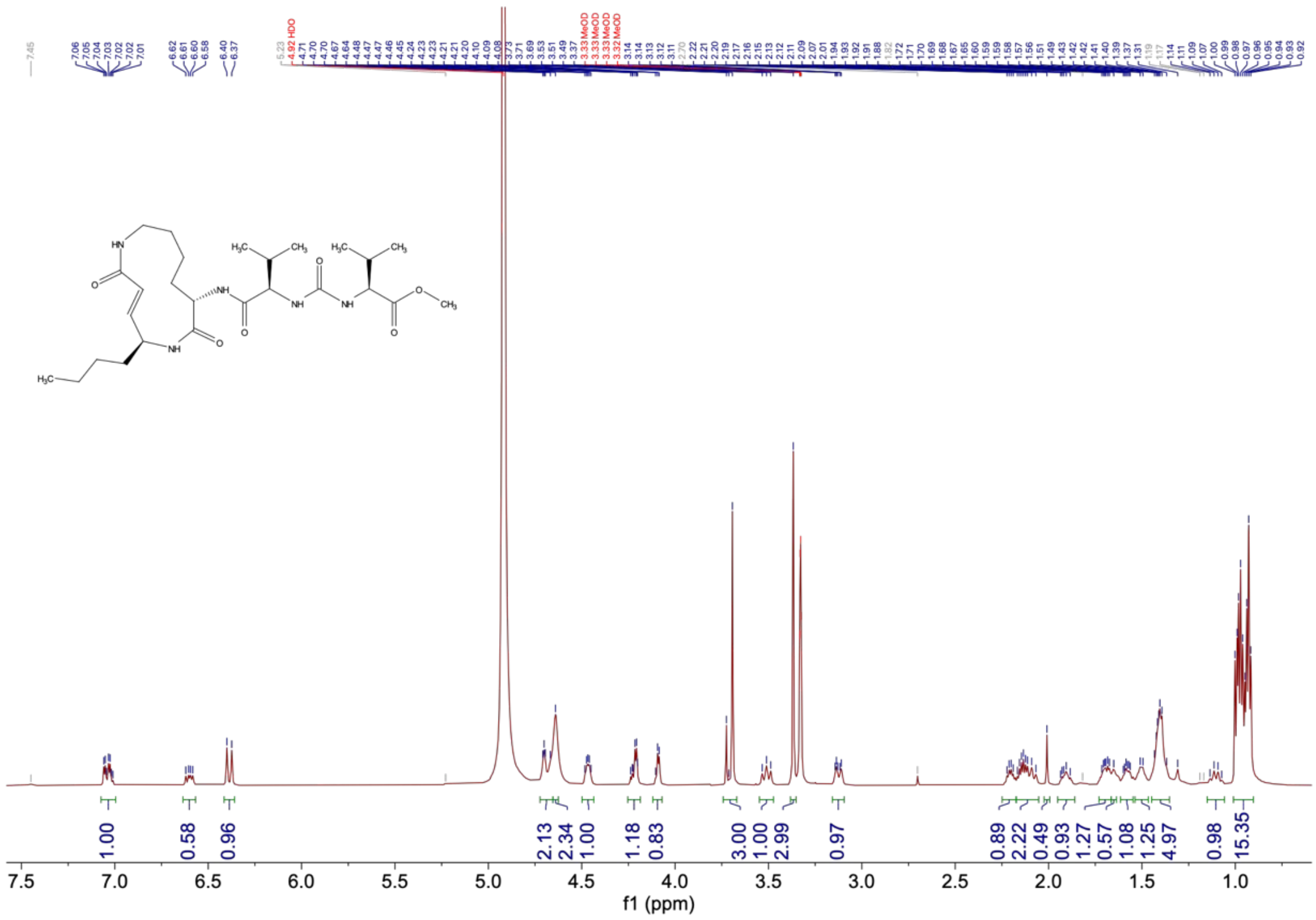

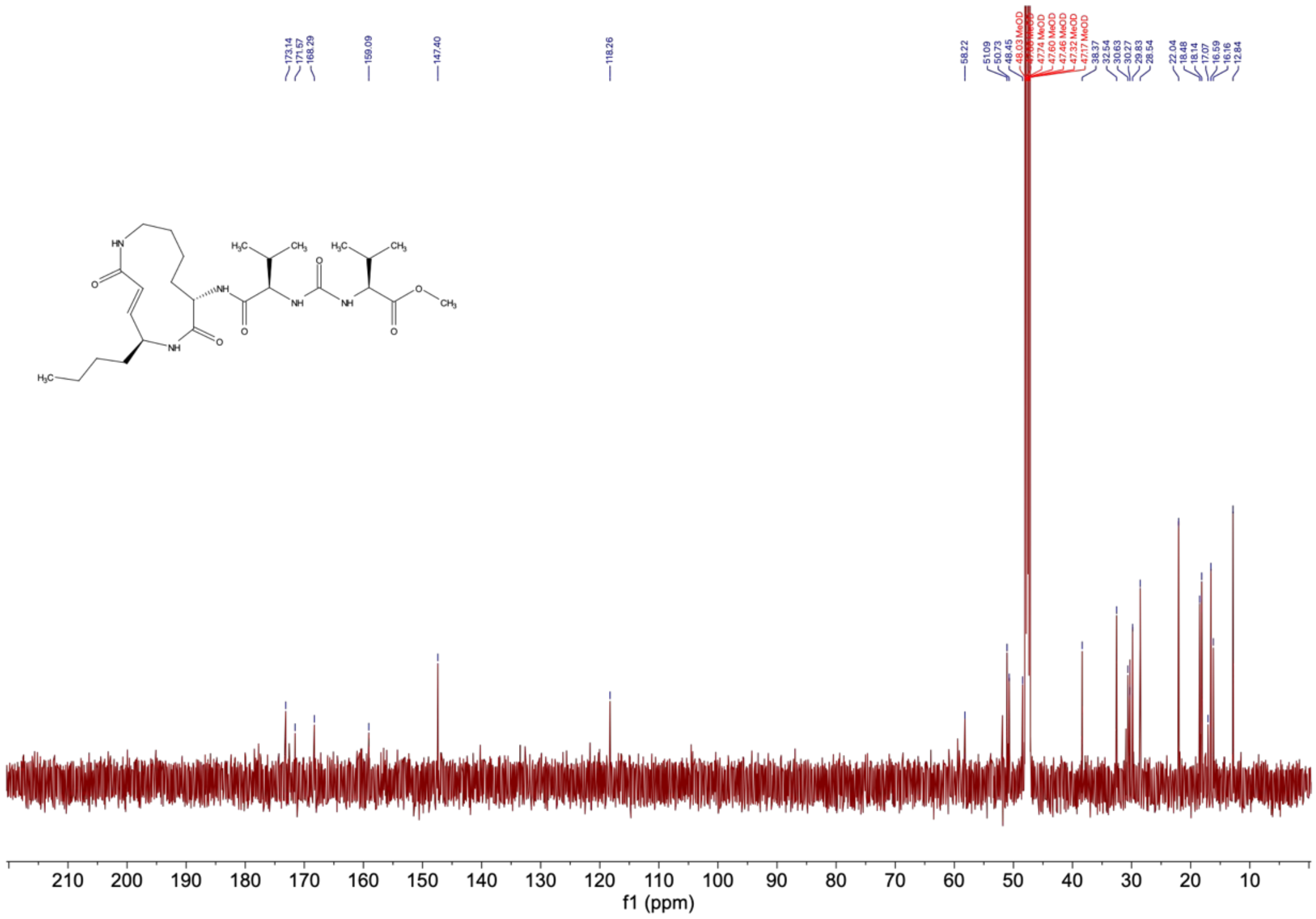

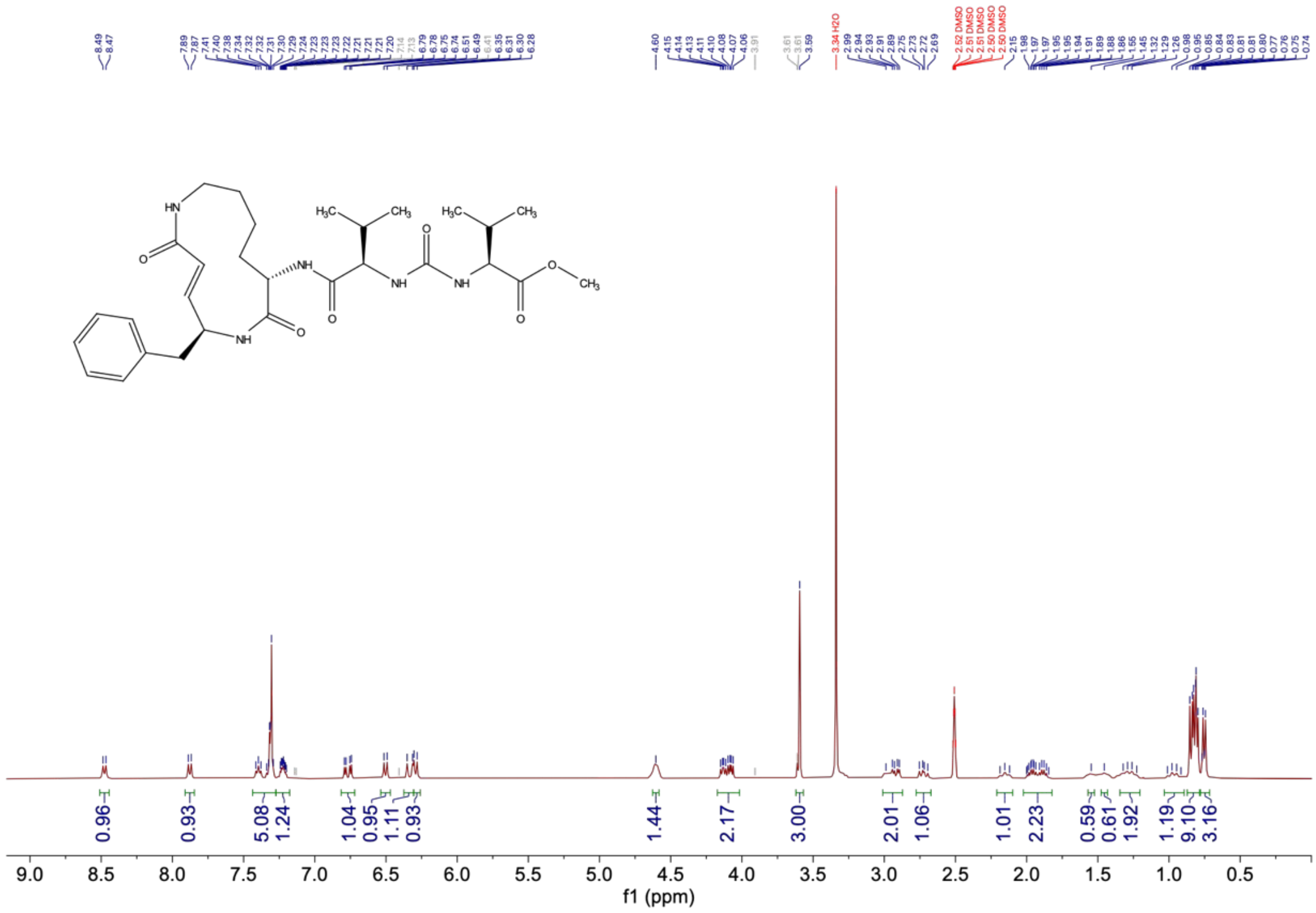

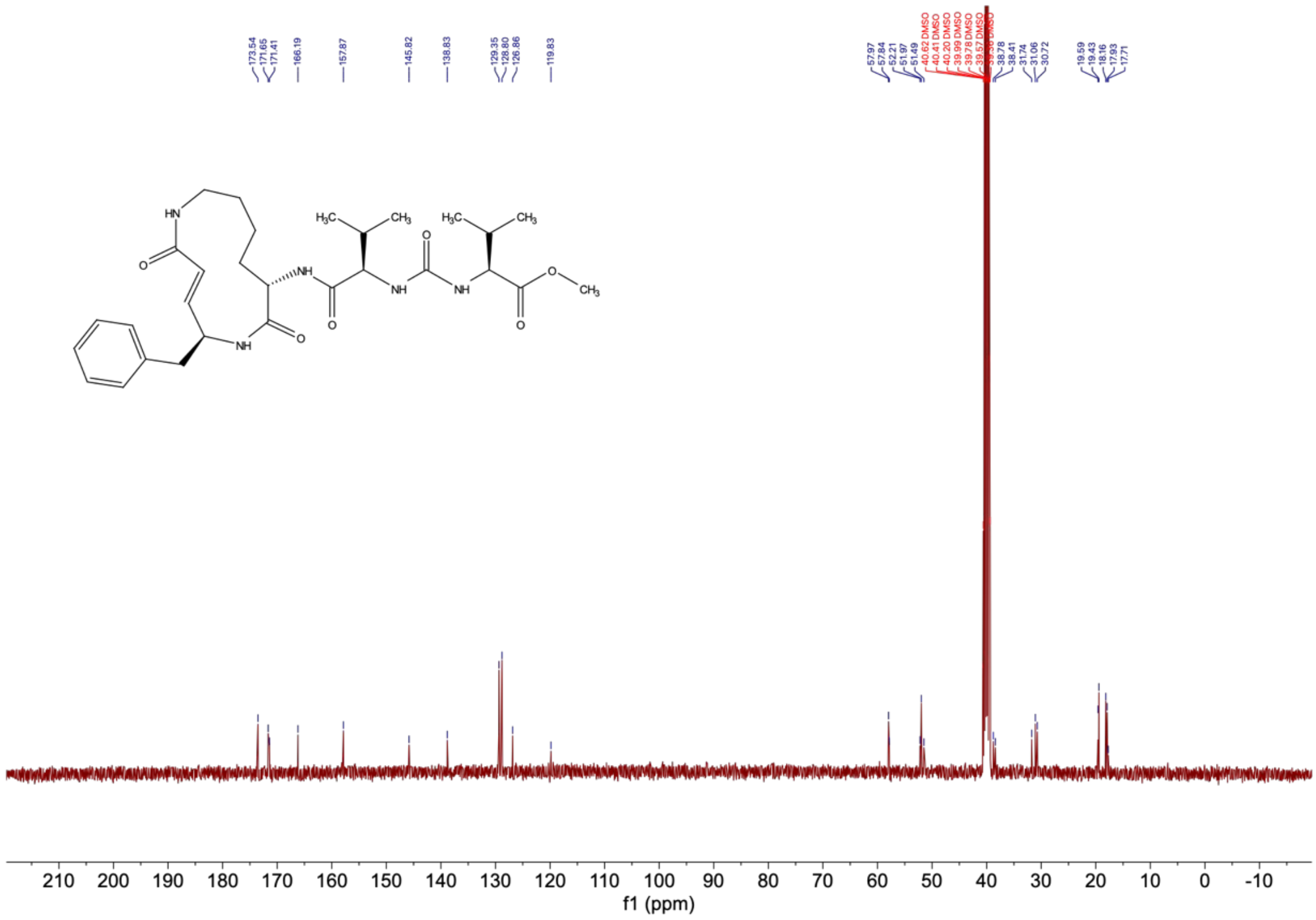

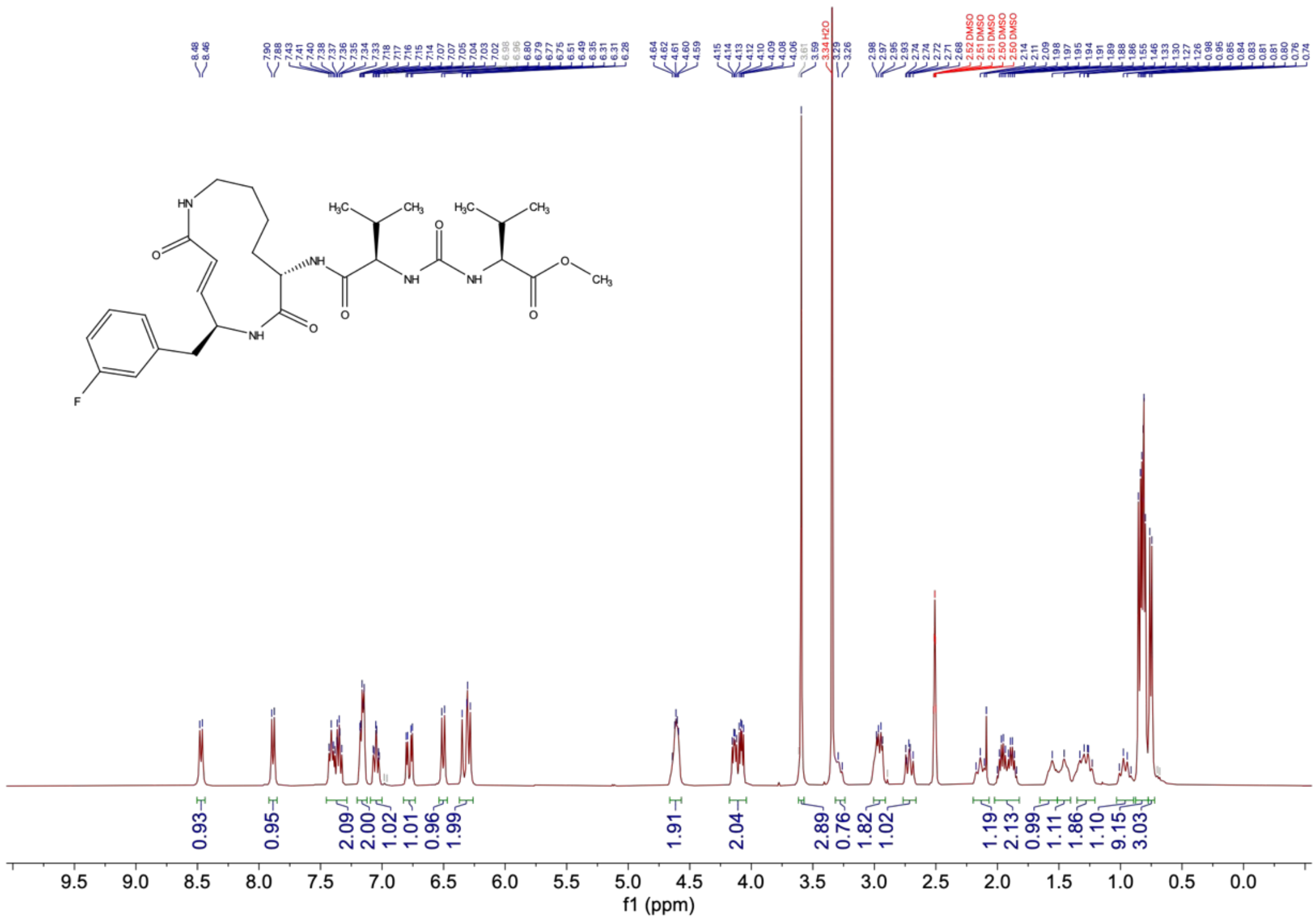
f1 (ppm)

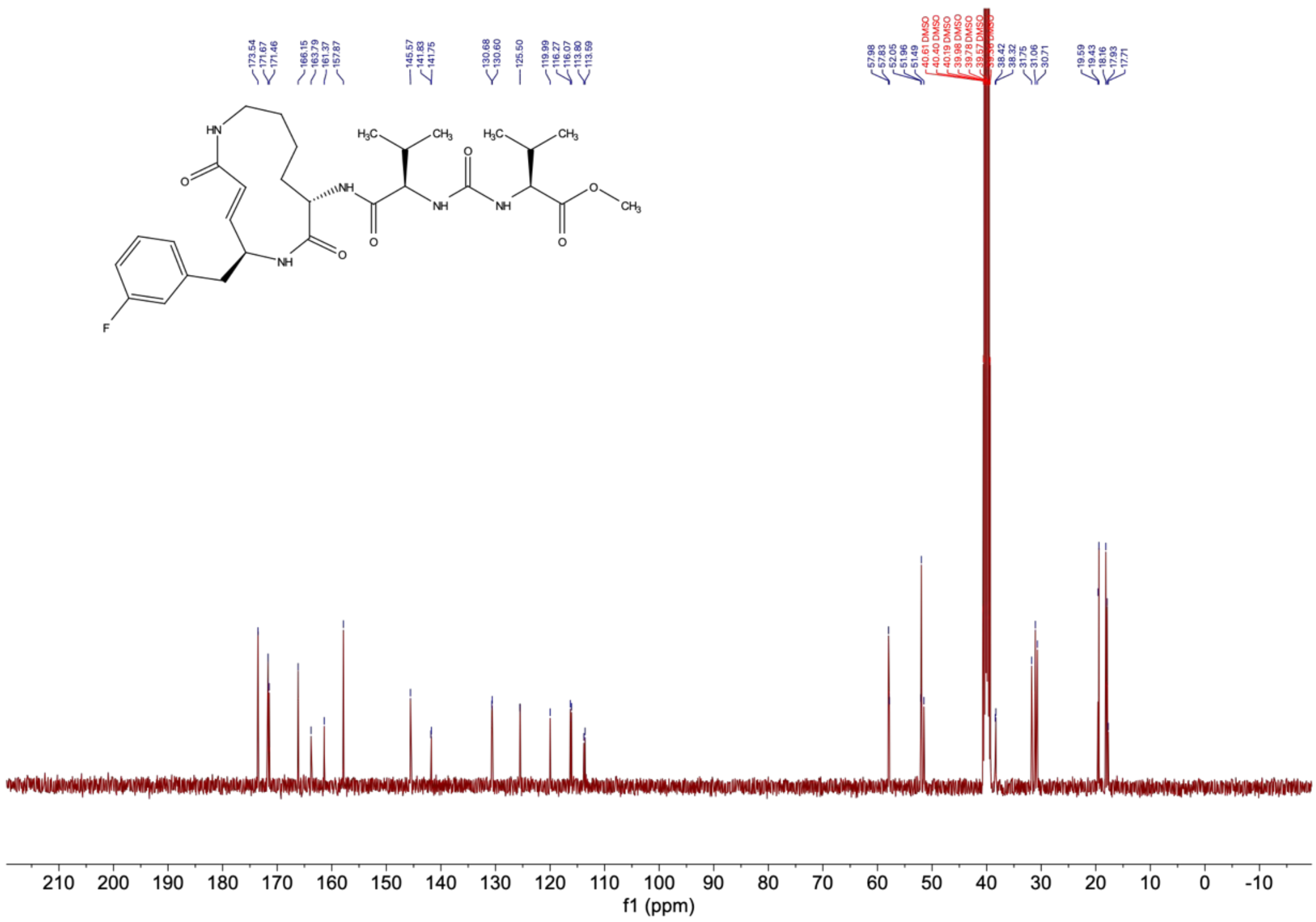
f1 (ppm)

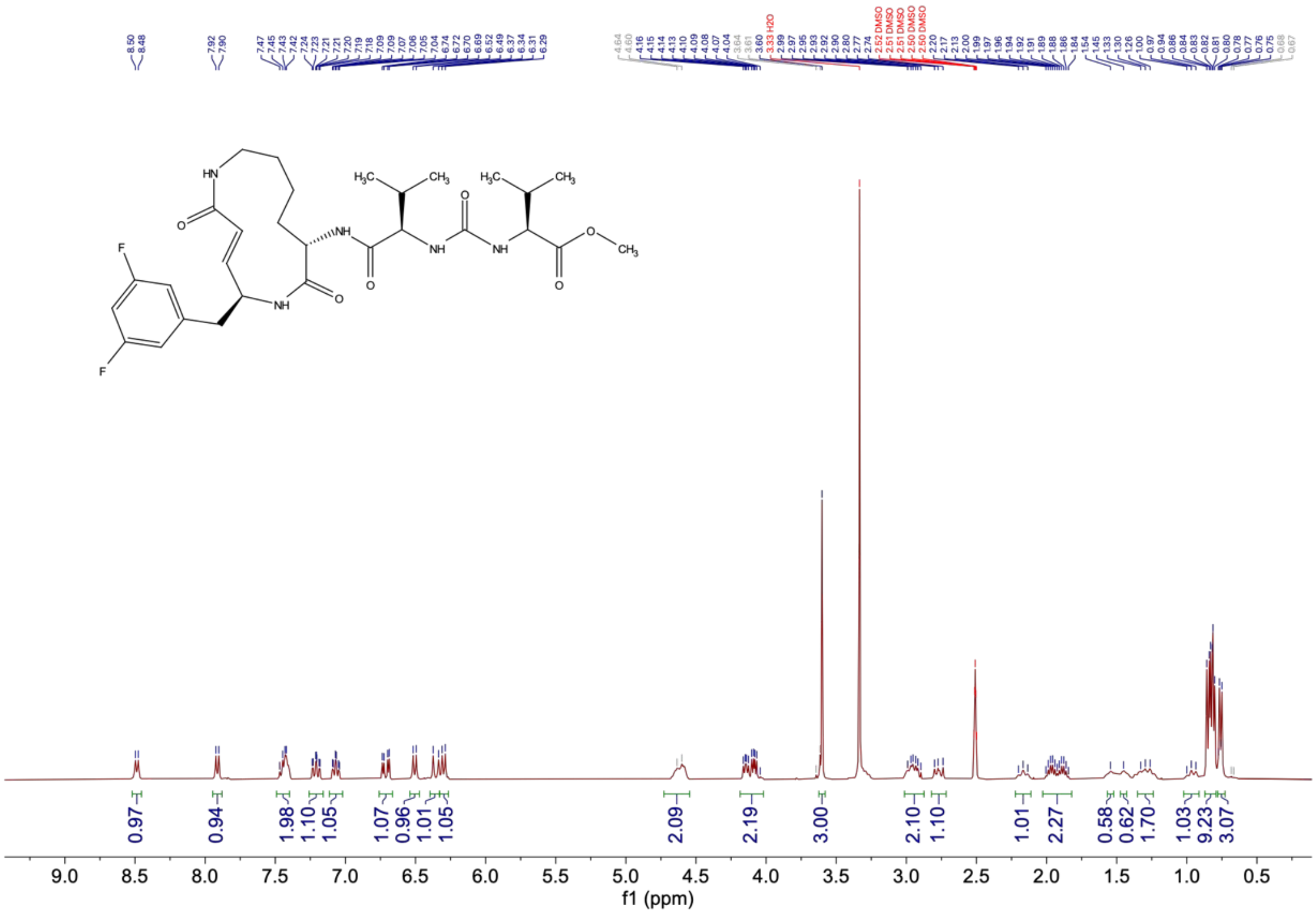

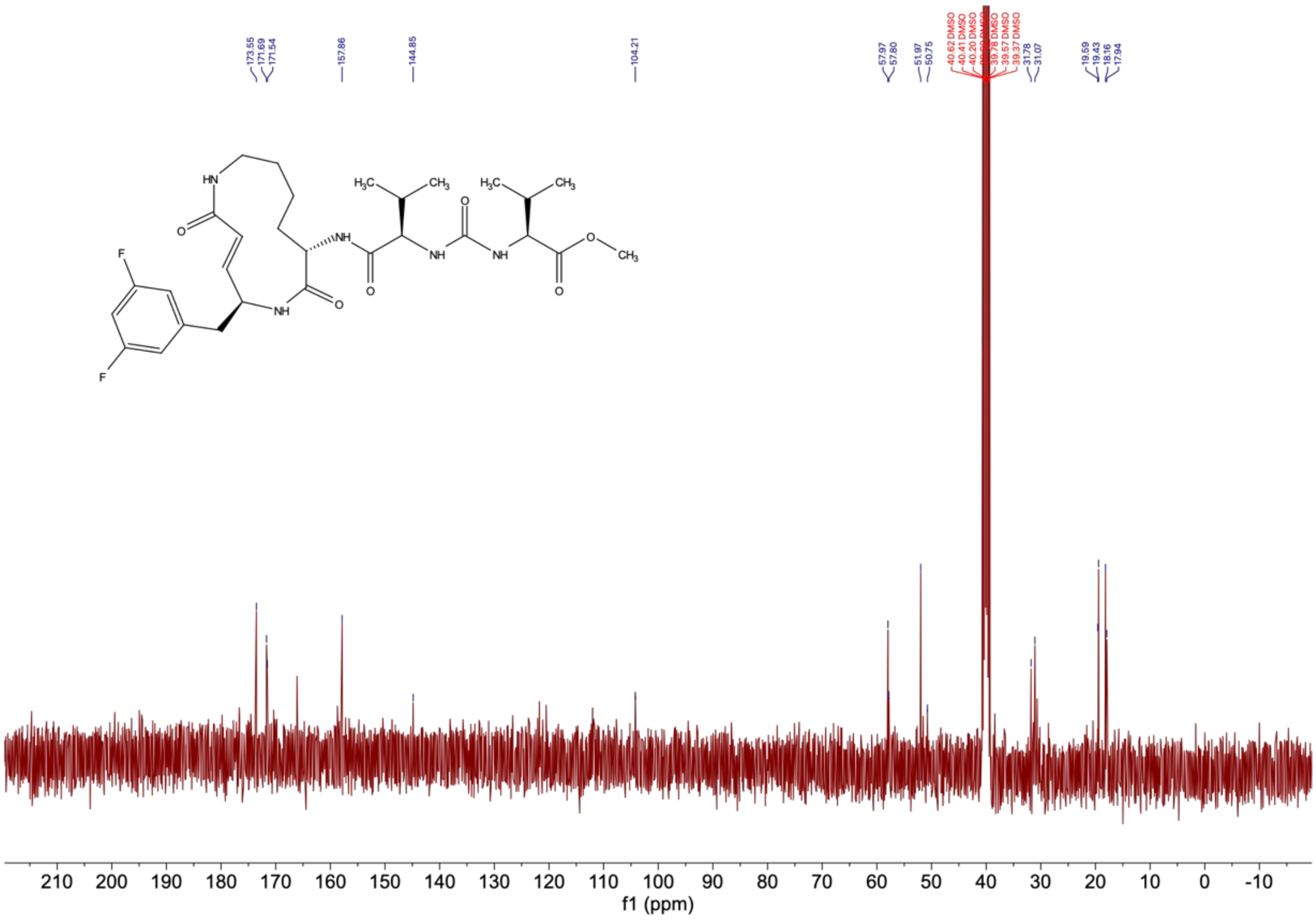

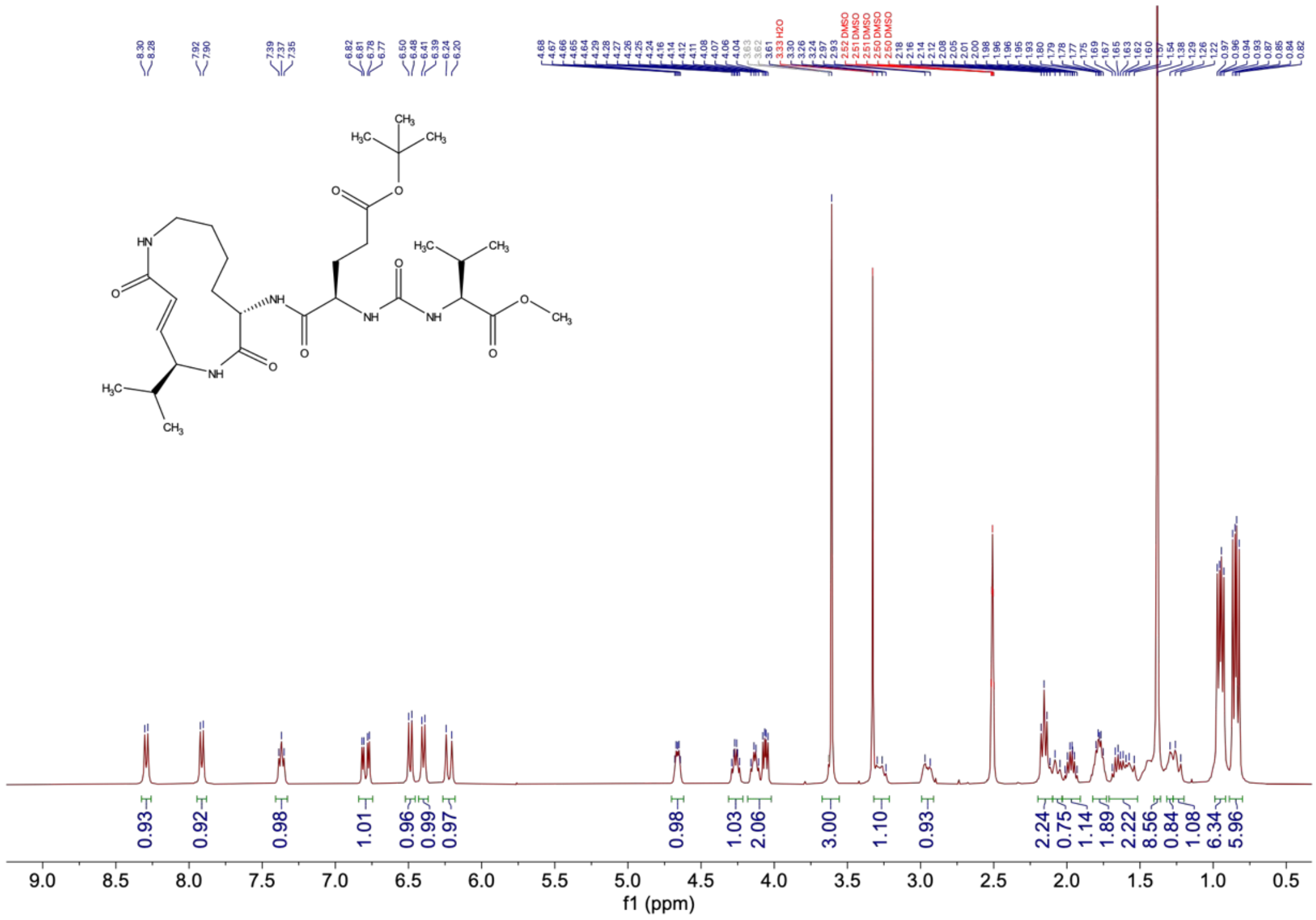

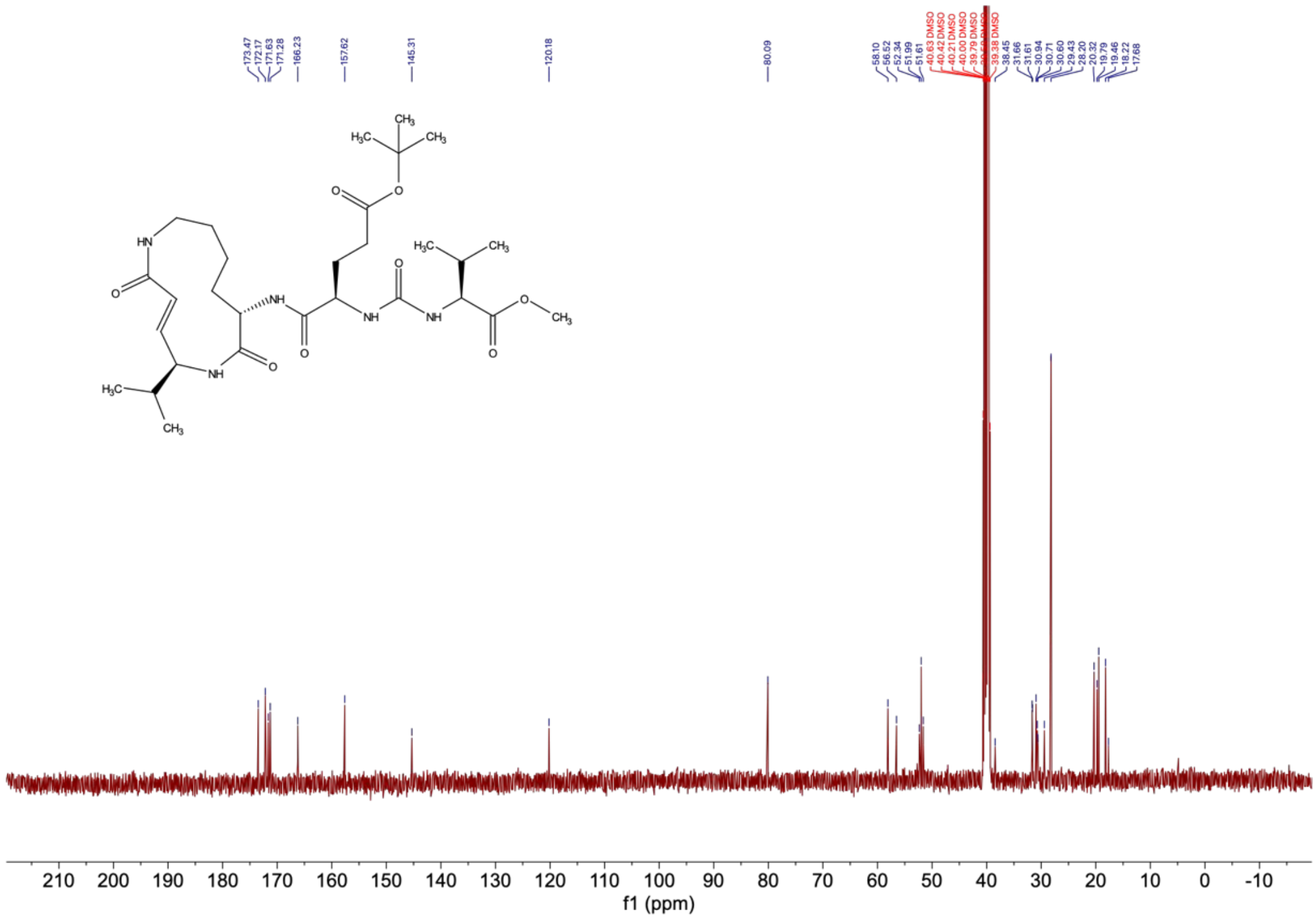

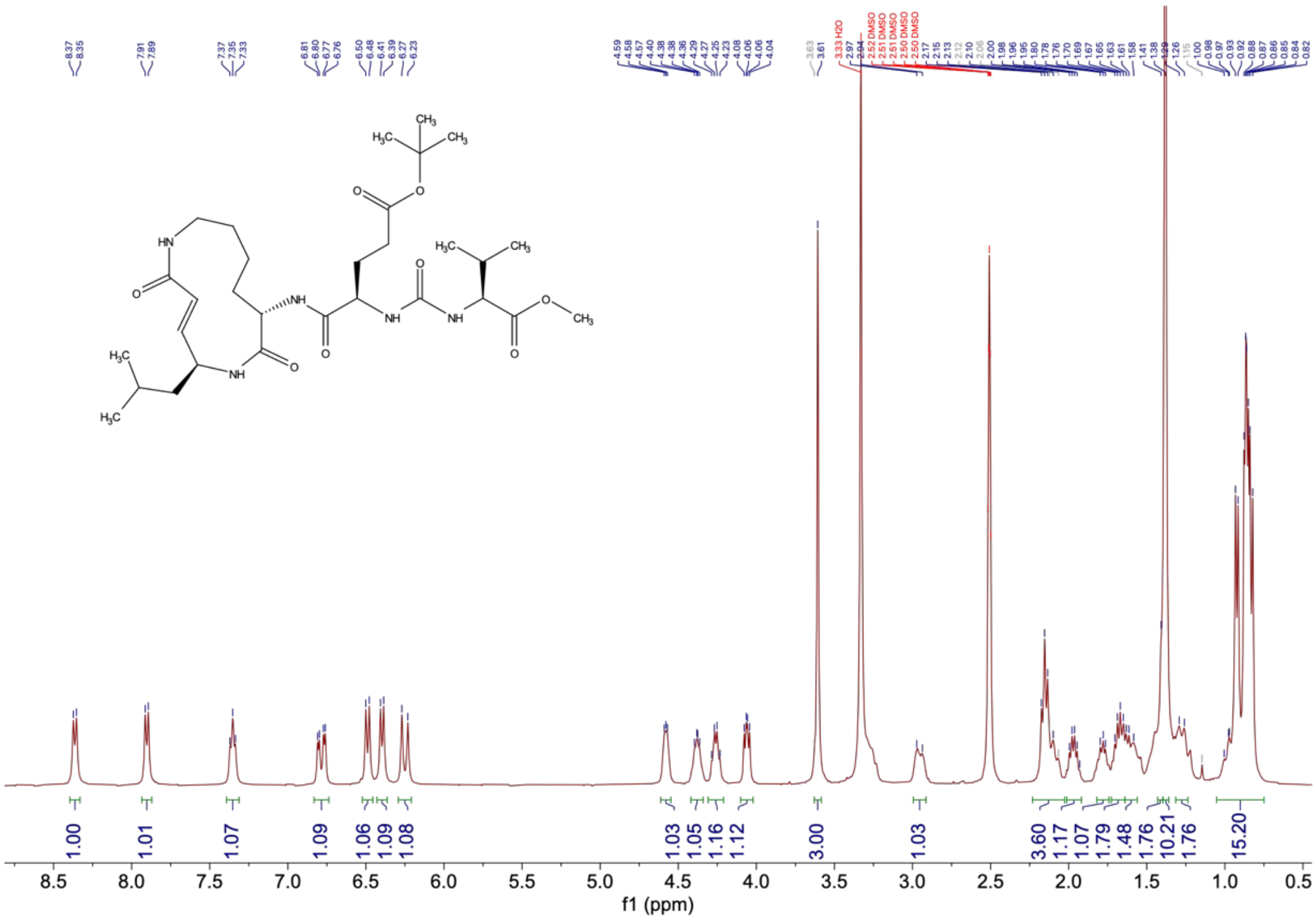

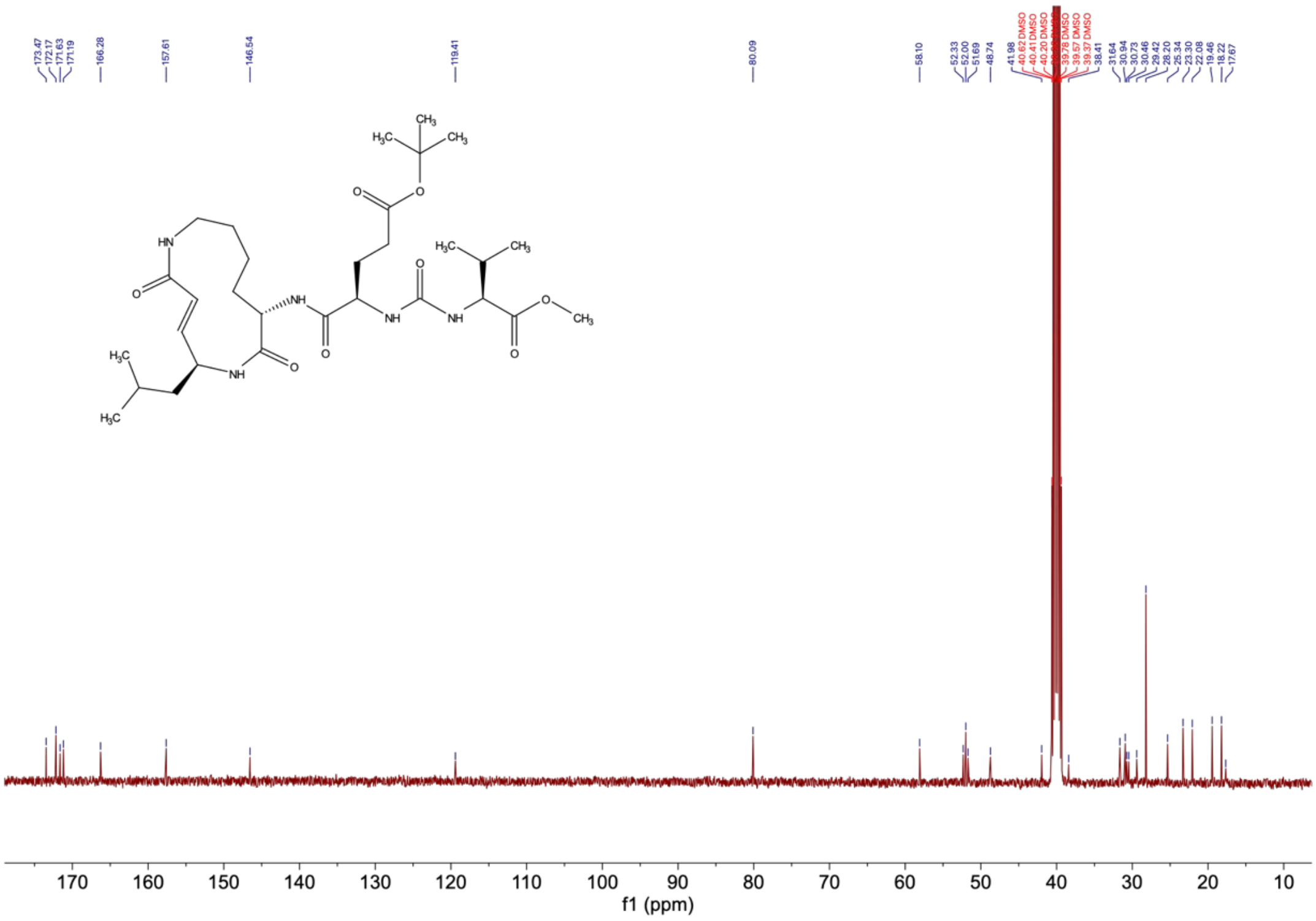

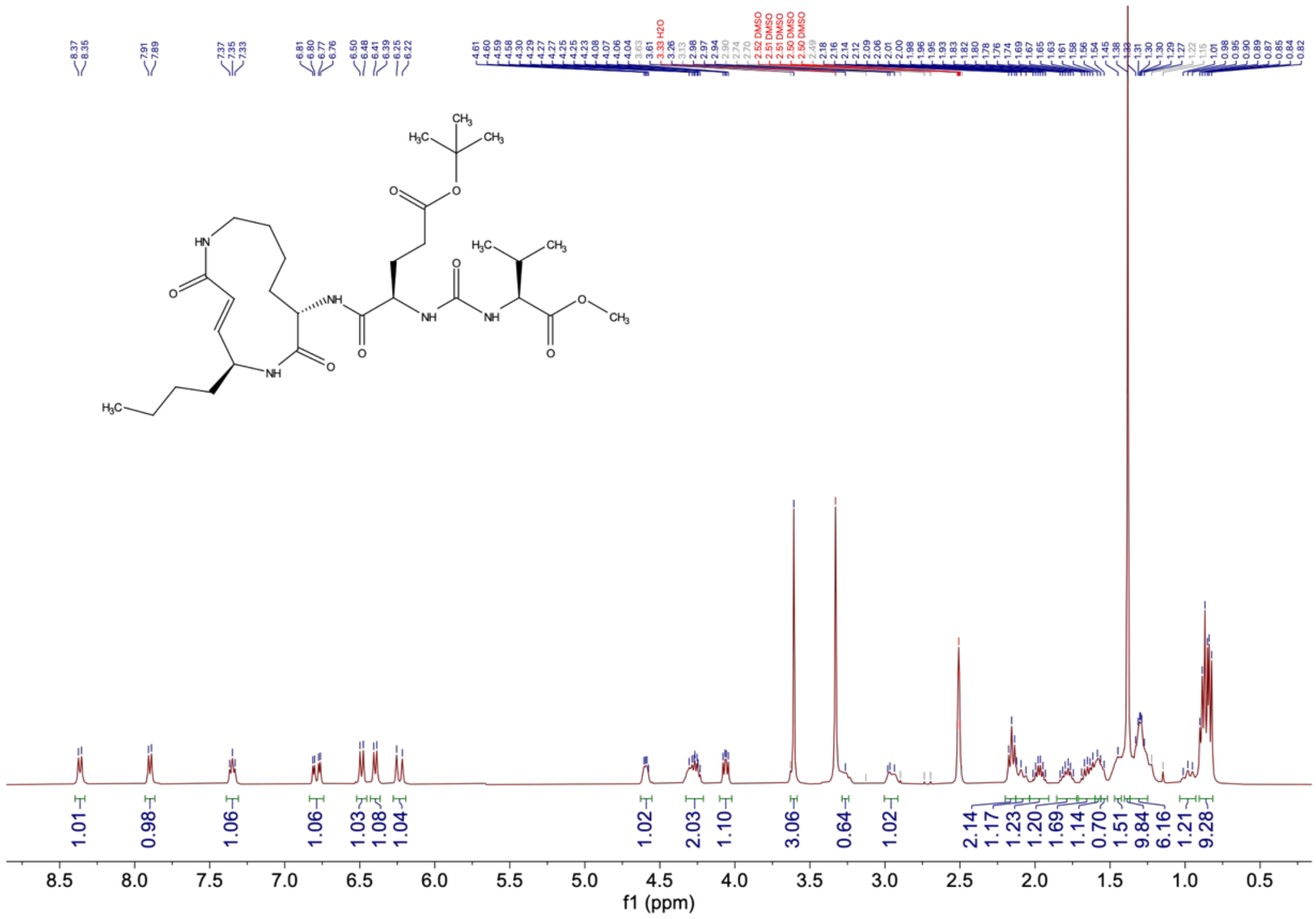

1.01
0.98
1.06
1.06
1.03
1.08
1.04
1.02
2.03
1.10
3.06
0.64
1.02
2.14
1.17
1.23
1.20
1.69
1.14
0.70
1.51
9.84
6.16
1.21
9.28
8.5 8.0 7.5 7.0 6.5 6.0 5.5 5.0 4.5 4.0 3.5 3.0 2.5 2.0 1.5 1.0 0.5
f1 (ppm)

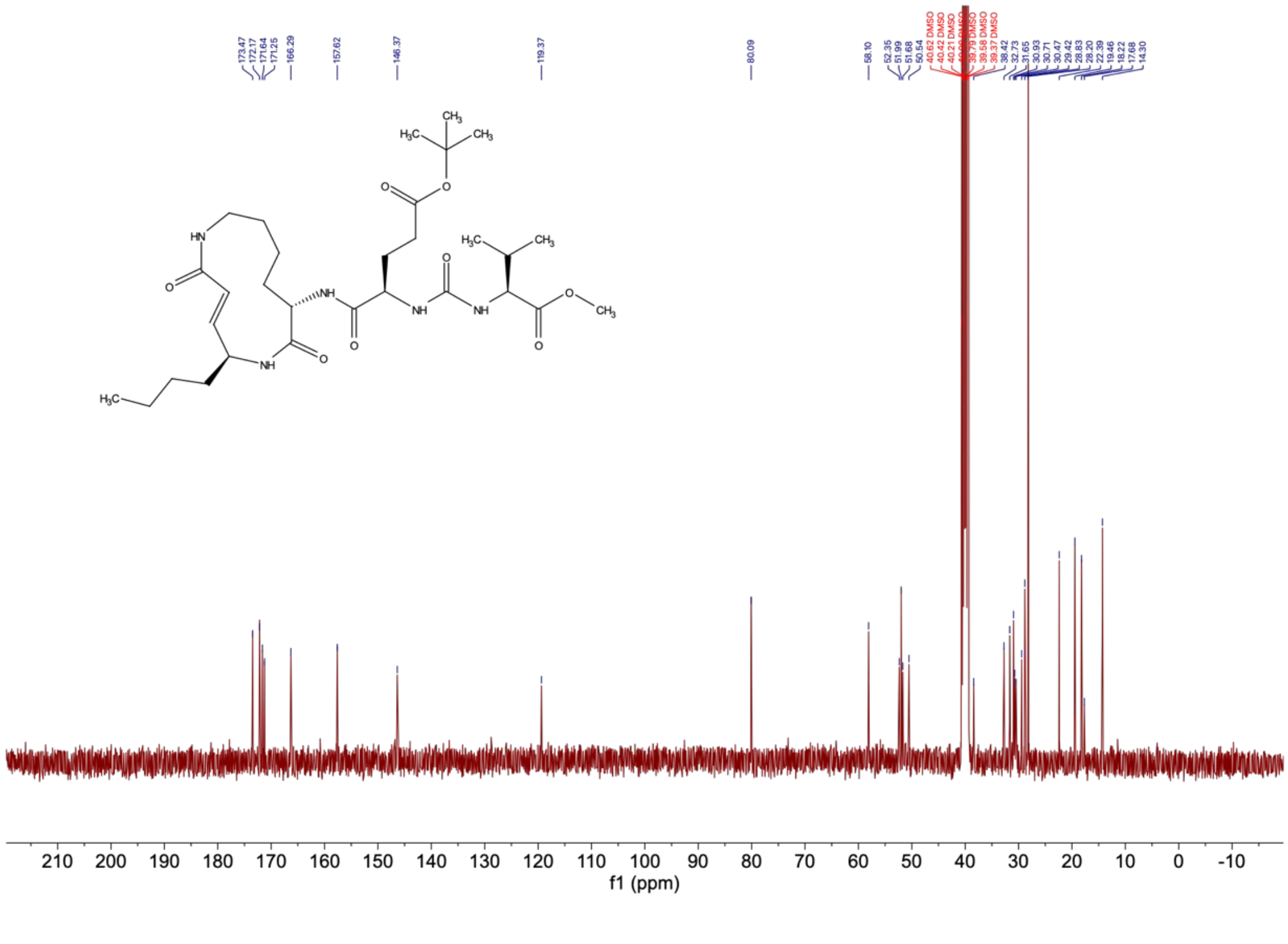

210 200 190 180 170 160 150 140 130 120 110 100 90 80 70 60 50 40 30 20 10 0 -10
f1 (ppm)

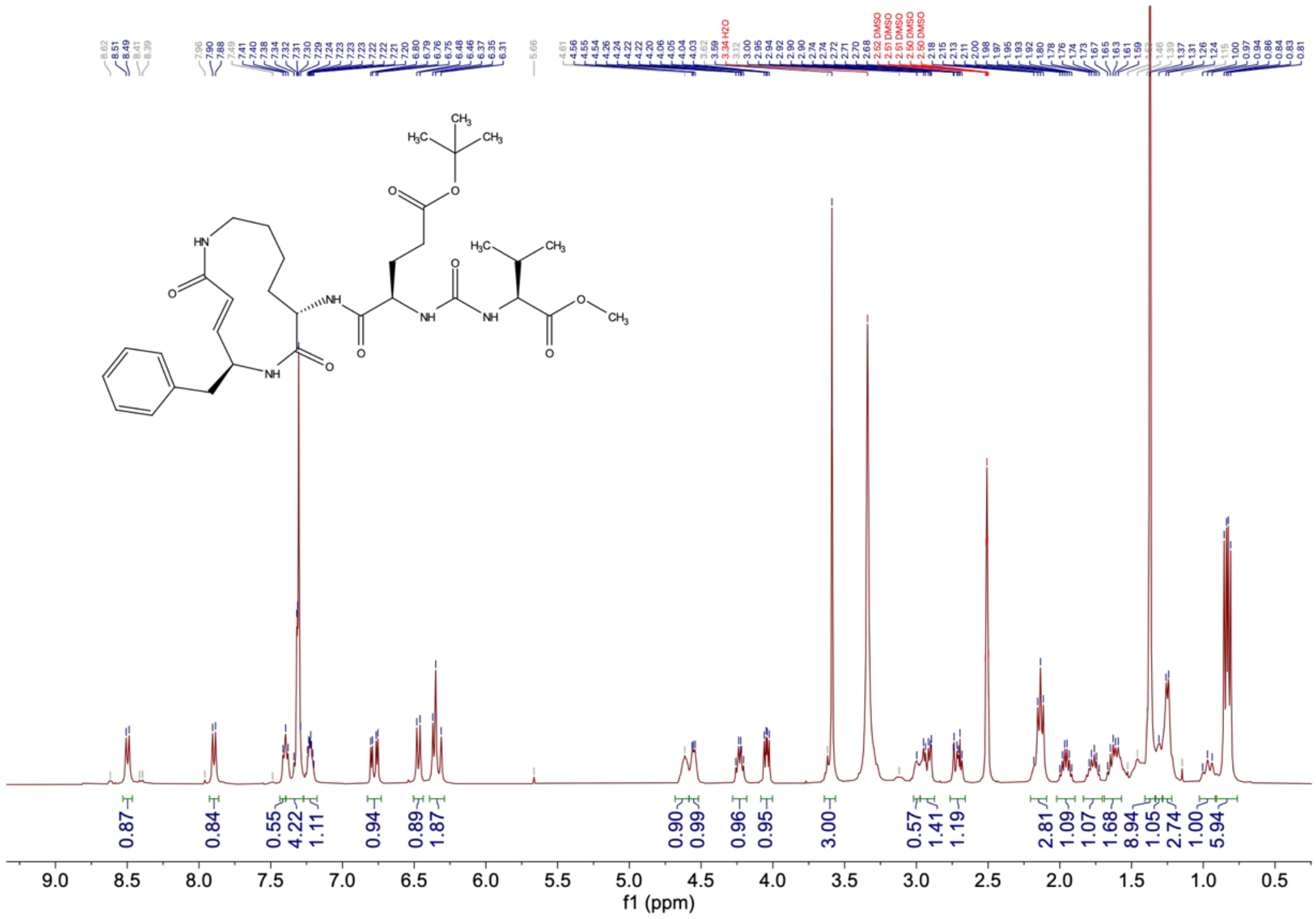

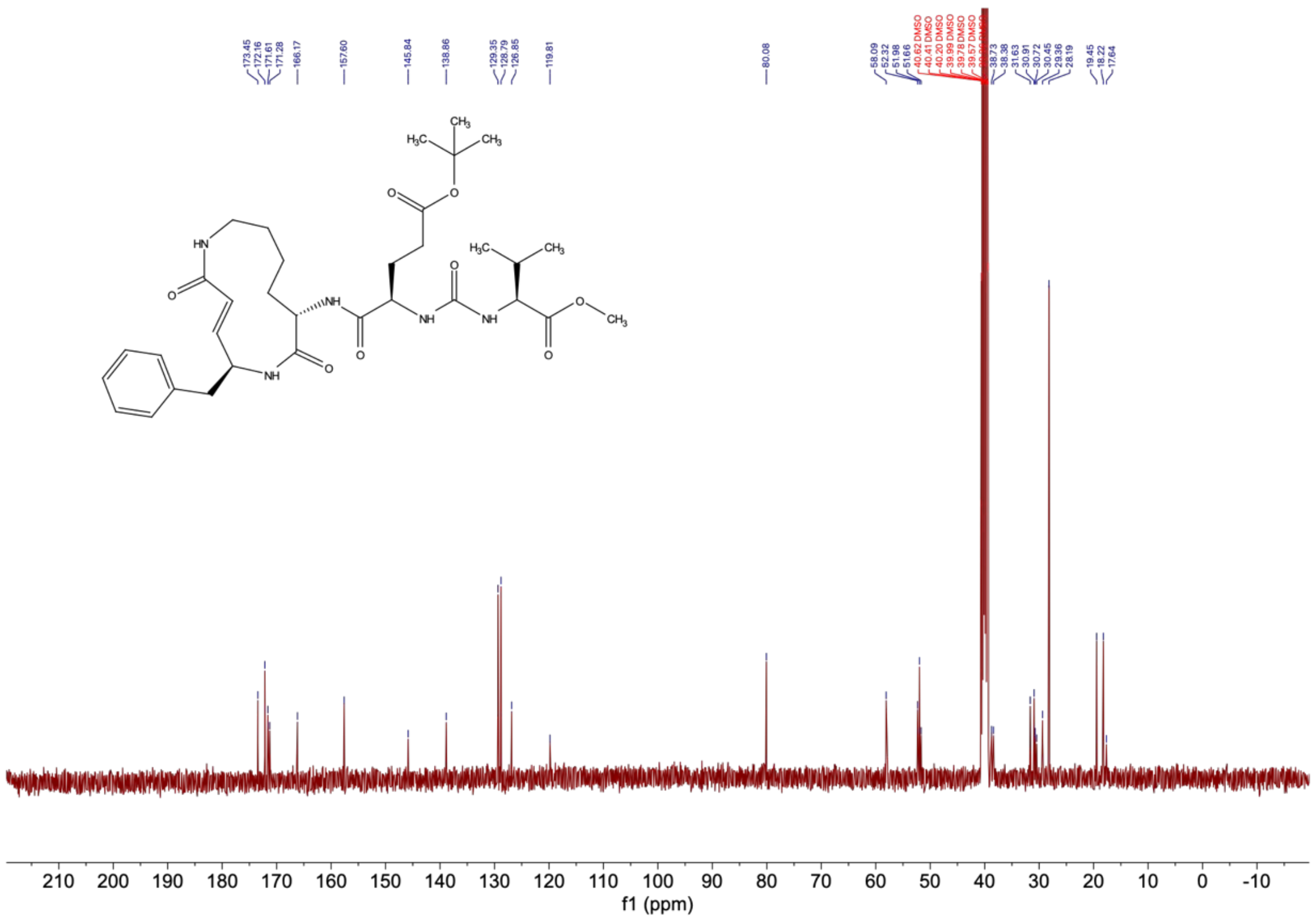

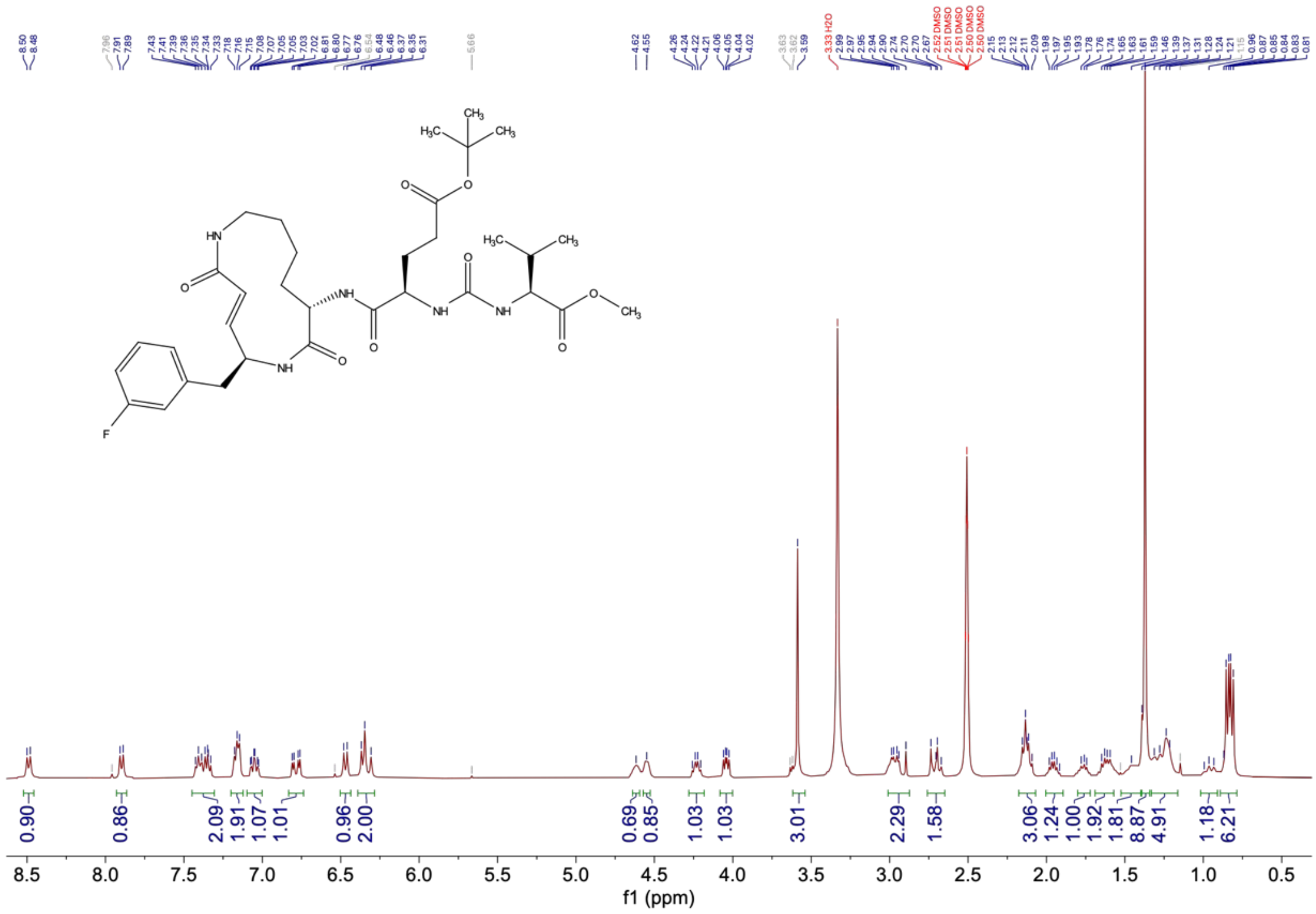

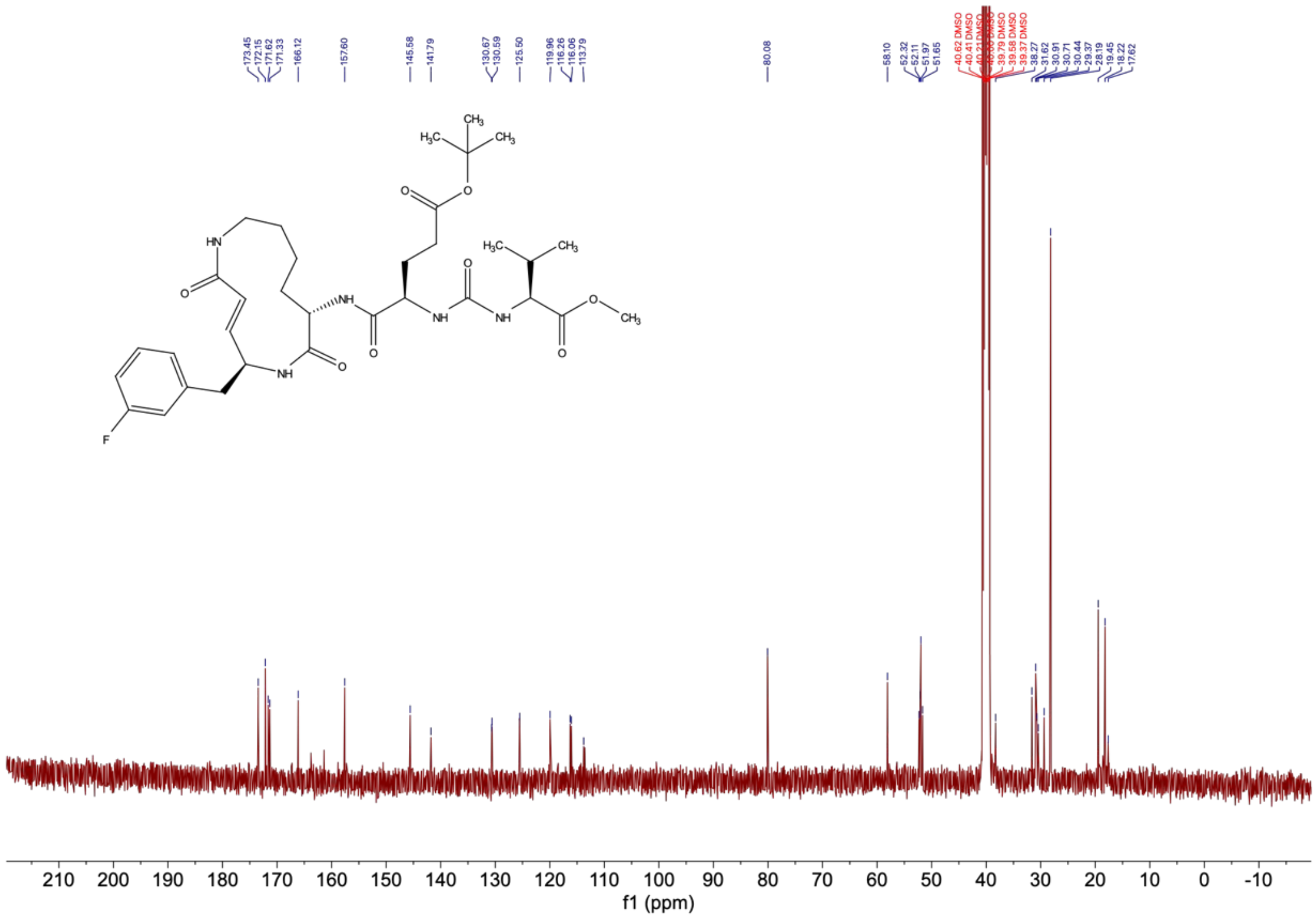

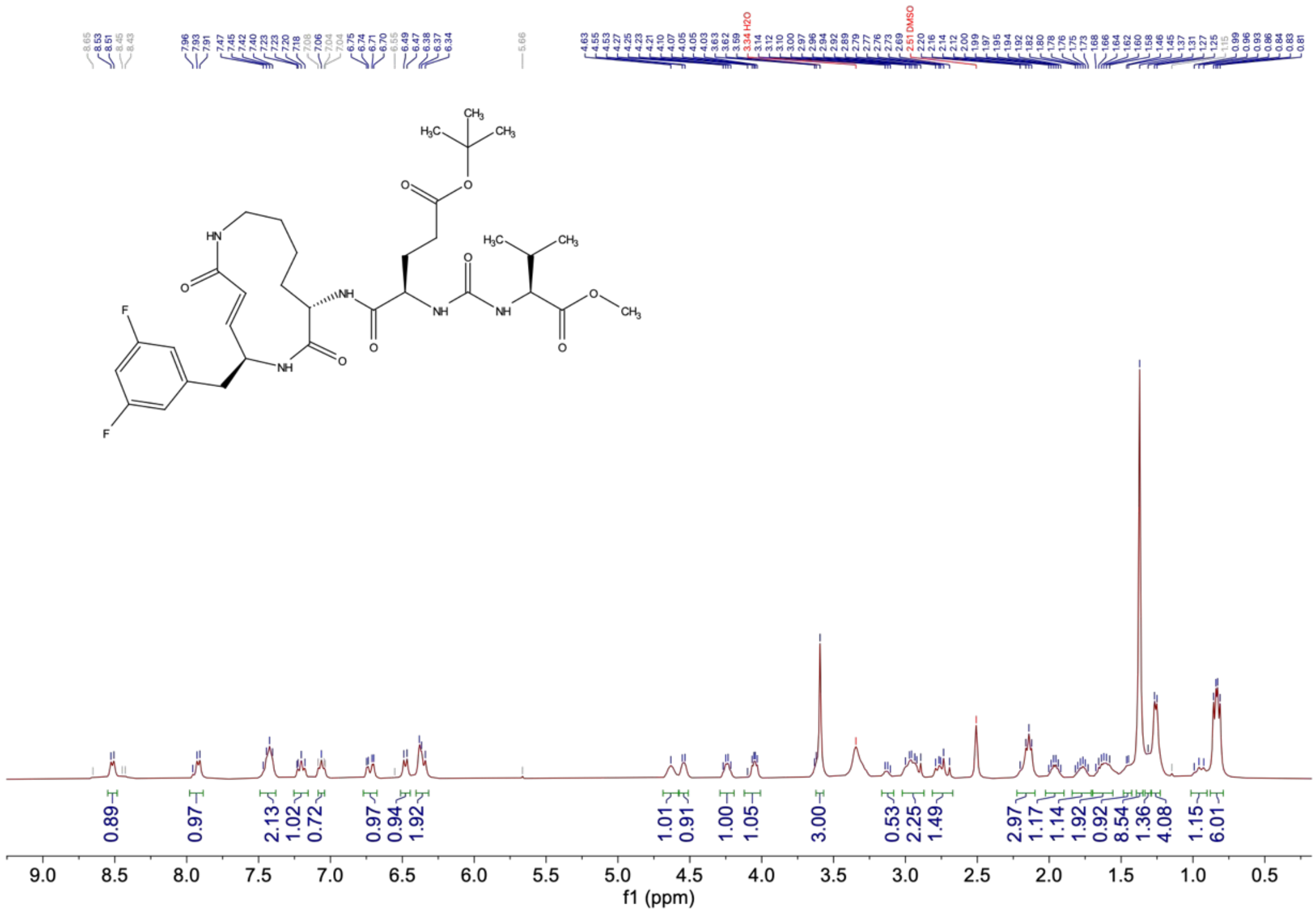

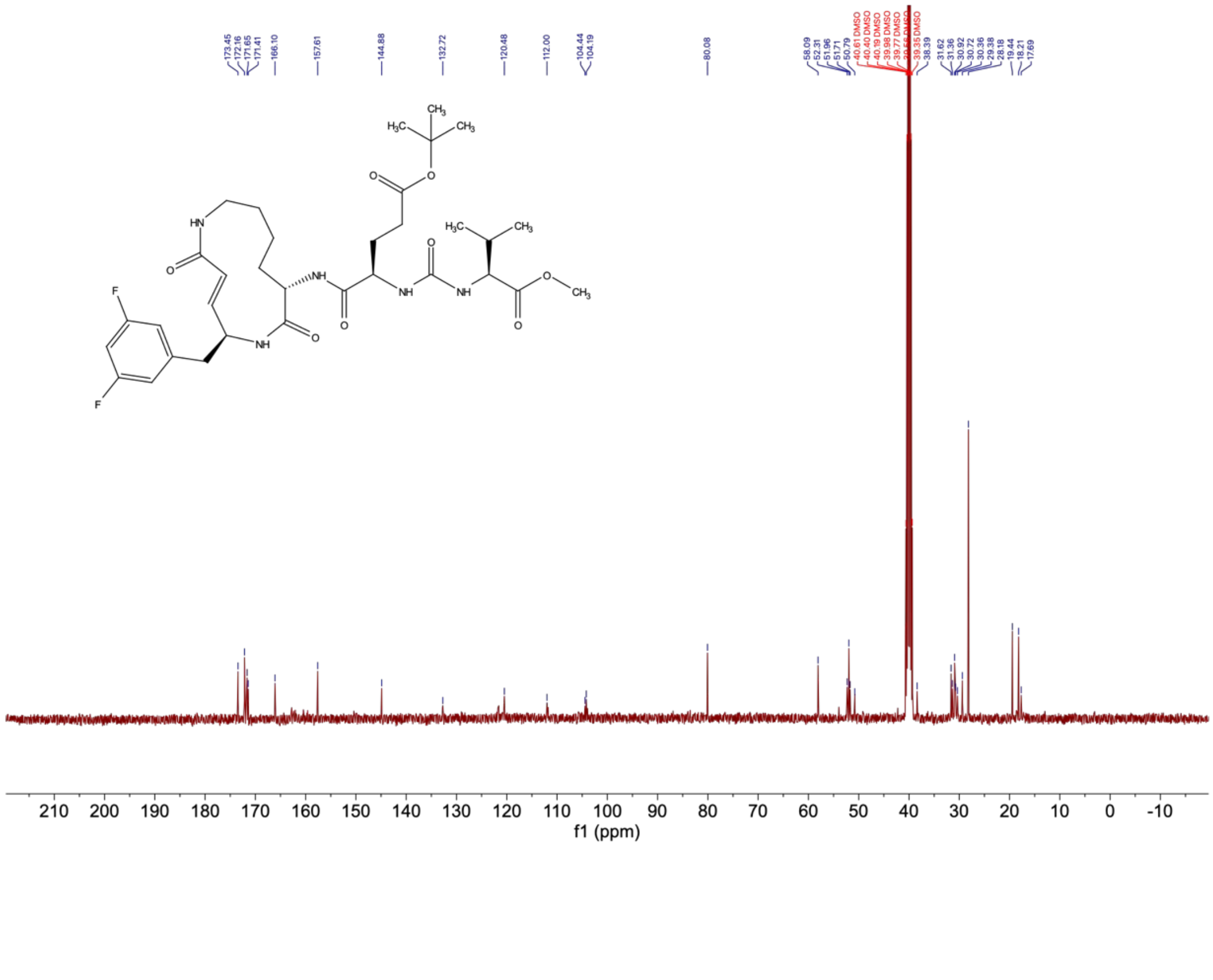

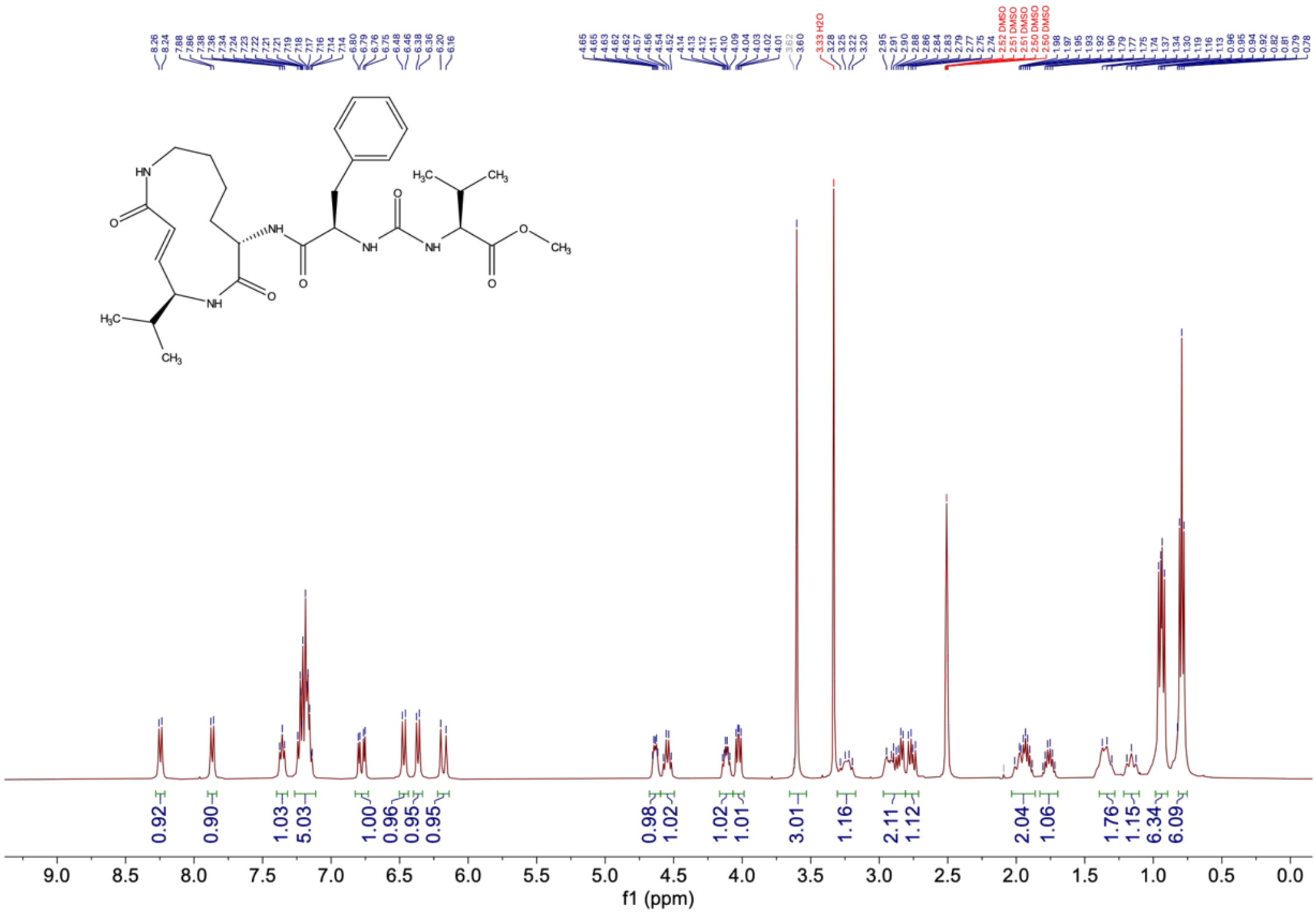
0.92
0.90
1.03
5.03
1.00
0.96
0.95
0.95
0.98
1.02
1.02
1.01
3.01
1.16
2.11
1.12
2.04
1.06
1.76
1.15
6.34
6.09
f1 (ppm)

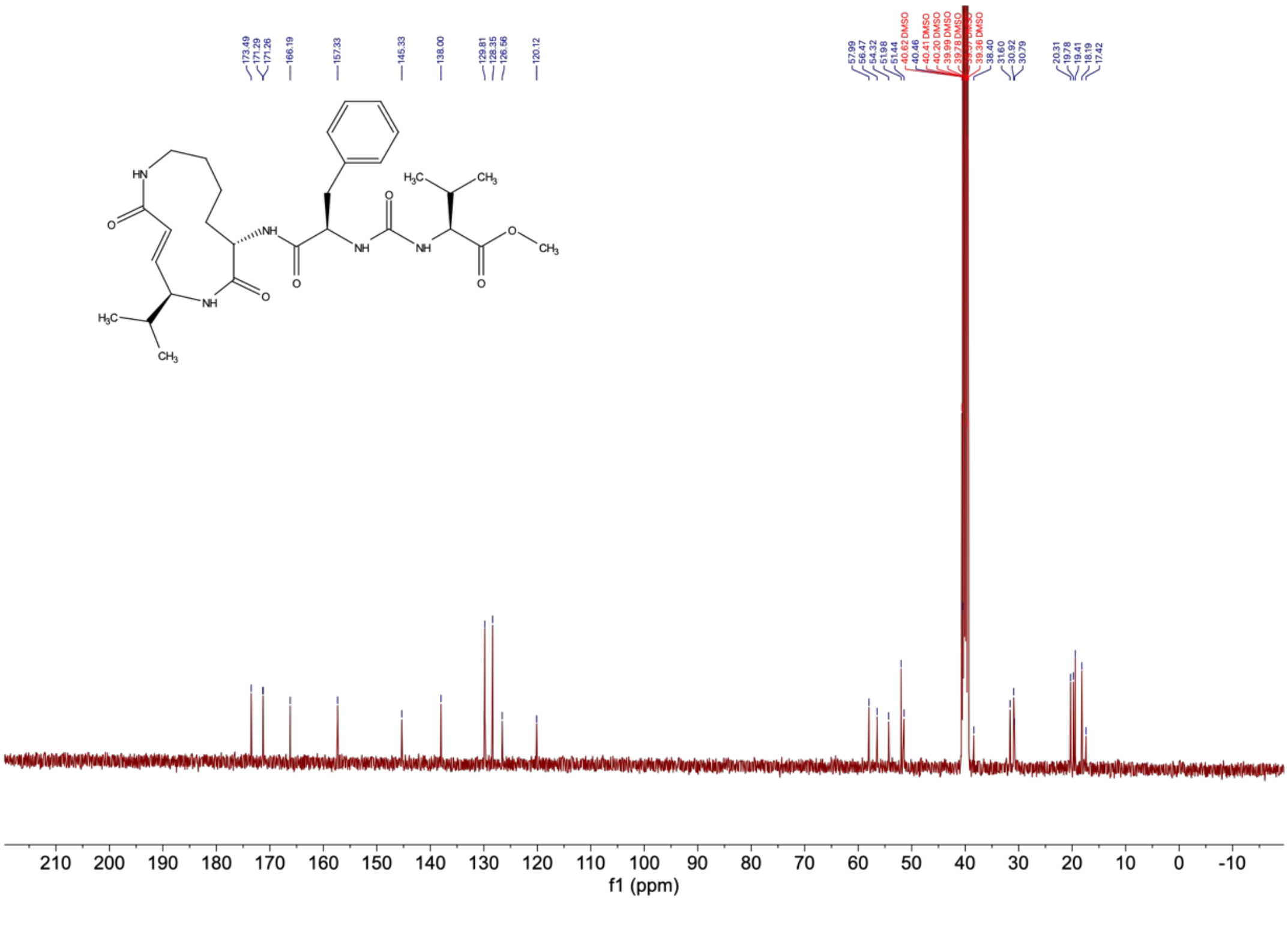
f1 (ppm)

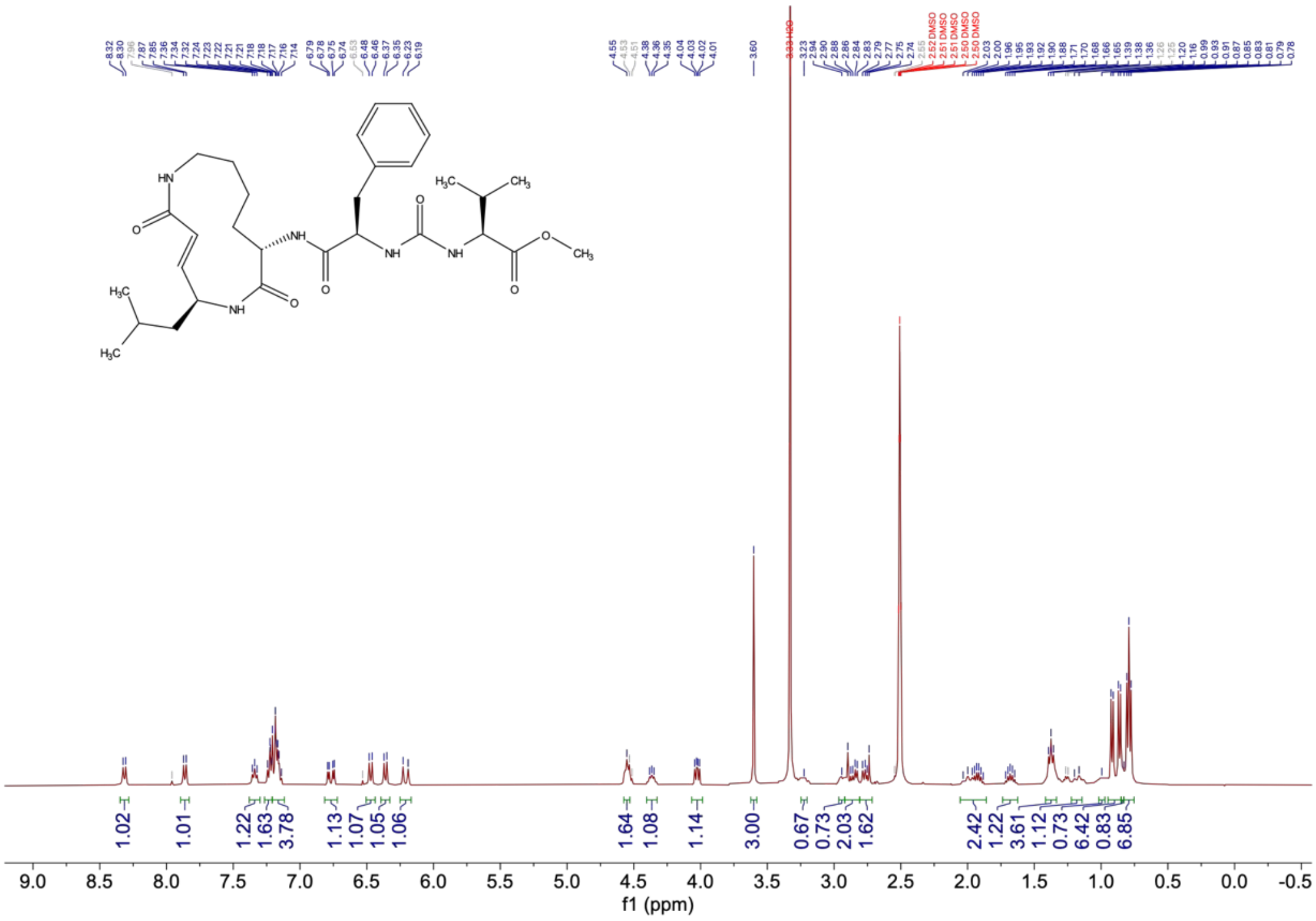

NH
O
NH
O
NH
O
NH
NH
O
O
O
H_3C
CH_3
CH_3
H_3C
H_3C
1.02
1.01
1.22
1.63
3.78
1.13
1.07
1.05
1.06
1.64
1.08
1.14
3.00
0.67
0.73
2.03
1.62
2.42
1.22
3.61
1.12
0.73
6.42
0.83
6.85
9.0 8.5 8.0 7.5 7.0 6.5 6.0 5.5 5.0 4.5 4.0 3.5 3.0 2.5 2.0 1.5 1.0 0.5 0.0 -0.5
f1 (ppm)

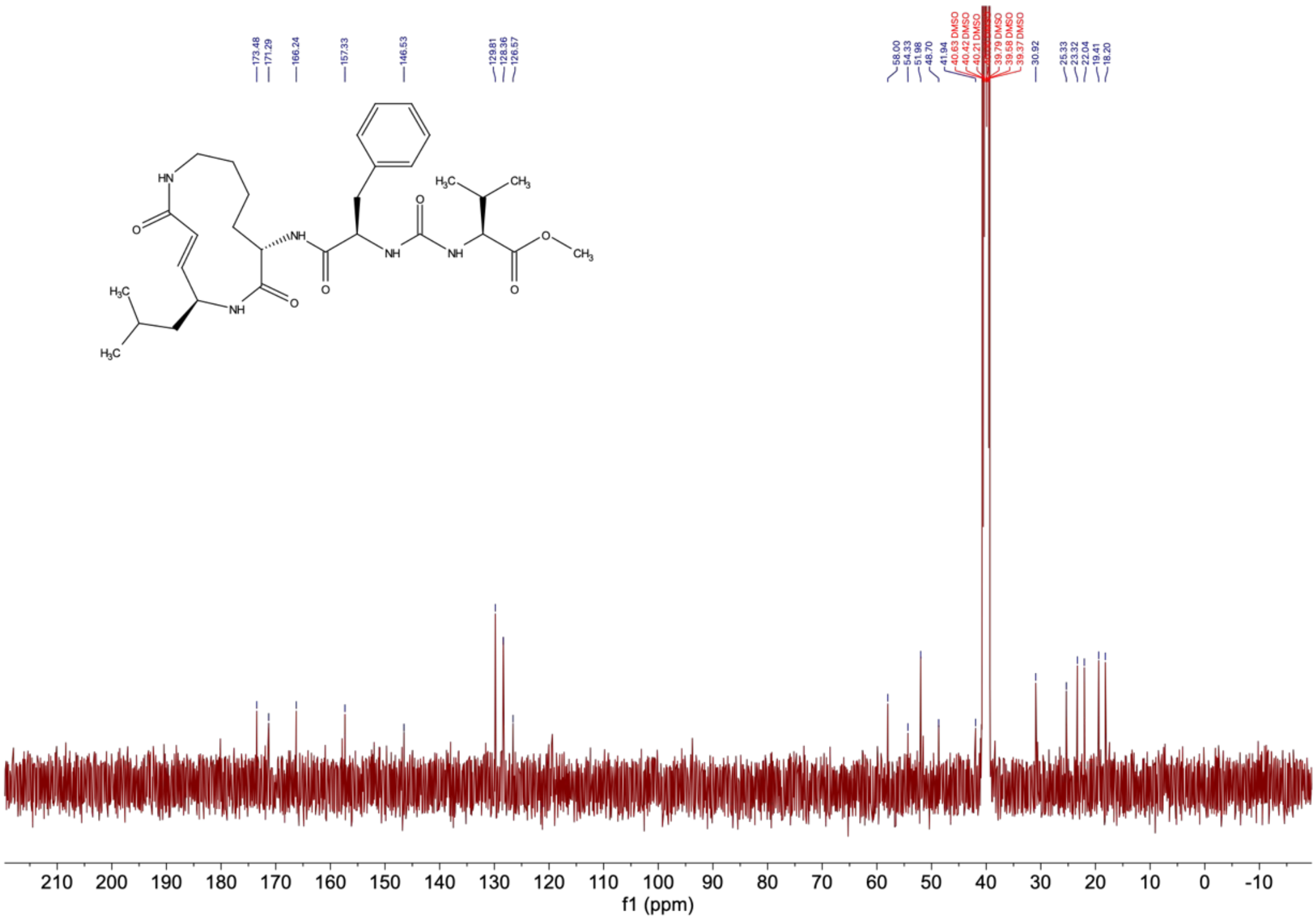

173.48
171.29
166.24
157.33
146.53
129.81
128.36
126.57
58.00
54.33
51.98
48.70
41.94
30.92
25.33
23.32
22.04
19.41
18.20
210 200 190 180 170 160 150 140 130 120 110 100 90 80 70 60 50 40 30 20 10 0 -10
f1 (ppm)

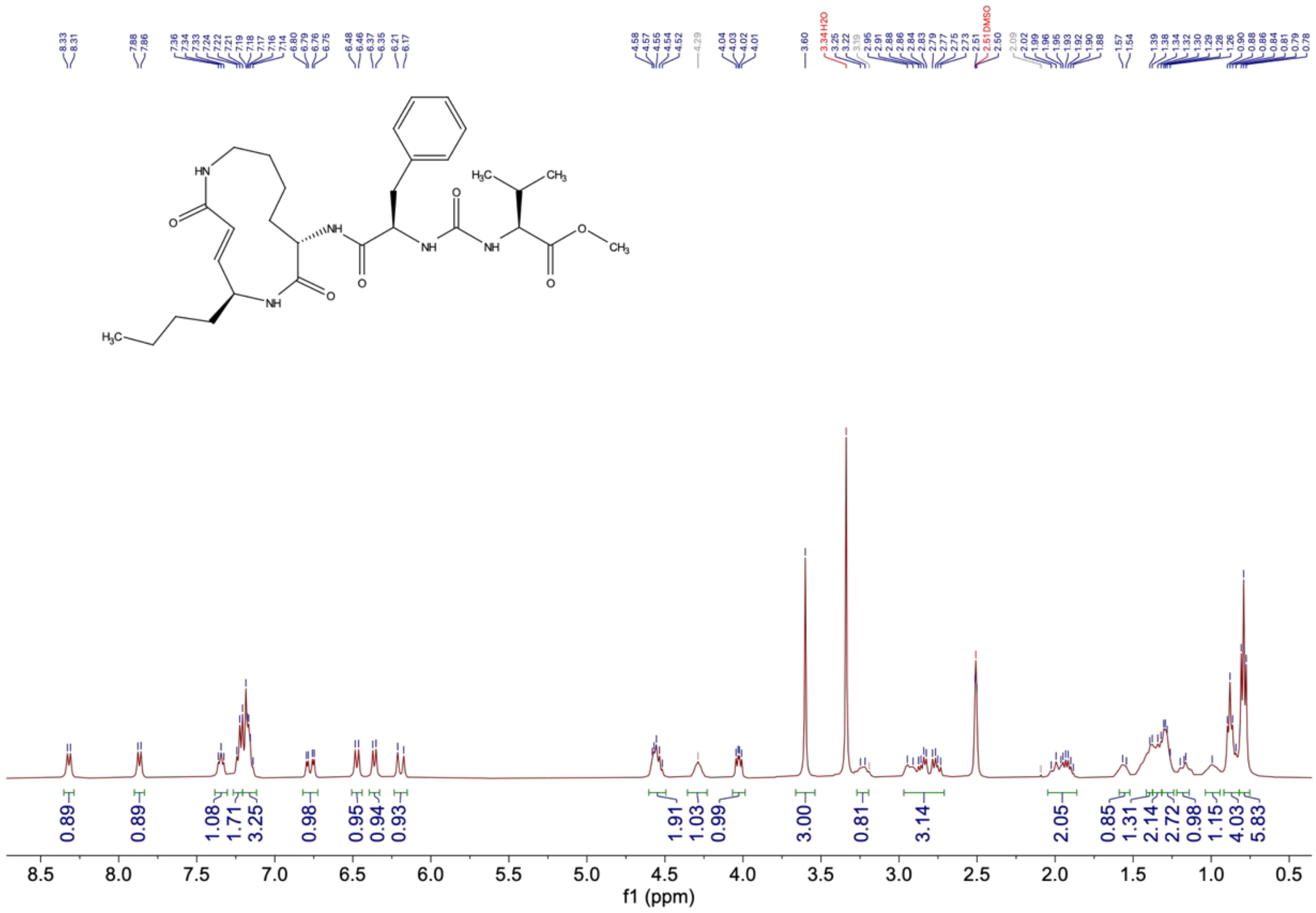

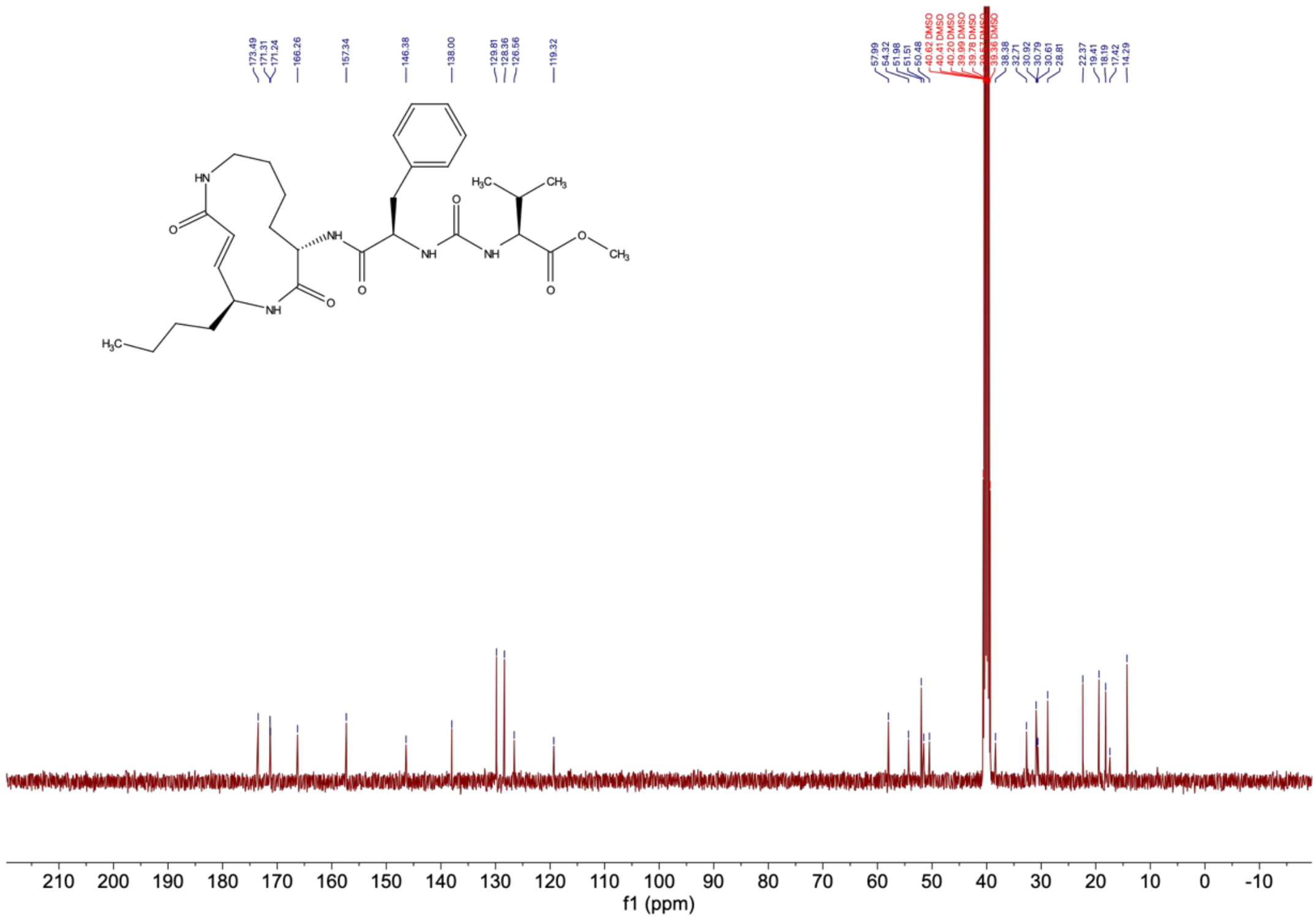

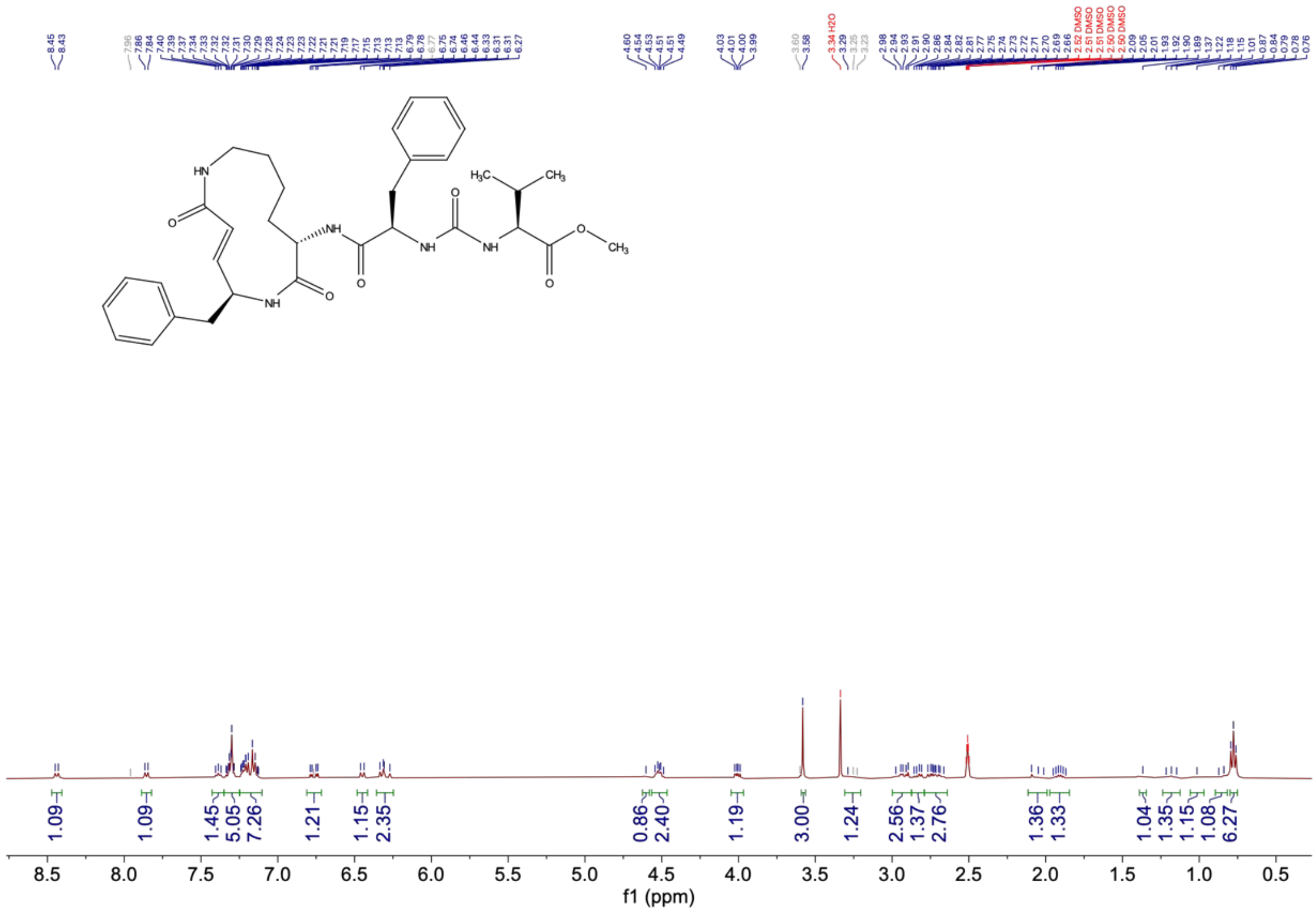

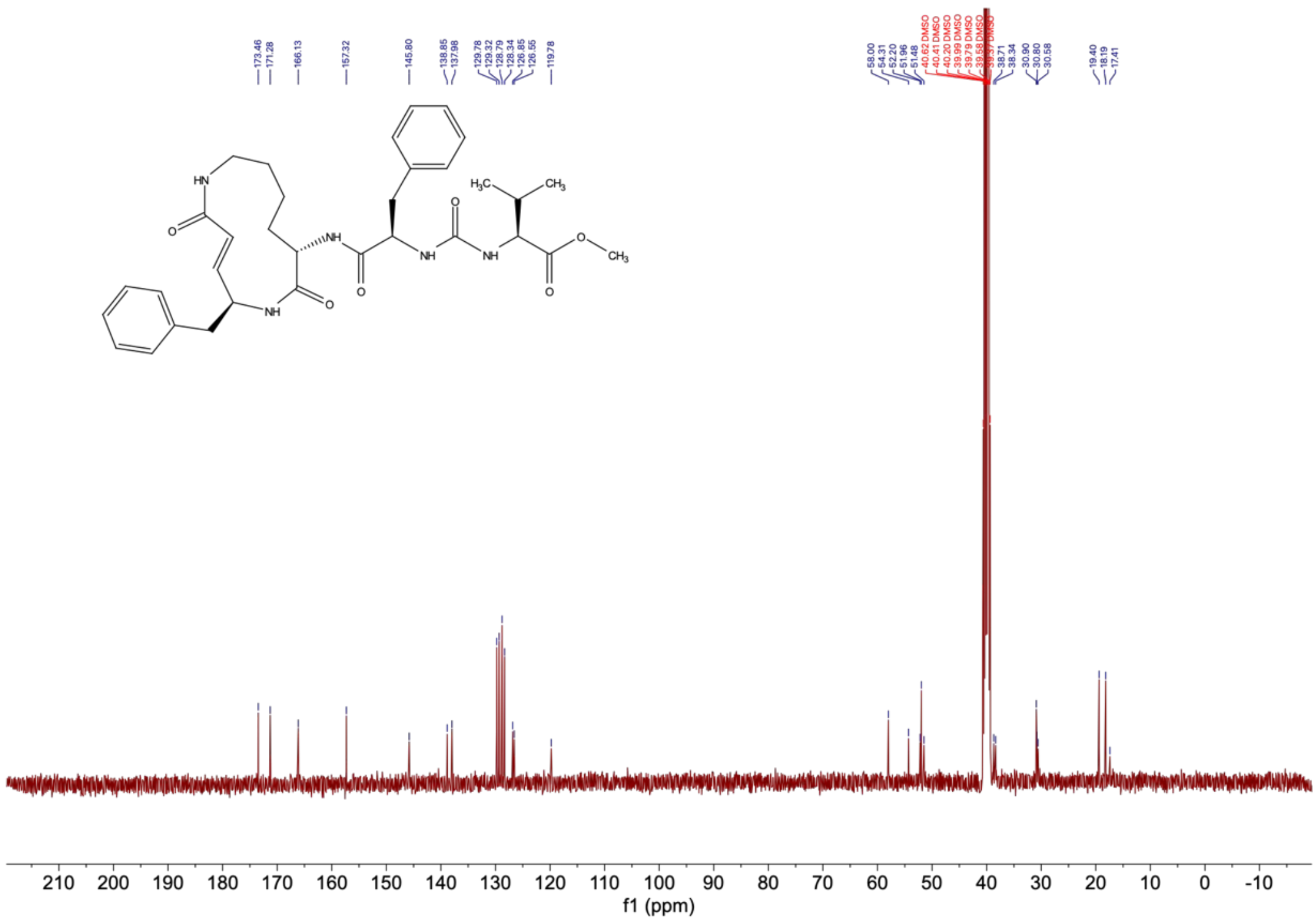

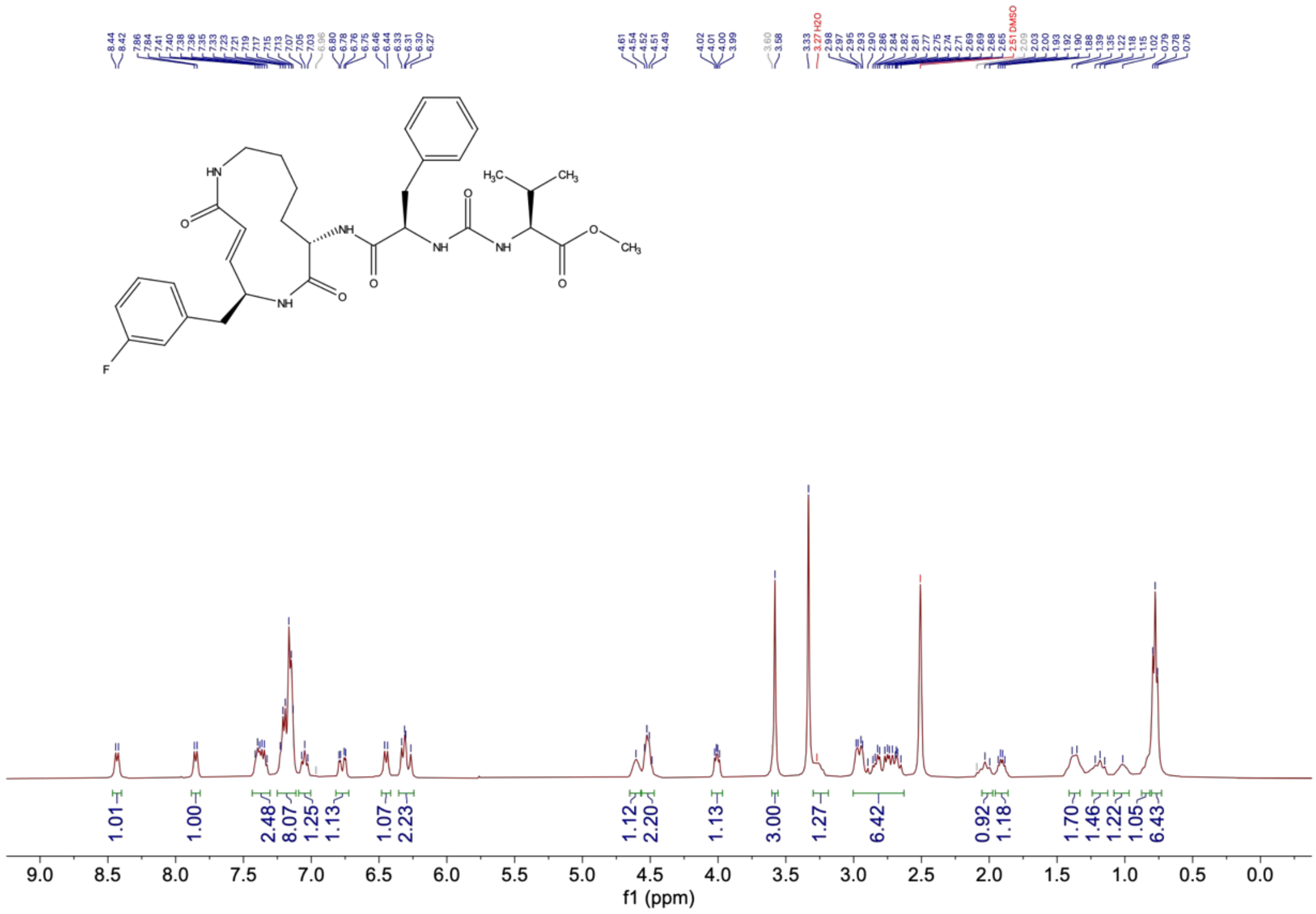

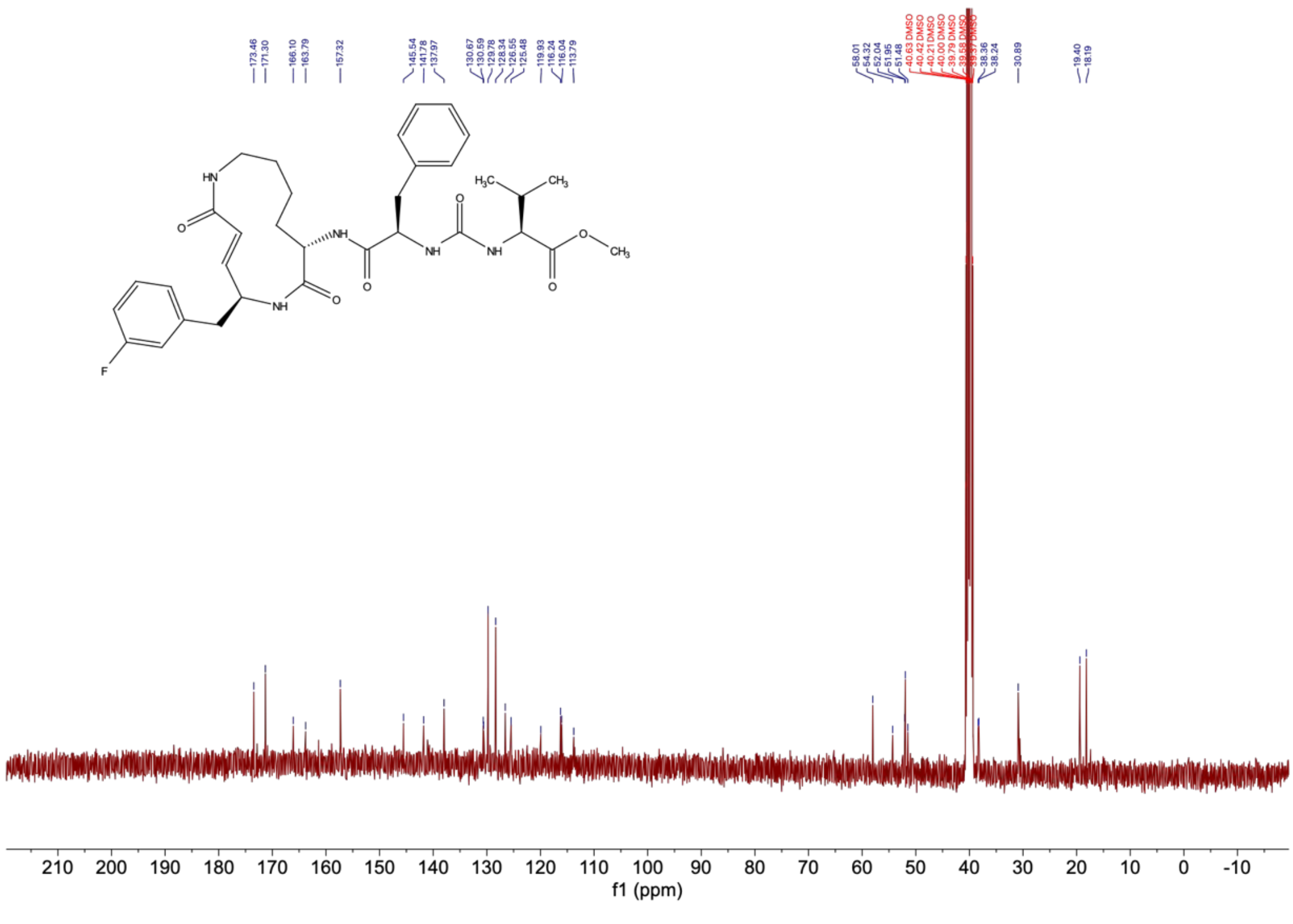

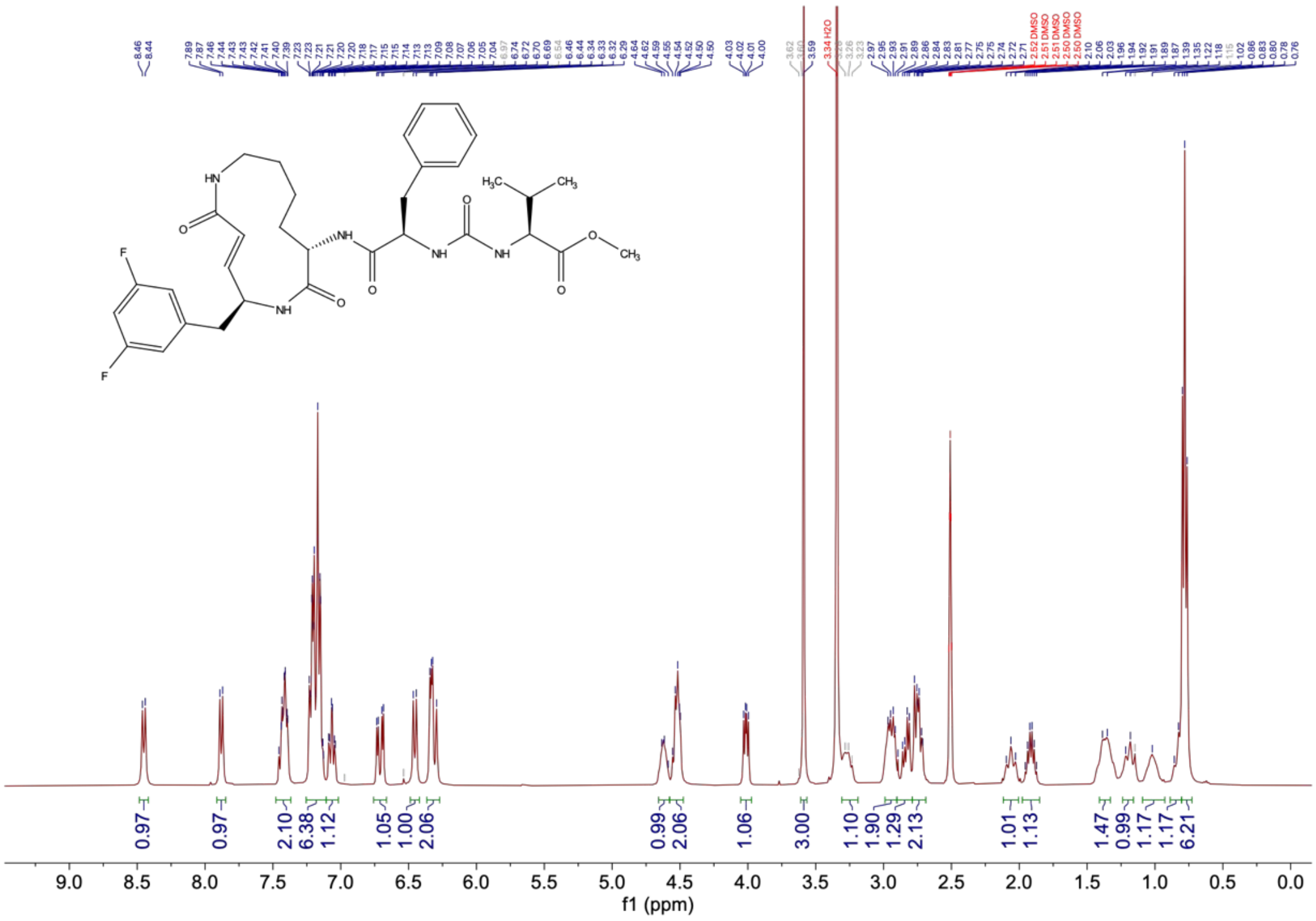

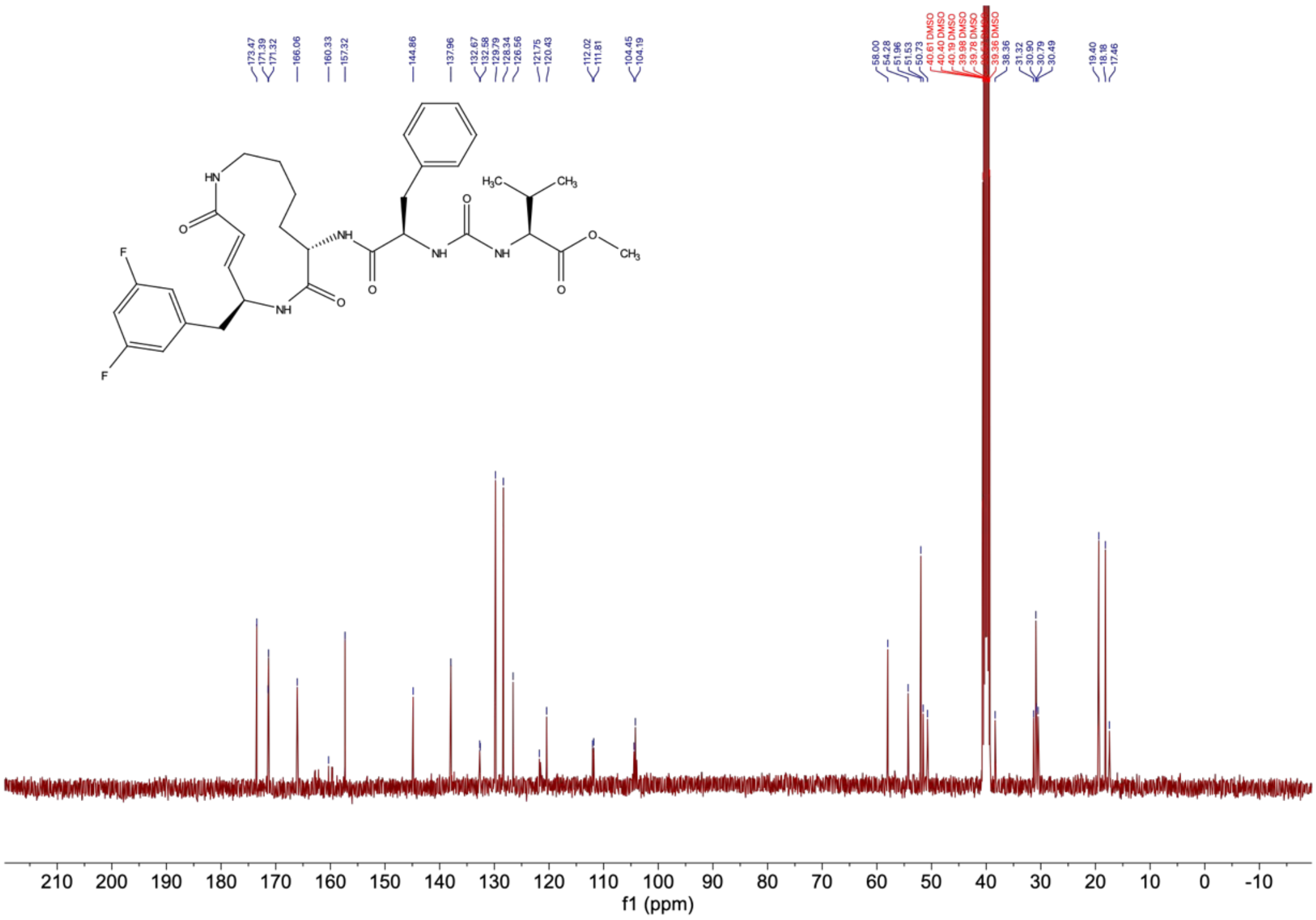

1. Totaro, K. A.; Barthelme, D.; Simpson, P. T.; Sauer, R. T.; Sello, J. K., Substrate-guided optimization of the syringolins yields potent proteasome inhibitors

with activity against leukemia cell lines. *Bioorganic & medicinal chemistry* **2015,** *23* (18), 6218-6222.
2. Silhan, J.; Fajtova, P.; Bartosova, J.; Hurysz, B. M.; Almaliti, J.; Miyamoto, Y.; Eckmann, L.; Gerwick, W. H.; O'Donoghue, A. J.; Boura, E., Structural elucidation of recombinant Trichomonas vaginalis 20S proteasome bound to covalent inhibitors. *Nature communications* **2024,** *15* (1), 8621.
3. Fajtova, P.; Zhang, H.; Urich, L.; da Silva, E. B.; da Silva, C. H.; Almaliti, J.; Al-Hindy, M.; Boura, E.; Fidock, D. A.; Kirkman, L. A., Recombinant proteasome provides new avenues for anti-malarial drug development. *bioRxiv* **2025**.
4. Trivedi, M.; Jana, S., Evaluation of Anti-aging Effect of the Novel Test Formulation in Cell-based Studies using Β-Galactosidase Activity, Collagen Levels, and Protection against Oxidative Stress. *J Cell Biol Histol* **2019,** *2* (1), 101.
5. Bigl, K.; Schmitt, A.; Meiners, I.; Münch, G.; Arendt, T., Comparison of results of the CellTiter Blue, the tetrazolium (3-[4, 5-dimethylthioazol-2-yl]-2, 5-diphenyl tetrazolium bromide), and the lactate dehydrogenase assay applied in brain cells after exposure to advanced glycation endproducts. *Toxicology in vitro* **2007,** *21* (5), 962-971.
6. Sebaugh, J., Guidelines for accurate EC50/IC50 estimation. *Pharmaceutical statistics* **2011,** *10* (2), 128-134.
7. Wang, F.; Liu, Y.; Yu, Z.; Li, S.; Feng, S.; Cheng, Y.; Agard, D. A., General and robust covalently linked graphene oxide affinity grids for high-resolution cryo-EM. *Proceedings of the National Academy of Sciences* **2020,** *117* (39), 24269-24273.
8. Zheng, S. Q.; Palovcak, E.; Armache, J.-P.; Verba, K. A.; Cheng, Y.; Agard, D. A., MotionCor2: anisotropic correction of beam-induced motion for improved cryo-electron microscopy. *Nature Methods* **2017,** *14* (4), 331-332.
9. de la Rosa-Trevin, J. M.; Quintana, A.; Del Cano, L.; Zaldivar, A.; Foche, I.; Gutierrez, J.; Gomez-Blanco, J.; Burguet-Castell, J.; Cuenca-Alba, J.; Abrishami, V.; Vargas, J.; Oton, J.; Sharov, G.; Vilas, J. L.; Navas, J.; Conesa, P.; Kazemi, M.; Marabini, R.; Sorzano, C. O.; Carazo, J. M., Scipion: A software framework toward integration, reproducibility and validation in 3D electron microscopy. *J. Struct. Biol.* **2016,** *195*, 93–99.
10. Punjani, A.; Rubinstein, J. L.; Fleet, D. J.; Brubaker, M. A., cryoSPARC: algorithms for rapid unsupervised cryo-EM structure determination. *Nature Methods* **2017,** *14* (3), 290-296.
11. Meng, E. C.; Goddard, T. D.; Pettersen, E. F.; Couch, G. S.; Pearson, Z. J.; Morris, J. H.; Ferrin, T. E., UCSF ChimeraX: Tools for structure building and analysis. *Protein Science* **2023,** *32* (11), e4792.
12. Long, D. L.; Burkholder, E.; Cronin, L., Polyoxometalate clusters, nanostructures and materials: from self assembly to designer materials and devices. *Chem. Soc. Rev.* **2007,** *36*, 105-121.
13. Agirre, J.; Atanasova, M.; Bagdonas, H.; Ballard, C. B.; Baslé, A.; Beilsten-Edmands, J.; Borges, R. J.; Brown, D. G.; Burgos-Mármol, J. J.; Berrisford, J. M.; Bond, P. S.; Caballero, I.; Catapano, L.; Chojnowski, G.; Cook, A. G.; Cowtan, K. D.; Croll, T. I.; Debreczeni, J.; Devenish, N. E.; Dodson, E. J.; Drevon, T. R.;

Emsley, P.; Evans, G.; Evans, P. R.; Fando, M.; Foadi, J.; Fuentes-Montero, L.; Garman, E. F.; Gerstel, M.; Gildea, R. J.; Hatti, K.; Hekkelman, M. L.; Heuser, P.; Hoh, S. W.; Hough, M. A.; Jenkins, H. T.; Jiménez, E.; Joosten, R. P.; Keegan, R. M.; Keep, N.; Krissinel, E. B.; Kolenko, P.; Kovalevskiy, O.; Lamzin, V. S.; Lawson, D. M.; Lebedev, A. A.; Leslie, A. G. W.; Lohkamp, B.; Long, F.; Malý, M.; McCoy, A. J.; McNicholas, S. J.; Medina, A.; Millán, C.; Murray, J. W.; Murshudov, G. N.; Nicholls, R. A.; Noble, M. E. M.; Oeffner, R.; Pannu, N. S.; Parkhurst, J. M.; Pearce, N.; Pereira, J.; Perrakis, A.; Powell, H. R.; Read, R. J.; Rigden, D. J.; Rochira, W.; Sammito, M.; Sánchez Rodríguez, F.; Sheldrick, G. M.; Shelley, K. L.; Simkovic, F.; Simpkin, A. J.; Skubak, P.; Sobolev, E.; Steiner, R. A.; Stevenson, K.; Tews, I.; Thomas, J. M. H.; Thorn, A.; Valls, J. T.; Uski, V.; Usón, I.; Vagin, A.; Velankar, S.; Vollmar, M.; Walden, H.; Waterman, D.; Wilson, K. S.; Winn, M. D.; Winter, G.; Wojdyr, M.; Yamashita, K., The CCP4 suite: integrative software for macromolecular crystallography. *Acta Crystallogr D Struct Biol* **2023,** *79* (Pt 6), 449-461.

14. Emsley, P.; Lohkamp, B.; Scott, W. G.; Cowtan, K., Features and development of Coot. *Acta Crystallogr D Biol Crystallogr* **2010,** *66* (Pt 4), 486-501.

15. Croll, T. I., ISOLDE: a physically realistic environment for model building into low-resolution electron-density maps. *Acta Crystallogr D Struct Biol* **2018,** *74* (Pt 6), 519-530.
